# Cross-trait genomic and sequential analyses of multiple omics datasets identified shared genetic components for the gut–eye axis

**DOI:** 10.1186/s40246-026-00947-6

**Published:** 2026-03-17

**Authors:** Yuelan Gao, Jianming Xu, Jun Yu, Yuyao Wang, Yuzhou Zhang, Ka Wai Kam, Mary Ho, Alvin L. Young, Chi Pui Pang, Clement C. Tham, Jason C. Yam, Li Jia Chen

**Affiliations:** 1 Department of Ophthalmology and Visual Sciences, The Chinese University of Hong Kong, Sha Tin, Hong Kong China; 2 Department of Ophthalmology and Visual Sciences, Prince of Wales Hospital, Hong Kong, China; 3 Hong Kong Eye Hospital, Hong Kong, China

**Keywords:** Gut–eye axis, Genetic pleiotropy, Gene–environment, Gut microbiota

## Abstract

**Background:**

The gut–eye axis (GEA) has been proposed as a framework for understanding comorbidity between gastrointestinal and ocular diseases. This study aimed to investigate their shared genetic architecture, pleiotropy, and putative biological pathways potentially influenced by environmental exposures and gut microbiota.

**Methods:**

This study integrated large-scale genome-wide association study summary data on five gastrointestinal and eight ocular diseases to assess genetic correlations and genetic overlap. Pleiotropic variants were identified, followed by functional and tissue-specific analyses. Gene–environment (G×E) interactions were evaluated using UK Biobank data. Mendelian randomization (MR) and mediation analyses were adopted to assess statistically inferred associations and potential mediating relationships involving gut microbiota.

**Results:**

Extensive genetic correlations were identified between 40 trait pairs. In total, 366 pleiotropic loci were identified, with 21 loci showing evidence of colocalized shared signals. Notably, 2p21, 4q24, 19q13.32 and 5p15.31 were colocalized across 2 trait pairs, highlighting them as recurrent pleiotropic loci of potential interest. Of the 603 genes associated with pleiotropic variants, 261 recurred across two or more trait pairs. These genes were enriched in immune and inflammatory pathways and included well-known loci such as *HLA-B* and *RBFOX1*. Twenty-six pleiotropic variants interacted with 16 modifiable exposures (e.g., diet, mental health, BMI, smoking, physical activity), suggesting that G×E interactions may contribute to gut–eye comorbidity risk. Bidirectional MR identified 11 genetically predicted associations, while mediation analysis suggested a potential statistical association involving the polyamine biosynthesis pathway in the relationship between gastroesophageal reflux disease and diabetic retinopathy.

**Conclusions:**

This study characterizes shared genetic and environmental architecture across GEA-related disorders and highlights putative contributions from immune-related pathways and gut microbiota to disease comorbidity. Our findings provide a hypothesis-generating framework for future replication and experimental validation.

**Supplementary Information:**

The online version contains supplementary material available at 10.1186/s40246-026-00947-6.

## Introduction

The gut–eye axis (GEA) has been proposed as a framework linking gut dysfunction and inflammation to the development and progression of various ocular diseases through bidirectional interactions between the gastrointestinal and ocular systems [1]. It involves multiple eye conditions, including age-related macular degeneration (AMD), uveitis, diabetic retinopathy (DR), dry eye disease (DED), primary angle-closure glaucoma (PACG), myopia, keratitis, and cataract [2–4]. Proposed biological mechanisms underlying the GEA include immune and inflammatory responses, with the gut microbiota and its metabolic products potentially contributing to these processes [5]. Moreover, accumulating evidence from recent studies has demonstrated significant associations between various gastrointestinal disorders, such as inflammatory bowel disease (IBD) and irritable bowel syndrome (IBS), and ocular diseases, providing novel insights into the GEA and its relevance to systemic comorbidity [6, 7]. However, systematic research elucidating the correlations and underlying molecular mechanisms between these two disease categories remains limited.

Genome-wide association studies (GWAS) are a crucial tool for uncovering the genetic basis of complex diseases [8]. Growing evidence indicates that genetic pleiotropy, where single variants affect multiple related diseases, is common across diverse disorders [9]. Identifying such shared genetic architecture may help reveal common susceptibility loci and biological pathways underlying cross-organ comorbidity. However, existing studies have largely lacked systematic cross-trait analyses to assess genetic correlations and pleiotropy between gastrointestinal and ocular disorders. Consequently, the extent of shared genetic determinants between these disease groups remains unclear.

Environmental factors also play a crucial role in gastrointestinal and ocular diseases. Factors such as air pollution [10, 11], dietary patterns [12, 13], lifestyle choices [14, 15], and socioeconomic status [16, 17] have all been shown to be closely associated with these diseases. Meanwhile, genetic susceptibility is significantly modulated by environmental exposures [18]. In recent years, gene–environment interactions (G×E) have increasingly been investigated in complex diseases to better understand variability in disease susceptibility and to inform more targeted prevention strategies [19]. Nevertheless, systematic studies investigating the interactions between environmental factors and key genetic variants involved in comorbid gastrointestinal and ocular diseases remain limited. Additionally, although the gut microbiota is implicated in both disease types, its genetic regulatory networks and potential causal links remain poorly understood [20].

Given the potential clinical and biological links between gastrointestinal and ocular diseases, this study systematically analyzed their genetic correlations and pleiotropy. We further integrated multi-omics approaches, including transcriptome- and proteome-wide association analyses, and incorporated environmental and gut microbiota assessments to explore putative pathways through which genetic susceptibility may relate to gut–eye comorbidity. Our study provides a broad, hypothesis-generating framework for future mechanistic and translational research.

## Methods

### GWAS dataset

GWAS summary statistics were retrieved from European-ancestry datasets. Ocular disease GWAS data (AMD, DR, DED, uveitis, myopia, cataract, PACG, and keratitis) and gastrointestinal disease GWAS data [gastroesophageal reflux disease (GORD), IBD, peptic ulcer disease (PUD), IBS, and diverticular disease (DD)] were obtained from publicly available sources [1, 21–23]. The five gastrointestinal diseases were selected based on previous literature on pleiotropy, aiming to capture a spectrum of conditions with potential shared genetic architecture [24]. The eight ocular diseases were chosen based on prior reports linking gut microbiota to eye disorders [25–27]. The GWAS data for gut microbiota included 412 distinct features, comprising 207 taxonomies (5 phyla, 10 classes, 13 orders, 26 families, 48 genera and 105 species) and 205 microbial pathways, derived from 7,738 participants [28]. All analyses were conducted at the summary level to minimize cohort-specific confounding. To address heterogeneity across data sources, all GWAS summary statistics were restricted to individuals of European ancestry and aligned to the GRCh37 (hg19) reference genome. Analyses were conducted using consistent quality control (QC) procedures. QC procedures included the removal of duplicate or missing rsIDs, multi-allelic SNPs, SNPs with ambiguous strand orientation, variants with minor allele frequency (MAF) < 1%, and SNPs mismatched to the 1000 Genomes Project reference. Disease definitions used in the GWAS analyses are summarized in Table S1.

### Individual-level data from the UKB cohort

The UK Biobank is a population-based cohort study that recruited over 500,000 adults aged 40 to 69 years from 22 assessment centers [29]. Samples were excluded if they met any of the following criteria: (i) outliers for genotype missingness or excess heterozygosity; (ii) showed genetic relatedness; (iii) were of non-European ancestry; or (iv) had missing disease diagnosis. After filtering, 394,616 participants were included in the G×E interaction analyses.

### Genetic correlation and genetic overlap

We assessed the genome-wide genetic correlations across the 40 trait pairs (5 gastrointestinal × 8 ocular diseases) using linkage disequilibrium score regression (LDSC) and high-definition likelihood (HDL) methods [30, 31]. The LDSC cross-trait intercept indicates potential sample overlap. As genetic correlation reflects only average genome-wide associations, we further examined the overall genetic overlap between traits using Genetic analysis incorporating Pleiotropy and Annotation (GPA) [32]. GPA is a statistical framework that jointly analyzes multiple GWAS datasets and functional annotation information, increasing power to detect shared genetic signals [33]. Unlike LDSC or HDL, which only estimates genome-wide correlations, GPA can identify weaker pleiotropic effects and provide interpretable model parameters for hypothesis testing of pleiotropy and annotation enrichment. The Bonferroni-corrected significance threshold was set at *P* < 1.25 × 10⁻³ (0.05/40 trait pairs).

### Identification of pleiotropic loci

For trait pairs with significant genetic correlation or genetic overlap, we used Pleiotropic Analysis under Composite Null Hypothesis (PLACO) to identify potential pleiotropic single-nucleotide polymorphisms (SNPs) [34]. Variants with *P* < 5 × 10⁻⁸ were considered to exhibit significant pleiotropy.

### Functional mapping and colocalization of pleiotropic loci

We applied the Functional Mapping and Annotation (FUMA) analysis to functionally annotate and localize potential pleiotropic loci [35]. Functional annotations, including ANNOVAR software tool categories were also provided by FUMA [36]. To assess whether these loci contained colocalized shared signals, we performed Bayesian colocalization analysis [37]. A posterior probability for hypothesis 4 (PP.H4) greater than 0.7 was considered evidence supporting a shared underlying signal [24].

### Functional enrichment of genes associated with pleiotropic variants

Based on the pleiotropic loci identified by PLACO, we performed gene-level multi-marker analysis of GenoMic annotation (MAGMA) to identify genes associated with pleiotropic variants [38]. Significant genes were defined as those with Bonferroni-corrected *P* < 0.05. Subsequently, we conducted multi-level functional enrichment analyses to annotate significant genes to pathways and biological processes, using the Gene Ontology (GO) and Kyoto Encyclopedia of Genes and Genomes (KEGG) databases [39, 40]. We further applied Gene Set Enrichment Analysis (GSEA, R package clusterProfiler) [41], Tissue-Specific Expression Analysis (TSEA, R package TissueEnrich) [42], and Cell-Type Specific Expression Analysis (CSEA, online WebCSEA platform) to explore potential shared biological pathways of genes associated with pleiotropic variants [43]. For GSEA and TSEA, a false discovery rate (FDR)–adjusted *P*-value < 0.05 was considered statistically significant. CSEA annotated across 1,355 cell types, and a *P*-value reaching the Bonferroni-corrected threshold of 3.69 × 10⁻⁵ (0.05/1,355) was deemed statistically significant.

### Transcriptome- and proteome-wide association studies

To further assess the effect of candidate gene expression implicated by pleiotropic variants, we performed Transcriptome-Wide Association Studies (TWAS) using the FUSION software, integrating GTEx v8 multi-tissue expression data from 14 relevant normal tissues associated with the eye or gastrointestinal diseases [44]. To explore protein-level associations, we conducted proteome-wide association studies (PWAS) using precomputed cis-pQTL weights from the INTERVAL study comprising 3,153 plasma proteins [45]. Genes with a FDR–adjusted *P*-value < 0.05 were considered statistically significant.

### Gene–Environment interaction analysis of pleiotropic variants in UK Biobank

To further assess whether pleiotropic variants statistically interacted with environmental exposures in relation to gastrointestinal–ocular disease risk, we performed G×E interaction analyses using UK Biobank data. The analysis included 64 modifiable environmental exposures derived from baseline questionnaire data, physical measurements, and linked environmental records, encompassing lifestyle factors, dietary intake, air pollutants, traffic-related air pollution, mental health, and antibiotic exposure. The selection, coding, and quality control procedures for these exposures followed those described in a previously published study by You et al. [46]. First, logistic regression models (1 = comorbidity cases, 0 = controls) were applied to assess associations between each environmental exposure and gastrointestinal–ocular disease comorbidity. Exposures showing significant associations (*P*_FDR_ < 0.05) were then included in multi-interaction analyses with pleiotropic genetic variants using the formula:

$$\begin{gathered} logit{\text{ }}P{\text{ }}\left( {Y|G,{\text{ }}E} \right){\text{ }} = \hfill \\ {\beta}_{0} {\mkern 1mu} + {\mkern 1mu} {\beta}_{G} G{\mkern 1mu} + {\mkern 1mu} {\beta}_{E} E{\mkern 1mu} + {\mkern 1mu} {\beta}_{{Interact}} G \times E{\mkern 1mu} + {\mkern 1mu} Z{\gamma}, \hfill \\ \end{gathered} $$  

Where Y represents eye-gastrointestinal comorbidity, E denotes exposure, G indicates the additively coded genotype of a pleiotropic SNP, β_Interact_ represents the interaction coefficient quantifying the G×E effects, and Z is a vector of covariates with corresponding coefficients γ. The interaction coefficient (β_Interact_) was tested using Wald tests within the logistic regression framework [46]. Gene–environment interactive effects at each locus were corrected for multiple testing using the FDR method to identify significant interactions. All models were adjusted for age, sex, education, assessment center, Townsend Deprivation Index (TDI), and the top 10 principal components to control for potential confounders.

### Mendelian randomization and mediation analysis

**Fig. 1:**
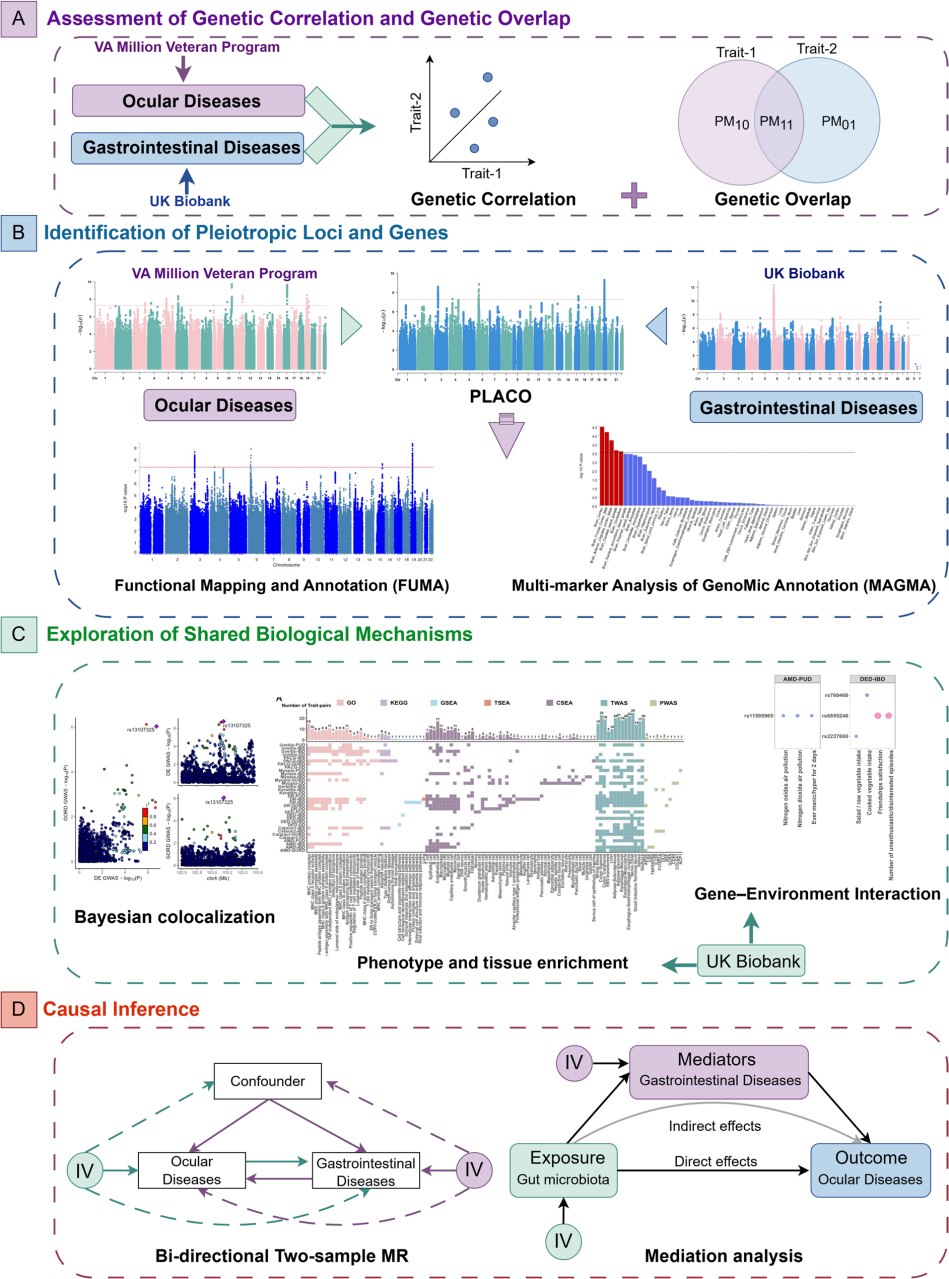
Comprehensive pleiotropy analysis of 5 gastrointestinal and 8 ocular diseases. We assessed genetic correlations and overlaps using GWAS summary data, identified pleiotropic variant loci via PLACO, and performed functional annotation and gene prioritization with FUMA. MAGMA was used to select genes associated with pleiotropic variants, and their tissue- and cell-specific expression characteristics were explored at gene and protein levels. A gene-environment interaction analysis was conducted using UK Biobank data. Finally, bidirectional Mendelian Randomization and mediation analysis were used to evaluate the causal role of gut microbiota in the comorbidity of gastrointestinal and ocular diseases. *GWAS* Genome-Wide Association Study, *PLACO* Pleiotropic Analysis under Composite Null Hypothesis, *GO* Gene Ontology, *KEGG* Kyoto Encyclopedia of Genes and Genomes, *GSEA* Gene Set Enrichment Analysis, *TSEA* Tissue-Specific Expression Analysis, *CSEA* Cell-Type Specific Expression

To further assess genetically predicted associations between ocular and gastrointestinal diseases, we performed bidirectional two-sample Mendelian randomization (MR) analysis [47]. Independent SNPs associated with the exposures at genome-wide significance (*P* < 5 × 10⁻⁸) were clumped for linkage disequilibrium using a clumping distance of 10,000 kb (r² < 0.001) [48]. We adopted this stringent clumping threshold to ensure instrument independence and reduce potential bias due to residual linkage disequilibrium [49]. The inverse variance weighted (IVW) method was used as the primary estimator [50], with robustness evaluated using MR-Egger [51], weighted median [52], weighted mode [53], and simple mode approaches. Horizontal pleiotropy was assessed using the MR-Egger intercept test, and heterogeneity was evaluated with Cochran’s Q statistic [51]. Given growing evidence that gut microbiota may contribute to the pathogenesis of gastrointestinal and ocular diseases, we further integrated MR and mediation analyses, based on significant MR associations, to explore whether gut microbiota-related pathways might be involved in statistically inferred associations among gastrointestinal and ocular diseases. All analyses were performed using R software (version 4.0.3). Figure 1 outlines the overall analytical workflow.

## Results

### Genetic correlations and shared architecture of gut–eye trait pairs

**Fig. 2:**
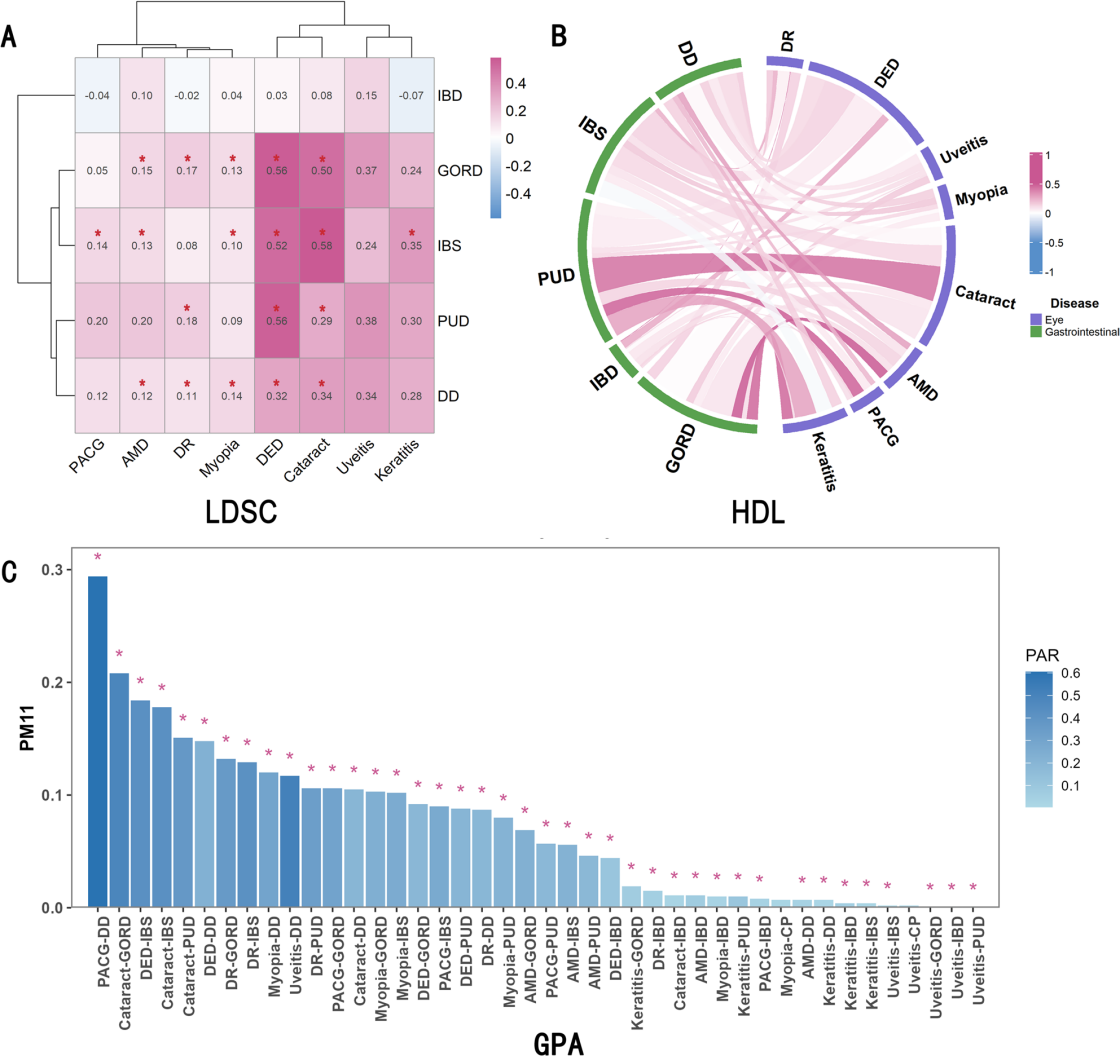
Genetic correlation and genetic overlap between gastrointestinal and ocular diseases. **A** Heatmap of genetic correlations calculated by Linkage Disequilibrium Score Regression (LDSC), illustrating the genetic correlations among 40 pairs of gastrointestinal and ocular disease traits. The color scale represents the magnitude and direction of genetic correlations, with white indicating zero, blue indicating negative correlations, and red indicating positive correlations. Red asterisks denote statistically significant genetic correlations after Bonferroni correction (*P* < 1.25 × 10⁻³, corresponding to 0.05/40 trait pairs) **B** Chord diagram based on high-definition likelihood (HDL) analysis illustrating genetic correlation relationships among gastrointestinal (green) and ocular (purple) diseases. The color gradient reflects the direction and strength of genetic correlations (white = zero, blue = negative, red = positive). **C** Bar plot of genetic overlap derived from the Gene Pleiotropy and Annotation (GPA) model. Asterisks indicate Bonferroni-corrected statistical significance (*P* < 1.25 × 10⁻³). PM₁₁ indicates the proportion of genetic variants associated with both traits; the Pleiotropic Association Ratio (PAR) is defined as PM₁₁ divided by the sum of PM₁₀, PM₀₁, and PM₁₁, reflecting the proportion of pleiotropic single nucleotide polymorphisms (SNPs) among all SNPs associated with at least one trait, representing the strength of shared genetic effects. *AMD* Age-related Macular Degeneration, *DR* Diabetic Retinopathy, *DED* Dry Eye Disease, *PACG* Primary Angle-Closure Glaucoma, *GORD* Gastroesophageal Reflux Disease, *IBD* Inflammatory Bowel Disease, *IBS* Irritable Bowel Syndrome, *PUD* Peptic Ulcer Disease, *DD* Diverticular Disease

Widespread genetic correlations and overlaps were identified across the 40 ocular-gastrointestinal trait pairs. LDSC identified 19 pairs with significant genetic correlations (Fig. 2A), consistent with HDL results (Fig. 2B; Figure S1). The remaining 21 trait pairs were found to be significant based on GPA (Fig. 2C). Cross-trait LDSC intercepts ranged from − 0.011 to 0.012, indicating minimal sample overlap and confirming the reliability of the genetic correlation estimates (Table S2). These findings reveal widespread genetic sharing among 40 gut–eye trait pairs, highlighting pervasive pleiotropy.

### Pleiotropic loci between gastrointestinal and ocular diseases

**Fig. 3:**
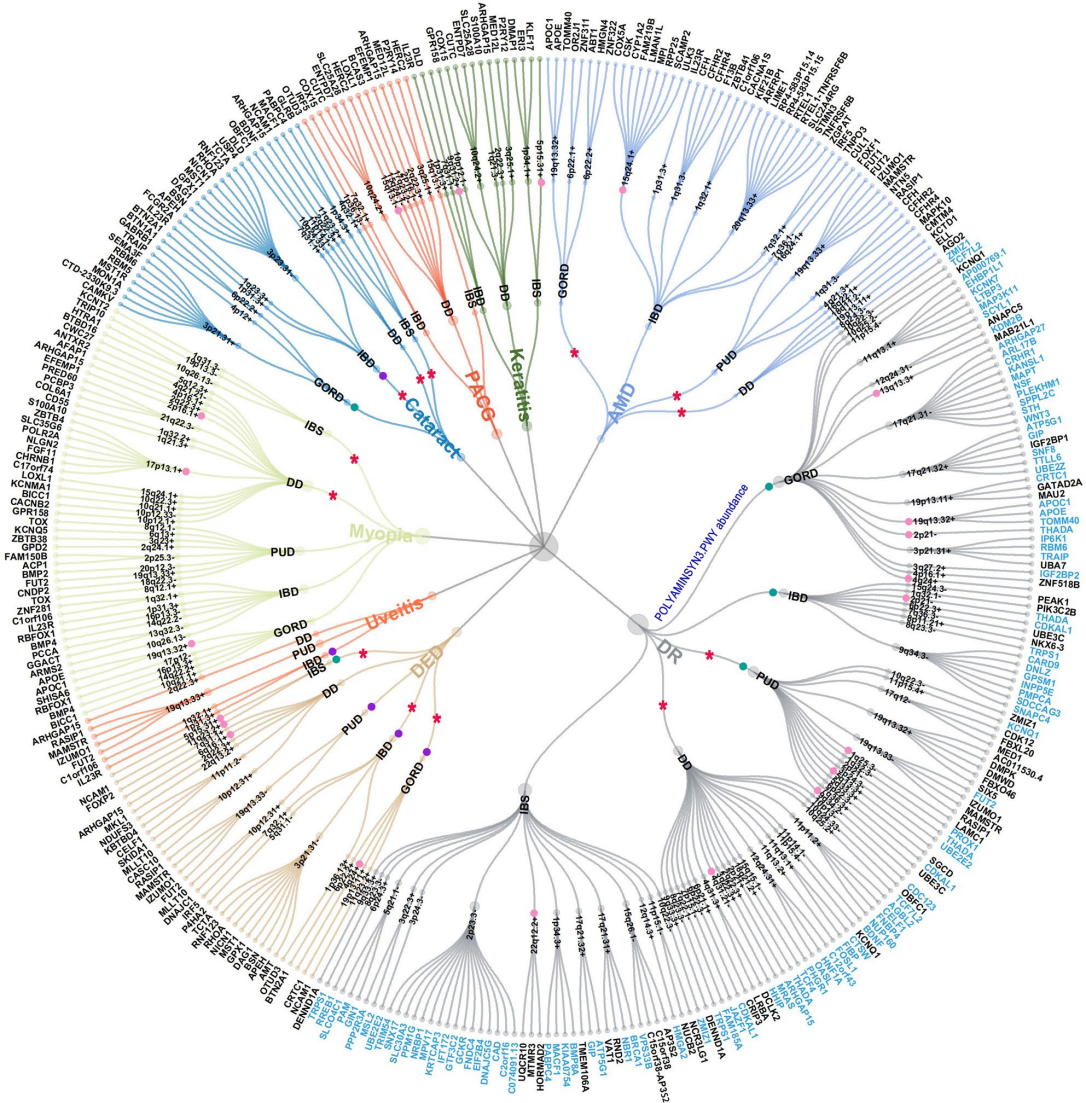
Panorama of pleiotropic associations between gastrointestinal and ocular diseases. This figure summarizes key pleiotropic genetic connections between 8 ocular and 5 gastrointestinal diseases. A total of 366 significant pleiotropic loci were identified across 183 chromosomal regions, with some loci showing concordant or discordant allelic effects. Twenty-one loci exhibited strong evidence of shared causality (PP.H4 > 0.7), and several trait pairs involved significant gene–environment interactions or causal links. The innermost ring represents the 8 ocular diseases, and the second ring shows 5 gastrointestinal diseases. The third ring displays the chromosomal regions with pleiotropic loci, marked with “+” for concordant and “−” for discordant allelic effects. The fourth ring shows MAGMA-significant genes with FUMA functional annotation, where blue gene labels indicate genes shared by gastrointestinal diseases, diabetes, and diabetic retinopathy, reflecting the dependency of DR on diabetes, whereas black labels indicate genes shared only between gastrointestinal diseases and diabetic retinopathy. Gene labels are displayed only when pleiotropic loci could be mapped to at least one statistically significant gene based on MAGMA analysis. Additional symbols indicate shared causal loci (pink dots), G×E interactions (red stars), and causal effects inferred from bidirectional Mendelian randomization, with green dots indicating causal effects from gastrointestinal diseases to ocular diseases, and purple dots indicating causal effects from ocular diseases to gastrointestinal diseases. The size of nodes is used solely for visualization purposes and does not encode quantitative information such as the number of loci or effect size. The blue text on the radial lines extending outward from the center indicates mediation effects through gut microbiota. *AMD* Age-related Macular Degeneration, *DR* Diabetic Retinopathy, *DED* Dry Eye Disease, *PACG* Primary Angle-Closure Glaucoma, *GORD* Gastroesophageal Reflux Disease, *IBD* Inflammatory Bowel Disease, *IBS* Irritable Bowel Syndrome, *PUD* Peptic Ulcer Disease, *DD* Diverticular Disease

PLACO analysis of the 40 trait pairs identified 29,990 potential pleiotropic SNPs (Figure S2; Table S3), and FUMA further identified 366 significant pleiotropic loci, spanning 183 distinct chromosomal regions (Fig. 3; Table S4). Several pleiotropic chromosomal regions recurred across multiple trait pairs, highlighting extensive genetic pleiotropy (Table S5): 1q32.1, 2p16.1, and 2q22.3 were identified in eight trait pairs; 1p31.3 appeared in six trait pairs; 10q26.13, 19q13.33, 4q24, and 6p22.3 were detected in five trait pairs; the remaining 175 unique regions were detected in fewer than five trait pairs. These recurring pleiotropic loci across multiple trait pairs highlight recurrent genomic regions that may contribute to shared susceptibility in gut–eye comorbidity.

Among the 366 pleiotropic loci, 231 of 366 (63.11%) top SNPs exhibited consistent directions of association across gastrointestinal and ocular diseases, suggesting that these variants may simultaneously increase or decrease the risk of both disease groups (Fig. 3; Table S6). Functional annotation based on ANNOVAR (Figure S3) further revealed that 31/366 variants (24 unique) were mapped to exonic regions, including 21 within mRNA exons and 10 within non-coding RNA exons. These analyses highlight recurrent pleiotropic loci that may be relevant to shared susceptibility across multiple gut–eye trait pairs.

### Colocalized loci in gut–eye disease pairs

Further colocalization analysis identified 21 loci (17 unique: 2p21, 4q24, 13q13.3, 19q13.32, 1q25.3, 5q13.3, 9q34.2, 22q12.2, 4q31.21, 15q24.1, 5p15.31, 11q23.2, 6q16.1, 9q31.2, 2p16.1, 17p13.1, 15q13.1) with evidence of colocalized shared signals (PP.H4 > 0.7), of which 15 top SNPs were prioritized as candidate variants underlying the shared signals (Table 1, Table S7, Figure S4). Four chromosomal regions showed colocalization across two trait pairs each, highlighting their potential relevance to the gut–eye axis: 19q13.32 (rs429358: *APOE*) in DR–GORD and Myopia–GORD; 2p21 (rs1322 and rs78487399: *THADA*) in DR–GORD and DR–IBD; 4q24 (rs13107325: *SLC39A8*) in DR–GORD and DED–GORD; 5p15.31 (rs150079703 and rs4562016: *RP11-122F24.1* and RP11-404K5.3) in DED–IBS and Keratitis-IBS. These colocalized variants nominate genes, including *APOE*, *THADA*, and *SLC39A8*, as candidate contributors to gut–eye comorbidity.

**Table 1:** 21 Colocalized Loci identified by colocalization analysis performed on 366 Pleiotropic Loci

Trait pair	Top SNP	Locus boundary	Region	Nearest gene	PP.H3	PP.H4	Best causal	SNP.PP.H4
DR-GORD	rs1322	2:43449385–43818957	2p21	*THADA*	0.0225	0.9116	rs1322	0.1855
DR-GORD	rs13107325	4:103001649–103198082	4q24	*SLC39A8*	0.1340	0.7646	rs13135092	0.5005
DR-GORD	rs9544488	13:36047374–36095039	13q13.3	*NBEA*	0.0329	0.7935	rs9544448	0.2561
DR-GORD	rs429358	19:45392254–45428234	19q13.32	*APOE*	0.0219	0.9349	rs429358	0.9990
DR-IBD	rs78487399	2:43449385–43907630	2p21	*THADA*	0.0087	0.9870	rs78487399	0.6355
DR-PUD	rs12146099	1:182946746–183115341	1q25.3	*RNU6-41P*	0.0793	0.8035	rs12146099	0.2002
DR-PUD	rs7732628	5:76430636–76439250	5q13.3	*ZBED3-AS1*	0.0688	0.8477	rs7732628	0.6441
DR-PUD	rs8176719	9:136132908–136155000	9q34.2	*ABO*	0.0017	0.9955	rs115478735	0.9999
DR-IBS	rs41172	22:30130115–30584388	22q12.2	*MTMR3:CTA-85E5.10*	0.0414	0.7377	rs41172	0.4075
DR-DD	rs11727676	4:145621328–146087979	4q31.21	*HHIP*	0.0026	0.9887	rs11727676	0.9879
AMD-IBD	rs12903896	15:75031521–75281132	15q24.1	*CYP1A2*	0.0267	0.9444	rs12903896	0.6031
DED-GORD	rs13107325	4:103001649–103198082	4q24	*SLC39A8*	0.0083	0.9850	rs13107325	0.8241
DED-IBS	rs150079703	5:7188657–7302389	5p15.31	*RP11-122F24.1*	0.0863	0.7854	rs150079703	0.1615
DED-IBS	rs7128314	11:112826709–112938783	11q23.2	*NCAM1*	0.0991	0.8235	rs7105462	0.0545
DED-DD	rs4839715	6:98214814–98546547	6q16.1	*RP11-436D23.1*	0.2418	0.7561	rs4839715	0.4870
Keratitis-IBD	rs10739246	9:110241529–110274529	9q31.2	*KLF4*	0.0477	0.7118	rs13302327	0.1958
Keratitis-IBS	rs4562016	5:7188657–7270814	5p15.31	*RP11-404K5.3*	0.0871	0.8400	rs150079703	0.0931
Myopia-GORD	rs429358	19:45392254–45424351	19q13.32	*APOE*	0.0333	0.8977	rs429358	0.9893
Myopia-DD	rs11899380	2:55724295–56201773	2p16.1	*EFEMP1*	0.0029	0.9971	rs11899380	0.9285
Myopia-DD	rs12942267	17:7318061–7437665	17p13.1	*ZBTB4*	0.0078	0.9922	rs9217	0.6142
PACG-IBS	rs6497279	15:28344238–28573738	15q13.1	*HERC2*	0.0294	0.7722	rs6497279	0.4199

*DR* Diabetic retinopathy, *AMD* Age-related macular degeneration, *DED* Dry Eye disease, *PACG* Primary angle-closure glaucoma, *GORD* Gastro-oesophageal reflux disease, *IBD* Inflammatory bowel disease, *PUD* Peptic ulcer disease, *IBS* Irritable bowel syndrome, *DD* Diverticular disease. Top SNP, the variant within each locus showing the strongest evidence for shared association; PP.H3, the posterior probability that both traits are associated within a locus but are driven by distinct causal variants; Best causal, the SNP with the highest posterior probability of being the shared causal variant; SNP.PP.H4, the SNP-level posterior probability supporting a shared causal variant (hypothesis H4)

### Genes associated with pleiotropic variants underlying gut–eye diseases

MAGMA analysis (Figures S5–S6) further identified 603 genes associated with pleiotropic variants, among which 261 were repeatedly detected across two or more trait pairs, indicating strong evidence of pleiotropy. Of these 603 significant genes, 354 were functionally annotated as mapped genes in the FUMA (Table S8), further supporting their biological relevance. Specifically, the most frequently identified gene was *HLA-B* (OMIM 142830), detected across 18 trait pairs, followed by *RBFOX1* (OMIM 605104) in 16 trait pairs and *ATF6B* (OMIM 600984) in 13 trait pairs. In addition, *CFB* (OMIM 138470), *CLIC1* (OMIM 602872), *LSM2* (OMIM 607282), *PRRC2A* (OMIM 142580), and *TNXB* (OMIM 600985) were each found to be associated with pleiotropic variants across 12 trait pairs. Overall, a core set of genes associated with pleiotropic variants, including *HLA-B*, *RBFOX1* and *ATF6B*, repeatedly appeared across multiple trait pairs, representing candidate targets for future functional validation.

### Functional and tissue enrichment of genes associated with pleiotropic variants

**Fig. 4:**
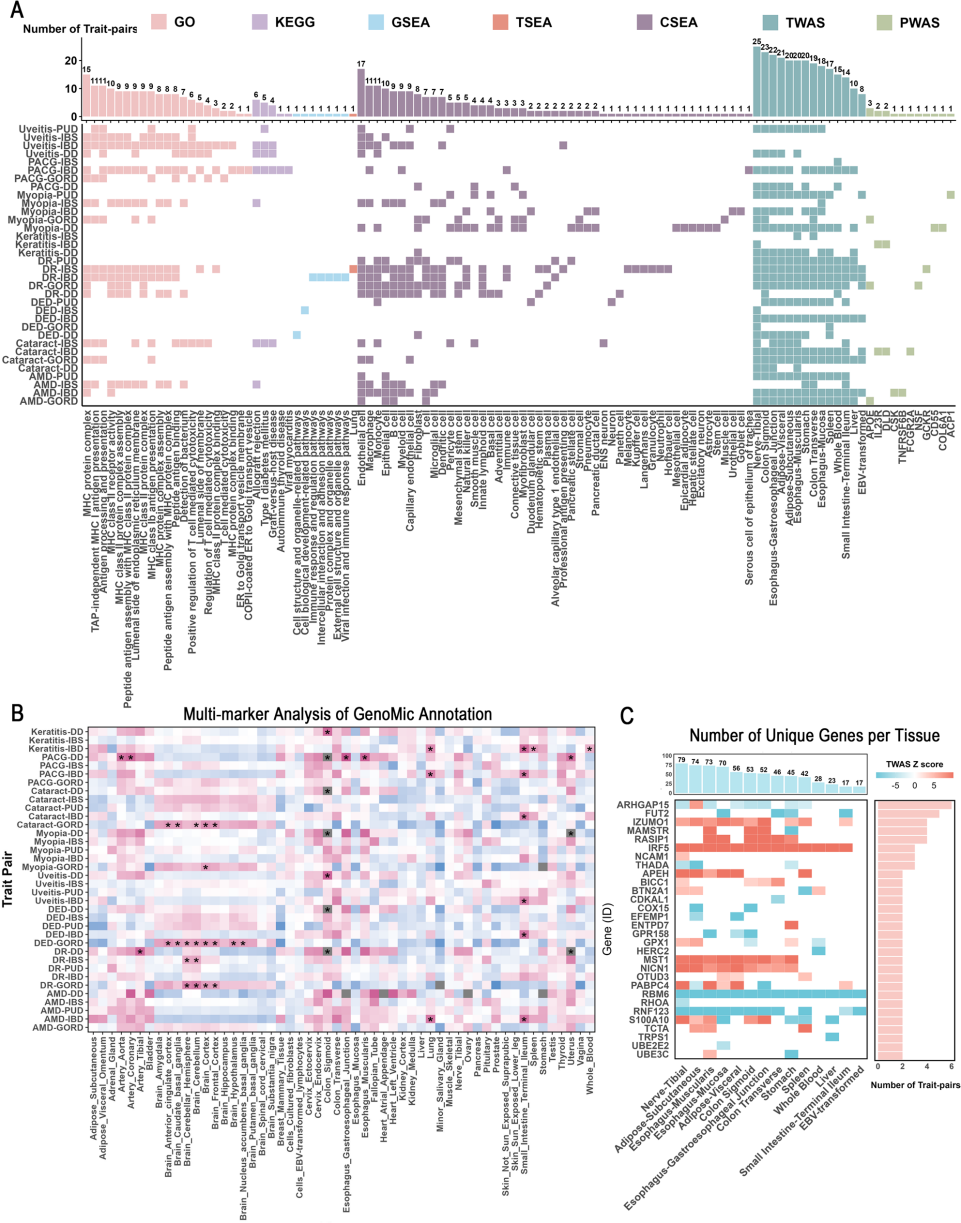
Prioritization of candidate genes and characterization of phenotype and tissue specificity. **A** Heatmap displaying enrichment results across multiple functional annotation categories. The x-axis represents annotation terms, and the y-axis corresponds to trait pairs. Significant annotations are color-filled. Bar plots indicate the number of trait pairs significantly enriched per annotation and the number of significant annotations per trait pair. GO and KEGG analyses revealed that genes mapped to pleiotropic variants are primarily enriched in core immune pathways including immune response, antigen processing and presentation, and cell-mediated cytotoxicity. GSEA showed involvement of risk genes in immune regulation, cell development and adhesion, antigen processing/presentation, and various viral infection-related processes. TSEA indicated notable enrichment of genes associated with pleiotropic variants in lung tissue. CSEA revealed that these risk genes exhibit cell-type-specific expression in multiple immune cell types, mainly B cells, T cells, monocytes, macrophages, and dendritic cells. **B** Tissue enrichment analysis via MAGMA demonstrated significant enrichment of genes in multiple gastrointestinal and immune-related tissues, such as terminal ileum, colon, esophageal gastroesophageal junction, spleen, whole blood, and lung. Color intensity represents the direction and magnitude of β values, with red indicating positive associations and blue indicating negative associations. Asterisks denote statistically significant associations after Bonferroni correction. **C** Heatmap showing tissue-specific expression of candidate genes (y-axis) across 14 normal tissues relevant to gastrointestinal and ocular systems (x-axis). Included genes exhibit significant expression in at least two trait pairs and one relevant tissue simultaneously

GO and KEGG enrichment analyses (Fig. 4A; Table S9) revealed that genes associated with pleiotropic variants were significantly enriched in multiple pathways related to the major histocompatibility complex, involving key immune functions such as immune response, antigen processing and presentation, and cell-mediated cytotoxicity. GSEA analysis (Fig. 4A; Table S10) further revealed that these risk genes are primarily involved in immune-related pathways, including immune regulation, cell development and adhesion, antigen processing and presentation, as well as pathways related to various viral infections. Subsequently, tissue expression enrichment analysis using MAGMA (Fig. 4B; Figures S7–S8) showed that these genes associated with pleiotropic variants were significantly enriched in various gastrointestinal- and immune-related tissues, including the small intestine terminal ileum, colon sigmoid, esophagus gastroesophageal junction, esophagus muscularis, spleen, whole blood, uterus, brain, lung, and arteries. In addition, TSEA also identified significant enrichment of these genes associated with pleiotropic variants in lung tissue (Fig. 4A; Table S11). Notably, CSEA analysis revealed that these risk genes were enriched in multiple immune cell types, including B cells, macrophages, dendritic cells, T cells, and monocytes, as well as in certain endothelial and mesenchymal cells (Fig. 4A; Table S12; Figure S9). Therefore, our findings suggest that genes associated with pleiotropic variants are primarily related to immune and gastrointestinal pathways, highlighting immune dysregulation and gut-associated processes as potential contributors to the gut–eye axis.

### Transcriptome- and proteome-wide associations with gut–eye disease risk

We further examined the transcriptional levels of these 603 genes (identified by MAGMA analysis) using TWAS (Table S13). A total of 154 genes showed significant transcriptional associations with at least one gut-eye trait pair. Notably, decreased expression of *RBM6* was consistently associated with increased gut–eye disease risk across all tissues examined (Fig. 4C), whereas elevated expression of *IRF5* was significantly linked to increased disease risk in all tissues except EBV-transformed lymphocytes. In addition, *ARHGAP15* also showed significant transcriptomic associations across multiple tissues.

We then assessed the protein expression levels of these 603 genes using PWAS (Table S14), identifying 11 significant genes. Notably, plasma levels of *APOE* were significantly correlated with the risk of GORD and three ocular diseases (myopia, DR and AMD, *P*_FDR_ < 0.05; Table S14). Additionally, *IL23R* and *DLD* showed significant associations between IBD and two ocular conditions (keratitis and cataract, *P*_FDR_ < 0.05). These transcriptome- and proteome-wide analyses nominate specific genes and proteins, including *RBM6*, *IRF5*, *ARHGAP15*, *APOE*, *IL23R*, and *DLD*, as candidate molecular correlates linking genetic variation to gut–eye disease risk.

### Gene–environment interactions of pleiotropic variants in gut–eye comorbidity

**Fig. 5:**
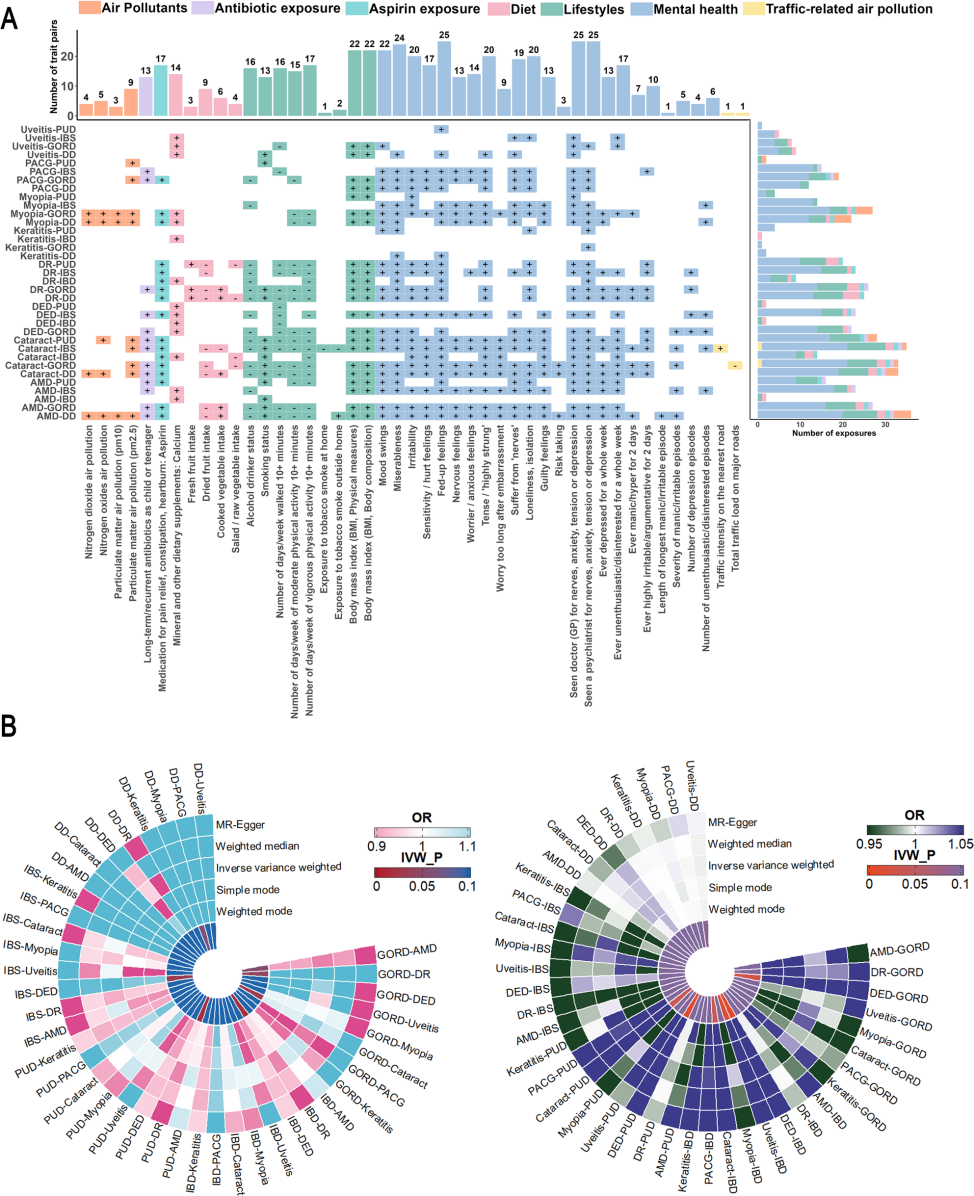
Pleiotropic variant–exposure interactions on comorbidity risk and bi-directional Mendelian randomization analysis between gastrointestinal and ocular diseases. **A** Associations between 64 modifiable exposures and 40 gastrointestinal–ocular comorbid trait pairs. Significant associations are color-coded, with “+” and “−” indicating FDR-adjusted significant positive and negative effects, respectively. The results reveal that most exposures are significantly linked to comorbidity risk, particularly highlighting consistent risk directions for adverse mental health conditions and lifestyle factors across multiple comorbid traits. **B** Circular plots illustrating causal effects of gastrointestinal diseases on ocular diseases (left) and ocular diseases on gastrointestinal diseases (right). The innermost ring represents *P*-values derived from the Inverse Variance Weighted method, while rings from inside to outside display OR estimates from MR-Egger, Weighted Median, Inverse Variance Weighted, Simple Mode, and Weighted Mode methods, respectively

We further explored statistical gene–environment interactions between candidate pleiotropic variants and modifiable environmental exposures in relation to gastrointestinal–ocular comorbidity risk (Tables S15–S16). Overall, an average of 45 environmental exposures were significantly associated with 35 gut–eye disease pairs (*P*_FDR_ < 0.05). When exposures were considered by category, distinct disease-specific patterns of gene–environment interaction became apparent (Fig. 5A; Table S17).

26 pleiotropic genetic variants interacted with 16 modifiable exposures across 13 gut-eye disease pairs (Table 2; Table S18). Antibiotic exposure, particularly early-life and recurrent use, showed consistent positive associations with multiple gut–eye disease pairs and exhibited significant interactions with pleiotropic variants including rs62396201, rs12462498, and rs2353487, predominantly observed in disease pairs involving inflammatory gastrointestinal and ocular conditions. Diet-related exposures interacted with variants such as rs17720293 and rs703965, modulating genetic susceptibility across several gut–eye disease pairs. Lifestyle and adiposity-related factors also demonstrated clear patterns: higher BMI was positively associated with, whereas physical activity was inversely associated with, the risk of multiple disease pairs, with significant G×E interaction terms observed for BMI (rs7631010, rs150079703) and physical activity (rs12236873, rs13107325, rs7128314), based on observational analyses. In addition, smoking and adverse mental health traits—including fed-up feelings, tension, irritability, and loneliness—showed significant interactions with pleiotropic variants (rs3820330, rs1546707, rs4319542, rs61320678, rs2279623, rs2784272) in disease pairs characterized by chronic inflammatory burden. Collectively, these findings indicate that specific categories of modifiable exposures show statistically significant gene–environment interactions with pleiotropic variants across gastrointestinal–ocular disease pairs, highlighting the importance of considering environmental context when interpreting genetic associations.

**Table 2:** Significant results of gene–environment interactions in UK Biobank

Trait	SNP	Exposure	Interaction		
			OR	*P*	FDR_P
AMD-GORD	rs62396201	Long-term/recurrent antibiotics	1.806 (1.214–2.689)	3.57E–03	1.78E–02
AMD-GORD	rs17720293	Dried fruit intake	0.932 (0.891–0.975)	2.40E–03	1.20E–02
AMD-GORD	rs62396201	Fed-up feelings	0.835 (0.731–0.954)	7.99E–03	3.99E–02
AMD-GORD	rs17720293	Worry too long after embarrassment	0.734 (0.599–0.899)	2.80E–03	1.40E–02
AMD-PUD	rs7707527	Fed-up feelings	0.688 (0.525–0.902)	6.83E–03	2.39E–02
AMD-PUD	rs370509910	Fed-up feelings	0.446 (0.252–0.790)	5.61E–03	2.39E–02
AMD-DD	rs150394100	Walked	1.183 (1.068–1.310)	1.27E–03	2.79E–02
AMD-DD	rs1546707	Fed-up feelings	0.747 (0.624–0.894)	1.45E–03	3.18E–02
Cataract-IBD	rs3820330	Smoking status	1.290 (1.080–1.541)	5.02E–03	3.01E–02
Cataract-IBS	rs1940725	Tense	1.305 (1.097–1.552)	2.60E–03	2.08E–02
Cataract-IBS	rs4319542	Loneliness, isolation	0.735 (0.618–0.875)	5.26E–04	4.21E–03
Cataract-DD	rs2353487	Long-term/recurrent antibiotics	0.709 (0.563–0.892)	3.41E–03	2.72E–02
Cataract-DD	rs4333882	Seen doctor for nerves, anxiety, or depression	0.880 (0.807–0.961)	4.28E–03	3.43E–02
DR-PUD	rs703965	Salad / raw vegetable intake	0.863 (0.792–0.941)	8.34E–04	2.33E–02
DR-PUD	rs61320678	Miserableness	2.222 (1.431–3.450)	3.75E–04	1.05E–02
DR-DD	rs12236873	Moderate physical activity	1.123 (1.066–1.182)	1.08E–05	4.12E–04
DR-DD	rs889970	Fed-up feelings	1.484 (1.215–1.812)	1.11E–04	4.23E–03
DED-GORD	rs12462498	Long-term/recurrent antibiotics	0.296 (0.123–0.714)	6.74E–03	3.37E–02
DED-GORD	rs13107325	Vigorous physical activity	1.408 (1.080–1.836)	1.16E–02	2.89E–02
DED-GORD	rs12462498	Vigorous physical activity	0.814 (0.726–0.913)	4.27E–04	2.13E–03
DED-IBD	rs3197999	Calcium	0.218 (0.076–0.630)	4.90E–03	4.90E–02
DED-IBS	rs28469251	Walked	1.187 (1.054–1.338)	4.84E–03	2.42E–02
DED-IBS	rs7128314	Vigorous physical activity	1.229 (1.051–1.437)	9.91E–03	4.96E–02
DED-IBS	rs7631010	Body mass index	0.943 (0.898–0.990)	1.80E–02	4.50E–02
DED-IBS	rs150079703	Body mass index	0.944 (0.902–0.987)	1.20E–02	4.50E–02
Myopia-IBS	rs2279623	Irritability	0.384 (0.209–0.705)	2.02E–03	1.01E–02
Myopia-DD	rs1888693	Vigorous physical activity	1.108 (1.035–1.187)	3.19E–03	4.15E–02
Myopia-DD	rs12764415	Vigorous physical activity	1.190 (1.071–1.324)	1.29E–03	3.35E–02
Myopia-DD	rs2784272	Tense	1.588 (1.194–2.112)	1.47E–03	3.82E–02

*DR* Diabetic retinopathy, *AMD* Age-related macular degeneration, *DED* Dry Eye disease, *PACG* Primary angle-closure glaucoma, *GORD* Gastro-oesophageal reflux disease, *IBD* Inflammatory bowel disease, *PUD* Peptic ulcer disease, *IBS* Irritable bowel syndrome, *DD* Diverticular disease

### Bidirectional Mendelian randomization

We applied bidirectional MR analyses to further assess genetically predicted associations across 40 gastrointestinal–ocular trait pairs (Figs. 5B). In the direction of gastrointestinal diseases affecting ocular diseases, five significant genetically predicted positive associations were identified, including GORD–cataract, GORD–DR, IBD–DR, PUD–DR, and IBS–DED (Table S19). Conversely, six significant genetically predicted positive associations were detected, including DED–GORD, cataract–IBD, DED–IBD, uveitis–IBD, cataract–PUD, and DED–PUD (Table S20). The MR-Egger intercepts provided no evidence of directional pleiotropy, although this test may have limited power given the number of instrumental variables, and Cochran’s Q tests detected no significant heterogeneity across trait pairs (Table S21). Notably, no evidence of bidirectional genetically predicted associations was observed for any trait pair.

### Mediation analysis

Subsequently, we integrated genetic and gut microbiome data for Mendelian randomization and mediation analyses to elucidate the microbiome’s contributory role in the gut–eye disease pairs. The gut microbial taxa polyamine biosynthesis pathway (POLYAMINSYN3.PWY) exhibited a significant genetically predicted positive association with DR (*P* < 0.01), suggesting a potential association with increased DR risk. Additionally, POLYAMINSYN3.PWY showed a genetically predicted negative association with GORD (*P* < 0.01), while GORD showed a genetically predicted positive association with DR (*P* = 0.04). Mediation analysis further suggested that POLYAMINSYN3.PWY may account for approximately 6.3% of the statistically inferred association between GORD and DR (Table S22). These results raise the possibility of a microbiome-related pathway involved in the statistically inferred association between gastrointestinal and ocular diseases, which warrants further investigation.

## Discussion

This study represents a large-scale investigation of the shared genetic architecture between gastrointestinal and ocular diseases based primarily on summary-level statistical analyses. We identified 366 shared loci and 603 genes associated with pleiotropic variants significantly enriched in immune and inflammatory pathways, suggesting shared biological pathways that may contribute to cross-organ comorbidities. Importantly, we uncovered 26 gene–environment interactions involving 16 modifiable exposures, emphasizing that environmental factors statistically interact with genetic variants. Furthermore, mediation analysis suggested a potential statistical association involving the polyamine biosynthesis pathway in the relationship between GORD and DR. However, these findings should be interpreted as hypothesis-generating, given the sequential analytical framework and the inherent uncertainty of summary-level statistical inference.

Currently, research on the genetic correlations between gastrointestinal and ocular diseases remains limited. Although some studies have suggested clinical comorbidity between some gastrointestinal (e.g., IBD, IBS, and PUD) and ocular diseases (e.g., DED, DR, and keratitis), systematic investigations into their shared genetic architecture have been limited [6, 54–59]. This study provides widespread evidence of shared genetic architecture between gastrointestinal and ocular diseases, including substantial genetic correlation and pleiotropy that may reflect common etiological pathways. However, all analyses in this study were restricted to individuals of European ancestry, which may limit the generalizability of our findings to other populations.

Current research on shared loci between gastrointestinal and ocular diseases remains limited. Taleban S et al. [60] identified potential shared immune-related risk loci, including *TSPAN14* and *TNFSF14*, between IBD and uveitis. However, their study focused on specific disease pairs and did not aim to systematically characterize the broader shared genetic architecture. This study identified 366 loci across five gastrointestinal and eight ocular diseases, including 21 loci with statistically inferred colocalized shared signals. Several pleiotropic regions recurred across multiple trait pairs, suggesting their potential roles in disease co-occurrence. In our study, *TSPAN14* and *TNFSF14* were not detected as pleiotropic loci for IBD and uveitis. This may be due to differences in study populations, phenotype definitions, analytical strategies, and/or statistical power. These discrepancies highlight the challenges of replication in complex genetic studies and reinforce the need for cautious interpretation of individual findings, including those from the present study. In the absence of an independent replication cohort, the novel findings reported here should be considered preliminary and hypothesis-generating. Further replication in independent cohorts, along with larger sample sizes and more refined phenotyping, will be essential to clarify these discrepancies.

Our pathway analyses suggested that genes associated with gastrointestinal–ocular comorbidity risk are predominantly enriched in immune regulation and inflammation-related pathways. These findings are consistent with the well-established immune-mediated inflammatory pathology observed in various gastrointestinal and ocular diseases, supporting the putative involvement of immune-inflammatory responses in the GEA [61]. Dysbiosis of the gut microbiota may profoundly affect host immunity, manifesting as altered T cell and B cell responses, cytokine secretion, and host metabolic processes. These immune and metabolic changes, in turn, contribute to the development of specific ocular diseases [62, 63]. Furthermore, TSEA and CSEA suggested enrichment of genes associated with pleiotropic variants in gastrointestinal and immune-related tissues and cell types. Notably, the repeated enrichment observed in lung tissue across MAGMA and TSEA was not an expected finding for a study focused on the gut-eye axis and should therefore be interpreted cautiously. This signal may reflect shared immune or mucosal inflammatory programs across barrier tissues, but it may also partly arise from properties of the reference expression datasets or the statistical enrichment framework rather than lung-specific pathogenic relevance. Accordingly, this finding should not be overinterpreted as evidence of a lung-specific mechanism in gut–eye comorbidity, and further validation is required to clarify its biological relevance.

At the gene level, transcriptome-wide and proteome-wide analyses further highlighted *IRF5* and *ARHGAP15* as notable candidate genes associated with both gastrointestinal and ocular diseases. Increased expression of *IRF5* was consistently associated with elevated disease risk across nearly all tissues examined, in line with the strong enrichment of immune and inflammatory pathways observed in our functional analyses. *IRF5* encodes a key transcription factor involved in innate immune activation, cytokine production, and interferon signaling, and has been implicated in multiple immune-mediated disorders [64]. *ARHGAP15* is a regulator of immune cell signaling and inflammatory responses [65]. Previous studies have also reported its involvement in several gastrointestinal disorders [66, 67]. Its recurrence across multiple trait pairs in our analysis may reflect its broad immunological functions, although additional studies are needed to clarify its specific role. In addition, several other pleiotropic loci mapped to notable genes, including *APOE*, *NCAM1*, *HHIP*, *SLC39A8* and *ABO*, which have been associated with immune regulation, metabolic processes, or disease susceptibility [68–70]. The remaining loci were mapped to genes involved in diverse biological processes or with less well-characterized functions, highlighting the need for further functional studies. Collectively, these findings suggest that immune dysregulation and related pathways may contribute to shared genetic susceptibility to gastrointestinal and ocular diseases, while also providing candidate genes for future investigation.

Environmental factors are established risk factors for various gastrointestinal and ocular diseases [71, 72]. Our findings further suggest that lifestyle factors, mental health-related traits, and air pollution exposures are statistically associated with gut–eye comorbidity risk. In addition, the observed gene–environment interactions indicate that environmental exposures may be associated with variation in genetic effects. However, these G×E findings should be interpreted cautiously, as the analyses were observational and therefore susceptible to residual confounding. In particular, interactions involving questionnaire-derived and self-reported exposures, such as fed-up feelings and related mental health traits, are difficult to interpret biologically and may reflect complex behavioral, psychosocial, or health-related correlates rather than direct mechanistic pathways. Accordingly, these findings are best regarded as exploratory statistical associations that require further validation.

The gut microbiota has been implicated in neurological, metabolic, and inflammatory diseases, with dysbiosis contributing to ocular disease risk via microbial metabolites and systemic immune responses [73]. Polyamines, such as spermidine and spermine, are key metabolites in the polyamine biosynthesis pathway and regulate inflammation, oxidative stress, and cell death [74]. They may protect retinal pigment epithelium cells from high-glucose–induced damage, highlighting spermidine as a potential therapeutic target for DR [75]. Moreover, polyamine oxidation drives neurodegeneration, and neural injury is linked to the development of DR [76, 77]. Our analyses suggested that the polyamine biosynthesis pathway may be statistically implicated in the association between GORD and DR. These findings raise the possibility that gut microbiota-related pathways may be relevant to disease risk along the GEA, although this interpretation remains preliminary and requires further experimental validation.

This study has several limitations. First, all analyses were restricted to individuals of European ancestry. Differences in linkage disequilibrium structure and allele frequencies across populations may influence genetic effect estimates, potentially leading to population-specific association signals and limiting the generalizability of our findings to non-European populations. Second, the Mendelian randomization results should be interpreted with caution. Although we used genome-wide significant, independent SNPs as instruments and performed multiple sensitivity analyses, the core assumptions of MR, including relevance, independence, and exclusion restriction, cannot be fully empirically verified in summary-level analyses. Accordingly, these findings are best regarded as genetically predicted and hypothesis-generating associations rather than definitive evidence of causality. Third, although gut microbiome analyses suggested a potential mediating role, the effects observed were modest, indicating other factors may also contribute. Fourth, although all analyses were conducted using summary-level approaches with harmonized quality control procedures, residual heterogeneity in phenotype definition, study design, and cohort composition across the contributing datasets cannot be entirely excluded. Fifth, gene–environment interaction analyses were observational and may be affected by residual confounding, measurement error, reporting bias, misclassification, and unmeasured environmental factors, particularly for questionnaire-derived exposures. Sixth, this study did not include an independent replication cohort; therefore, the novel findings should be regarded as preliminary and hypothesis-generating pending validation in independent datasets and functional studies. More broadly, as this study was based on a sequential series of computational and statistical inferences, uncertainty at each stage may have propagated across downstream analyses.

## Conclusion

In summary, this study systematically characterized shared genetic architecture between gastrointestinal and ocular diseases. We identified 366 pleiotropic loci and 603 candidate genes, suggesting statistically inferred links involving immune and inflammatory responses, gene–environment interactions, and potential microbiome-related pathways. These findings support the possibility that genetic pleiotropy, host–microbiome interactions, and environmental exposures may contribute to the gut–eye axis. Overall, our results should be regarded as preliminary and hypothesis-generating, providing a framework for future replication, functional studies, and experimental validation.

## Supplementary Information

Below is the link to the electronic supplementary material.

Supplementary Material 1.

## Data Availability

All analyses were based on publicly available data. The sources of the GWAS summary statistics included in this study are cited in the methods section. The individual-level phenotypic and genetic data used in this study are available from the UK Biobank resource, with access application number 91320. In accordance with data privacy laws, UK Biobank data is subject to restricted access and can be obtained by submitting an access request through the UK Biobank Access Management System (https://ams.ukbiobank.ac.uk/ams/).
