# Supplementary material for: Cross-trait genomic and sequential analyses of multiple omics datasets identified shared genetic components for the gut–eye axis
Source: Hum Genomics. 2026 Mar 17;20:78. doi: 10.1186/s40246-026-00947-6 (PMC13107603; doi:10.1186/s40246-026-00947-6)
Supplement: Supplementary file 1 — Supplementary Material 1. [file 40246_2026_947_MOESM1_ESM.docx]

**Supplementary information**

**Table S1.** Details of GWAS Summary Data Sources....................................................3

**Table S2.** Genetic Correlation and Genetic Overlap Estimations Between 40 Pairwise Traits...............................................................................................................................4

**Table S3.** Summary of genome-wide significant pleiotropic SNPs and FUMA-Annotated pleiotropic genomic risk loci for each pair of traits.........................7

**Table S4.** Pleiotropic Genomic Loci Identified by FUMA Using PLACO Results....10

**Table S5.** Three Chromosomal Regions Identified Across Eight Trait Pairs..............19

**Table S6.** Effect Sizes and *P* Values of Top SNPs in Pleiotropic Loci From Original GWAS Summary Statistics...........................................................................................20

**Table S7.** Colocalized Loci Identified by Colocalization Analysis Performed on 366 Pleiotropic Loci ...........................................................................................................29

**Table S8.** Candidate Pleiotropic Genes Identified by MAGMA based on the mapped genes in FUMA............................................................................................................38

**Table S9.** Significant enriched GO and KEGG pathways across the 40 trait pairs.....47

**Table S10.** Enriched pathways in GSEA analysis across the 40 trait pairs.................58

**Table S11.** Enriched tissues across the 40 gastrointestinal-eye trait pairs in TSEA...........................................................................................................................60

**Table S12.** Enriched tissues across the 40 gastrointestinal-eye trait pairs in CSEA...........................................................................................................................61

**Table S13.** Significant TWAS results based on candidate pleiotropic genes.............................................................................................................................87

**Table S14.** Significant PWAS results based on candidate pleiotropic genes...........................................................................................................................122

**Table S15.** Data field of Modifiable exposure for G×E analysis in UK Biobank......................................................................................................................123**Table S16.** Characteristics for G×E analysis in UK Biobank....................................125

**Table S17.** Significant results between 64 modifiable exposures and 40 trait pairs in UK Biobank................................................................................................................128

**Table S18.** Significant results of G×E analysis in UK Biobank......................................................................................................................150**Table S19.** Mendelian Randomization estimates across the 40 gastrointestinal-eye trait pairs.....................................................................................................................152

**Table S20.** Mendelian Randomization estimates across the 40 eye-gastrointestinal trait pairs.....................................................................................................................153

**Table S21.** MR-Egger intercept and Cochrane Q test for the two-sample

Mendelian randomization...........................................................................................154

**Table S22.** The mediation effect using two-stage MR...............................................155

**Figure S1.** Comparing genetic correlation estimates from LDSC and HDL methods......................................................................................................................156

**Figure S2.** PLACO identified a total of 29,990 single nucleotide variants as potential pleiotropic variants across 40 gastrointestinal–ocular trait pairs...............................157

**Figure S3.** ANNOVAR category annotation.............................................................158

**Figure S4.** Supporting the presence of shared causal variants between the two traits............................................................................................................................159

**Figure S5.** Candidate Pleiotropic Genes Identified by MAGMA (AMD, Cataract, DED, DR)...................................................................................................................160

**Figure S6.** Candidate Pleiotropic Genes Identified by MAGMA (Keratitis, Myopia, PACG, Uveitis)...........................................................................................................161

**Figure S7.** Expression Heatmap (GORD, IBD, PUD)..............................................162

**Figure S8.** Enrichment analysis by MAGMA...........................................................163

**Figure S9.** Summary of enriched cell types by CSEA..............................................164

**Table S1.** Details of GWAS Summary Data Sources

| **Disease** | **Abbreviations** | **Definitions** | **Source** | **Sample size** | **GCST ID** | **Ancestry** |
| --- | --- | --- | --- | --- | --- | --- |
| Age-related macular degeneration | AMD | Matched hospital-bases ICD9 or ICD10 coding | MVP | 426887 | GCST90475849 | EUR |
| Diabetic retinopathy | DR | Matched hospital-bases ICD9 or ICD10 coding | MVP | 432209 | GCST90475689 | EUR |
| Dry Eye disease | DED | Matched hospital-bases ICD9 or ICD10 coding | MVP | 398636 | GCST90475895 | EUR |
| Uveitis | UV | Matched hospital-bases ICD9 or ICD10 coding | MVP | 448251 | GCST90477735 | EUR |
| Myopia | Myopia | Matched hospital-bases ICD9 or ICD10 coding | MVP | 398816 | GCST90475880 | EUR |
| Cataract | CAT | Matched hospital-bases ICD9 or ICD10 coding | MVP | 404086 | GCST90477674 | EUR |
| Primary angle-closure glaucoma | PACG | Matched hospital-bases ICD9 or ICD10 coding | MVP | 446964 | GCST90475876 | EUR |
| Keratitis | KAT | Matched hospital-bases ICD9 or ICD10 coding | MVP | 435345 | GCST90477721 | EUR |
| Diabetes | Diabetes | Matched hospital-bases ICD9 or ICD10 coding | MVP | 315668 | GCST90475265 | EUR |
| Gastro-oesophageal reflux disease | GORD | UKB data field: 131585 | The UK Biobank | 456327 | [GCST011503](https://www.ebi.ac.uk/gwas/studies/GCST011503) | EUR |
| Inflammatory bowel disease | IBD | A combination of Crohn’s diseases (UKB data field: 131627), ulcerative colitis (UKB data field: 131629) diagnoses | The UK Biobank | 456327 | [GCST011501](https://www.ebi.ac.uk/gwas/studies/GCST011501) | EUR |
| Peptic ulcer disease | PUD | A combination of gastric ulcer cases (UKB data field: 131591), duodenal ulcer cases (UKB data field: 131593), other site peptic ulcer cases (UKB data field: 131595) and gastro-jejunal ulcer cases (UKB data field: 131597) | The UK Biobank | 456327 | [GCST011504](https://www.ebi.ac.uk/gwas/studies/GCST011504) | EUR |
| Irritable bowel syndrome | IBS | UKB data field: 131639 | The UK Biobank and Bellygenes initiative | 486601 | GCST90016564 | EUR |
| Diverticular disease | DD | Matched hospital-bases ICD9 or ICD10 coding | The UK Biobank | 451099 | GCST008105 | EUR |

Note: MAP: The Million Veteran Program.

**Table S2.** Genetic Correlation and Genetic Overlap Estimations Between 40 Pairwise Traits^a^

|  | **Genetic correlation (LDSC)** | | | | | **Genetic correlation (HDL)** | | | **Genetic overlap** | | |
| --- | --- | --- | --- | --- | --- | --- | --- | --- | --- | --- | --- |
| **Trait pair** | **Intercept (SE)** | ***P*_Int_** | **Genetic covariance (SE)** | **Genetic correlation (SE)** | ***P*_LDSC_** | **Genetic covariance (SE)** | **Genetic correlation (SE)** | ***P*_HDL_** | **PM 11** | **PAR^b^** | ***P* _GPA_** |
| DR-GORD | -0.0007 (0.0058) | 9.02e-01 | 0.0071 (0.0012) | 0.1714 (0.0288) | 2.70e-09 | 0.0071 (0.0008) | 0.1478 (0.0188) | 3.29e-15 | 0.132 | 0.361 | <1e −300 |
| DR-IBD | 0.0078 (0.0056) | 1.65e-01 | -0.0005 (0.0011) | -0.0196 (0.0433) | 6.51e-01 | 0.0003 (0.0010) | 0.0081 (0.0324) | 8.02e-01 | 0.015 | 0.056 | <1e −300 |
| DR-PUD | -0.0024 (0.0064) | 7.08e-01 | 0.0045 (0.0013) | 0.1777 (0.0516) | 5.69e-04 | 0.0047 (0.0009) | 0.1682 (0.0342) | 9.00e-07 | 0.106 | 0.341 | <1e −300 |
| DR-IBS | -0.0065 (0.0067) | 3.32e-01 | 0.0029 (0.0014) | 0.0758 (0.0362) | 3.64e-02 | 0.0027 (0.0008) | 0.0643 (0.0190) | 7.27e-04 | 0.129 | 0.419 | <1e −300 |
| DR-DD | -0.0041 (0.0068) | 5.50e-01 | 0.0050 (0.0014) | 0.1102 (0.0319) | 5.49e-04 | 0.0047 (0.0011) | 0.0852 (0.0217) | 8.62e-05 | 0.087 | 0.192 | 4.25e-11 |
| DED-GORD | 0.0037 (0.0052) | 4.78e-01 | 0.0152 (0.0011) | 0.5627 (0.0412) | 1.49e-42 | 0.0157 (0.0008) | 0.5136 (0.0331) | 2.22e-54 | 0.092 | 0.190 | 5.18e-03 |
| DED-IBD | 0.0122 (0.0054) | 2.40e-02 | 0.0005 (0.0010) | 0.0328 (0.0619) | 5.96e-01 | 0.0022 (0.0008) | 0.1101 (0.0401) | 6.03e-03 | 0.044 | 0.109 | 6.44e-64 |
| DED-PUD | -0.0039 (0.0047) | 4.08e-01 | 0.0091 (0.0010) | 0.5567 (0.0599) | 1.42e-20 | 0.0084 (0.0007) | 0.4681 (0.0457) | 1.18e-24 | 0.088 | 0.203 | 1.57e-21 |
| DED-IBS | -0.0062 (0.0052) | 2.34e-01 | 0.0130 (0.0011) | 0.5191 (0.0432) | 2.82e-33 | 0.0121 (0.0007) | 0.4553 (0.0293) | 2.48e-54 | 0.184 | 0.422 | <1e −300 |
| DED-DD | -0.0004 (0.0062) | 9.52e-01 | 0.0095 (0.0012) | 0.3232 (0.0421) | 1.73e-14 | 0.0096 (0.0007) | 0.266 (0.0213) | 9.37e-36 | 0.148 | 0.227 | 1.36e-03 |
| Uveitis-GORD | -0.0043 (0.0051) | 4.00e-01 | 0.0023 (0.0008) | 0.3662 (0.1353) | 6.80e-03 | 0.0016 (0.0007) | 0.1075 (0.0463) | 2.01e-02 | 0.001 | 0.004 | <1e −300 |
| Uveitis-IBD | -0.0027 (0.005) | 5.93e-01 | 0.0006 (0.0008) | 0.1511 (0.2028) | 4.56e-01 | 0.0002 (0.0006) | 0.0188 (0.0604) | 7.56e-01 | 0.001 | 0.067 | <1e −300 |
| Uveitis-PUD | -0.0066 (0.0046) | 1.53e-01 | 0.0014 (0.0007) | 0.3750 (0.1917) | 5.05e-02 | 0.0007 (0.0005) | 0.0813 (0.0625) | 1.94e-01 | 0.001 | 0.007 | 4.36e-193 |
| Uveitis-IBS | -0.002 (0.0045) | 6.59e-01 | 0.0014 (0.0008) | 0.2387 (0.1443) | 9.81e-02 | 0.0013 (0.0004) | 0.1001 (0.0333) | 2.62e-03 | 0.002 | 0.011 | <1e −300 |
| Uveitis-DD | -0.0002 (0.0051) | 9.70e-01 | 0.0023 (0.0009) | 0.3357 (0.1381) | 1.50e-02 | 0.0024 (0.0006) | 0.1394 (0.0353) | 7.95e-05 | 0.117 | 0.515 | 1.59e-47 |
| Myopia-GORD | -0.0037 (0.006) | 5.33e-01 | 0.0051 (0.0013) | 0.1297 (0.0324) | 6.16e-05 | 0.0048 (0.0009) | 0.1146 (0.0210) | 4.50e-08 | 0.103 | 0.240 | <1e −300 |
| Myopia-IBD | 0.0057 (0.0062) | 3.59e-01 | 0.0008 (0.0011) | 0.0361 (0.0492) | 4.62e-01 | 0.0015 (0.0007) | 0.0540 (0.0254) | 3.34e-02 | 0.010 | 0.033 | 6.47e-114 |
| Myopia-PUD | -0.0024 (0.0059) | 6.81e-01 | 0.0022 (0.0011) | 0.0945 (0.0474) | 4.62e-02 | 0.0015 (0.0008) | 0.0637 (0.0335) | 5.69e-02 | 0.080 | 0.216 | 2.95e-156 |
| Myopia-IBS | 0.0029 (0.0056) | 6.03e-01 | 0.0035 (0.0011) | 0.0981 (0.0300) | 1.08e-03 | 0.0037 (0.0008) | 0.1015 (0.0224) | 5.69e-06 | 0.102 | 0.262 | <1e −300 |
| Myopia-DD | 0.0011 (0.0063) | 8.66e-01 | 0.0060 (0.0013) | 0.1413 (0.0316) | 7.90e-06 | 0.0061 (0.0009) | 0.1232 (0.0193) | 1.73e-10 | 0.120 | 0.305 | 5.01e-134 |
| Cataract-GORD | -0.0007 (0.0048) | 8.77e-01 | 0.0132 (0.0010) | 0.5042 (0.0372) | 6.30e-42 | 0.0129 (0.0007) | 0.4420 (0.0258) | 1.12e-65 | 0.208 | 0.488 | <1e −300 |
| Cataract-IBD | 0.009 (0.0048) | 6.00e-02 | 0.0013 (0.0009) | 0.0827 (0.0562) | 1.41e-01 | 0.0026 (0.0007) | 0.1401 (0.0404) | 5.19e-04 | 0.011 | 0.026 | 2.63e-29 |
| Cataract-PUD | 0.0001 (0.0055) | 9.81e-01 | 0.0070 (0.0010) | 0.2905 (0.0418) | 3.68e-12 | 0.0083 (0.0007) | 0.4863 (0.0492) | 4.79e-23 | 0.151 | 0.374 | <1e −300 |
| Cataract-IBS | 0.0032 (0.0051) | 5.29e-01 | 0.0092 (0.0010) | 0.5824 (0.0656) | 7.16e-19 | 0.0077 (0.0007) | 0.2954 (0.0264) | 5.02e-29 | 0.178 | 0.422 | <1e −300 |
| Cataract-DD | -0.0074 (0.0057) | 1.93e-01 | 0.0098 (0.0011) | 0.3417 (0.0389) | 1.58e-18 | 0.0091 (0.0008) | 0.2556 (0.0244) | 1.17e-25 | 0.105 | 0.198 | 4.15e-94 |
| AMD-GORD | 0.0028 (0.0055) | 6.12e-01 | 0.0040 (0.0011) | 0.1465 (0.0405) | 2.98e-04 | 0.0040 (0.0008) | 0.1428 (0.0293) | 1.12e-06 | 0.069 | 0.231 | <1e −300 |
| AMD-IBD | 0.0058 (0.0054) | 2.85e-01 | 0.0017 (0.0009) | 0.1049 (0.0576) | 6.87e-02 | 0.0019 (0.0007) | 0.1015 (0.0366) | 5.51e-03 | 0.011 | 0.071 | <1e −300 |
| AMD-PUD | 0.0034 (0.0057) | 5.49e-01 | 0.0032 (0.0011) | 0.1951 (0.0669) | 3.53e-03 | 0.0029 (0.0007) | 0.1759 (0.0449) | 8.88e-05 | 0.046 | 0.185 | 6.09e-159 |
| AMD-IBS | -0.0032 (0.0053) | 5.48e-01 | 0.0033 (0.0009) | 0.1287 (0.0356) | 3.01e-04 | 0.0030 (0.0007) | 0.1229 (0.0324) | 1.46e-04 | 0.056 | 0.220 | <1e −300 |
| AMD-DD | -0.0033 (0.0055) | 5.48e-01 | 0.0036 (0.0010) | 0.1218 (0.0345) | 4.13e-04 | 0.0031 (0.0007) | 0.0924 (0.0214) | 1.52e-05 | 0.007 | 0.035 | 7.64e-71 |
| PACG-GORD | 0.0031 (0.0055) | 5.74e-01 | 0.0011 (0.0011) | 0.0473 (0.0449) | 2.92e-01 | 0.0018 (0.0007) | 0.0692 (0.0277) | 1.26e-02 | 0.106 | 0.311 | <1e −300 |
| PACG-IBD | 0.0027 (0.0056) | 6.30e-01 | -0.0006 (0.0009) | -0.0448 (0.0617) | 4.68e-01 | -0.0005(0.0007) | -0.0262(0.0376) | 4.86e-01 | 0.008 | 0.035 | 1.66e-52 |
| PACG-PUD | -0.0032 (0.005) | 5.20e-01 | 0.0028 (0.0009) | 0.1998 (0.0620) | 1.28e-03 | 0.0026 (0.0006) | 0.1673 (0.0421) | 7.02e-05 | 0.057 | 0.184 | 7.59e-40 |
| PACG-IBS | -0.0009 (0.0054) | 8.66e-01 | 0.0031 (0.0009) | 0.1441 (0.0431) | 8.39e-04 | 0.0028 (0.0007) | 0.1218 (0.0316) | 1.17e-04 | 0.09 | 0.268 | 2.37e-191 |
| PACG-DD | -0.0112 (0.0054) | 3.80e-02 | 0.0031 (0.0010) | 0.1220 (0.0397) | 2.11e-03 | 0.0024 (0.0007) | 0.0765 (0.0221) | 5.36e-04 | 0.294 | 0.605 | 4.34e-31 |
| Keratitis-GORD | 0.0023 (0.0052) | 6.60e-01 | 0.0023 (0.0009) | 0.2436 (0.0980) | 1.29e-02 | 0.0025 (0.0006) | 0.1534 (0.0374) | 4.19e-05 | 0.019 | 0.070 | 3.00e-04 |
| Keratitis-IBD | 0.0033 (0.0049) | 5.01e-01 | -0.0004 (0.0009) | -0.0672 (0.1538) | 6.62e-01 | 0.0003 (0.0006) | 0.0258 (0.0574) | 6.53e-01 | 0.004 | 0.053 | 1.01e-15 |
| Keratitis-PUD | 0.0069 (0.0047) | 1.42e-01 | 0.0017 (0.0008) | 0.3048 (0.1390) | 2.83e-02 | 0.0029 (0.0006) | 0.3020 (0.0683) | 9.89e-06 | 0.010 | 0.104 | 1.88e-44 |
| Keratitis-IBS | 0.0008 (0.0046) | 8.64e-01 | 0.0030 (0.0009) | 0.3487 (0.1067) | 1.08e-03 | 0.0032 (0.0006) | 0.2272 (0.0469) | 1.24e-06 | 0.004 | 0.022 | 2.73e-59 |
| Keratitis-DD | -0.0054 (0.0053) | 3.08e-01 | 0.0029 (0.0010) | 0.2843 (0.0931) | 2.27e-03 | 0.0029 (0.0007) | 0.1488 (0.0383) | 1.01e-04 | 0.007 | 0.021 | 5.39e-45 |

Note: Abbreviations: AMD, Age-related macular degeneration; DR, Diabetic retinopathy; DED, Dry Eye disease; PACG, Primary angle-closure glaucoma; GORD, Gastro-oesophageal reflux disease; GPA, genetic analysis incorporating pleiotropy and annotation method; IBD, inflammatory bowel disease; IBS, irritable bowel syndrome; LDSC, linkage disequilibrium score regression; PAR, pleiotropy association ratio; PM 11, proportion of genetic variants associated with both traits.

^a^Genetic correlation and genetic overlap were estimated by LDSC/HDL and GPA methods, respectively. Bonferroni-corrected significance threshold was set at P < 1.25 × 10^−3^ (.05/40), producing a final union set of 40 pairwise traits with significant genetic correlation or genetic overlap for subsequent analysis. Significant results with P value reaching the Bonferroni corrected threshold are highlighted in red.

^b^ We introduced PAR as PM 11/(PM 10 + PM 01 + PM 11) to represent the proportion of pleiotropic single nucleotide polymorphisms (SNPs) associated with both traits against the proportion of SNPs associated with at least 1 trait.

**Table S3.** Summary of genome-wide significant pleiotropic SNPs and FUMA-Annotated pleiotropic genomic risk loci for each pair of traits

| **Trait pair** | **Total SNPs** | **No. SNP**  **-PLACO^b^** | **No. Loci**  **-FUMA^c^** | **No. region^d^** | **Chromosomal region** |
| --- | --- | --- | --- | --- | --- |
| DR-GORD | 7935702 | 3835 | 24 | 23 | 2p21, 2p16.1, 2q36.3, 3p21.31, 3q27.2, 4p16.1, 4q24, 6p21.1, 10q22.3, 10q23.33, 10q25.2, 11p15.4, 11q13.1, 12q14.3, 12q24.31, 13q13.3, 14q24.3, 16q12.2, 17q21.31, 17q21.32, 19p13.11, 19q13.32, 20q11.22 |
| DR-IBD | 9295875 | 3784 | 23 | 20 | 1p31.3, 1p31.1, 1q32.1, 2p21, 2p16.1, 2q31.1, 3q27.2, 4q24, 6p24.3, 6p22.3, 6p22.1, 7q36.3, 8p11.21, 8q23.3, 9q34.3, 10q23.33, 10q25.2, 11q14.3, 15q24.3, 20q12 |
| DR-PUD | 7935702 | 794 | 28 | 27 | 1q25.3, 1q32.3, 2p23.3, 2p21, 2p16.1, 2q24.3, 3p25.2, 3p24.3, 4p16.1, 4q31.22, 5q13.3, 5q21.1, 5q33.3, 6p22.3, 7p15.1, 7q36.3, 9p21.3, 9q34.2, 10q21.1, 10q22.3, 11p15.4, 12p13.32, 17q12, 18q21.2, 19q13.32, 19q13.33, 21q21.1 |
| DR-IBS | 7935702 | 3314 | 26 | 25 | 1p34.3, 2p23.3, 2p16.1, 2q36.3, 3p24.3, 3p12.1, 3q22.3, 3q27.1, 3q27.3, 5q21.1, 6p24.3, 6p21.1, 8p23.1, 8q23.3, 9p21.3, 9q33.3, 10q11.23, 10q22.3, 11p15.1, 12q14.3, 15q26.1, 17q21.31, 17q21.32, 20q11.22, 22q12.2 |
| DR-DD | 8734843 | 2538 | 38 | 36 | 2p21, 2p16.1, 2q22.3, 3p25.2, 3q22.3, 4q31.21, 4q31.3, 5p13.2, 5q21.1, 6p24.3, 6p22.3, 6p21.1, 6q16.1, 7p21.2, 7p15.1, 7q22.1, 8q23.3, 8q24.12, 9q33.3, 9q34.2, 10p13, 10q24.33, 10q25.2, 11p15.5, 11p15.4, 11p14.1, 11p11.2, 11q13.1, 12q24.31, 15q15.1, 15q23, 16q23.1, 18q21.2, 18q21.32, 20q12, 21q22.3 |
| DED-GORD | 7937716 | 139 | 5 | 5 | 4q24, 6p22.2, 6p21.32, 11q23.2, 19p13.11 |
| DED-IBD | 9298152 | 228 | 10 | 10 | 1p36.13, 1q23.3, 1q32.1, 3p21.31, 4q24, 5q31.1, 7q31.1, 7q32.1, 10q11.21, 21q21.1 |
| DED-PUD | 7937716 | 68 | 2 | 2 | 10p12.31, 19q13.33 |
| DED-IBS | 7937716 | 166 | 5 | 5 | 2q22.3, 3p14.3, 5p15.31, 11q23.2, 16q21 |
| DED-DD | 8737137 | 319 | 9 | 9 | 1q42.2, 2q22.3, 6q16.1, 7q31.1, 10p12.31, 11p11.2, 18q11.2, 19q13.2, 22q13.2 |
| Uveitis-GORD^a^ | 7936709 | 447 | NA | NA | NA |
| Uveitis-IBD | 9296823 | 2215 | 3 | 3 | 1p31.3, 1q32.1, 21q22.2 |
| Uveitis-PUD | 7936709 | 209 | 2 | 2 | 2p22.1, 19q13.33 |
| Uveitis-IBS | 7936709 | 318 | 1 | 1 | 4q35.2 |
| Uveitis-DD | 8735788 | 88 | 1 | 1 | 2q22.3 |
| Myopia-GORD | 7936889 | 404 | 17 | 16 | 1p34.1, 1q32.1, 4q21.21, 4q22.1, 6p22.3, 8q12.1, 9p22.2, 9q31.3, 10q21.1, 11q14.1, 13q32.3, 14q22.2, 16p13.3, 17p12, 18q12.3, 19q13.32 |
| Myopia-IBD | 9297305 | 201 | 16 | 15 | 1p31.3, 1q32.1, 2p16.1, 3q23, 5p13.1, 8q12.1, 8q21.12, 10q26.13, 11q13.5, 12p13.31, 13q32.3, 14q22.2, 15q14, 16p13.3, 18q12.3 |
| Myopia-PUD | 7936889 | 295 | 14 | 14 | 1p31.1, 1q313, 2p25.3, 2q24.1, 3q23, 6q13, 8q12.1, 11p15.4, 14q22.2, 15q14, 18q22.3, 19q13.33, 19q21.1, 20p12.3 |
| Myopia-IBS | 7936889 | 102 | 5 | 5 | 1q31.3, 3q25.2, 10q26.13, 19p13.3, 22q13.1 |
| Myopia-DD | 8736279 | 900 | 26 | 25 | 1q21.3, 1q32.1, 1q32.2, 1q41, 2p16.1, 2q22.3, 2q35, 3p25.1, 4p16.1, 4q21.21, 5q12.3, 6q22.33, 7q36.3, 8q24.12, 10p12.1, 10p12.33, 10q21.1, 10q22.3, 12p12.31, 15q14, 15q24.1, 17p13.1, 17q11.2, 21q22.3, 22q13.33 |
| Cataract-GORD | 7937725 | 249 | 5 | 5 | 3p21.31, 4p12, 6p22.2, 15q25.1, 19p13.11 |
| Cataract-IBD | 9298175 | 255 | 6 | 6 | 1p36.13, 1p31.3, 1q23.3, 3p23.31, 5p13.1, 7q31.1 |
| Cataract-PUD | 7937725 | 2 | 2 | 2 | 17q21.2, 19q13.41 |
| Cataract-IBS | 7937725 | 42 | 8 | 7 | 1p34.3, 3q26.33, 4p16.2, 4q25, 4q32.1, 9q31.2, 11q23.2 |
| Cataract-DD | 8737146 | 218 | 8 | 8 | 10p12.1, 10q24.33, 11p14.1, 1q42.2, 2q22.3, 6q16.1, 7q11.23, 9q34.2 |
| AMD-GORD | 7275898 | 921 | 5 | 5 | 1q32.2, 6p22.2, 6p22.1, 10q26.13, 19q13.32 |
| AMD-IBD | 7935700 | 2476 | 14 | 12 | 1p31.3, 1q31.3, 1q32.1, 1q32.2, 6p22.1, 7q32.1, 7q36.1, 10q26.13, 15q24.1, 20q13.33, 21q22.2, 22q13.1 |
| AMD-PUD | 9295403 | 477 | 7 | 7 | 1q31.3, 4q21.3, 5q13.3, 9p21.3, 10q26.13, 16q24.1, 19q13.33 |
| AMD-IBS | 7935700 | 1552 | 5 | 5 | 1q31.3, 3q25.5, 10q26.13, 19p13.3, 22q13.1 |
| AMD-DD | 8734375 | 2561 | 22 | 17 | 1p22.2,2q24.3,8q24.3,9p24.3,9p24.2,9p24.1,9p23,9p21.2,11p15.5,11p14.3,13q14.11,16q22.1,18p11.32,18p11.31,18q11.2,19p13.3,19p13.11 |
| PACG-GORD | 7937646 | 17 | 2 | 2 | 4q24, 11q23.2 |
| PACG-IBD | 9298091 | 49 | 2 | 2 | 1p36.13, 7q32.1 |
| PACG-PUD^a^ | 7937646 | 0 | NA | NA | NA |
| PACG-IBS | 7937646 | 29 | 2 | 2 | 6p22.3, 15q13.1 |
| PACG-DD | 8737061 | 232 | 11 | 11 | 1q42.2, 2p16.1, 2q22.3, 3q25.1, 5p13.2, 5q22.1, 10q24.2, 11p14.1, 15q13.1, 15q24.1, 17q23.2 |
| Keratitis-GORD^a^ | 7937715 | 0 | NA | NA | NA |
| Keratitis-IBD | 9298155 | 131 | 4 | 4 | 1p31.3, 1q32.1, 7q31.1, 9q31.2 |
| Keratitis-PUD^a^ | 7937715 | 0 | NA | NA | NA |
| Keratitis-IBS | 7937715 | 116 | 3 | 3 | 1p34.1, 5p15.31, 13q14.3 |
| Keratitis-DD | 8737134 | 299 | 5 | 5 | 1q21.3, 2q22.3, 3q25.1, 10p12.1, 10q24.2 |
| Total |  | 29990 | 366 | 347 |  |

Note: Abbreviations: AMD, Age-related macular degeneration; DR, Diabetic retinopathy; DED, Dry Eye disease; PACG, Primary angle-closure glaucoma; GORD, Gastro-oesophageal reflux disease; GPA, genetic analysis incorporating pleiotropy and annotation method; IBD, inflammatory bowel disease; IBS, irritable bowel syndrome; LDSC, linkage disequilibrium score regression; PAR, pleiotropy association ratio; PM 11, proportion of genetic variants associated with both traits.

^a^ No significant pleiotropic SNPs were identified, thus no pleiotropic loci were detected.

^b^ Number of significant pleiotropic SNPs identified by PLACO.

^c^ Number of pleiotropic genomic risk loci defined by FUMA.

^d^ Number of pleiotropic region defined by FUMA.

**Table S4.** Pleiotropic Genomic Loci Identified by FUMA Using PLACO Results

| **No.** | **Trait-pair** | **CHR** | **BP** | **Region** | **rsID** | **nearestGene** | **Functional**  **annotation** | **CADD** |
| --- | --- | --- | --- | --- | --- | --- | --- | --- |
| 1 | DR-GORD | 2 | 43521167 | 2p21 | rs1322 | *THADA* | intronic | 12.45 |
| 2 | DR-GORD | 2 | 60649143 | 2p16.1 | rs12997266 | *BCL11A* | intergenic | 2.767 |
| 3 | DR-GORD | 2 | 226992454 | 2q36.3 | rs1522811 | *AC068138.1* | intergenic | 3.146 |
| 4 | DR-GORD | 3 | 49774658 | 3p21.31 | rs73077175 | *IP6K1* | intronic | 0.969 |
| 5 | DR-GORD | 3 | 185469491 | 3q27.2 | rs12636310 | *IGF2BP2* | intronic | 1.875 |
| 6 | DR-GORD | 4 | 10452986 | 4p16.1 | rs5028371 | *ZNF518B* | intronic | 2.085 |
| 7 | DR-GORD | 4 | 103188709 | 4q24 | rs13107325 | *SLC39A8* | exonic | 23.1 |
| 8 | DR-GORD | 6 | 43814154 | 6p21.1 | rs7765815 | *SLC39A8* | intergenic | 1.922 |
| 9 | DR-GORD | 10 | 80926036 | 10q22.3 | rs703994 | *ZMIZ1* | intronic | 0.755 |
| 10 | DR-GORD | 10 | 94478104 | 10q23.33 | rs11187145 | *Y_RNA* | intergenic | 0.122 |
| 11 | DR-GORD | 10 | 114847444 | 10q25.2 | rs11451675 | *TCF7L2* | intronic | 11.3 |
| 12 | DR-GORD | 11 | 2840424 | 11p15.4 | rs233448 | *KCNQ1* | intronic | 3.255 |
| 13 | DR-GORD | 11 | 65326154 | 11q13.1 | rs12789028 | *LTBP3* | UTR5 | 12.77 |
| 14 | DR-GORD | 12 | 66383320 | 12q14.3 | rs1585897 | *HMGA2* | intergenic | 4.721 |
| 15 | DR-GORD | 12 | 121892786 | 12q24.31 | rs10849888 | *KDM2B* | intronic | 0.172 |
| 16 | DR-GORD | 13 | 36081761 | 13q13.3 | rs9544488 | *NBEA* | intronic | 5.551 |
| 17 | DR-GORD | 14 | 77384293 | 14q24.3 | rs4903516 | *RP11-488C13.4* | intergenic | 15.02 |
| 18 | DR-GORD | 16 | 53614746 | 16q12.2 | rs12928335 | *RPGRIP1L* | intergenic | 0.558 |
| 19 | DR-GORD | 17 | 44338735 | 17q21.31 | rs2732686 | *RP11-259G18.3* | upstream | 4.268 |
| 20 | DR-GORD | 17 | 47037194 | 17q21.32 | rs11867603 | *GIP* | intronic | 0.653 |
| 21 | DR-GORD | 19 | 18808915 | 19p13.11 | rs7258722 | *CRTC1* | intronic | 9.239 |
| 22 | DR-GORD | 19 | 19436229 | 19p13.11 | rs111234557 | *MAU2* | intronic | 1.791 |
| 23 | DR-GORD | 19 | 45411941 | 19q13.32 | rs429358 | *APOE* | exonic | 12.64 |
| 24 | DR-GORD | 20 | 33256838 | 20q11.22 | rs143220569 | *PIGU* | intronic | 0.822 |
| 25 | DR-IBD | 1 | 67684934 | 1p31.3 | rs4385674 | *C1orf141:IL23R* | intronic | 1.961 |
| 26 | DR-IBD | 1 | 72837239 | 1p31.1 | rs7531118 | *RPL31P12* | intergenic | 11.35 |
| 27 | DR-IBD | 1 | 204426295 | 1q32.1 | rs1008833 | *PIK3C2B* | intronic | 11.47 |
| 28 | DR-IBD | 1 | 206943968 | 1q32.1 | rs3024493 | *IL10* | intronic | 7.722 |
| 29 | DR-IBD | 2 | 43809347 | 2p21 | rs78487399 | *THADA* | intronic | 0.984 |
| 30 | DR-IBD | 2 | 59436587 | 2p16.1 | rs10181565 | *AC007131.1* | intergenic | 0.049 |
| 31 | DR-IBD | 2 | 169706079 | 2q31.1 | rs2140046 | *NOSTRIN:SPC25* | intronic | 4.245 |
| 32 | DR-IBD | 3 | 185515274 | 3q27.2 | rs11708719 | *IGF2BP2* | intronic | 7.079 |
| 33 | DR-IBD | 4 | 103001649 | 4q24 | rs1813006 | *BANK1* | intergenic | 1.848 |
| 34 | DR-IBD | 6 | 7245458 | 6p24.3 | rs1815311 | *RREB1* | intronic | 1.293 |
| 35 | DR-IBD | 6 | 20654300 | 6p22.3 | rs6456366 | *CDKAL1* | intronic | 0.198 |
| 36 | DR-IBD | 6 | 29614419 | 6p22.1 | rs1233370 | *SUMO2P1* | intergenic | 2.33 |
| 37 | DR-IBD | 6 | 41702979 | 6p22.1 | rs11759908 | *TFEB* | intronic | 9.017 |
| 38 | DR-IBD | 6 | 43798981 | 6p22.1 | rs57266556 | *RP11-344J7.2* | intergenic | 10.38 |
| 39 | DR-IBD | 7 | 156970641 | 7q36.3 | rs3802123 | *UBE3C* | intronic | 0.127 |
| 40 | DR-IBD | 8 | 41509259 | 8p11.21 | rs12549902 | *NKX6-3* | upstream | 1.923 |
| 41 | DR-IBD | 8 | 116559435 | 8q23.3 | rs3808434 | *TRPS1* | intronic | 12.23 |
| 42 | DR-IBD | 9 | 139300876 | 9q34.3 | rs11145912 | *SDCCAG3* | intronic | 1.437 |
| 43 | DR-IBD | 10 | 94294291 | 10q23.33 | rs4646955 | *IDE* | intronic | 0.44 |
| 44 | DR-IBD | 10 | 114643471 | 10q25.2 | rs4918784 | *RP11-57H14.3* | intergenic | 4.392 |
| 45 | DR-IBD | 11 | 89970603 | 11q14.3 | rs12789669 | *DISC1FP1* | intergenic | 0.365 |
| 46 | DR-IBD | 15 | 77551445 | 15q24.3 | rs12909924 | *PEAK1* | intronic | 2.556 |
| 47 | DR-IBD | 20 | 39911039 | 20q12 | rs74577821 | *ZHX3* | intronic | 0.398 |
| 48 | DR-PUD | 1 | 182968079 | 1q25.3 | rs12146099 | *RNU6-41P* | intergenic | 0.431 |
| 49 | DR-PUD | 1 | 214177319 | 1q32.3 | rs61320678 | *PROX1* | intronic | 8.237 |
| 50 | DR-PUD | 2 | 27742603 | 2p23.3 | rs780093 | *GCKR* | intronic | 1.541 |
| 51 | DR-PUD | 2 | 43638712 | 2p21 | rs13029250 | *THADA* | intronic | 5.929 |
| 52 | DR-PUD | 2 | 58916786 | 2p16.1 | rs60635548 | *LINC01122* | ncRNA_intronic | 4.023 |
| 53 | DR-PUD | 2 | 60557705 | 2p16.1 | rs7589501 | *AC007381.3* | intergenic | 3.743 |
| 54 | DR-PUD | 2 | 166183577 | 2q24.3 | rs17184707 | *SCN2A* | intergenic | 6.268 |
| 55 | DR-PUD | 3 | 12413339 | 3p25.2 | rs2120825 | *PPARG* | intronic | 3.955 |
| 56 | DR-PUD | 3 | 23457080 | 3p24.3 | rs13094957 | *UBE2E2* | intronic | 1.118 |
| 57 | DR-PUD | 4 | 6277049 | 4p16.1 | rs6833959 | *WFS1* | intronic | 0.022 |
| 58 | DR-PUD | 4 | 147209404 | 4q31.22 | rs13140054 | *SLC10A7* | intronic | 1.43 |
| 59 | DR-PUD | 5 | 76435346 | 5q13.3 | rs7732628 | *ZBED3-AS1* | ncRNA_intronic | 7.67 |
| 60 | DR-PUD | 5 | 101779346 | 5q21.1 | rs79602013 | *SLCO6A1* | intronic | 4.574 |
| 61 | DR-PUD | 5 | 156000025 | 5q33.3 | rs115498181 | *SGCD* | intronic | 4.007 |
| 62 | DR-PUD | 6 | 20536356 | 6p22.3 | rs6905138 | *CDKAL1* | intronic | 3.471 |
| 63 | DR-PUD | 7 | 28187806 | 7p15.1 | rs1635851 | *JAZF1* | intronic | 3.006 |
| 64 | DR-PUD | 7 | 157026633 | 7q36.3 | rs1182442 | *UBE3C* | intronic | 3.853 |
| 65 | DR-PUD | 9 | 22128180 | 9p21.3 | rs12379111 | *CDKN2B-AS1* | intergenic | 2.927 |
| 66 | DR-PUD | 9 | 136132908 | 9q34.2 | rs8176719 | *ABO* | ncRNA_exonic | 15.84 |
| 67 | DR-PUD | 10 | 56639564 | 10q21.1 | rs11592488 | *PCDH15* | intronic | 3.698 |
| 68 | DR-PUD | 10 | 80955067 | 10q22.3 | rs703965 | *ZMIZ1* | intronic | 8.618 |
| 69 | DR-PUD | 11 | 2869452 | 11p15.4 | rs45477500 | *KCNQ1:KCNQ1-AS1* | ncRNA_intronic | 10.33 |
| 70 | DR-PUD | 12 | 4328521 | 12p13.32 | rs117233107 | *CCND2-AS1* | intergenic | 1.4 |
| 71 | DR-PUD | 17 | 37396201 | 17q12 | rs34473775 | *RP11-690G19.4* | ncRNA_intronic | 3.87 |
| 72 | DR-PUD | 18 | 53183396 | 18q21.2 | rs624244 | *TCF4* | intronic | 0.476 |
| 73 | DR-PUD | 19 | 46268902 | 19q13.32 | rs2341097 | *SIX5:AC074212.5* | exonic | 22.2 |
| 74 | DR-PUD | 19 | 49206674 | 19q13.33 | rs601338 | *FUT2* | exonic | 52 |
| 75 | DR-PUD | 21 | 16574661 | 21q21.1 | rs60893125 | *AF127577.12* | intergenic | 1.735 |
| 76 | DR-IBS | 1 | 39942297 | 1p34.3 | rs61779310 | *MACF1* | intronic | 0.734 |
| 77 | DR-IBS | 2 | 27730940 | 2p23.3 | rs1260326 | *GCKR* | exonic | 13.22 |
| 78 | DR-IBS | 2 | 58879493 | 2p16.1 | rs6724384 | *LINC01122* | ncRNA_intronic | 3.832 |
| 79 | DR-IBS | 2 | 227171148 | 2q36.3 | rs13012754 | *AC068138.1* | intergenic | 0.27 |
| 80 | DR-IBS | 3 | 23370914 | 3p24.3 | rs9848331 | *rs9848331* | intronic | 4.385 |
| 81 | DR-IBS | 3 | 84512131 | 3p12.1 | rs4334651 | *AC107025.1* | intergenic | 1.363 |
| 82 | DR-IBS | 3 | 135785491 | 3q22.3 | rs10048942 | *PPP2R3A* | intronic | 3.106 |
| 83 | DR-IBS | 3 | 184066556 | 3q27.1 | rs9869577 | *EIF2B5:CLCN2* | intronic | 4.504 |
| 84 | DR-IBS | 3 | 186661006 | 3q27.3 | rs9860157 | *ST6GAL1* | intronic | 0.357 |
| 85 | DR-IBS | 5 | 102754950 | 5q21.1 | rs13188193 | *CTD-2154H6.1* | intergenic | 2.513 |
| 86 | DR-IBS | 6 | 7230680 | 6p24.3 | rs9502564 | *RREB1* | exonic | 23.2 |
| 87 | DR-IBS | 6 | 41995361 | 6p21.1 | rs4365924 | *CCND3* | intronic | 0.38 |
| 88 | DR-IBS | 8 | 11051256 | 8p23.1 | rs2409729 | *XKR6* | intronic | 0.656 |
| 89 | DR-IBS | 8 | 116588546 | 8q23.3 | rs2049865 | *TRPS1* | intronic | 0.925 |
| 90 | DR-IBS | 9 | 22132576 | 9p21.3 | rs78432974 | *CDKN2B-AS1* | intergenic | 0.868 |
| 91 | DR-IBS | 9 | 126714308 | 9q33.3 | rs4838083 | *DENND1A* | intergenic | 0.325 |
| 92 | DR-IBS | 10 | 52443318 | 10q11.23 | rs200803583 | *NUTM2HP* | ncRNA_exonic | 2.513 |
| 93 | DR-IBS | 10 | 80971810 | 10q22.3 | rs2802362 | *ZMIZ1* | intronic | 2.535 |
| 94 | DR-IBS | 11 | 17405617 | 11p15.1 | rs1002226 | *RP1-239B22.5* | downstream | 5.762 |
| 95 | DR-IBS | 12 | 66351826 | 12q14.3 | rs1351394 | *HMGA2* | UTR3 | 6.416 |
| 96 | DR-IBS | 15 | 90394356 | 15q26.1 | rs4932148 | *AP3S2* | intronic | 0.397 |
| 97 | DR-IBS | 15 | 91539050 | 15q26.1 | rs28404354 | *PRC1* | upstream | 0.99 |
| 98 | DR-IBS | 17 | 41375509 | 17q21.31 | rs111274735 | *LINC00854* | ncRNA_intronic | 0.756 |
| 99 | DR-IBS | 17 | 47042840 | 17q21.32 | rs28627277 | *GIP* | intronic | 0.149 |
| 100 | DR-IBS | 20 | 32567561 | 20q11.22 | rs60012358 | *RP5-1125A11.1* | intergenic | 0.717 |
| 101 | DR-IBS | 22 | 30422865 | 22q12.2 | rs41172 | *MTMR3:CTA-85E5.10* | ncRNA_intronic | 0.971 |
| 102 | DR-DD | 2 | 43907630 | 2p21 | rs7560838 | *PLEKHH2:AC011242.5* | ncRNA_exonic | 3.363 |
| 103 | DR-DD | 2 | 59319706 | 2p16.1 | rs7594743 | *LINC01122* | intergenic | 0.603 |
| 104 | DR-DD | 2 | 60579624 | 2p16.1 | rs12999941 | *AC007381.3* | ncRNA_intronic | 4.634 |
| 105 | DR-DD | 2 | 144349201 | 2q22.3 | rs368217240 | *ARHGAP15-RP11* | ncRNA_intronic | 0.958 |
| 106 | DR-DD | 3 | 12413339 | 3p25.2 | rs2120825 | *PPARG* | intronic | 3.955 |
| 107 | DR-DD | 3 | 138076975 | 3q22.3 | rs4678411 | *MRAS* | intronic | 1.731 |
| 108 | DR-DD | 4 | 145659064 | 4q31.21 | rs11727676 | *HHIP* | exonic | 12.31 |
| 109 | DR-DD | 4 | 151171269 | 4q31.3 | rs2305980 | *DCLK2* | intronic | 12.19 |
| 110 | DR-DD | 5 | 37778273 | 5p13.2 | rs2973068 | *AC008869.1* | intergenic | 1.287 |
| 111 | DR-DD | 5 | 102754950 | 5q21.1 | rs13188193 | *CTD-2154H6.1* | intergenic | 2.513 |
| 112 | DR-DD | 6 | 7231843 | 6p24.3 | rs9379084 | *RREB1* | exonic | 32 |
| 113 | DR-DD | 6 | 20765686 | 6p22.3 | rs201464451 | *CDKAL1:RP3-348I23.2* | ncRNA_intronic | 2.274 |
| 114 | DR-DD | 6 | 43308363 | 6p21.1 | rs1563788 | *ZNF318* | intronic | 0.2 |
| 115 | DR-DD | 6 | 98440843 | 6q16.1 | rs5878501 | *RP11-436D23.1* | ncRNA_intronic | 1.752 |
| 116 | DR-DD | 7 | 15047280 | 7p21.2 | rs4719430 | *AC006045.3* | intergenic | 0.317 |
| 117 | DR-DD | 7 | 28247511 | 7p15.1 | rs11448038 | *JAZF1-AS1* | ncRNA_intronic | 2.112 |
| 118 | DR-DD | 7 | 102429288 | 7q22.1 | rs7794717 | *FAM185A* | intronic | 2.214 |
| 119 | DR-DD | 8 | 116588546 | 8q23.3 | rs2049865 | *TRPS1* | intronic | 0.925 |
| 120 | DR-DD | 8 | 122267821 | 8q24.12 | rs1553506 | *AC027238.1* | intergenic | 0.664 |
| 121 | DR-DD | 9 | 126263312 | 9q33.3 | rs12236873 | *DENND1A* | intronic | 1.739 |
| 122 | DR-DD | 9 | 136132908 | 9q34.2 | rs8176719 | *ABO* | ncRNA_exonic | 15.84 |
| 123 | DR-DD | 10 | 12309268 | 10p13 | rs11257658 | *RN7SL232P* | intergenic | 0.899 |
| 124 | DR-DD | 10 | 105670849 | 10q24.33 | rs3752946 | *OBFC1* | intronic | 2.234 |
| 125 | DR-DD | 10 | 114891443 | 10q25.2 | rs11599737 | *TCF7L2* | intronic | 10.82 |
| 126 | DR-DD | 11 | 1711588 | 11p15.5 | rs2334418 | *AP006285.6* | intergenic | 1.31 |
| 127 | DR-DD | 11 | 2852886 | 11p15.4 | rs234859 | *KCNQ1* | intronic | 2.907 |
| 128 | DR-DD | 11 | 27690399 | 11p14.1 | rs2353487 | *BDNF-AS:BDNF* | ncRNA_intronic | 5.477 |
| 129 | DR-DD | 11 | 47669249 | 11p11.2 | rs7107792 | *MTCH2* | intergenic | 6.276 |
| 130 | DR-DD | 11 | 65644027 | 11q13.1 | rs583887 | *EFEMP2* | intergenic | 2.167 |
| 131 | DR-DD | 12 | 121463562 | 12q24.31 | rs11408808 | *OASL* | intronic | 0.107 |
| 132 | DR-DD | 12 | 121856217 | 12q24.31 | rs889970 | *RNF34* | intronic | 0.803 |
| 133 | DR-DD | 15 | 40645133 | 15q15.1 | rs7164606 | *PHGR1* | intronic | 2.634 |
| 134 | DR-DD | 15 | 68454480 | 15q23 | rs71400388 | *PIAS1* | intronic | 7.372 |
| 135 | DR-DD | 16 | 75301974 | 16q23.1 | rs72804106 | *BCAR1* | upstream | 9.511 |
| 136 | DR-DD | 18 | 53183396 | 18q21.2 | rs624244 | *TCF4* | intronic | 0.476 |
| 137 | DR-DD | 18 | 57824038 | 18q21.32 | rs7240682 | *RP11-795H16.3* | ncRNA_intronic | 6.492 |
| 138 | DR-DD | 20 | 39832628 | 20q12 | rs17265513 | *ZHX3* | exonic | 22 |
| 139 | DR-DD | 21 | 47306241 | 21q22.3 | rs9976088 | *PCBP3* | intronic | 7.097 |
| 140 | Uveitis-IBD | 1 | 67705958 | 1p31.3 | rs11209026 | *IL23R* | exonic | 26.5 |
| 141 | Uveitis-IBD | 1 | 200884985 | 1q32.1 | rs905634 | *C1orf106* | downstream | 2.128 |
| 142 | Uveitis-IBD | 21 | 40465534 | 21q22.2 | rs2836878 | *RPL23AP12* | intergenic | 0.842 |
| 143 | Uveitis-PUD | 2 | 40858754 | 2p22.1 | rs732707 | *SLC8A1* | intergenic | 4.436 |
| 144 | Uveitis-PUD | 19 | 49201217 | 19q13.33 | rs368565 | *FUT2* | intronic | 2.584 |
| 145 | Uveitis-IBS | 4 | 188838702 | 4q35.2 | rs72718909 | *RP11-713C19.2* | intergenic | 4.532 |
| 146 | Uveitis-DD | 2 | 144379627 | 2q22.3 | rs4146022 | *ARHGAP15* | intronic | 17.81 |
| 147 | AMD-GORD | 1 | 207977083 | 1q32.2 | rs7523273 | *C1orf132* | ncRNA_exonic | 0.24 |
| 148 | AMD-GORD | 6 | 26668216 | 6p22.2 | rs62396201 | *ZNF322* | intergenic | 1.331 |
| 149 | AMD-GORD | 6 | 28214698 | 6p22.1 | rs17720293 | *ZKSCAN4* | intronic | 10.52 |
| 150 | AMD-GORD | 10 | 124202675 | 10q26.13 | rs111266031 | *PLEKHA1* | intergenic | 3.489 |
| 151 | AMD-GORD | 19 | 45411941 | 19q13.32 | rs429358 | *APOE* | exonic | 12.64 |
| 152 | AMD-IBD | 1 | 67719129 | 1p31.3 | rs1343151 | *IL23R* | intronic | 6.93 |
| 153 | AMD-IBD | 1 | 195741951 | 1q31.3 | rs12130962 | *RP11-476B1.1* | intergenic | 1.985 |
| 154 | AMD-IBD | 1 | 196449432 | 1q31.3 | rs188357630 | *KCNT2* | intronic | 0.913 |
| 155 | AMD-IBD | 1 | 197570844 | 1q31.3 | rs12144001 | *DENND1B* | intronic | 0.833 |
| 156 | AMD-IBD | 1 | 200890353 | 1q32.1 | rs296544 | *MROH3P* | ncRNA_intronic | 1.059 |
| 157 | AMD-IBD | 1 | 208018219 | 1q32.2 | rs2745979 | *C1orf132* | ncRNA_intronic | 0.134 |
| 158 | AMD-IBD | 6 | 29609951 | 6p22.1 | rs1233372 | *SUMO2P1* | intergenic | 0.317 |
| 159 | AMD-IBD | 7 | 128576086 | 7q32.1 | rs3757387 | *IRF5* | intergenic | 1.106 |
| 160 | AMD-IBD | 7 | 148398634 | 7q36.1 | rs243546 | *CUL1* | intronic | 2.615 |
| 161 | AMD-IBD | 10 | 124106025 | 10q26.13 | rs78200813 | *RNU6-728P* | downstream | 0.726 |
| 162 | AMD-IBD | 15 | 75052495 | 15q24.1 | rs12903896 | *CYP1A2* | intergenic | 4.296 |
| 163 | AMD-IBD | 20 | 62322699 | 20q13.33 | rs6011033 | *RTEL1:RTEL1-TNFRSF6B* | intronic | 0.1 |
| 164 | AMD-IBD | 21 | 40467643 | 21q22.2 | rs2836884 | *RPL23AP12* | intergenic | 1.443 |
| 165 | AMD-IBD | 22 | 39659088 | 22q13.1 | rs4820371 | *AL031590.1* | intergenic | 0.247 |
| 166 | AMD-PUD | 1 | 196721105 | 1q31.3 | rs11585965 | *CFH* | intergenic | 0.001 |
| 167 | AMD-PUD | 4 | 87240157 | 4q21.3 | rs17449582 | *MAPK10* | intronic | 3.236 |
| 168 | AMD-PUD | 5 | 76430636 | 5q13.3 | rs7707527 | *ZBED3-AS1* | ncRNA_intronic | 3.944 |
| 169 | AMD-PUD | 9 | 22132698 | 9p21.3 | rs10965246 | *CDKN2B-AS1* | intergenic | 0.815 |
| 170 | AMD-PUD | 10 | 124028529 | 10q26.13 | rs72826311 | *BTBD16* | intergenic | 0.002 |
| 171 | AMD-PUD | 16 | 86549101 | 16q24.1 | rs370509910 | *FOXF1* | intergenic | 9.058 |
| 172 | AMD-PUD | 19 | 49232226 | 19q13.33 | rs2287922 | *RASIP1* | exonic | 26.2 |
| 173 | AMD-IBS | 1 | 197030087 | 1q31.3 | rs17514253 | *F13B* | exonic | 0.765 |
| 174 | AMD-IBS | 3 | 153957088 | 3q25.5 | rs408419 | *ARHGEF26* | intronic | 2.873 |
| 175 | AMD-IBS | 10 | 124091572 | 10q26.13 | rs3887220 | *BTBD16* | intronic | 2.006 |
| 176 | AMD-IBS | 19 | 6730855 | 19p13.3 | rs2279623 | *GPR108* | intronic | 5 |
| 177 | AMD-IBS | 22 | 39644273 | 22q13.1 | rs130651 | *PDGFB* | intergenic | 2.266 |
| 178 | AMD-DD | 1 | 89495245 | 1p22.2 | rs112500140 | *GBP3* | intergenic | 6.313 |
| 179 | AMD-DD | 2 | 168560474 | 2q24.3 | rs115488970 | *CTAGE14P* | intergenic | 1.277 |
| 180 | AMD-DD | 8 | 141549575 | 8q24.3 | rs2447389 | *AGO2* | intronic | 7.539 |
| 181 | AMD-DD | 9 | 114565 | 9p24.3 | rs1629857 | *RP11-143M1.4* | upstream | 3.139 |
| 182 | AMD-DD | 9 | 1581468 | 9p24.3 | rs583230 | *RNA5SP279* | intergenic | 3.852 |
| 183 | AMD-DD | 9 | 3694163 | 9p24.2 | rs16919460 | *RP11-509J21.3* | intergenic | 0.112 |
| 184 | AMD-DD | 9 | 4774015 | 9p24.1 | rs296859 | *RP11-307I14.4* | intergenic | 0.488 |
| 185 | AMD-DD | 9 | 6625864 | 9p24.1 | rs2578273 | *GLDC* | intronic | 2.766 |
| 186 | AMD-DD | 9 | 7026624 | 9p24.1 | rs76752978 | *KDM4C* | intronic | 6.662 |
| 187 | AMD-DD | 9 | 10749606 | 9p23 | rs1928727 | *RP11-421B23.2* | intergenic | 4.566 |
| 188 | AMD-DD | 9 | 25999871 | 9p21.2 | rs2171101 | *RP11-477G9.1* | intergenic | 1.639 |
| 189 | AMD-DD | 11 | 495057 | 11p15.5 | rs67912009 | *RNH1* | intronic | 1.144 |
| 190 | AMD-DD | 11 | 870524 | 11p15.5 | rs10466744 | *CHID1* | intronic | 2.444 |
| 191 | AMD-DD | 11 | 24152669 | 11p14.3 | rs76747679 | *RP11-2F20.1* | intergenic | 4.473 |
| 192 | AMD-DD | 13 | 42029797 | 13q14.11 | rs12430400 | *RGCC* | intergenic | 1.618 |
| 193 | AMD-DD | 16 | 66709086 | 16q22.1 | rs113573226 | *CMTM4* | intronic | 0.32 |
| 194 | AMD-DD | 18 | 659958 | 18p11.32 | rs2847613 | *TYMS* | intronic | 0.26 |
| 195 | AMD-DD | 18 | 4195337 | 18p11.31 | rs1546707 | *DLGAP1* | intronic | 3.408 |
| 196 | AMD-DD | 18 | 24213499 | 18q11.2 | rs76748134 | *KCTD1* | intronic | 0.777 |
| 197 | AMD-DD | 19 | 392705 | 19p13.3 | rs150394100 | *AC010641.1* | intergenic | 0.473 |
| 198 | AMD-DD | 19 | 646357 | 19p13.3 | rs150383413 | *RNF126* | intergenic | 1.939 |
| 199 | AMD-DD | 19 | 18606807 | 19p13.11 | rs34937778 | *ELL* | intronic | 1.635 |
| 200 | Cataract-GORD | 3 | 50242387 | 3p21.31 | rs1858828 | *SLC38A3* | upstream | 5.173 |
| 201 | Cataract-GORD | 4 | 47350953 | 4p12 | rs13104400 | *GABRB1* | intronic | 6.577 |
| 202 | Cataract-GORD | 6 | 26497520 | 6p22.2 | rs2024970 | *BTN1A1* | intergenic | 4.234 |
| 203 | Cataract-GORD | 15 | 81061267 | 15q25.1 | rs12437863 | *KIAA1199* | intergenic | 0.839 |
| 204 | Cataract-GORD | 19 | 19488718 | 19p13.11 | rs12973258 | *GATAD2A* | intergenic | 4.817 |
| 205 | Cataract-IBD | 1 | 20142413 | 1p36.13 | rs3820330 | *RP11-91K11.2* | ncRNA_intronic | 3.608 |
| 206 | Cataract-IBD | 1 | 67689608 | 1p31.3 | rs41396545 | *C1orf141:IL23R* | intronic | 7.889 |
| 207 | Cataract-IBD | 1 | 161479745 | 1q23.3 | rs1801274 | *FCGR2A* | exonic | 0.979 |
| 208 | Cataract-IBD | 3 | 49674458 | 3p23.31 | rs9862080 | *BSN* | intronic | 8.347 |
| 209 | Cataract-IBD | 5 | 40419429 | 5p13.1 | rs2084031 | *AC108105.1* | intergenic | 4.023 |
| 210 | Cataract-IBD | 7 | 107499725 | 7q31.1 | rs6964893 | *DLD* | intergenic | 3.024 |
| 211 | Cataract-PUD | 17 | 39867248 | 17q21.2 | rs34074411 | *JUP* | intronic | 2.405 |
| 212 | Cataract-PUD | 19 | 51802650 | 19q13.41 | rs1710353 | *IGLON5* | intergenic | 1.544 |
| 213 | Cataract-IBS | 1 | 39981740 | 1p34.3 | rs41267043 | *BMP8A:OXCT2P1* | ncRNA_exonic | 12.13 |
| 214 | Cataract-IBS | 3 | 181938437 | 3q26.33 | rs7611110 | *RP11-416O18.2* | intergenic | 3.817 |
| 215 | Cataract-IBS | 4 | 5410382 | 4p16.2 | rs7670828 | *STK32B* | intronic | 0.785 |
| 216 | Cataract-IBS | 4 | 112303764 | 4q25 | rs147098535 | *RNU6-289P* | intergenic | 0.217 |
| 217 | Cataract-IBS | 4 | 158035818 | 4q32.1 | rs11100094 | *GLRB* | intronic | 0.011 |
| 218 | Cataract-IBS | 9 | 109011615 | 9q31.2 | rs2818292 | *RP11-308N19.1* | intergenic | 0.108 |
| 219 | Cataract-IBS | 11 | 112852759 | 11q23.2 | rs1940725 | *NCAM1* | intronic | 4.766 |
| 220 | Cataract-IBS | 11 | 113451229 | 11q23.2 | rs4319542 | *DRD2* | intergenic | 2.752 |
| 221 | Cataract-DD | 1 | 234352899 | 1q42.2 | rs4333882 | *SLC35F3* | intronic | 3.843 |
| 222 | Cataract-DD | 2 | 144425160 | 2q22.3 | rs138141174 | *ARHGAP15* | intronic | 0.863 |
| 223 | Cataract-DD | 6 | 98369230 | 6q16.1 | rs4339469 | *RP11-436D23.1* | ncRNA_intronic | 0.442 |
| 224 | Cataract-DD | 7 | 73480805 | 7q11.23 | rs2528794 | *ELN* | intronic | 0.808 |
| 225 | Cataract-DD | 9 | 136132908 | 9q34.2 | rs8176719 | *ABO* | ncRNA_exonic | 15.84 |
| 226 | Cataract-DD | 10 | 25864681 | 10p12.1 | rs12268257 | *GPR158* | intronic | 7.124 |
| 227 | Cataract-DD | 10 | 105639514 | 10q24.33 | rs10748858 | *RP11-541N10.3* | ncRNA_exonic | 4.909 |
| 228 | Cataract-DD | 11 | 27690399 | 11p14.1 | rs2353487 | *BDNF-AS:BDNF* | ncRNA_intronic | 5.477 |
| 229 | DED-GORD | 4 | 103188709 | 4q24 | rs13107325 | *SLC39A8* | exonic | 23.1 |
| 230 | DED-GORD | 6 | 26483048 | 6p22.2 | rs7755997 | *BTN2A1* | intergenic | 1.687 |
| 231 | DED-GORD | 6 | 33460609 | 6p21.32 | rs79984539 | *ZBTB9* | intergenic | 3.121 |
| 232 | DED-GORD | 11 | 112911839 | 11q23.2 | rs7942723 | *NCAM1* | intronic | 8.483 |
| 233 | DED-GORD | 19 | 18832950 | 19p13.11 | rs12462498 | *CRTC1* | intronic | 4.233 |
| 234 | DED-IBD | 1 | 20177398 | 1p36.13 | rs72658556 | *RP11-91K11.2* | intergenic | 6.847 |
| 235 | DED-IBD | 1 | 161477028 | 1q23.3 | rs4657040 | *FCGR2A* | intronic | 3.777 |
| 236 | DED-IBD | 1 | 206955041 | 1q32.1 | rs3122605 | *IL10* | intergenic | 3.83 |
| 237 | DED-IBD | 3 | 49721532 | 3p21.31 | rs3197999 | *MST1* | exonic | 21 |
| 238 | DED-IBD | 4 | 103112470 | 4q24 | rs6855246 | *SLC39A8* | intergenic | 0.313 |
| 239 | DED-IBD | 5 | 131462836 | 5q31.1 | rs11749300 | *AC063976.1* | intergenic | 10.15 |
| 240 | DED-IBD | 7 | 107586254 | 7q31.1 | rs2237690 | *LAMB1* | intronic | 2.68 |
| 241 | DED-IBD | 7 | 128579666 | 7q32.1 | rs3823536 | *IRF5* | intronic | 2.245 |
| 242 | DED-IBD | 10 | 43612226 | 10q11.21 | rs760466 | *RET* | intronic | 0.47 |
| 243 | DED-IBD | 21 | 16812599 | 21q21.1 | rs1297260 | *AJ006998.2* | intergenic | 4.359 |
| 244 | DED-PUD | 10 | 21824619 | 10p12.31 | rs10828248 | *MLLT10* | intronic | 3.906 |
| 245 | DED-PUD | 19 | 49218060 | 19q13.33 | rs35866622 | *MAMSTR* | intronic | 13.01 |
| 246 | DED-IBS | 2 | 144391918 | 2q22.3 | rs28469251 | *ARHGAP15* | intronic | 0.602 |
| 247 | DED-IBS | 3 | 58446010 | 3p14.3 | rs7631010 | *PDHB* | intergenic | 1.966 |
| 248 | DED-IBS | 5 | 7194421 | 5p15.31 | rs150079703 | *RP11-122F24.1* | intergenic | 1.829 |
| 249 | DED-IBS | 11 | 112905039 | 11q23.2 | rs7128314 | *NCAM1* | intronic | 0.888 |
| 250 | DED-IBS | 16 | 60721629 | 16q21 | rs35418299 | *GNPATP* | intergenic | 7.74 |
| 251 | DED-DD | 1 | 234352899 | 1q42.2 | rs4333882 | *SLC35F3* | intronic | 3.843 |
| 252 | DED-DD | 2 | 144391918 | 2q22.3 | rs28469251 | *ARHGAP15* | intronic | 0.602 |
| 253 | DED-DD | 6 | 98365289 | 6q16.1 | rs4839715 | *RP11-436D23.1* | ncRNA_intronic | 3.363 |
| 254 | DED-DD | 7 | 113999637 | 7q31.1 | rs2690837 | *FOXP2* | intronic | 1.196 |
| 255 | DED-DD | 10 | 21830104 | 10p12.31 | rs11012732 | *MLLT10* | intronic | 2.739 |
| 256 | DED-DD | 11 | 47441356 | 11p11.2 | rs3781628 | *PSMC3* | intronic | 0.814 |
| 257 | DED-DD | 18 | 20010871 | 18q11.2 | rs12455655 | *RP11-863N1.4* | intergenic | 4.052 |
| 258 | DED-DD | 19 | 38815217 | 19q13.2 | rs35478630 | *KCNK6* | intronic | 2.113 |
| 259 | DED-DD | 22 | 41062014 | 22q13.2 | rs5995893 | *GAPDHP37* | intergenic | 0.126 |
| 260 | Keratitis-IBD | 1 | 67699915 | 1p31.3 | rs79755370 | *IL23R* | intronic | 1.948 |
| 261 | Keratitis-IBD | 1 | 206943968 | 1q32.1 | rs3024493 | *IL10* | intronic | 7.722 |
| 262 | Keratitis-IBD | 7 | 107476913 | 7q31.1 | rs990107 | *PIGCP2* | intergenic | 12.76 |
| 263 | Keratitis-IBD | 9 | 110242366 | 9q31.2 | rs10739246 | *KLF4* | intergenic | 3.866 |
| 264 | Keratitis-IBS | 1 | 44801248 | 1p34.1 | rs12063694 | *ERI3* | intronic | 4.571 |
| 265 | Keratitis-IBS | 5 | 7241924 | 5p15.31 | rs4562016 | *RP11-404K5.3* | intergenic | 0.224 |
| 266 | Keratitis-IBS | 13 | 53939598 | 13q14.3 | rs5803650 | *AL450423.1* | intergenic | 2.94 |
| 267 | Keratitis-DD | 1 | 151979770 | 1q21.3 | rs2012674 | *NBPF18P* | ncRNA_intronic | 2.199 |
| 268 | Keratitis-DD | 2 | 144325926 | 2q22.3 | rs4372823 | *ARHGAP15:RP11-570L15.2:RP11-570L15.1* | ncRNA_intronic | 8.305 |
| 269 | Keratitis-DD | 3 | 151075674 | 3q25.1 | rs6790448 | *MED12L:P2RY12* | intronic | 5.021 |
| 270 | Keratitis-DD | 10 | 25793302 | 10p12.1 | rs943985 | *GPR158* | intronic | 0.387 |
| 271 | Keratitis-DD | 10 | 101360807 | 10q24.2 | rs35562233 | *snoU13* | upstream | 2.834 |
| 272 | Myopia-GORD | 1 | 46611044 | 1p34.1 | rs1319055 | *PIK3R3* | intronic | 2.107 |
| 273 | Myopia-GORD | 1 | 200366457 | 1q32.1 | rs6664603 | *ZNF281* | intergenic | 0.426 |
| 274 | Myopia-GORD | 4 | 81977699 | 4q21.21 | rs77285094 | *BMP3* | UTR3 | 1.279 |
| 275 | Myopia-GORD | 4 | 89751858 | 4q22.1 | rs7660000 | *FAM13A* | intronic | 3.738 |
| 276 | Myopia-GORD | 6 | 22057566 | 6p22.3 | rs55775505 | *CASC15* | ncRNA_intronic | 1.045 |
| 277 | Myopia-GORD | 8 | 60186467 | 8q12.1 | rs12547193 | *SNORA51* | intergenic | 5.701 |
| 278 | Myopia-GORD | 8 | 60580660 | 8q12.1 | rs10504295 | *RP11-379I19.3* | intergenic | 0.812 |
| 279 | Myopia-GORD | 9 | 18363396 | 9p22.2 | rs2791442 | *RP11-570H19.2* | intergenic | 18.47 |
| 280 | Myopia-GORD | 9 | 111864892 | 9q31.3 | rs113168638 | *TMEM245* | intronic | 1.499 |
| 281 | Myopia-GORD | 10 | 60365975 | 10q21.1 | rs12258523 | *BICC1* | intronic | 1.898 |
| 282 | Myopia-GORD | 11 | 83246281 | 11q14.1 | rs12281149 | *DLG2* | intronic | 4.213 |
| 283 | Myopia-GORD | 13 | 100769870 | 13q32.3 | rs57756661 | *PCCA* | intronic | 0.64 |
| 284 | Myopia-GORD | 14 | 54415291 | 14q22.2 | rs12898159 | *MIR5580* | upstream | 0.931 |
| 285 | Myopia-GORD | 16 | 7356555 | 16p13.3 | rs8060728 | *RBFOX1* | intronic | 4.674 |
| 286 | Myopia-GORD | 17 | 11406081 | 17p12 | rs2969185 | *SHISA6* | intronic | 3.363 |
| 287 | Myopia-GORD | 18 | 42881004 | 18q12.3 | rs12455689 | *SLC14A2* | intronic | 3.798 |
| 288 | Myopia-GORD | 19 | 45411941 | 19q13.32 | rs429358 | *APOE* | exonic | 12.64 |
| 289 | Myopia-IBD | 1 | 67685443 | 1p31.3 | rs7518660 | *C1orf141:IL23R* | intronic | 0.051 |
| 290 | Myopia-IBD | 1 | 200311674 | 1q32.1 | rs2790110 | *LINC00862* | ncRNA_exonic | 1.899 |
| 291 | Myopia-IBD | 1 | 201012597 | 1q32.1 | rs41267497 | *CACNA1S* | exonic | 0.127 |
| 292 | Myopia-IBD | 2 | 56102744 | 2p16.1 | rs11899888 | *EFEMP1* | intronic | 3.249 |
| 293 | Myopia-IBD | 3 | 141158614 | 3q23 | rs7636914 | *ZBTB38* | intronic | 2.181 |
| 294 | Myopia-IBD | 5 | 40440063 | 5p13.1 | rs7713270 | *AC108105.1* | intergenic | 0.44 |
| 295 | Myopia-IBD | 8 | 59852429 | 8q12.1 | rs7813733 | *TOX* | intronic | 12.21 |
| 296 | Myopia-IBD | 8 | 78648781 | 8q21.12 | rs1001947 | *RP11-38H17.1* | intergenic | 1.16 |
| 297 | Myopia-IBD | 10 | 124230750 | 10q26.13 | rs2672592 | *HTRA1* | intronic | 0.97 |
| 298 | Myopia-IBD | 11 | 76292573 | 11q13.5 | rs7126418 | *RP11-672A2.7* | intergenic | 9.307 |
| 299 | Myopia-IBD | 12 | 9313304 | 12p13.31 | rs7968679 | *PZP* | intronic | 0.678 |
| 300 | Myopia-IBD | 13 | 101175664 | 13q32.3 | rs837323 | *PCCA* | intronic | 18.17 |
| 301 | Myopia-IBD | 14 | 54422399 | 14q22.2 | rs2738265 | *BMP4* | intronic | 8.101 |
| 302 | Myopia-IBD | 15 | 35010136 | 15q14 | rs4924158 | *GJD2* | intergenic | 19 |
| 303 | Myopia-IBD | 16 | 7459211 | 16p13.3 | rs55977430 | *RBFOX1* | intronic | 7.259 |
| 304 | Myopia-IBD | 18 | 42888797 | 18q12.3 | rs9952980 | *SLC14A2* | intronic | 11.19 |
| 305 | Myopia-PUD | 1 | 72829623 | 1p31.1 | rs11588959 | *RPL31P12* | intergenic | 0.621 |
| 306 | Myopia-PUD | 1 | 196682346 | 1q313 | rs7535263 | *CFH* | intronic | 1.633 |
| 307 | Myopia-PUD | 2 | 211317 | 2p25.3 | rs62114494 | *SH3YL1* | intergenic | 1.208 |
| 308 | Myopia-PUD | 2 | 157397251 | 2q24.1 | rs3769361 | *GPD2* | intronic | 4.732 |
| 309 | Myopia-PUD | 3 | 141154542 | 3q23 | rs6440008 | *ZBTB38* | intronic | 4.287 |
| 310 | Myopia-PUD | 6 | 73521952 | 6q13 | rs9446760 | *KCNQ5* | intronic | 3.893 |
| 311 | Myopia-PUD | 8 | 60126579 | 8q12.1 | rs3110125 | *SNORA51* | intergenic | 10.21 |
| 312 | Myopia-PUD | 10 | 60260545 | 19q21.1 | rs7900904 | *BICC1* | intergenic | 1.928 |
| 313 | Myopia-PUD | 11 | 6273744 | 11p15.4 | rs10500661 | *CCKBR* | intergenic | 11.83 |
| 314 | Myopia-PUD | 14 | 54414132 | 14q22.2 | rs11623717 | *MIR5580* | intergenic | 4.321 |
| 315 | Myopia-PUD | 15 | 35005068 | 15q14 | rs649782 | *GJD2* | intergenic | 3.147 |
| 316 | Myopia-PUD | 18 | 72174023 | 18q22.3 | rs12971120 | *CNDP2* | intronic | 2.808 |
| 317 | Myopia-PUD | 19 | 49206674 | 19q13.33 | rs601338 | *FUT2* | exonic | 52 |
| 318 | Myopia-PUD | 20 | 6755170 | 20p12.3 | rs235766 | *BMP2* | intronic | 0.945 |
| 319 | Myopia-IBS | 1 | 197030087 | 1q31.3 | rs17514253 | *F13B* | exonic | 0.765 |
| 320 | Myopia-IBS | 3 | 153957088 | 3q25.2 | rs408419 | *ARHGEF26* | intronic | 2.873 |
| 321 | Myopia-IBS | 10 | 124091572 | 10q26.13 | rs3887220 | *BTBD16* | intronic | 2.006 |
| 322 | Myopia-IBS | 19 | 6730855 | 19p13.3 | rs2279623 | *GPR108* | intronic | 5 |
| 323 | Myopia-IBS | 22 | 39644273 | 22q13.1 | rs130651 | *PDGFB* | intergenic | 2.266 |
| 324 | Myopia-DD | 1 | 151991140 | 1q21.3 | rs1979637 | *NBPF18P:AL450992.6* | ncRNA_exonic | 3.905 |
| 325 | Myopia-DD | 1 | 200451422 | 1q32.1 | rs34049751 | *RP11-469A15.2* | intergenic | 0.821 |
| 326 | Myopia-DD | 1 | 207481316 | 1q32.2 | rs6698351 | *RP11-6J21.2* | ncRNA_intronic | 1.367 |
| 327 | Myopia-DD | 1 | 219704939 | 1q41 | rs76926608 | *RP11-95P13.2* | intergenic | 1.889 |
| 328 | Myopia-DD | 1 | 221081261 | 1q41 | rs2784272 | *HLX* | intergenic | 0.814 |
| 329 | Myopia-DD | 2 | 56079609 | 2p16.1 | rs11899380 | *EFEMP1* | intergenic | 5.502 |
| 330 | Myopia-DD | 2 | 144302171 | 2q22.3 | rs7597834 | *ARHGAP15:RP11-570L15.2:RP11-570L15.1* | ncRNA_intronic | 3.603 |
| 331 | Myopia-DD | 2 | 219991157 | 2q35 | rs72951773 | *NHEJ1:SLC23A3* | intronic | 0.185 |
| 332 | Myopia-DD | 3 | 16006898 | 3p25.1 | rs12637948 | *AC090945.1* | intergenic | 2.02 |
| 333 | Myopia-DD | 4 | 7917074 | 4p16.1 | rs62290573 | *AFAP1* | intronic | 2.65 |
| 334 | Myopia-DD | 4 | 80827799 | 4q21.21 | rs1139638 | *ANTXR2* | UTR3 | 7.998 |
| 335 | Myopia-DD | 5 | 64295363 | 5q12.3 | rs10471645 | *CWC27* | intronic | 0.091 |
| 336 | Myopia-DD | 6 | 129802971 | 6q22.33 | rs7775030 | *LAMA2* | intronic | 1.307 |
| 337 | Myopia-DD | 7 | 158852436 | 7q36.3 | rs62485830 | *VIPR2* | intronic | 2.675 |
| 338 | Myopia-DD | 8 | 122259446 | 8q24.12 | rs4870765 | *AC027238.1* | intergenic | 18.58 |
| 339 | Myopia-DD | 10 | 18440444 | 10p12.33 | rs1888693 | *CACNB2* | intronic | 4.448 |
| 340 | Myopia-DD | 10 | 25734059 | 10p12.1 | rs12764415 | *GPR158* | intronic | 4.153 |
| 341 | Myopia-DD | 10 | 60265656 | 10q21.1 | rs12262629 | *BICC1* | intergenic | 1.082 |
| 342 | Myopia-DD | 10 | 79124594 | 10q22.3 | rs11002137 | *KCNMA1:RP11-619F23.2* | ncRNA_intronic | 5.466 |
| 343 | Myopia-DD | 12 | 9313304 | 12p12.31 | rs7968679 | *PZP* | intronic | 0.678 |
| 344 | Myopia-DD | 15 | 35001752 | 15q14 | rs1915807 | *GJD2* | intergenic | 1.579 |
| 345 | Myopia-DD | 15 | 74226708 | 15q24.1 | rs2028386 | *LOXL1* | intronic | 6.252 |
| 346 | Myopia-DD | 17 | 7372637 | 17p13.1 | rs12942267 | *ZBTB4* | intronic | 0.116 |
| 347 | Myopia-DD | 17 | 31251098 | 17q11.2 | rs4605221 | *TMEM98* | intergenic | 0.054 |
| 348 | Myopia-DD | 21 | 47423509 | 21q22.3 | rs13051496 | *COL6A1* | exonic | 15.86 |
| 349 | Myopia-DD | 22 | 50697119 | 22q13.33 | rs742184 | *MAPK12* | intronic | 4.643 |
| 350 | PACG-GORD | 4 | 103198082 | 4q24 | rs13135092 | *SLC39A8* | intronic | 10.31 |
| 351 | PACG-GORD | 11 | 112861779 | 11q23.2 | rs4456284 | *NCAM1* | intronic | 1.188 |
| 352 | PACG-IBD | 1 | 20191436 | 1p36.13 | rs4654897 | *OTUD3* | intergenic | 6.298 |
| 353 | PACG-IBD | 7 | 128581835 | 7q32.1 | rs11761199 | *IRF5* | intronic | 5.68 |
| 354 | PACG-IBS | 6 | 17891547 | 6p22.3 | rs75982572 | *KIF13A* | intronic | 3.598 |
| 355 | PACG-IBS | 15 | 28380303 | 15q13.1 | rs6497279 | *HERC2* | intronic | 3.794 |
| 356 | PACG-DD | 1 | 234352899 | 1q42.2 | rs4333882 | *SLC35F3* | intronic | 3.843 |
| 357 | PACG-DD | 2 | 56095104 | 2p16.1 | rs11125608 | *EFEMP1* | intronic | 6.537 |
| 358 | PACG-DD | 2 | 144298063 | 2q22.3 | rs36168014 | *ARHGAP15:RP11-570L15.2:RP11-570L15.1* | ncRNA_intronic | 0.4 |
| 359 | PACG-DD | 3 | 151011326 | 3q25.1 | rs9810067 | *MED12L* | intronic | 8.767 |
| 360 | PACG-DD | 5 | 37721865 | 5p13.2 | rs1862577 | *WDR70* | intronic | 3.423 |
| 361 | PACG-DD | 5 | 111006695 | 5q22.1 | rs59011279 | *STARD4-AS1:NREP* | ncRNA_intronic | 6.347 |
| 362 | PACG-DD | 10 | 101360742 | 10q24.2 | rs112381543 | *snoU13* | upstream | 0.352 |
| 363 | PACG-DD | 11 | 27748493 | 11p14.1 | rs17309930 | *RP11-587D21.4* | ncRNA_intronic | 12.33 |
| 364 | PACG-DD | 15 | 28419085 | 15q13.1 | rs56743903 | *HERC2* | intronic | 0.981 |
| 365 | PACG-DD | 15 | 74225388 | 15q24.1 | rs4886778 | *LOXL1* | intronic | 3.116 |
| 366 | PACG-DD | 17 | 58959336 | 17q23.2 | rs138760546 | *BCAS3* | intronic | 3.195 |

**Table S5.** Three Chromosomal Regions Identified Across Eight Trait Pairs

| **No.** | **Region** | **Trait-pair** | **CHR** | **BP** | **rsID** | **nearestGene** | **Functional**  **annotation** |
| --- | --- | --- | --- | --- | --- | --- | --- |
| 1 | 1q32.1 | DR-IBD | 1 | 204426295 | rs1008833 | *PIK3C2B* | intronic |
| 1 | 1q32.1 | DR-IBD | 1 | 206943968 | rs3024493 | *IL10* | intronic |
| 1 | 1q32.1 | Uveitis-IBD | 1 | 200884985 | rs905634 | *C1orf106* | downstream |
| 1 | 1q32.1 | AMD-IBD | 1 | 200890353 | rs296544 | *MROH3P* | ncRNA_intronic |
| 1 | 1q32.1 | DED-IBD | 1 | 206955041 | rs3122605 | *IL10* | intergenic |
| 1 | 1q32.1 | Keratitis-IBD | 1 | 206943968 | rs3024493 | *IL10* | intronic |
| 1 | 1q32.1 | Myopia-GORD | 1 | 200366457 | rs6664603 | *ZNF281* | intergenic |
| 1 | 1q32.1 | Myopia-IBD | 1 | 200311674 | rs2790110 | *LINC00862* | ncRNA_exonic |
| 1 | 1q32.1 | Myopia-IBD | 1 | 201012597 | rs41267497 | *CACNA1S* | exonic |
| 1 | 1q32.1 | Myopia-DD | 1 | 200451422 | rs34049751 | *RP11-469A15.2* | intergenic |
| 2 | 2p16.1 | DR-GORD | 2 | 60649143 | rs12997266 | *BCL11A* | intergenic |
| 2 | 2p16.1 | DR-IBD | 2 | 59436587 | rs10181565 | *AC007131.1* | intergenic |
| 2 | 2p16.1 | DR-PUD | 2 | 58916786 | rs60635548 | *LINC01122* | ncRNA_intronic |
| 2 | 2p16.1 | DR-PUD | 2 | 60557705 | rs7589501 | *AC007381.3* | intergenic |
| 2 | 2p16.1 | DR-IBS | 2 | 58879493 | rs6724384 | *LINC01122* | ncRNA_intronic |
| 2 | 2p16.1 | DR-DD | 2 | 59319706 | rs7594743 | *LINC01122* | intergenic |
| 2 | 2p16.1 | DR-DD | 2 | 60579624 | rs12999941 | *AC007381.3* | ncRNA_intronic |
| 2 | 2p16.1 | Myopia-IBD | 2 | 56102744 | rs11899888 | *EFEMP1* | intronic |
| 2 | 2p16.1 | Myopia-DD | 2 | 56079609 | rs11899380 | *EFEMP1* | intergenic |
| 2 | 2p16.1 | PACG-DD | 2 | 56095104 | rs11125608 | *EFEMP1* | intronic |
| 3 | 2q22.3 | DR-DD | 2 | 144349201 | rs368217240 | *ARHGAP15-RP11* | ncRNA_intronic |
| 3 | 2q22.3 | Uveitis-DD | 2 | 144379627 | rs4146022 | *ARHGAP15* | intronic |
| 3 | 2q22.3 | Cataract-DD | 2 | 144425160 | rs138141174 | *ARHGAP15* | intronic |
| 3 | 2q22.3 | DED-IBS | 2 | 144391918 | rs28469251 | *ARHGAP15* | intronic |
| 3 | 2q22.3 | DED-DD | 2 | 144391918 | rs28469251 | *ARHGAP15* | intronic |
| 3 | 2q22.3 | Keratitis-DD | 2 | 144325926 | rs4372823 | *ARHGAP15:RP11-570L15.2:RP11-570L15.1* | ncRNA_intronic |
| 3 | 2q22.3 | Myopia-DD | 2 | 144302171 | rs7597834 | *ARHGAP15:RP11-570L15.2:RP11-570L15.1* | ncRNA_intronic |
| 3 | 2q22.3 | PACG-DD | 2 | 144298063 | rs36168014 | *ARHGAP15:RP11-570L15.2:RP11-570L15.1* | ncRNA_intronic |

**Table S6.** Effect Sizes and P Values of Top SNPs in Pleiotropic Loci From Original GWAS Summary Statistics

| **No.** | **Trait.pair** | **Region** | **Top.SNP** | **A1** | **A2** | **OREYE** | ***P*EYE** | **ORGUT** | ***P*GUT** |
| --- | --- | --- | --- | --- | --- | --- | --- | --- | --- |
| 1 | DR-GORD | 2p21 | rs1322 | G | A | 1.09E+00 | 1.81E-16 | 9.65E-01 | 2.90E-05 |
| 2 | DR-GORD | 2p16.1 | rs12997266 | G | A | 1.05E+00 | 1.44E-08 | 1.02E+00 | 1.40E-03 |
| 3 | DR-GORD | 2q36.3 | rs1522811 | C | A | 1.07E+00 | 3.26E-12 | 1.03E+00 | 8.30E-04 |
| 4 | DR-GORD | 3p21.31 | rs73077175 | G | A | 1.03E+00 | 1.38E-04 | 1.04E+00 | 2.70E-07 |
| 5 | DR-GORD | 3q27.2 | rs12636310 | A | G | 9.25E-01 | 2.90E-15 | 9.76E-01 | 1.50E-03 |
| 6 | DR-GORD | 4p16.1 | rs5028371 | G | C | 9.57E-01 | 8.62E-08 | 9.77E-01 | 2.50E-04 |
| 7 | DR-GORD | 4q24 | rs13107325 | C | T | 9.33E-01 | 3.26E-06 | 9.43E-01 | 9.30E-07 |
| 8 | DR-GORD | 6p21.1 | rs7765815 | A | T | 1.07E+00 | 4.69E-15 | 9.79E-01 | 3.70E-03 |
| 9 | DR-GORD | 10q22.3 | rs703994 | T | C | 1.05E+00 | 5.77E-08 | 9.79E-01 | 1.30E-03 |
| 10 | DR-GORD | 10q23.33 | rs11187145 | C | T | 1.10E+00 | 1.06E-15 | 1.02E+00 | 2.30E-02 |
| 11 | DR-GORD | 10q25.2 | rs11451675 | T | TG | 1.07E+00 | 6.79E-14 | 9.79E-01 | 1.40E-03 |
| 12 | DR-GORD | 11p15.4 | rs233448 | C | T | 1.07E+00 | 1.98E-14 | 9.84E-01 | 2.10E-02 |
| 13 | DR-GORD | 11q13.1 | rs12789028 | G | A | 9.31E-01 | 5.12E-13 | 9.70E-01 | 1.50E-04 |
| 14 | DR-GORD | 12q14.3 | rs1585897 | C | A | 9.49E-01 | 7.63E-10 | 9.82E-01 | 4.20E-03 |
| 15 | DR-GORD | 12q24.31 | rs10849888 | A | G | 9.55E-01 | 8.02E-08 | 1.03E+00 | 1.20E-05 |
| 16 | DR-GORD | 13q13.3 | rs9544488 | T | C | 1.04E+00 | 1.15E-05 | 1.03E+00 | 2.60E-06 |
| 17 | DR-GORD | 14q24.3 | rs4903516 | C | A | 1.05E+00 | 2.74E-06 | 1.03E+00 | 2.90E-04 |
| 18 | DR-GORD | 16q12.2 | rs12928335 | C | T | 1.10E+00 | 2.71E-10 | 9.69E-01 | 6.10E-03 |
| 19 | DR-GORD | 17q21.31 | rs2732686 | C | T | 9.54E-01 | 9.54E-07 | 1.04E+00 | 1.10E-05 |
| 20 | DR-GORD | 17q21.32 | rs11867603 | G | T | 1.06E+00 | 4.15E-11 | 1.02E+00 | 7.70E-04 |
| 21 | DR-GORD | 19p13.11 | rs7258722 | T | A | 1.04E+00 | 2.13E-07 | 1.03E+00 | 1.10E-04 |
| 22 | DR-GORD | 19p13.11 | rs111234557 | C | G | 9.18E-01 | 1.05E-11 | 9.62E-01 | 1.40E-04 |
| 23 | DR-GORD | 19q13.32 | rs429358 | T | C | 1.09E+00 | 2.55E-13 | 1.04E+00 | 3.50E-05 |
| 24 | DR-GORD | 20q11.22 | rs143220569 | G | A | 1.12E+00 | 3.23E-06 | 1.06E+00 | 2.40E-04 |
| 25 | DR-IBD | 1p31.3 | rs4385674 | C | T | 1.04E+00 | 6.85E-02 | 1.49E+00 | 1.40E-19 |
| 26 | DR-IBD | 1p31.1 | rs7531118 | T | C | 9.48E-01 | 9.72E-10 | 9.49E-01 | 2.50E-03 |
| 27 | DR-IBD | 1q32.1 | rs1008833 | A | G | 1.07E+00 | 9.29E-08 | 9.34E-01 | 4.10E-03 |
| 28 | DR-IBD | 1q32.1 | rs3024493 | C | A | 1.03E+00 | 1.70E-02 | 8.42E-01 | 9.10E-15 |
| 29 | DR-IBD | 2p21 | rs78487399 | G | C | 1.12E+00 | 1.36E-15 | 8.77E-01 | 1.40E-06 |
| 30 | DR-IBD | 2p16.1 | rs10181565 | G | A | 1.05E+00 | 1.07E-06 | 1.07E+00 | 7.90E-04 |
| 31 | DR-IBD | 2q31.1 | rs2140046 | T | C | 9.50E-01 | 9.76E-09 | 9.49E-01 | 3.00E-03 |
| 32 | DR-IBD | 3q27.2 | rs11708719 | A | G | 9.02E-01 | 6.79E-09 | 9.16E-01 | 7.70E-03 |
| 33 | DR-IBD | 4q24 | rs1813006 | G | T | 9.21E-01 | 1.17E-06 | 8.91E-01 | 1.60E-03 |
| 34 | DR-IBD | 6p24.3 | rs1815311 | A | G | 9.35E-01 | 2.50E-14 | 1.05E+00 | 4.60E-03 |
| 35 | DR-IBD | 6p22.3 | rs6456366 | G | C | 9.07E-01 | 1.90E-16 | 9.41E-01 | 1.50E-02 |
| 36 | DR-IBD | 6p22.1 | rs1233370 | C | A | 1.07E+00 | 2.01E-05 | 9.10E-01 | 2.00E-06 |
| 37 | DR-IBD | 6p22.1 | rs11759908 | C | T | 1.04E+00 | 9.21E-07 | 9.32E-01 | 5.10E-05 |
| 38 | DR-IBD | 6p22.1 | rs57266556 | C | T | 9.42E-01 | 8.17E-07 | 9.02E-01 | 1.60E-05 |
| 39 | DR-IBD | 7q36.3 | rs3802123 | G | A | 1.07E+00 | 1.95E-14 | 9.63E-01 | 3.60E-02 |
| 40 | DR-IBD | 8p11.21 | rs12549902 | G | A | 9.41E-01 | 2.03E-14 | 9.63E-01 | 2.90E-02 |
| 41 | DR-IBD | 8q23.3 | rs3808434 | A | G | 1.07E+00 | 1.84E-16 | 9.59E-01 | 1.40E-02 |
| 42 | DR-IBD | 9q34.3 | rs11145912 | G | A | 1.04E+00 | 1.47E-06 | 9.21E-01 | 1.60E-06 |
| 43 | DR-IBD | 10q23.33 | rs4646955 | A | G | 9.34E-01 | 3.41E-14 | 1.04E+00 | 2.70E-02 |
| 44 | DR-IBD | 10q25.2 | rs4918784 | A | G | 9.38E-01 | 2.90E-10 | 1.06E+00 | 2.80E-03 |
| 45 | DR-IBD | 11q14.3 | rs12789669 | C | T | 9.65E-01 | 3.04E-04 | 9.20E-01 | 6.40E-06 |
| 46 | DR-IBD | 15q24.3 | rs12909924 | T | G | 9.45E-01 | 4.97E-09 | 1.06E+00 | 4.60E-03 |
| 47 | DR-IBD | 20q12 | rs74577821 | A | T | 9.37E-01 | 1.56E-09 | 9.34E-01 | 2.00E-03 |
| 48 | DR-PUD | 1q25.3 | rs12146099 | G | A | 9.45E-01 | 7.09E-11 | 1.05E+00 | 9.20E-05 |
| 49 | DR-PUD | 1q32.3 | rs61320678 | T | G | 1.06E+00 | 9.46E-09 | 9.63E-01 | 4.50E-03 |
| 50 | DR-PUD | 2p23.3 | rs780093 | T | C | 9.42E-01 | 5.53E-13 | 1.03E+00 | 7.40E-03 |
| 51 | DR-PUD | 2p21 | rs13029250 | G | T | 1.05E+00 | 2.41E-09 | 9.59E-01 | 1.80E-04 |
| 52 | DR-PUD | 2p16.1 | rs60635548 | A | G | 1.05E+00 | 8.82E-08 | 9.65E-01 | 1.90E-03 |
| 53 | DR-PUD | 2p16.1 | rs7589501 | A | G | 1.04E+00 | 3.84E-06 | 1.05E+00 | 1.00E-04 |
| 54 | DR-PUD | 2q24.3 | rs17184707 | C | T | 9.57E-01 | 3.00E-05 | 9.51E-01 | 1.90E-04 |
| 55 | DR-PUD | 3p25.2 | rs2120825 | T | G | 1.11E+00 | 4.88E-14 | 1.05E+00 | 4.30E-03 |
| 56 | DR-PUD | 3p24.3 | rs13094957 | T | C | 1.08E+00 | 1.74E-12 | 9.70E-01 | 2.30E-02 |
| 57 | DR-PUD | 4p16.1 | rs6833959 | G | A | 9.37E-01 | 3.82E-10 | 9.63E-01 | 5.10E-03 |
| 58 | DR-PUD | 4q31.22 | rs13140054 | G | A | 9.63E-01 | 1.69E-06 | 1.04E+00 | 1.00E-03 |
| 59 | DR-PUD | 5q13.3 | rs7732628 | G | A | 1.04E+00 | 1.29E-05 | 1.06E+00 | 8.40E-07 |
| 60 | DR-PUD | 5q21.1 | rs79602013 | T | C | 8.63E-01 | 3.81E-15 | 1.06E+00 | 2.90E-02 |
| 61 | DR-PUD | 5q33.3 | rs115498181 | T | C | 8.74E-01 | 1.01E-06 | 8.42E-01 | 2.20E-07 |
| 62 | DR-PUD | 6p22.3 | rs6905138 | A | G | 9.19E-01 | 1.67E-11 | 1.05E+00 | 1.00E-02 |
| 63 | DR-PUD | 7p15.1 | rs1635851 | C | T | 1.07E+00 | 5.25E-15 | 1.03E+00 | 1.60E-02 |
| 64 | DR-PUD | 7q36.3 | rs1182442 | G | A | 9.37E-01 | 1.29E-14 | 9.65E-01 | 2.60E-03 |
| 65 | DR-PUD | 9p21.3 | rs12379111 | C | G | 1.13E+00 | 2.07E-13 | 1.04E+00 | 3.20E-02 |
| 66 | DR-PUD | 9q34.2 | rs8176719 | T | TC | 9.53E-01 | 7.58E-09 | 1.07E+00 | 8.60E-08 |
| 67 | DR-PUD | 10q21.1 | rs11592488 | A | T | 9.66E-01 | 7.05E-05 | 9.48E-01 | 1.00E-05 |
| 68 | DR-PUD | 10q22.3 | rs703965 | C | T | 9.43E-01 | 8.23E-14 | 1.02E+00 | 3.90E-02 |
| 69 | DR-PUD | 11p15.4 | rs45477500 | G | A | 8.80E-01 | 3.16E-06 | 8.63E-01 | 6.10E-04 |
| 70 | DR-PUD | 12p13.32 | rs117233107 | G | A | 1.32E+00 | 4.21E-13 | 1.13E+00 | 2.80E-02 |
| 71 | DR-PUD | 17q12 | rs34473775 | G | T | 1.05E+00 | 2.26E-07 | 9.62E-01 | 2.50E-03 |
| 72 | DR-PUD | 18q21.2 | rs624244 | G | A | 9.54E-01 | 4.35E-08 | 9.66E-01 | 3.70E-03 |
| 73 | DR-PUD | 19q13.32 | rs2341097 | C | T | 9.52E-01 | 5.96E-09 | 9.69E-01 | 7.10E-03 |
| 74 | DR-PUD | 19q13.33 | rs601338 | G | A | 1.03E+00 | 2.73E-04 | 9.15E-01 | 2.40E-15 |
| 75 | DR-PUD | 21q21.1 | rs60893125 | G | A | 1.04E+00 | 7.49E-04 | 9.38E-01 | 2.60E-06 |
| 76 | DR-IBS | 1p34.3 | rs61779310 | C | G | 9.26E-01 | 7.32E-13 | 9.71E-01 | 2.72E-04 |
| 77 | DR-IBS | 2p23.3 | rs1260326 | T | C | 9.40E-01 | 1.43E-13 | 1.02E+00 | 6.30E-04 |
| 78 | DR-IBS | 2p16.1 | rs6724384 | C | T | 1.05E+00 | 8.19E-08 | 1.02E+00 | 2.16E-03 |
| 79 | DR-IBS | 2q36.3 | rs13012754 | C | G | 9.29E-01 | 4.62E-16 | 9.74E-01 | 1.97E-03 |
| 80 | DR-IBS | 3p24.3 | rs9848331 | G | A | 1.05E+00 | 7.27E-07 | 1.03E+00 | 1.48E-04 |
| 81 | DR-IBS | 3p12.1 | rs4334651 | G | T | 1.04E+00 | 5.47E-06 | 9.66E-01 | 6.24E-05 |
| 82 | DR-IBS | 3q22.3 | rs10048942 | A | G | 9.52E-01 | 1.40E-07 | 9.72E-01 | 4.25E-04 |
| 83 | DR-IBS | 3q27.1 | rs9869577 | C | T | 1.05E+00 | 1.88E-09 | 1.02E+00 | 9.88E-04 |
| 84 | DR-IBS | 3q27.3 | rs9860157 | G | A | 9.51E-01 | 1.82E-08 | 9.77E-01 | 1.85E-03 |
| 85 | DR-IBS | 5q21.1 | rs13188193 | C | T | 8.60E-01 | 8.71E-16 | 9.60E-01 | 8.53E-03 |
| 86 | DR-IBS | 6p24.3 | rs9502564 | G | T | 9.62E-01 | 1.47E-05 | 1.03E+00 | 1.08E-05 |
| 87 | DR-IBS | 6p21.1 | rs4365924 | T | C | 1.05E+00 | 3.64E-07 | 9.68E-01 | 2.46E-04 |
| 88 | DR-IBS | 8p23.1 | rs2409729 | C | G | 1.04E+00 | 2.04E-04 | 9.62E-01 | 3.31E-06 |
| 89 | DR-IBS | 8q23.3 | rs2049865 | C | A | 1.07E+00 | 5.32E-16 | 1.02E+00 | 7.45E-03 |
| 90 | DR-IBS | 9p21.3 | rs78432974 | C | T | 1.14E+00 | 1.47E-12 | 9.56E-01 | 6.65E-03 |
| 91 | DR-IBS | 9q33.3 | rs4838083 | G | T | 9.41E-01 | 4.64E-10 | 1.02E+00 | 1.65E-03 |
| 92 | DR-IBS | 10q11.23 | rs200803583 | A | C | 9.08E-01 | 2.75E-06 | 9.34E-01 | 3.19E-04 |
| 93 | DR-IBS | 10q22.3 | rs2802362 | A | G | 9.36E-01 | 1.66E-15 | 1.02E+00 | 1.34E-02 |
| 94 | DR-IBS | 11p15.1 | rs1002226 | C | T | 1.07E+00 | 3.13E-15 | 1.02E+00 | 2.88E-02 |
| 95 | DR-IBS | 12q14.3 | rs1351394 | T | C | 9.40E-01 | 5.96E-15 | 9.78E-01 | 8.90E-04 |
| 96 | DR-IBS | 15q26.1 | rs4932148 | C | T | 1.08E+00 | 1.18E-16 | 1.02E+00 | 4.05E-03 |
| 97 | DR-IBS | 15q26.1 | rs28404354 | C | T | 9.53E-01 | 3.53E-09 | 1.03E+00 | 1.95E-04 |
| 98 | DR-IBS | 17q21.31 | rs111274735 | T | A | 9.56E-01 | 6.62E-07 | 1.08E+00 | 1.42E-05 |
| 99 | DR-IBS | 17q21.32 | rs28627277 | G | T | 1.06E+00 | 2.05E-10 | 9.77E-01 | 8.52E-03 |
| 100 | DR-IBS | 20q11.22 | rs60012358 | A | C | 9.41E-01 | 3.83E-05 | 9.49E-01 | 6.83E-05 |
| 101 | DR-IBS | 22q12.2 | rs41172 | T | C | 1.04E+00 | 1.86E-06 | 1.03E+00 | 4.49E-05 |
| 102 | DR-DD | 2p21 | rs7560838 | C | A | 1.11E+00 | 1.44E-13 | 1.00E+00 | 6.90E-05 |
| 103 | DR-DD | 2p16.1 | rs7594743 | C | T | 1.06E+00 | 1.96E-10 | 1.00E+00 | 4.80E-03 |
| 104 | DR-DD | 2p16.1 | rs12999941 | C | T | 1.05E+00 | 3.08E-09 | 9.98E-01 | 2.00E-03 |
| 105 | DR-DD | 2q22.3 | rs368217240 | T | TC | 1.02E+00 | 1.15E-01 | 1.01E+00 | 7.50E-32 |
| 106 | DR-DD | 3p25.2 | rs2120825 | T | G | 1.11E+00 | 4.88E-14 | 1.00E+00 | 3.70E-03 |
| 107 | DR-DD | 3q22.3 | rs4678411 | G | A | 1.04E+00 | 6.21E-07 | 1.00E+00 | 3.00E-04 |
| 108 | DR-DD | 4q31.21 | rs11727676 | T | C | 9.23E-01 | 2.70E-09 | 1.00E+00 | 7.00E-07 |
| 109 | DR-DD | 4q31.3 | rs2305980 | T | A | 9.62E-01 | 3.97E-05 | 9.97E-01 | 1.50E-05 |
| 110 | DR-DD | 5p13.2 | rs2973068 | G | C | 9.68E-01 | 4.33E-04 | 9.96E-01 | 8.00E-10 |
| 111 | DR-DD | 5q21.1 | rs13188193 | C | T | 8.60E-01 | 8.71E-16 | 1.00E+00 | 4.80E-03 |
| 112 | DR-DD | 6p24.3 | rs9379084 | G | A | 1.08E+00 | 5.12E-10 | 1.00E+00 | 2.80E-03 |
| 113 | DR-DD | 6p22.3 | rs201464451 | C | T | 9.38E-01 | 1.19E-14 | 1.00E+00 | 7.70E-04 |
| 114 | DR-DD | 6p21.1 | rs1563788 | C | T | 9.65E-01 | 4.49E-05 | 9.97E-01 | 2.50E-05 |
| 115 | DR-DD | 6q16.1 | rs5878501 | G | GT | 9.68E-01 | 1.15E-03 | 9.96E-01 | 2.90E-10 |
| 116 | DR-DD | 7p21.2 | rs4719430 | G | C | 1.05E+00 | 6.86E-08 | 1.00E+00 | 9.30E-05 |
| 117 | DR-DD | 7p15.1 | rs11448038 | T | TA | 1.07E+00 | 1.57E-15 | 9.98E-01 | 1.70E-04 |
| 118 | DR-DD | 7q22.1 | rs7794717 | C | T | 1.03E+00 | 3.39E-03 | 9.96E-01 | 2.50E-14 |
| 119 | DR-DD | 8q23.3 | rs2049865 | C | A | 1.07E+00 | 5.32E-16 | 9.97E-01 | 4.90E-08 |
| 120 | DR-DD | 8q24.12 | rs1553506 | G | T | 1.03E+00 | 5.02E-04 | 9.96E-01 | 1.20E-09 |
| 121 | DR-DD | 9q33.3 | rs12236873 | A | G | 9.46E-01 | 5.25E-08 | 9.98E-01 | 1.70E-04 |
| 122 | DR-DD | 9q34.2 | rs8176719 | T | TC | 9.53E-01 | 7.58E-09 | 1.00E+00 | 7.30E-15 |
| 123 | DR-DD | 10p13 | rs11257658 | G | A | 9.15E-01 | 1.70E-15 | 1.00E+00 | 2.30E-03 |
| 124 | DR-DD | 10q24.33 | rs3752946 | A | T | 9.65E-01 | 4.44E-06 | 1.00E+00 | 1.80E-08 |
| 125 | DR-DD | 10q25.2 | rs11599737 | G | A | 1.10E+00 | 3.96E-10 | 1.00E+00 | 1.80E-03 |
| 126 | DR-DD | 11p15.5 | rs2334418 | C | G | 9.53E-01 | 6.09E-09 | 1.00E+00 | 4.70E-04 |
| 127 | DR-DD | 11p15.4 | rs234859 | T | C | 1.07E+00 | 7.33E-14 | 9.98E-01 | 1.70E-03 |
| 128 | DR-DD | 11p14.1 | rs2353487 | C | T | 1.02E+00 | 7.65E-03 | 9.96E-01 | 1.20E-13 |
| 129 | DR-DD | 11p11.2 | rs7107792 | A | C | 1.04E+00 | 3.75E-08 | 1.00E+00 | 4.20E-04 |
| 130 | DR-DD | 11q13.1 | rs583887 | T | C | 1.06E+00 | 7.19E-08 | 1.00E+00 | 7.50E-05 |
| 131 | DR-DD | 12q24.31 | rs11408808 | T | TA | 1.05E+00 | 3.50E-07 | 1.00E+00 | 5.70E-04 |
| 132 | DR-DD | 12q24.31 | rs889970 | G | A | 9.52E-01 | 4.02E-08 | 1.00E+00 | 1.40E-03 |
| 133 | DR-DD | 15q15.1 | rs7164606 | G | A | 1.03E+00 | 6.45E-03 | 9.95E-01 | 1.80E-10 |
| 134 | DR-DD | 15q23 | rs71400388 | A | C | 9.59E-01 | 4.83E-05 | 1.00E+00 | 3.00E-05 |
| 135 | DR-DD | 16q23.1 | rs72804106 | G | T | 1.09E+00 | 4.68E-10 | 9.97E-01 | 2.80E-04 |
| 136 | DR-DD | 18q21.2 | rs624244 | G | A | 9.54E-01 | 4.35E-08 | 9.98E-01 | 5.40E-05 |
| 137 | DR-DD | 18q21.32 | rs7240682 | C | G | 9.37E-01 | 7.75E-13 | 1.00E+00 | 1.10E-02 |
| 138 | DR-DD | 20q12 | rs17265513 | T | C | 9.40E-01 | 2.16E-10 | 1.00E+00 | 2.70E-03 |
| 139 | DR-DD | 21q22.3 | rs9976088 | T | G | 1.04E+00 | 5.02E-05 | 1.01E+00 | 6.50E-01 |
| 140 | Uveitis-IBD | 1p31.3 | rs11209026 | G | A | 1.16E+00 | 9.46E-04 | 1.51E+00 | 2.40E-24 |
| 141 | Uveitis-IBD | 1q32.1 | rs905634 | C | T | 1.06E+00 | 1.59E-02 | 1.14E+00 | 7.80E-13 |
| 142 | Uveitis-IBD | 21q22.2 | rs2836878 | G | A | 1.05E+00 | 3.29E-02 | 1.21E+00 | 2.10E-21 |
| 143 | Uveitis-PUD | 2p22.1 | rs732707 | C | T | 8.92E-01 | 8.59E-05 | 1.06E+00 | 1.30E-05 |
| 144 | Uveitis-PUD | 19q13.33 | rs368565 | C | T | 1.07E+00 | 2.02E-03 | 1.08E+00 | 9.30E-11 |
| 145 | Uveitis-IBS | 4q35.2 | rs72718909 | T | C | 8.71E-01 | 8.52E-06 | 1.04E+00 | 1.97E-04 |
| 146 | Uveitis-DD | 2q22.3 | rs4146022 | G | A | 9.28E-01 | 2.04E-02 | 9.93E-01 | 3.30E-22 |
| 147 | AMD-GORD | 1q32.2 | rs7523273 | A | G | 1.05E+00 | 4.41E-11 | 9.82E-01 | 6.90E-03 |
| 148 | AMD-GORD | 6p22.2 | rs62396201 | G | A | 9.72E-01 | 1.03E-05 | 9.68E-01 | 4.40E-07 |
| 149 | AMD-GORD | 6p22.1 | rs17720293 | C | T | 1.04E+00 | 3.25E-03 | 1.06E+00 | 5.80E-09 |
| 150 | AMD-GORD | 10q26.13 | rs111266031 | C | T | 1.10E+00 | 1.83E-14 | 1.03E+00 | 2.80E-03 |
| 151 | AMD-GORD | 19q13.32 | rs429358 | T | C | 1.05E+00 | 2.62E-08 | 1.04E+00 | 3.50E-05 |
| 152 | AMD-IBD | 1p31.3 | rs1343151 | G | A | 1.02E+00 | 2.16E-03 | 1.18E+00 | 1.20E-19 |
| 153 | AMD-IBD | 1q31.3 | rs12130962 | A | C | 1.10E+00 | 6.32E-06 | 8.30E-01 | 1.20E-05 |
| 154 | AMD-IBD | 1q31.3 | rs188357630 | G | T | 1.25E+00 | 1.44E-15 | 8.58E-01 | 2.70E-02 |
| 155 | AMD-IBD | 1q31.3 | rs12144001 | G | C | 1.09E+00 | 2.33E-08 | 8.92E-01 | 3.10E-03 |
| 156 | AMD-IBD | 1q32.1 | rs296544 | G | T | 9.74E-01 | 2.99E-05 | 8.98E-01 | 3.40E-10 |
| 157 | AMD-IBD | 1q32.2 | rs2745979 | G | C | 1.05E+00 | 2.35E-13 | 1.06E+00 | 2.70E-03 |
| 158 | AMD-IBD | 6p22.1 | rs1233372 | A | G | 1.03E+00 | 3.79E-04 | 1.09E+00 | 7.20E-07 |
| 159 | AMD-IBD | 7q32.1 | rs3757387 | T | C | 9.76E-01 | 1.62E-04 | 9.11E-01 | 4.50E-08 |
| 160 | AMD-IBD | 7q36.1 | rs243546 | T | C | 9.67E-01 | 9.05E-06 | 1.07E+00 | 7.10E-05 |
| 161 | AMD-IBD | 10q26.13 | rs78200813 | C | G | 9.02E-01 | 9.35E-16 | 1.08E+00 | 2.80E-02 |
| 162 | AMD-IBD | 15q24.1 | rs12903896 | C | T | 9.56E-01 | 7.23E-12 | 9.22E-01 | 9.30E-06 |
| 163 | AMD-IBD | 20q13.33 | rs6011033 | A | G | 9.71E-01 | 1.18E-04 | 9.06E-01 | 2.00E-06 |
| 164 | AMD-IBD | 21q22.2 | rs2836884 | T | C | 1.02E+00 | 4.25E-02 | 1.20E+00 | 2.10E-20 |
| 165 | AMD-IBD | 22q13.1 | rs4820371 | T | C | 1.06E+00 | 6.45E-12 | 1.08E+00 | 2.00E-04 |
| 166 | AMD-PUD | 1q31.3 | rs11585965 | C | A | 1.08E+00 | 2.10E-16 | 9.51E-01 | 1.30E-03 |
| 167 | AMD-PUD | 4q21.3 | rs17449582 | C | T | 1.03E+00 | 1.89E-06 | 1.04E+00 | 2.70E-04 |
| 168 | AMD-PUD | 5q13.3 | rs7707527 | C | G | 1.02E+00 | 5.83E-04 | 1.06E+00 | 1.20E-06 |
| 169 | AMD-PUD | 9p21.3 | rs10965246 | T | C | 1.05E+00 | 1.92E-08 | 1.04E+00 | 4.90E-03 |
| 170 | AMD-PUD | 10q26.13 | rs72826311 | G | A | 8.72E-01 | 2.01E-10 | 8.84E-01 | 1.20E-04 |
| 171 | AMD-PUD | 16q24.1 | rs370509910 | G | T | 9.22E-01 | 2.10E-10 | 9.43E-01 | 1.20E-02 |
| 172 | AMD-PUD | 19q13.33 | rs2287922 | G | A | 9.67E-01 | 1.11E-07 | 9.28E-01 | 2.90E-11 |
| 173 | AMD-IBS | 1q31.3 | rs17514253 | C | T | 7.85E-01 | 5.65E-16 | 1.61E+00 | 1.70E-02 |
| 174 | AMD-IBS | 3q25.5 | rs408419 | A | T | 1.07E+00 | 5.08E-12 | 9.72E-01 | 1.13E-02 |
| 175 | AMD-IBS | 10q26.13 | rs3887220 | C | T | 9.48E-01 | 2.98E-16 | 9.80E-01 | 3.03E-03 |
| 176 | AMD-IBS | 19p13.3 | rs2279623 | A | C | 1.05E+00 | 8.91E-12 | 1.02E+00 | 8.70E-03 |
| 177 | AMD-IBS | 22q13.1 | rs130651 | G | A | 9.46E-01 | 8.29E-16 | 9.80E-01 | 8.80E-03 |
| 178 | AMD-DD | 1p22.2 | rs112500140 | A | G | 1.02E+00 | 5.91E-01 | 1.00E+00 | 1.40E-01 |
| 179 | AMD-DD | 2q24.3 | rs115488970 | A | G | 9.58E-01 | 1.67E-01 | 1.00E+00 | 2.20E-01 |
| 180 | AMD-DD | 8q24.3 | rs2447389 | G | A | 9.82E-01 | 9.53E-03 | 1.00E+00 | 4.30E-01 |
| 181 | AMD-DD | 9p24.3 | rs1629857 | T | G | 9.60E-01 | 2.85E-01 | 9.99E-01 | 5.70E-01 |
| 182 | AMD-DD | 9p24.3 | rs583230 | T | C | 1.00E+00 | 7.48E-01 | 1.00E+00 | 2.60E-01 |
| 183 | AMD-DD | 9p24.2 | rs16919460 | G | T | 9.90E-01 | 5.93E-01 | 1.00E+00 | 2.10E-01 |
| 184 | AMD-DD | 9p24.1 | rs296859 | C | T | 1.01E+00 | 4.13E-01 | 9.99E-01 | 2.00E-01 |
| 185 | AMD-DD | 9p24.1 | rs2578273 | G | A | 1.01E+00 | 3.34E-01 | 1.00E+00 | 2.60E-01 |
| 186 | AMD-DD | 9p24.1 | rs76752978 | T | C | 9.65E-01 | 5.55E-02 | 9.98E-01 | 2.50E-01 |
| 187 | AMD-DD | 9p23 | rs1928727 | G | T | 1.01E+00 | 3.72E-02 | 1.00E+00 | 7.80E-01 |
| 188 | AMD-DD | 9p21.2 | rs2171101 | C | T | 1.01E+00 | 1.36E-01 | 1.00E+00 | 3.80E-01 |
| 189 | AMD-DD | 11p15.5 | rs67912009 | G | T | 1.01E+00 | 3.69E-01 | 1.00E+00 | 1.10E-01 |
| 190 | AMD-DD | 11p15.5 | rs10466744 | G | A | 1.01E+00 | 3.32E-01 | 1.00E+00 | 5.30E-03 |
| 191 | AMD-DD | 11p14.3 | rs76747679 | C | T | 9.66E-01 | 2.07E-01 | 9.98E-01 | 3.10E-01 |
| 192 | AMD-DD | 13q14.11 | rs12430400 | G | A | 1.01E+00 | 7.06E-02 | 1.00E+00 | 6.90E-01 |
| 193 | AMD-DD | 16q22.1 | rs113573226 | C | T | 9.31E-01 | 2.10E-03 | 9.98E-01 | 2.80E-01 |
| 194 | AMD-DD | 18p11.32 | rs2847613 | T | C | 9.87E-01 | 1.13E-01 | 1.00E+00 | 2.80E-01 |
| 195 | AMD-DD | 18p11.31 | rs1546707 | C | T | 1.01E+00 | 5.08E-01 | 1.00E+00 | 5.60E-01 |
| 196 | AMD-DD | 18q11.2 | rs76748134 | C | T | 9.86E-01 | 5.48E-01 | 1.00E+00 | 9.60E-01 |
| 197 | AMD-DD | 19p13.3 | rs150394100 | G | A | 1.04E+00 | 1.38E-01 | 1.00E+00 | 5.10E-01 |
| 198 | AMD-DD | 19p13.3 | rs150383413 | C | G | 1.02E+00 | 4.27E-01 | 1.00E+00 | 5.80E-01 |
| 199 | AMD-DD | 19p13.11 | rs34937778 | G | A | 1.00E+00 | 9.57E-01 | 1.00E+00 | 7.40E-01 |
| 200 | Cataract-GORD | 3p21.31 | rs1858828 | G | T | 9.82E-01 | 9.10E-05 | 9.68E-01 | 2.90E-07 |
| 201 | Cataract-GORD | 4p12 | rs13104400 | T | A | 9.77E-01 | 2.41E-07 | 9.79E-01 | 1.10E-03 |
| 202 | Cataract-GORD | 6p22.2 | rs2024970 | T | A | 9.85E-01 | 7.76E-04 | 9.65E-01 | 2.30E-08 |
| 203 | Cataract-GORD | 15q25.1 | rs12437863 | C | T | 1.03E+00 | 1.19E-05 | 1.04E+00 | 5.20E-05 |
| 204 | Cataract-GORD | 19p13.11 | rs12973258 | T | C | 9.69E-01 | 9.02E-08 | 9.67E-01 | 4.20E-05 |
| 205 | Cataract-IBD | 1p36.13 | rs3820330 | C | A | 1.02E+00 | 3.87E-03 | 1.12E+00 | 5.50E-09 |
| 206 | Cataract-IBD | 1p31.3 | rs41396545 | A | G | 1.01E+00 | 3.67E-02 | 1.17E+00 | 9.90E-17 |
| 207 | Cataract-IBD | 1q23.3 | rs1801274 | A | G | 1.01E+00 | 1.74E-02 | 1.13E+00 | 2.70E-12 |
| 208 | Cataract-IBD | 3p23.31 | rs9862080 | A | G | 1.02E+00 | 1.35E-03 | 9.11E-01 | 2.80E-07 |
| 209 | Cataract-IBD | 5p13.1 | rs2084031 | T | C | 9.84E-01 | 6.46E-04 | 9.01E-01 | 3.00E-09 |
| 210 | Cataract-IBD | 7q31.1 | rs6964893 | G | T | 1.02E+00 | 2.40E-03 | 1.11E+00 | 5.40E-09 |
| 211 | Cataract-PUD | 17q21.2 | rs34074411 | C | T | 9.88E-01 | 9.86E-03 | 9.30E-01 | 2.60E-10 |
| 212 | Cataract-PUD | 19q13.41 | rs1710353 | A | G | 9.74E-01 | 5.87E-08 | 9.67E-01 | 3.80E-03 |
| 213 | Cataract-IBS | 1p34.3 | rs41267043 | G | A | 9.70E-01 | 5.40E-06 | 1.04E+00 | 3.41E-04 |
| 214 | Cataract-IBS | 3q26.33 | rs7611110 | A | C | 9.79E-01 | 3.12E-05 | 9.71E-01 | 7.57E-05 |
| 215 | Cataract-IBS | 4p16.2 | rs7670828 | G | A | 1.02E+00 | 5.23E-05 | 9.65E-01 | 1.11E-05 |
| 216 | Cataract-IBS | 4q25 | rs147098535 | C | T | 1.03E+00 | 3.00E-05 | 9.56E-01 | 1.54E-05 |
| 217 | Cataract-IBS | 4q32.1 | rs11100094 | A | G | 1.02E+00 | 2.22E-04 | 1.04E+00 | 9.38E-06 |
| 218 | Cataract-IBS | 9q31.2 | rs2818292 | C | A | 1.03E+00 | 1.07E-05 | 9.69E-01 | 1.04E-04 |
| 219 | Cataract-IBS | 11q23.2 | rs1940725 | T | C | 9.85E-01 | 1.88E-03 | 9.63E-01 | 7.17E-08 |
| 220 | Cataract-IBS | 11q23.2 | rs4319542 | C | G | 1.03E+00 | 2.08E-08 | 1.02E+00 | 2.07E-03 |
| 221 | Cataract-DD | 1q42.2 | rs4333882 | A | G | 9.85E-01 | 9.79E-03 | 9.93E-01 | 2.50E-24 |
| 222 | Cataract-DD | 2q22.3 | rs138141174 | A | T | 1.02E+00 | 4.07E-04 | 1.01E+00 | 9.20E-23 |
| 223 | Cataract-DD | 6q16.1 | rs4339469 | T | G | 9.84E-01 | 4.35E-04 | 9.96E-01 | 1.70E-11 |
| 224 | Cataract-DD | 7q11.23 | rs2528794 | G | A | 9.75E-01 | 9.54E-04 | 1.00E+00 | 6.10E-08 |
| 225 | Cataract-DD | 9q34.2 | rs8176719 | T | TC | 9.89E-01 | 2.58E-02 | 1.00E+00 | 7.30E-15 |
| 226 | Cataract-DD | 10p12.1 | rs12268257 | C | T | 1.02E+00 | 5.50E-03 | 1.01E+00 | 4.50E-11 |
| 227 | Cataract-DD | 10q24.33 | rs10748858 | T | G | 1.02E+00 | 3.58E-06 | 9.97E-01 | 6.40E-08 |
| 228 | Cataract-DD | 11p14.1 | rs2353487 | C | T | 1.01E+00 | 1.58E-02 | 9.96E-01 | 1.20E-13 |
| 229 | DED-GORD | 4q24 | rs13107325 | C | T | 9.50E-01 | 1.77E-07 | 9.43E-01 | 9.30E-07 |
| 230 | DED-GORD | 6p22.2 | rs7755997 | G | A | 9.80E-01 | 2.49E-04 | 9.55E-01 | 1.30E-12 |
| 231 | DED-GORD | 6p21.32 | rs79984539 | A | G | 9.38E-01 | 1.49E-07 | 9.55E-01 | 1.10E-03 |
| 232 | DED-GORD | 11q23.2 | rs7942723 | T | G | 9.76E-01 | 1.27E-05 | 9.67E-01 | 2.40E-07 |
| 233 | DED-GORD | 19p13.11 | rs12462498 | C | T | 1.03E+00 | 1.01E-04 | 1.04E+00 | 5.10E-07 |
| 234 | DED-IBD | 1p36.13 | rs72658556 | T | A | 1.02E+00 | 3.92E-03 | 1.15E+00 | 1.60E-12 |
| 235 | DED-IBD | 1q23.3 | rs4657040 | T | C | 9.83E-01 | 2.19E-03 | 1.11E+00 | 2.20E-07 |
| 236 | DED-IBD | 1q32.1 | rs3122605 | G | A | 1.02E+00 | 7.87E-03 | 1.17E+00 | 3.70E-11 |
| 237 | DED-IBD | 3p21.31 | rs3197999 | G | A | 1.02E+00 | 1.60E-03 | 9.05E-01 | 5.70E-08 |
| 238 | DED-IBD | 4q24 | rs6855246 | A | G | 9.51E-01 | 4.02E-07 | 8.97E-01 | 6.70E-04 |
| 239 | DED-IBD | 5q31.1 | rs11749300 | T | C | 1.02E+00 | 8.67E-04 | 9.23E-01 | 2.40E-06 |
| 240 | DED-IBD | 7q31.1 | rs2237690 | C | T | 1.02E+00 | 5.83E-04 | 1.09E+00 | 4.30E-07 |
| 241 | DED-IBD | 7q32.1 | rs3823536 | G | A | 9.84E-01 | 2.60E-03 | 9.12E-01 | 6.90E-08 |
| 242 | DED-IBD | 10q11.21 | rs760466 | C | T | 9.77E-01 | 1.42E-04 | 9.25E-01 | 2.80E-05 |
| 243 | DED-IBD | 21q21.1 | rs1297260 | C | T | 1.01E+00 | 8.31E-03 | 1.13E+00 | 1.70E-12 |
| 244 | DED-PUD | 10p12.31 | rs10828248 | A | G | 9.62E-01 | 1.70E-12 | 9.73E-01 | 1.80E-02 |
| 245 | DED-PUD | 19q13.33 | rs35866622 | C | T | 1.02E+00 | 3.99E-04 | 9.22E-01 | 7.40E-13 |
| 246 | DED-IBS | 2q22.3 | rs28469251 | C | T | 9.79E-01 | 1.07E-04 | 1.03E+00 | 1.10E-05 |
| 247 | DED-IBS | 3p14.3 | rs7631010 | A | T | 1.03E+00 | 2.69E-06 | 1.03E+00 | 5.27E-04 |
| 248 | DED-IBS | 5p15.31 | rs150079703 | C | G | 1.02E+00 | 7.58E-05 | 1.04E+00 | 4.21E-07 |
| 249 | DED-IBS | 11q23.2 | rs7128314 | C | T | 9.76E-01 | 1.29E-05 | 1.04E+00 | 1.14E-07 |
| 250 | DED-IBS | 16q21 | rs35418299 | A | G | 1.02E+00 | 3.79E-04 | 1.03E+00 | 4.76E-06 |
| 251 | DED-DD | 1q42.2 | rs4333882 | A | G | 9.85E-01 | 6.26E-02 | 9.93E-01 | 2.50E-24 |
| 252 | DED-DD | 2q22.3 | rs28469251 | C | T | 9.79E-01 | 1.07E-04 | 9.95E-01 | 2.10E-20 |
| 253 | DED-DD | 6q16.1 | rs4839715 | G | A | 1.03E+00 | 9.64E-07 | 1.00E+00 | 1.40E-11 |
| 254 | DED-DD | 7q31.1 | rs2690837 | G | C | 1.02E+00 | 1.04E-04 | 1.00E+00 | 7.00E-06 |
| 255 | DED-DD | 10p12.31 | rs11012732 | A | G | 9.62E-01 | 6.76E-12 | 9.98E-01 | 2.90E-04 |
| 256 | DED-DD | 11p11.2 | rs3781628 | G | T | 1.03E+00 | 3.31E-05 | 9.97E-01 | 1.40E-05 |
| 257 | DED-DD | 18q11.2 | rs12455655 | A | T | 1.02E+00 | 3.69E-04 | 9.89E-01 | 3.70E-01 |
| 258 | DED-DD | 19q13.2 | rs35478630 | A | G | 1.02E+00 | 1.37E-03 | 9.96E-01 | 1.80E-08 |
| 259 | DED-DD | 22q13.2 | rs5995893 | T | C | 9.76E-01 | 5.49E-04 | 9.96E-01 | 1.40E-07 |
| 260 | Keratitis-IBD | 1p31.3 | rs79755370 | C | A | 1.06E+00 | 4.32E-02 | 1.52E+00 | 1.80E-23 |
| 261 | Keratitis-IBD | 1q32.1 | rs3024493 | C | A | 9.55E-01 | 1.77E-02 | 8.42E-01 | 9.10E-15 |
| 262 | Keratitis-IBD | 7q31.1 | rs990107 | T | G | 9.33E-01 | 2.50E-05 | 1.09E+00 | 3.30E-07 |
| 263 | Keratitis-IBD | 9q31.2 | rs10739246 | A | G | 1.06E+00 | 4.57E-05 | 1.07E+00 | 8.20E-05 |
| 264 | Keratitis-IBS | 1p34.1 | rs12063694 | A | G | 9.26E-01 | 2.63E-06 | 9.72E-01 | 2.04E-04 |
| 265 | Keratitis-IBS | 5p15.31 | rs4562016 | G | A | 9.40E-01 | 4.45E-05 | 1.03E+00 | 1.66E-06 |
| 266 | Keratitis-IBS | 13q14.3 | rs5803650 | C | CT | 1.06E+00 | 6.78E-05 | 9.55E-01 | 2.97E-08 |
| 267 | Keratitis-DD | 1q21.3 | rs2012674 | C | G | 9.44E-01 | 2.15E-04 | 1.00E+00 | 8.40E-11 |
| 268 | Keratitis-DD | 2q22.3 | rs4372823 | A | G | 1.04E+00 | 2.54E-02 | 1.01E+00 | 4.30E-54 |
| 269 | Keratitis-DD | 3q25.1 | rs6790448 | A | G | 9.61E-01 | 7.26E-03 | 9.96E-01 | 2.30E-11 |
| 270 | Keratitis-DD | 10p12.1 | rs943985 | C | G | 9.72E-01 | 5.22E-02 | 1.01E+00 | 2.00E-21 |
| 271 | Keratitis-DD | 10q24.2 | rs35562233 | C | T | 9.33E-01 | 1.16E-03 | 9.95E-01 | 3.70E-12 |
| 272 | Myopia-GORD | 1p34.1 | rs1319055 | A | T | 9.57E-01 | 6.21E-07 | 9.67E-01 | 3.20E-04 |
| 273 | Myopia-GORD | 1q32.1 | rs6664603 | C | T | 9.55E-01 | 5.74E-14 | 9.78E-01 | 6.20E-04 |
| 274 | Myopia-GORD | 4q21.21 | rs77285094 | T | C | 8.71E-01 | 4.83E-16 | 9.62E-01 | 2.20E-02 |
| 275 | Myopia-GORD | 4q22.1 | rs7660000 | C | T | 1.04E+00 | 1.40E-11 | 1.02E+00 | 6.00E-03 |
| 276 | Myopia-GORD | 6p22.3 | rs55775505 | C | T | 1.05E+00 | 3.35E-15 | 1.02E+00 | 8.70E-03 |
| 277 | Myopia-GORD | 8q12.1 | rs12547193 | G | A | 1.07E+00 | 2.00E-16 | 1.02E+00 | 3.40E-02 |
| 278 | Myopia-GORD | 8q12.1 | rs10504295 | G | A | 1.03E+00 | 3.17E-07 | 1.02E+00 | 2.00E-04 |
| 279 | Myopia-GORD | 9p22.2 | rs2791442 | T | C | 9.58E-01 | 8.00E-12 | 1.02E+00 | 1.20E-03 |
| 280 | Myopia-GORD | 9q31.3 | rs113168638 | G | GT | 1.03E+00 | 3.26E-04 | 9.56E-01 | 1.40E-06 |
| 281 | Myopia-GORD | 10q21.1 | rs12258523 | T | C | 9.53E-01 | 7.90E-16 | 9.82E-01 | 6.80E-03 |
| 282 | Myopia-GORD | 11q14.1 | rs12281149 | T | C | 9.64E-01 | 2.57E-08 | 9.76E-01 | 1.70E-03 |
| 283 | Myopia-GORD | 13q32.3 | rs57756661 | C | T | 9.53E-01 | 1.78E-16 | 1.02E+00 | 6.60E-03 |
| 284 | Myopia-GORD | 14q22.2 | rs12898159 | C | T | 9.57E-01 | 1.29E-13 | 9.79E-01 | 8.90E-04 |
| 285 | Myopia-GORD | 16p13.3 | rs8060728 | C | G | 1.06E+00 | 1.12E-13 | 1.02E+00 | 5.40E-03 |
| 286 | Myopia-GORD | 17p12 | rs2969185 | C | A | 9.61E-01 | 1.04E-11 | 1.02E+00 | 1.70E-04 |
| 287 | Myopia-GORD | 18q12.3 | rs12455689 | T | C | 1.06E+00 | 1.48E-13 | 9.80E-01 | 1.30E-02 |
| 288 | Myopia-GORD | 19q13.32 | rs429358 | T | C | 1.05E+00 | 1.41E-07 | 1.04E+00 | 3.50E-05 |
| 289 | Myopia-IBD | 1p31.3 | rs7518660 | G | A | 1.02E+00 | 1.05E-02 | 1.15E+00 | 5.20E-16 |
| 290 | Myopia-IBD | 1q32.1 | rs2790110 | C | A | 9.53E-01 | 3.23E-16 | 9.66E-01 | 4.40E-02 |
| 291 | Myopia-IBD | 1q32.1 | rs41267497 | G | A | 1.02E+00 | 5.00E-03 | 1.15E+00 | 3.60E-09 |
| 292 | Myopia-IBD | 2p16.1 | rs11899888 | A | G | 9.36E-01 | 8.91E-15 | 9.45E-01 | 1.60E-02 |
| 293 | Myopia-IBD | 3q23 | rs7636914 | A | G | 1.04E+00 | 3.30E-10 | 9.52E-01 | 5.50E-03 |
| 294 | Myopia-IBD | 5p13.1 | rs7713270 | C | T | 9.84E-01 | 5.63E-03 | 8.98E-01 | 8.00E-10 |
| 295 | Myopia-IBD | 8q12.1 | rs7813733 | C | T | 9.72E-01 | 1.07E-05 | 9.19E-01 | 2.10E-06 |
| 296 | Myopia-IBD | 8q21.12 | rs1001947 | C | A | 1.03E+00 | 1.47E-05 | 9.29E-01 | 2.20E-04 |
| 297 | Myopia-IBD | 10q26.13 | rs2672592 | G | T | 9.66E-01 | 4.09E-08 | 1.06E+00 | 1.20E-03 |
| 298 | Myopia-IBD | 11q13.5 | rs7126418 | A | T | 1.02E+00 | 4.28E-04 | 8.97E-01 | 1.90E-10 |
| 299 | Myopia-IBD | 12p13.31 | rs7968679 | A | G | 9.57E-01 | 1.82E-12 | 9.51E-01 | 5.70E-03 |
| 300 | Myopia-IBD | 13q32.3 | rs837323 | C | T | 1.04E+00 | 7.70E-10 | 9.56E-01 | 8.50E-03 |
| 301 | Myopia-IBD | 14q22.2 | rs2738265 | C | G | 9.56E-01 | 1.79E-15 | 1.04E+00 | 1.20E-02 |
| 302 | Myopia-IBD | 15q14 | rs4924158 | T | C | 9.46E-01 | 3.68E-14 | 9.51E-01 | 1.70E-02 |
| 303 | Myopia-IBD | 16p13.3 | rs55977430 | G | A | 1.08E+00 | 6.43E-11 | 8.97E-01 | 1.10E-03 |
| 304 | Myopia-IBD | 18q12.3 | rs9952980 | T | C | 1.04E+00 | 2.40E-11 | 1.05E+00 | 1.10E-02 |
| 305 | Myopia-PUD | 1p31.1 | rs11588959 | G | T | 1.04E+00 | 9.59E-07 | 9.54E-01 | 1.10E-03 |
| 306 | Myopia-PUD | 1q313 | rs7535263 | G | A | 1.03E+00 | 4.83E-09 | 1.03E+00 | 5.10E-03 |
| 307 | Myopia-PUD | 2p25.3 | rs62114494 | C | T | 1.05E+00 | 7.26E-15 | 9.70E-01 | 7.90E-03 |
| 308 | Myopia-PUD | 2q24.1 | rs3769361 | A | G | 1.04E+00 | 3.55E-09 | 1.05E+00 | 2.00E-04 |
| 309 | Myopia-PUD | 3q23 | rs6440008 | T | C | 1.04E+00 | 1.15E-12 | 1.03E+00 | 6.30E-03 |
| 310 | Myopia-PUD | 6q13 | rs9446760 | T | C | 1.04E+00 | 2.36E-09 | 1.03E+00 | 6.50E-03 |
| 311 | Myopia-PUD | 8q12.1 | rs3110125 | A | G | 1.05E+00 | 1.30E-14 | 9.72E-01 | 1.10E-02 |
| 312 | Myopia-PUD | 19q21.1 | rs7900904 | G | T | 9.59E-01 | 2.09E-13 | 9.72E-01 | 1.10E-02 |
| 313 | Myopia-PUD | 11p15.4 | rs10500661 | T | C | 1.02E+00 | 9.14E-03 | 9.03E-01 | 4.10E-14 |
| 314 | Myopia-PUD | 14q22.2 | rs11623717 | A | G | 9.57E-01 | 2.34E-13 | 9.71E-01 | 9.00E-03 |
| 315 | Myopia-PUD | 15q14 | rs649782 | A | C | 9.63E-01 | 6.87E-11 | 1.03E+00 | 1.70E-02 |
| 316 | Myopia-PUD | 18q22.3 | rs12971120 | A | G | 1.04E+00 | 2.64E-10 | 9.60E-01 | 1.80E-03 |
| 317 | Myopia-PUD | 19q13.33 | rs601338 | G | A | 9.88E-01 | 3.73E-02 | 9.15E-01 | 2.40E-15 |
| 318 | Myopia-PUD | 20p12.3 | rs235766 | G | C | 1.04E+00 | 3.61E-09 | 9.68E-01 | 4.30E-03 |
| 319 | Myopia-IBS | 1q31.3 | rs17514253 | C | T | 1.00E+00 | 9.38E-01 | 1.61E+00 | 1.70E-02 |
| 320 | Myopia-IBS | 3q25.2 | rs408419 | A | T | 1.00E+00 | 9.85E-01 | 9.72E-01 | 1.13E-02 |
| 321 | Myopia-IBS | 10q26.13 | rs3887220 | C | T | 9.93E-01 | 2.92E-01 | 9.80E-01 | 3.03E-03 |
| 322 | Myopia-IBS | 19p13.3 | rs2279623 | A | C | 9.92E-01 | 3.20E-01 | 1.02E+00 | 8.70E-03 |
| 323 | Myopia-IBS | 22q13.1 | rs130651 | G | A | 1.01E+00 | 5.27E-01 | 9.80E-01 | 8.80E-03 |
| 324 | Myopia-DD | 1q21.3 | rs1979637 | G | C | 1.01E+00 | 2.84E-01 | 1.00E+00 | 1.40E-03 |
| 325 | Myopia-DD | 1q32.1 | rs34049751 | T | TA | 1.04E+00 | 2.45E-12 | 1.00E+00 | 8.30E-04 |
| 326 | Myopia-DD | 1q32.2 | rs6698351 | G | A | 1.04E+00 | 5.99E-10 | 1.00E+00 | 5.10E-05 |
| 327 | Myopia-DD | 1q41 | rs76926608 | G | A | 1.08E+00 | 1.10E-04 | 1.01E+00 | 1.20E-09 |
| 328 | Myopia-DD | 1q41 | rs2784272 | G | A | 1.02E+00 | 2.29E-04 | 1.00E+00 | 2.80E-08 |
| 329 | Myopia-DD | 2p16.1 | rs11899380 | G | A | 9.29E-01 | 1.27E-16 | 9.93E-01 | 4.60E-19 |
| 330 | Myopia-DD | 2q22.3 | rs7597834 | G | A | 1.02E+00 | 1.83E-02 | 1.01E+00 | 8.60E-55 |
| 331 | Myopia-DD | 2q35 | rs72951773 | A | T | 9.64E-01 | 1.75E-05 | 1.00E+00 | 6.00E-05 |
| 332 | Myopia-DD | 3p25.1 | rs12637948 | G | A | 9.68E-01 | 8.42E-04 | 1.00E+00 | 2.70E-07 |
| 333 | Myopia-DD | 4p16.1 | rs62290573 | A | G | 1.03E+00 | 6.71E-06 | 1.00E+00 | 2.40E-05 |
| 334 | Myopia-DD | 4q21.21 | rs1139638 | A | C | 9.54E-01 | 6.58E-11 | 1.00E+00 | 2.20E-03 |
| 335 | Myopia-DD | 5q12.3 | rs10471645 | T | C | 1.03E+00 | 1.24E-03 | 1.00E+00 | 2.10E-11 |
| 336 | Myopia-DD | 6q22.33 | rs7775030 | A | G | 1.05E+00 | 1.59E-13 | 1.00E+00 | 1.40E-02 |
| 337 | Myopia-DD | 7q36.3 | rs62485830 | C | T | 9.51E-01 | 9.52E-12 | 9.98E-01 | 3.80E-04 |
| 338 | Myopia-DD | 8q24.12 | rs4870765 | G | C | 9.81E-01 | 3.29E-03 | 9.96E-01 | 1.90E-09 |
| 339 | Myopia-DD | 10p12.33 | rs1888693 | G | A | 9.83E-01 | 3.57E-03 | 1.00E+00 | 2.70E-10 |
| 340 | Myopia-DD | 10p12.1 | rs12764415 | C | T | 1.03E+00 | 3.44E-03 | 1.00E+00 | 7.40E-10 |
| 341 | Myopia-DD | 10q21.1 | rs12262629 | G | A | 9.51E-01 | 3.42E-16 | 9.98E-01 | 2.40E-03 |
| 342 | Myopia-DD | 10q22.3 | rs11002137 | T | C | 9.64E-01 | 3.58E-10 | 9.98E-01 | 2.30E-03 |
| 343 | Myopia-DD | 12p12.31 | rs7968679 | A | G | 9.57E-01 | 1.82E-12 | 9.98E-01 | 1.30E-03 |
| 344 | Myopia-DD | 15q14 | rs1915807 | C | G | 9.63E-01 | 1.74E-10 | 9.98E-01 | 5.20E-03 |
| 345 | Myopia-DD | 15q24.1 | rs2028386 | C | G | 9.73E-01 | 4.45E-06 | 9.97E-01 | 1.50E-06 |
| 346 | Myopia-DD | 17p13.1 | rs12942267 | C | T | 9.59E-01 | 3.63E-12 | 9.96E-01 | 6.70E-11 |
| 347 | Myopia-DD | 17q11.2 | rs4605221 | C | T | 9.50E-01 | 1.48E-12 | 9.98E-01 | 5.90E-03 |
| 348 | Myopia-DD | 21q22.3 | rs13051496 | C | T | 9.77E-01 | 1.42E-03 | 1.00E+00 | 8.30E-01 |
| 349 | Myopia-DD | 22q13.33 | rs742184 | C | T | 9.74E-01 | 6.59E-05 | 1.00E+00 | 3.20E-06 |
| 350 | PACG-GORD | 4q24 | rs13135092 | A | G | 9.05E-01 | 5.65E-04 | 9.45E-01 | 7.60E-07 |
| 351 | PACG-GORD | 11q23.2 | rs4456284 | C | T | 9.18E-01 | 5.71E-04 | 9.49E-01 | 7.70E-08 |
| 352 | PACG-IBD | 1p36.13 | rs4654897 | T | C | 9.36E-01 | 4.76E-04 | 1.16E+00 | 5.70E-13 |
| 353 | PACG-IBD | 7q32.1 | rs11761199 | A | G | 9.44E-01 | 5.03E-04 | 9.18E-01 | 4.70E-07 |
| 354 | PACG-IBS | 6p22.3 | rs75982572 | G | A | 8.76E-01 | 3.01E-05 | 9.48E-01 | 9.34E-05 |
| 355 | PACG-IBS | 15q13.1 | rs6497279 | T | C | 1.66E+00 | 2.24E-09 | 6.00E-01 | 1.03E-04 |
| 356 | PACG-DD | 1q42.2 | rs4333882 | A | G | 9.57E-01 | 5.47E-02 | 9.93E-01 | 2.50E-24 |
| 357 | PACG-DD | 2p16.1 | rs11125608 | T | G | 9.23E-01 | 1.56E-06 | 1.00E+00 | 8.90E-10 |
| 358 | PACG-DD | 2q22.3 | rs36168014 | G | GT | 9.53E-01 | 4.78E-03 | 9.96E-01 | 4.00E-14 |
| 359 | PACG-DD | 3q25.1 | rs9810067 | C | G | 1.05E+00 | 5.07E-03 | 1.00E+00 | 8.40E-11 |
| 360 | PACG-DD | 5p13.2 | rs1862577 | G | A | 1.05E+00 | 6.57E-03 | 1.00E+00 | 2.50E-10 |
| 361 | PACG-DD | 5q22.1 | rs59011279 | C | T | 8.93E-01 | 2.01E-05 | 1.00E+00 | 4.60E-06 |
| 362 | PACG-DD | 10q24.2 | rs112381543 | G | A | 9.29E-01 | 3.35E-03 | 9.94E-01 | 3.80E-13 |
| 363 | PACG-DD | 11p14.1 | rs17309930 | C | A | 1.04E+00 | 3.81E-02 | 1.01E+00 | 6.80E-17 |
| 364 | PACG-DD | 15q13.1 | rs56743903 | G | A | 6.45E-01 | 2.13E-16 | 1.00E+00 | 7.00E-03 |
| 365 | PACG-DD | 15q24.1 | rs4886778 | C | A | 1.06E+00 | 4.04E-04 | 9.97E-01 | 1.60E-06 |
| 366 | PACG-DD | 17q23.2 | rs138760546 | A | C | 8.64E-01 | 4.60E-07 | 1.00E+00 | 8.40E-05 |

Note: OR and P value were obtained from single-trait GWAS of corresponding pairwise traits.

**Table S7.** Colocalized Loci Identified by Colocalization Analysis Performed on 366 Pleiotropic Loci

| No. | Trait-pairs | Region | Top SNP | Nearest Gene | Chr | Start | End | PP.H3 | PP.H4 |
| --- | --- | --- | --- | --- | --- | --- | --- | --- | --- |
| 1 | DR-GORD | 2p21 | rs1322 | *THADA* | 2 | 43449385 | 43818957 | 2.25E-02 | 9.12E-01 |
| 2 | DR-GORD | 2p16.1 | rs12997266 | *BCL11A* | 2 | 60603950 | 60672771 | 7.18E-02 | 1.10E-02 |
| 3 | DR-GORD | 2q36.3 | rs1522811 | *AC068138.1* | 2 | 226866226 | 227019461 | 9.08E-02 | 2.24E-01 |
| 4 | DR-GORD | 3p21.31 | rs73077175 | *IP6K1* | 3 | 49774658 | 49980596 | 6.18E-01 | 7.09E-02 |
| 5 | DR-GORD | 3q27.2 | rs12636310 | *IGF2BP2* | 3 | 185459675 | 185480388 | 4.11E-02 | 1.73E-01 |
| 6 | DR-GORD | 4p16.1 | rs5028371 | *ZNF518B* | 4 | 10421208 | 10513072 | 8.63E-02 | 2.41E-01 |
| 7 | DR-GORD | 4q24 | rs13107325 | *SLC39A8* | 4 | 103001649 | 103198082 | 1.34E-01 | 7.65E-01 |
| 8 | DR-GORD | 6p21.1 | rs7765815 | *SLC39A8* | 6 | 43804103 | 43826681 | 3.39E-01 | 8.87E-02 |
| 9 | DR-GORD | 10q22.3 | rs703994 | *ZMIZ1* | 10 | 80906729 | 80932298 | 8.85E-02 | 8.94E-03 |
| 10 | DR-GORD | 10q23.33 | rs11187145 | *Y_RNA* | 10 | 94312353 | 94478104 | 6.59E-02 | 8.62E-03 |
| 11 | DR-GORD | 10q25.2 | rs11451675 | *TCF7L2* | 10 | 114847444 | 114861304 | 1.14E-01 | 6.29E-03 |
| 12 | DR-GORD | 11p15.4 | rs233448 | *KCNQ1* | 11 | 2836213 | 2857897 | 5.17E-02 | 1.01E-02 |
| 13 | DR-GORD | 11q13.1 | rs12789028 | *LTBP3* | 11 | 65211979 | 65368434 | 3.21E-01 | 5.25E-01 |
| 14 | DR-GORD | 12q14.3 | rs1585897 | *HMGA2* | 12 | 66351826 | 66389968 | 5.46E-02 | 5.00E-03 |
| 15 | DR-GORD | 12q24.31 | rs10849888 | *KDM2B* | 12 | 121824291 | 121992762 | 3.46E-01 | 4.13E-02 |
| 16 | DR-GORD | 13q13.3 | rs9544488 | *NBEA* | 13 | 36047374 | 36095039 | 3.29E-02 | 7.93E-01 |
| 17 | DR-GORD | 14q24.3 | rs4903516 | *RP11-488C13.4* | 14 | 77352149 | 77385148 | 6.59E-02 | 2.01E-01 |
| 18 | DR-GORD | 16q12.2 | rs12928335 | *RPGRIP1L* | 16 | 53548788 | 53751546 | 1.43E-01 | 3.52E-03 |
| 19 | DR-GORD | 17q21.31 | rs2732686 | *RP11-259G18.3* | 17 | 43463493 | 44865498 | 7.21E-01 | 2.40E-01 |
| 20 | DR-GORD | 17q21.32 | rs11867603 | *GIP* | 17 | 46840529 | 47145848 | 2.24E-01 | 8.72E-02 |
| 21 | DR-GORD | 19p13.11 | rs7258722 | *CRTC1* | 19 | 18750269 | 18898681 | 9.37E-01 | 3.07E-03 |
| 22 | DR-GORD | 19p13.11 | rs111234557 | *MAU2* | 19 | 19370340 | 19657500 | 2.71E-01 | 3.61E-01 |
| 23 | DR-GORD | 19q13.32 | rs429358 | *APOE* | 19 | 45392254 | 45428234 | 2.19E-02 | 9.35E-01 |
| 24 | DR-GORD | 20q11.22 | rs143220569 | *PIGU* | 20 | 32719068 | 33412361 | 4.70E-02 | 5.42E-01 |
| 25 | DR-IBD | 1p31.3 | rs4385674 | *C1orf141:IL23R* | 1 | 67684934 | 67743552 | 5.29E-02 | 6.14E-03 |
| 26 | DR-IBD | 1p31.1 | rs7531118 | *RPL31P12* | 1 | 72748669 | 72838406 | 7.25E-02 | 1.60E-01 |
| 27 | DR-IBD | 1q32.1 | rs1008833 | *PIK3C2B* | 1 | 204400034 | 204551830 | 1.09E-01 | 1.47E-01 |
| 28 | DR-IBD | 1q32.1 | rs3024493 | *IL10* | 1 | 206933517 | 206968955 | 1.49E-01 | 2.61E-02 |
| 29 | DR-IBD | 2p21 | rs78487399 | *THADA* | 2 | 43449385 | 43907630 | 8.71E-03 | 9.87E-01 |
| 30 | DR-IBD | 2p16.1 | rs10181565 | *AC007131.1* | 2 | 59421886 | 59490261 | 1.16E-01 | 2.08E-02 |
| 31 | DR-IBD | 2q31.1 | rs2140046 | *NOSTRIN:SPC25* | 2 | 169706079 | 169744551 | 7.49E-02 | 2.67E-01 |
| 32 | DR-IBD | 3q27.2 | rs11708719 | *IGF2BP2* | 3 | 185515274 | 185515274 | 7.86E-02 | 1.73E-02 |
| 33 | DR-IBD | 4q24 | rs1813006 | *BANK1* | 4 | 102702364 | 103198082 | 1.90E-01 | 7.71E-02 |
| 34 | DR-IBD | 6p24.3 | rs1815311 | *RREB1* | 6 | 7232186 | 7255015 | 1.52E-01 | 1.70E-01 |
| 35 | DR-IBD | 6p22.3 | rs6456366 | *CDKAL1* | 6 | 20541180 | 20783394 | 2.03E-01 | 9.30E-02 |
| 36 | DR-IBD | 6p22.1 | rs1233370 | *SUMO2P1* | 6 | 29604124 | 29614419 | 1.00E+00 | 1.15E-05 |
| 37 | DR-IBD | 6p22.1 | rs11759908 | *TFEB* | 6 | 41702979 | 41702979 | 7.84E-01 | 9.60E-03 |
| 38 | DR-IBD | 6p22.1 | rs57266556 | *RP11-344J7.2* | 6 | 43791080 | 43801905 | 3.47E-01 | 6.22E-03 |
| 39 | DR-IBD | 7q36.3 | rs3802123 | *UBE3C* | 7 | 156927132 | 157063837 | 1.81E-01 | 3.91E-02 |
| 40 | DR-IBD | 8p11.21 | rs12549902 | *NKX6-3* | 8 | 41488652 | 41516581 | 1.35E-01 | 1.06E-02 |
| 41 | DR-IBD | 8q23.3 | rs3808434 | *TRPS1* | 8 | 116464988 | 116633906 | 6.70E-02 | 7.87E-02 |
| 42 | DR-IBD | 9q34.3 | rs11145912 | *SDCCAG3* | 9 | 139235823 | 139383579 | 9.81E-01 | 1.33E-03 |
| 43 | DR-IBD | 10q23.33 | rs4646955 | *IDE* | 10 | 94206326 | 94333955 | 8.51E-02 | 2.14E-02 |
| 44 | DR-IBD | 10q25.2 | rs4918784 | *RP11-57H14.3* | 10 | 114600396 | 114643471 | 7.04E-02 | 4.81E-02 |
| 45 | DR-IBD | 11q14.3 | rs12789669 | *DISC1FP1* | 11 | 89928597 | 90040889 | 1.83E-02 | 1.67E-01 |
| 46 | DR-IBD | 15q24.3 | rs12909924 | *PEAK1* | 15 | 77384002 | 77833718 | 3.58E-01 | 8.16E-02 |
| 47 | DR-IBD | 20q12 | rs74577821 | *ZHX3* | 20 | 39620847 | 40012021 | 1.32E-01 | 1.47E-01 |
| 48 | DR-PUD | 1q25.3 | rs12146099 | *RNU6-41P* | 1 | 182946746 | 183115341 | 7.93E-02 | 8.03E-01 |
| 49 | DR-PUD | 1q32.3 | rs61320678 | *PROX1* | 1 | 214173376 | 214192133 | 9.75E-02 | 3.36E-02 |
| 50 | DR-PUD | 2p23.3 | rs780093 | *GCKR* | 2 | 27598097 | 27752871 | 8.98E-02 | 1.00E-01 |
| 51 | DR-PUD | 2p21 | rs13029250 | *THADA* | 2 | 43449385 | 43851246 | 1.83E-01 | 7.03E-02 |
| 52 | DR-PUD | 2p16.1 | rs60635548 | *LINC01122* | 2 | 58870750 | 58991868 | 1.90E-01 | 5.54E-03 |
| 53 | DR-PUD | 2p16.1 | rs7589501 | *AC007381.3* | 2 | 60551365 | 60562714 | 2.22E-01 | 9.91E-03 |
| 54 | DR-PUD | 2q24.3 | rs17184707 | *SCN2A* | 2 | 166146520 | 166183665 | 9.59E-02 | 6.04E-03 |
| 55 | DR-PUD | 3p25.2 | rs2120825 | *PPARG* | 3 | 12329783 | 12413339 | 5.06E-02 | 3.28E-01 |
| 56 | DR-PUD | 3p24.3 | rs13094957 | *UBE2E2* | 3 | 23243300 | 23644184 | 4.48E-01 | 3.11E-02 |
| 57 | DR-PUD | 4p16.1 | rs6833959 | *WFS1* | 4 | 6276581 | 6279047 | 1.16E-01 | 7.53E-03 |
| 58 | DR-PUD | 4q31.22 | rs13140054 | *SLC10A7* | 4 | 147173341 | 147299995 | 8.16E-02 | 1.54E-01 |
| 59 | DR-PUD | 5q13.3 | rs7732628 | *ZBED3-AS1* | 5 | 76430636 | 76439250 | 6.88E-02 | 8.48E-01 |
| 60 | DR-PUD | 5q21.1 | rs79602013 | *SLCO6A1* | 5 | 100632099 | 102169794 | 3.36E-01 | 5.13E-02 |
| 61 | DR-PUD | 5q33.3 | rs115498181 | *SGCD* | 5 | 155586188 | 156174011 | 9.22E-01 | 6.37E-03 |
| 62 | DR-PUD | 6p22.3 | rs6905138 | *CDKAL1* | 6 | 20528834 | 20728731 | 7.20E-01 | 1.70E-03 |
| 63 | DR-PUD | 7p15.1 | rs1635851 | *JAZF1* | 7 | 28180818 | 28207300 | 8.40E-02 | 7.83E-02 |
| 64 | DR-PUD | 7q36.3 | rs1182442 | *UBE3C* | 7 | 156926134 | 157079517 | 1.07E-01 | 2.86E-01 |
| 65 | DR-PUD | 9p21.3 | rs12379111 | *CDKN2B-AS1* | 9 | 22128180 | 22132897 | 1.22E-01 | 7.63E-02 |
| 66 | DR-PUD | 9q34.2 | rs8176719 | *ABO* | 9 | 136132908 | 136155000 | 1.69E-03 | 9.95E-01 |
| 67 | DR-PUD | 10q21.1 | rs11592488 | *PCDH15* | 10 | 56629277 | 56706723 | 6.42E-02 | 6.71E-01 |
| 68 | DR-PUD | 10q22.3 | rs703965 | *ZMIZ1* | 10 | 80939219 | 80955067 | 9.75E-02 | 1.27E-02 |
| 69 | DR-PUD | 11p15.4 | rs45477500 | *KCNQ1:KCNQ1-AS1* | 11 | 2869452 | 2869452 | 1.19E-01 | 6.34E-03 |
| 70 | DR-PUD | 12p13.32 | rs117233107 | *CCND2-AS1* | 12 | 4328521 | 4328521 | 8.39E-02 | 1.83E-01 |
| 71 | DR-PUD | 17q12 | rs34473775 | *RP11-690G19.4* | 17 | 37389409 | 37780296 | 1.60E-01 | 2.22E-01 |
| 72 | DR-PUD | 18q21.2 | rs624244 | *TCF4* | 18 | 53183396 | 53183396 | 4.96E-02 | 1.75E-02 |
| 73 | DR-PUD | 19q13.32 | rs2341097 | *SIX5:AC074212.5* | 19 | 46219145 | 46374916 | 1.39E-01 | 1.32E-02 |
| 74 | DR-PUD | 19q13.33 | rs601338 | *FUT2* | 19 | 49103447 | 49254955 | 1.07E-01 | 4.85E-01 |
| 75 | DR-PUD | 21q21.1 | rs60893125 | *AF127577.12* | 21 | 16574661 | 16574661 | 3.35E-02 | 1.46E-01 |
| 76 | DR-IBS | 1p34.3 | rs61779310 | *MACF1* | 1 | 39551488 | 40088043 | 2.55E-01 | 4.53E-01 |
| 77 | DR-IBS | 2p23.3 | rs1260326 | *GCKR* | 2 | 27387429 | 28113911 | 6.44E-01 | 1.58E-01 |
| 78 | DR-IBS | 2p16.1 | rs6724384 | *LINC01122* | 2 | 58866584 | 58991868 | 1.23E-01 | 2.13E-02 |
| 79 | DR-IBS | 2q36.3 | rs13012754 | *AC068138.1* | 2 | 227026531 | 227199263 | 4.33E-04 | 4.53E-04 |
| 80 | DR-IBS | 3p24.3 | rs9848331 | *rs9848331* | 3 | 23212597 | 23605528 | 5.33E-01 | 1.10E-02 |
| 81 | DR-IBS | 3p12.1 | rs4334651 | *AC107025.1* | 3 | 84329787 | 84677528 | 9.14E-01 | 5.55E-03 |
| 82 | DR-IBS | 3q22.3 | rs10048942 | *PPP2R3A* | 3 | 135619585 | 135943509 | 5.78E-01 | 1.52E-01 |
| 83 | DR-IBS | 3q27.1 | rs9869577 | *EIF2B5:CLCN2* | 3 | 184066556 | 184066556 | 1.28E-01 | 4.11E-02 |
| 84 | DR-IBS | 3q27.3 | rs9860157 | *ST6GAL1* | 3 | 186657705 | 186663868 | 4.59E-02 | 7.05E-02 |
| 85 | DR-IBS | 5q21.1 | rs13188193 | *CTD-2154H6.1* | 5 | 101584734 | 102876932 | 7.27E-01 | 1.93E-02 |
| 86 | DR-IBS | 6p24.3 | rs9502564 | *RREB1* | 6 | 7230680 | 7230680 | 1.38E-01 | 3.78E-03 |
| 87 | DR-IBS | 6p21.1 | rs4365924 | *CCND3* | 6 | 41995361 | 41995361 | 3.51E-01 | 1.30E-02 |
| 88 | DR-IBS | 8p23.1 | rs2409729 | *XKR6* | 8 | 10982410 | 11086757 | 7.53E-02 | 4.28E-01 |
| 89 | DR-IBS | 8q23.3 | rs2049865 | *TRPS1* | 8 | 116464988 | 116632819 | 1.82E-04 | 2.48E-04 |
| 90 | DR-IBS | 9p21.3 | rs78432974 | *CDKN2B-AS1* | 9 | 22132576 | 22132897 | 1.20E-01 | 8.89E-03 |
| 91 | DR-IBS | 9q33.3 | rs4838083 | *DENND1A* | 9 | 126472839 | 126715275 | NA | NA |
| 92 | DR-IBS | 10q11.23 | rs200803583 | *NUTM2HP* | 10 | 52443318 | 52443318 | NA | NA |
| 93 | DR-IBS | 10q22.3 | rs2802362 | *ZMIZ1* | 10 | 80959973 | 80989082 | NA | NA |
| 94 | DR-IBS | 11p15.1 | rs1002226 | *RP1-239B22.5* | 11 | 17368013 | 17421886 | 1.38E-01 | 4.43E-03 |
| 95 | DR-IBS | 12q14.3 | rs1351394 | *HMGA2* | 12 | 66257355 | 66383320 | 2.54E-02 | 2.68E-01 |
| 96 | DR-IBS | 15q26.1 | rs4932148 | *AP3S2:C15orf38-AP3S2* | 15 | 90365422 | 90450289 | 1.38E-01 | 1.11E-01 |
| 97 | DR-IBS | 15q26.1 | rs28404354 | *PRC1* | 15 | 91505827 | 91571773 | 3.35E-02 | 4.26E-02 |
| 98 | DR-IBS | 17q21.31 | rs111274735 | *LINC00854* | 17 | 41172481 | 41457386 | NA | NA |
| 99 | DR-IBS | 17q21.32 | rs28627277 | *GIP* | 17 | 46745778 | 47045862 | NA | NA |
| 100 | DR-IBS | 20q11.22 | rs60012358 | *RP5-1125A11.1* | 20 | 32561680 | 32660190 | NA | NA |
| 101 | DR-IBS | 22q12.2 | rs41172 | *MTMR3:CTA-85E5.10* | 22 | 30130115 | 30584388 | 4.14E-02 | 7.38E-01 |
| 102 | DR-DD | 2p21 | rs7560838 | *PLEKHH2:AC011242.5* | 2 | 43451957 | 43907630 | 3.70E-02 | 2.30E-02 |
| 103 | DR-DD | 2p16.1 | rs7594743 | *LINC01122* | 2 | 59289176 | 59335104 | 3.13E-03 | 6.57E-03 |
| 104 | DR-DD | 2p16.1 | rs12999941 | *AC007381.3* | 2 | 60565274 | 60586707 | 6.67E-03 | 2.95E-03 |
| 105 | DR-DD | 2q22.3 | rs368217240 | *ARHGAP15:RP11-570L15.1* | 2 | 144274527 | 144349201 | 2.14E-02 | 9.46E-04 |
| 106 | DR-DD | 3p25.2 | rs2120825 | *PPARG* | 3 | 12329783 | 12471862 | 3.51E-02 | 3.35E-02 |
| 107 | DR-DD | 3q22.3 | rs4678411 | *MRAS* | 3 | 138050685 | 138123854 | 9.46E-03 | 8.08E-02 |
| 108 | DR-DD | 4q31.21 | rs11727676 | *HHIP* | 4 | 145621328 | 146087979 | 2.59E-03 | 9.89E-01 |
| 109 | DR-DD | 4q31.3 | rs2305980 | *DCLK2* | 4 | 151066145 | 151214005 | 6.46E-06 | 1.34E-05 |
| 110 | DR-DD | 5p13.2 | rs2973068 | *AC008869.1* | 5 | 37757064 | 37792042 | 5.61E-02 | 1.40E-02 |
| 111 | DR-DD | 5q21.1 | rs13188193 | *CTD-2154H6.1* | 5 | 102726073 | 102876932 | 6.25E-05 | 1.96E-04 |
| 112 | DR-DD | 6p24.3 | rs9379084 | *RREB1* | 6 | 7231843 | 7317551 | 1.15E-02 | 6.93E-04 |
| 113 | DR-DD | 6p22.3 | rs201464451 | *CDKAL1:RP3-348I23.2* | 6 | 20541180 | 20844151 | 8.91E-03 | 3.26E-02 |
| 114 | DR-DD | 6p21.1 | rs1563788 | *ZNF318* | 6 | 43260011 | 43354431 | 1.12E-01 | 3.96E-04 |
| 115 | DR-DD | 6q16.1 | rs5878501 | *RP11-436D23.1* | 6 | 98440843 | 98440843 | 1.29E-02 | 9.53E-03 |
| 116 | DR-DD | 7p21.2 | rs4719430 | *AC006045.3* | 7 | 15027607 | 15066045 | 1.89E-02 | 5.83E-03 |
| 117 | DR-DD | 7p15.1 | rs11448038 | *JAZF1-AS1* | 7 | 28138193 | 28247511 | 6.07E-03 | 1.86E-01 |
| 118 | DR-DD | 7q22.1 | rs7794717 | *FAM185A* | 7 | 102412361 | 102527785 | 3.47E-01 | 3.39E-02 |
| 119 | DR-DD | 8q23.3 | rs2049865 | *TRPS1* | 8 | 116464988 | 116645056 | 1.68E-05 | 1.65E-05 |
| 120 | DR-DD | 8q24.12 | rs1553506 | *AC027238.1* | 8 | 122256724 | 122273808 | 1.41E-08 | 5.92E-07 |
| 121 | DR-DD | 9q33.3 | rs12236873 | *DENND1A* | 9 | 126196925 | 126714308 | NA | NA |
| 122 | DR-DD | 9q34.2 | rs8176719 | *ABO* | 9 | 136132908 | 136339755 | NA | NA |
| 123 | DR-DD | 10p13 | rs11257658 | *RN7SL232P* | 10 | 12244079 | 12328010 | 1.37E-02 | 5.92E-03 |
| 124 | DR-DD | 10q24.33 | rs3752946 | *OBFC1* | 10 | 105591779 | 105672842 | NA | NA |
| 125 | DR-DD | 10q25.2 | rs11599737 | *TCF7L2* | 10 | 114891443 | 114891443 | NA | NA |
| 126 | DR-DD | 11p15.5 | rs2334418 | *AP006285.6* | 11 | 1692498 | 1715332 | 6.54E-02 | 6.17E-04 |
| 127 | DR-DD | 11p15.4 | rs234859 | *KCNQ1* | 11 | 2836213 | 2852886 | 5.82E-03 | 9.42E-04 |
| 128 | DR-DD | 11p14.1 | rs2353487 | *BDNF-AS:BDNF* | 11 | 27646247 | 27736207 | 2.15E-02 | 9.66E-03 |
| 129 | DR-DD | 11p11.2 | rs7107792 | *MTCH2* | 11 | 47401448 | 47918416 | 1.73E-01 | 3.09E-03 |
| 130 | DR-DD | 11q13.1 | rs583887 | *EFEMP2* | 11 | 65575263 | 65663547 | 2.77E-02 | 4.20E-03 |
| 131 | DR-DD | 12q24.31 | rs11408808 | *OASL* | 12 | 121432117 | 121463562 | 4.10E-02 | 7.21E-03 |
| 132 | DR-DD | 12q24.31 | rs889970 | *RNF34* | 12 | 121824291 | 121931864 | 3.71E-02 | 9.68E-04 |
| 133 | DR-DD | 15q15.1 | rs7164606 | *PHGR1* | 15 | 40642877 | 40722781 | 9.91E-01 | 3.43E-04 |
| 134 | DR-DD | 15q23 | rs71400388 | *PIAS1* | 15 | 68343218 | 68567752 | 6.65E-01 | 1.47E-03 |
| 135 | DR-DD | 16q23.1 | rs72804106 | *BCAR1* | 16 | 75234273 | 75517115 | 6.15E-01 | 7.77E-02 |
| 136 | DR-DD | 18q21.2 | rs624244 | *TCF4* | 18 | 52868977 | 53425763 | NA | NA |
| 137 | DR-DD | 18q21.32 | rs7240682 | *RP11-795H16.3* | 18 | 57732418 | 57912226 | NA | NA |
| 138 | DR-DD | 20q12 | rs17265513 | *ZHX3* | 20 | 39620847 | 39966771 | NA | NA |
| 139 | DR-DD | 21q22.3 | rs9976088 | *PCBP3* | 21 | 47155316 | 47371947 | NA | NA |
| 140 | Uveitis-IBD | 1p31.3 | rs11209026 | *IL23R* | 1 | 67596372 | 67764815 | 1.95E-02 | 6.99E-01 |
| 141 | Uveitis-IBD | 1q32.1 | rs905634 | *C1orf106* | 1 | 200874229 | 201014966 | 1.97E-01 | 1.43E-02 |
| 142 | Uveitis-IBD | 21q22.2 | rs2836878 | *RPL23AP12* | 21 | 40458508 | 40468838 | 8.15E-01 | 3.01E-03 |
| 143 | Uveitis-PUD | 2p22.1 | rs732707 | *SLC8A1* | 2 | 40813827 | 40858754 | 2.19E-01 | 8.46E-02 |
| 144 | Uveitis-PUD | 19q13.33 | rs368565 | *FUT2* | 19 | 49168942 | 49254955 | 1.19E-01 | 1.47E-02 |
| 145 | Uveitis-IBS | 4q35.2 | rs72718909 | *RP11-713C19.2* | 4 | 188743514 | 188838702 | 1.37E-02 | 4.90E-02 |
| 146 | Uveitis-DD | 2q22.3 | rs4146022 | *ARHGAP15* | 2 | 144275192 | 144444786 | 5.99E-03 | 6.26E-03 |
| 147 | AMD-GORD | 1q32.2 | rs7523273 | *C1orf132* | 1 | 207912179 | 208024062 | 7.68E-02 | 6.51E-03 |
| 148 | AMD-GORD | 6p22.2 | rs62396201 | *ZNF322* | 6 | 26350810 | 26789491 | 6.79E-01 | 5.09E-03 |
| 149 | AMD-GORD | 6p22.1 | rs17720293 | *ZKSCAN4* | 6 | 27249686 | 29211556 | 1.00E+00 | 1.12E-06 |
| 150 | AMD-GORD | 10q26.13 | rs111266031 | *PLEKHA1* | 10 | 124070153 | 124232915 | 6.73E-02 | 1.29E-02 |
| 151 | AMD-GORD | 19q13.32 | rs429358 | *APOE* | 19 | 45392254 | 45424351 | 1.39E-01 | 5.96E-01 |
| 152 | AMD-IBD | 1p31.3 | rs1343151 | *IL23R* | 1 | 67670213 | 67766926 | 3.05E-02 | 3.21E-03 |
| 153 | AMD-IBD | 1q31.3 | rs12130962 | *RP11-476B1.1* | 1 | 195741951 | 195741951 | 2.25E-01 | 3.69E-02 |
| 154 | AMD-IBD | 1q31.3 | rs188357630 | *KCNT2* | 1 | 196212077 | 197128358 | 3.04E-01 | 2.66E-02 |
| 155 | AMD-IBD | 1q31.3 | rs12144001 | *DENND1B* | 1 | 197570844 | 197570844 | 2.01E-01 | 4.64E-02 |
| 156 | AMD-IBD | 1q32.1 | rs296544 | *MROH3P* | 1 | 200874229 | 201027055 | 1.27E-01 | 5.77E-02 |
| 157 | AMD-IBD | 1q32.2 | rs2745979 | *C1orf132* | 1 | 207917499 | 208049502 | 9.77E-02 | 1.44E-01 |
| 158 | AMD-IBD | 6p22.1 | rs1233372 | *SUMO2P1* | 6 | 29609951 | 29609951 | 1.00E+00 | 1.20E-05 |
| 159 | AMD-IBD | 7q32.1 | rs3757387 | *IRF5* | 7 | 128567032 | 128688456 | 1.84E-01 | 3.93E-01 |
| 160 | AMD-IBD | 7q36.1 | rs243546 | *CUL1* | 7 | 148395484 | 148488964 | 1.21E-01 | 4.78E-01 |
| 161 | AMD-IBD | 10q26.13 | rs78200813 | *RNU6-728P* | 10 | 124106025 | 124106025 | 9.21E-02 | 1.69E-02 |
| 162 | AMD-IBD | 15q24.1 | rs12903896 | *CYP1A2* | 15 | 75031521 | 75281132 | 2.67E-02 | 9.44E-01 |
| 163 | AMD-IBD | 20q13.33 | rs6011033 | *RTEL1:RTEL1-TNFRSF6B* | 20 | 62275844 | 62373079 | 2.74E-01 | 3.98E-01 |
| 164 | AMD-IBD | 21q22.2 | rs2836884 | *RPL23AP12* | 21 | 40463283 | 40468838 | 3.96E-02 | 8.94E-03 |
| 165 | AMD-IBD | 22q13.1 | rs4820371 | *AL031590.1* | 22 | 39630017 | 39737810 | 9.61E-01 | 8.76E-04 |
| 166 | AMD-PUD | 1q31.3 | rs11585965 | *CFH* | 1 | 196219334 | 196884074 | 7.81E-02 | 2.02E-02 |
| 167 | AMD-PUD | 4q21.3 | rs17449582 | *MAPK10* | 4 | 87216986 | 87340481 | 1.85E-01 | 4.47E-01 |
| 168 | AMD-PUD | 5q13.3 | rs7707527 | *ZBED3-AS1* | 5 | 76430636 | 76435346 | 3.04E-02 | 4.18E-01 |
| 169 | AMD-PUD | 9p21.3 | rs10965246 | *CDKN2B-AS1* | 9 | 22132076 | 22136489 | 1.33E-01 | 1.66E-02 |
| 170 | AMD-PUD | 10q26.13 | rs72826311 | *BTBD16* | 10 | 124028529 | 124120567 | 9.45E-02 | 1.32E-02 |
| 171 | AMD-PUD | 16q24.1 | rs370509910 | *FOXF1* | 16 | 86542241 | 86573987 | 1.40E-01 | 7.93E-03 |
| 172 | AMD-PUD | 19q13.33 | rs2287922 | *RASIP1* | 19 | 49164952 | 49277753 | 7.91E-01 | 1.95E-01 |
| 173 | AMD-IBS | 1q31.3 | rs17514253 | *F13B* | 1 | 196251806 | 197814623 | 1.05E-01 | 3.05E-02 |
| 174 | AMD-IBS | 3q25.5 | rs408419 | *ARHGEF26* | 3 | 153758863 | 154088411 | 7.18E-02 | 3.90E-03 |
| 175 | AMD-IBS | 10q26.13 | rs3887220 | *BTBD16* | 10 | 124064829 | 124298099 | NA | NA |
| 176 | AMD-IBS | 19p13.3 | rs2279623 | *GPR108* | 19 | 6724444 | 6730855 | 1.08E-01 | 3.84E-03 |
| 177 | AMD-IBS | 22q13.1 | rs130651 | *PDGFB* | 22 | 39630017 | 39648097 | 4.32E-02 | 2.27E-02 |
| 178 | AMD-DD | 1p22.2 | rs112500140 | *GBP3* | 1 | 88514479 | 90490822 | 1.57E-03 | 3.78E-04 |
| 179 | AMD-DD | 2q24.3 | rs115488970 | *CTAGE14P* | 2 | 168557555 | 168560474 | 2.69E-06 | 7.75E-06 |
| 180 | AMD-DD | 8q24.3 | rs2447389 | *AGO2* | 8 | 140560344 | 141998477 | 4.22E-04 | 1.35E-04 |
| 181 | AMD-DD | 9p24.3 | rs1629857 | *RP11-143M1.4* | 9 | 102702 | 187493 | 6.63E-05 | 3.02E-05 |
| 182 | AMD-DD | 9p24.3 | rs583230 | *RNA5SP279* | 9 | 1558597 | 1598501 | 5.32E-04 | 1.26E-04 |
| 183 | AMD-DD | 9p24.2 | rs16919460 | *RP11-509J21.3* | 9 | 3694163 | 3816956 | 7.31E-04 | 1.82E-04 |
| 184 | AMD-DD | 9p24.1 | rs296859 | *RP11-307I14.4* | 9 | 4765926 | 4788865 | 2.18E-04 | 6.63E-05 |
| 185 | AMD-DD | 9p24.1 | rs2578273 | *GLDC* | 9 | 6623446 | 6625864 | 1.08E-03 | 2.73E-04 |
| 186 | AMD-DD | 9p24.1 | rs76752978 | *KDM4C* | 9 | 7013909 | 7057850 | 1.29E-03 | 2.97E-04 |
| 187 | AMD-DD | 9p23 | rs1928727 | *RP11-421B23.2* | 9 | 10714842 | 10761898 | 1.10E-03 | 2.48E-04 |
| 188 | AMD-DD | 9p21.2 | rs2171101 | *RP11-477G9.1* | 9 | 25999871 | 25999871 | 2.08E-04 | 4.79E-05 |
| 189 | AMD-DD | 11p15.5 | rs67912009 | *RNH1* | 11 | 467689 | 547502 | 5.98E-04 | 1.43E-04 |
| 190 | AMD-DD | 11p15.5 | rs10466744 | *CHID1* | 11 | 870524 | 870524 | 5.86E-04 | 1.48E-04 |
| 191 | AMD-DD | 11p14.3 | rs76747679 | *RP11-2F20.1* | 11 | 24132871 | 24162941 | 3.28E-04 | 8.38E-05 |
| 192 | AMD-DD | 13q14.11 | rs12430400 | *RGCC* | 13 | 41892538 | 42627724 | 2.66E-04 | 8.63E-05 |
| 193 | AMD-DD | 16q22.1 | rs113573226 | *CMTM4* | 16 | 66640416 | 66951216 | 6.48E-04 | 2.36E-04 |
| 194 | AMD-DD | 18p11.32 | rs2847613 | *TYMS* | 18 | 519982 | 679208 | 2.62E-02 | 7.17E-04 |
| 195 | AMD-DD | 18p11.31 | rs1546707 | *DLGAP1* | 18 | 4186069 | 4195364 | 1.07E-03 | 3.15E-04 |
| 196 | AMD-DD | 18q11.2 | rs76748134 | *KCTD1* | 18 | 24162121 | 24213499 | NA | NA |
| 197 | AMD-DD | 19p13.3 | rs150394100 | *AC010641.1* | 19 | 392705 | 392705 | 3.21E-03 | 2.31E-03 |
| 198 | AMD-DD | 19p13.3 | rs150383413 | *RNF126* | 19 | 646357 | 841590 | 5.34E-03 | 2.03E-03 |
| 199 | AMD-DD | 19p13.11 | rs34937778 | *ELL* | 19 | 17618510 | 19592065 | 1.18E-02 | 3.84E-04 |
| 200 | Cataract-GORD | 3p21.31 | rs1858828 | *SLC38A3* | 3 | 49897830 | 50250837 | 3.22E-01 | 4.89E-01 |
| 201 | Cataract-GORD | 4p12 | rs13104400 | *GABRB1* | 4 | 47289971 | 47350953 | 1.59E-01 | 1.14E-01 |
| 202 | Cataract-GORD | 6p22.2 | rs2024970 | *BTN1A1* | 6 | 26399737 | 26581168 | 2.20E-03 | 6.90E-04 |
| 203 | Cataract-GORD | 15q25.1 | rs12437863 | *KIAA1199* | 15 | 80993786 | 81068363 | 4.19E-02 | 5.58E-01 |
| 204 | Cataract-GORD | 19p13.11 | rs12973258 | *GATAD2A* | 19 | 19374061 | 19657500 | 2.40E-01 | 4.26E-01 |
| 205 | Cataract-IBD | 1p36.13 | rs3820330 | *RP11-91K11.2* | 1 | 20142270 | 20145298 | 6.38E-02 | 2.35E-03 |
| 206 | Cataract-IBD | 1p31.3 | rs41396545 | *C1orf141:IL23R* | 1 | 67689608 | 67730055 | 2.10E-01 | 4.69E-03 |
| 207 | Cataract-IBD | 1q23.3 | rs1801274 | *FCGR2A* | 1 | 161463601 | 161479745 | 1.98E-01 | 9.85E-03 |
| 208 | Cataract-IBD | 3p23.31 | rs9862080 | *BSN* | 3 | 49385350 | 49731861 | 6.49E-01 | 2.41E-02 |
| 209 | Cataract-IBD | 5p13.1 | rs2084031 | *AC108105.1* | 5 | 40320006 | 40440063 | 6.55E-02 | 1.81E-01 |
| 210 | Cataract-IBD | 7q31.1 | rs6964893 | *DLD* | 7 | 107486902 | 107586254 | 5.34E-02 | 8.47E-02 |
| 211 | Cataract-PUD | 17q21.2 | rs34074411 | *JUP* | 17 | 39867248 | 39867248 | 1.96E-02 | 3.41E-02 |
| 212 | Cataract-PUD | 19q13.41 | rs1710353 | *IGLON5* | 19 | 51797119 | 51802650 | 7.83E-02 | 7.81E-02 |
| 213 | Cataract-IBS | 1p34.3 | rs41267043 | *BMP8A:OXCT2P1* | 1 | 39944249 | 40088043 | 2.14E-01 | 2.14E-01 |
| 214 | Cataract-IBS | 3q26.33 | rs7611110 | *RP11-416O18.2* | 3 | 181935178 | 181979272 | 8.78E-03 | 1.94E-01 |
| 215 | Cataract-IBS | 4p16.2 | rs7670828 | *STK32B* | 4 | 5403092 | 5411046 | 2.39E-02 | 4.29E-01 |
| 216 | Cataract-IBS | 4q25 | rs147098535 | *RNU6-289P* | 4 | 112303764 | 112503872 | 1.56E-04 | 3.31E-04 |
| 217 | Cataract-IBS | 4q32.1 | rs11100094 | *GLRB* | 4 | 157996099 | 158108596 | 1.20E-04 | 2.00E-04 |
| 218 | Cataract-IBS | 9q31.2 | rs2818292 | *RP11-308N19.1* | 9 | 108901517 | 109150784 | NA | NA |
| 219 | Cataract-IBS | 11q23.2 | rs1940725 | *NCAM1* | 11 | 112826867 | 112938783 | 9.94E-01 | 6.32E-04 |
| 220 | Cataract-IBS | 11q23.2 | rs4319542 | *DRD2* | 11 | 113317745 | 113451229 | 2.29E-01 | 1.24E-01 |
| 221 | Cataract-DD | 1q42.2 | rs4333882 | *SLC35F3* | 1 | 234351328 | 234365086 | 7.32E-02 | 6.65E-02 |
| 222 | Cataract-DD | 2q22.3 | rs138141174 | *ARHGAP15* | 2 | 144383183 | 144503445 | 1.52E-02 | 7.11E-03 |
| 223 | Cataract-DD | 6q16.1 | rs4339469 | *RP11-436D23.1* | 6 | 98310291 | 98546547 | 1.29E-01 | 3.94E-01 |
| 224 | Cataract-DD | 7q11.23 | rs2528794 | *ELN* | 7 | 73474825 | 73552627 | 1.19E-03 | 5.65E-04 |
| 225 | Cataract-DD | 9q34.2 | rs8176719 | *ABO* | 9 | 136132908 | 136149500 | NA | NA |
| 226 | Cataract-DD | 10p12.1 | rs12268257 | *GPR158* | 10 | 25864681 | 25917499 | 8.13E-02 | 3.61E-03 |
| 227 | Cataract-DD | 10q24.33 | rs10748858 | *RP11-541N10.3* | 10 | 105584159 | 105672842 | NA | NA |
| 228 | Cataract-DD | 11p14.1 | rs2353487 | *BDNF-AS:BDNF* | 11 | 27646247 | 27736207 | 5.20E-02 | 8.72E-03 |
| 229 | DED-GORD | 4q24 | rs13107325 | *SLC39A8* | 4 | 103001649 | 103198082 | 8.34E-03 | 9.85E-01 |
| 230 | DED-GORD | 6p22.2 | rs7755997 | *BTN2A1* | 6 | 26350810 | 26893329 | 1.57E-01 | 4.45E-01 |
| 231 | DED-GORD | 6p21.32 | rs79984539 | *ZBTB9* | 6 | 33460609 | 33478116 | 4.08E-01 | 1.75E-01 |
| 232 | DED-GORD | 11q23.2 | rs7942723 | *NCAM1* | 11 | 112826867 | 112938783 | 2.50E-01 | 5.51E-01 |
| 233 | DED-GORD | 19p13.11 | rs12462498 | *CRTC1* | 19 | 18750269 | 18898681 | 1.96E-01 | 6.28E-01 |
| 234 | DED-IBD | 1p36.13 | rs72658556 | *RP11-91K11.2* | 1 | 20163440 | 20238860 | 8.96E-02 | 2.56E-02 |
| 235 | DED-IBD | 1q23.3 | rs4657040 | *FCGR2A* | 1 | 161477028 | 161477028 | 2.14E-01 | 1.47E-02 |
| 236 | DED-IBD | 1q32.1 | rs3122605 | *IL10* | 1 | 206939904 | 206968955 | 5.53E-02 | 3.21E-02 |
| 237 | DED-IBD | 3p21.31 | rs3197999 | *MST1* | 3 | 49385350 | 49731861 | 7.62E-02 | 2.00E-01 |
| 238 | DED-IBD | 4q24 | rs6855246 | *SLC39A8* | 4 | 103001649 | 103198082 | 1.16E-01 | 4.25E-01 |
| 239 | DED-IBD | 5q31.1 | rs11749300 | *AC063976.1* | 5 | 131336105 | 131607402 | 1.77E-01 | 4.87E-02 |
| 240 | DED-IBD | 7q31.1 | rs2237690 | *LAMB1* | 7 | 107499725 | 107594549 | 3.15E-01 | 1.60E-02 |
| 241 | DED-IBD | 7q32.1 | rs3823536 | *IRF5* | 7 | 128567032 | 128584084 | 6.42E-02 | 1.07E-01 |
| 242 | DED-IBD | 10q11.21 | rs760466 | *RET* | 10 | 43612226 | 43758782 | 2.01E-01 | 1.66E-01 |
| 243 | DED-IBD | 21q21.1 | rs1297260 | *AJ006998.2* | 21 | 16804330 | 16841303 | 7.02E-02 | 7.26E-02 |
| 244 | DED-PUD | 10p12.31 | rs10828248 | *MLLT10* | 10 | 21766969 | 22288132 | 2.06E-01 | 5.18E-02 |
| 245 | DED-PUD | 19q13.33 | rs35866622 | *MAMSTR* | 19 | 49168942 | 49254955 | 2.59E-01 | 5.00E-02 |
| 246 | DED-IBS | 2q22.3 | rs28469251 | *ARHGAP15* | 2 | 144383727 | 144427710 | 1.49E-03 | 1.50E-03 |
| 247 | DED-IBS | 3p14.3 | rs7631010 | *PDHB* | 3 | 58230436 | 58481236 | 1.93E-01 | 3.85E-01 |
| 248 | DED-IBS | 5p15.31 | rs150079703 | *RP11-122F24.1* | 5 | 7188657 | 7302389 | 8.63E-02 | 7.85E-01 |
| 249 | DED-IBS | 11q23.2 | rs7128314 | *NCAM1* | 11 | 112826709 | 112938783 | 9.91E-02 | 8.23E-01 |
| 250 | DED-IBS | 16q21 | rs35418299 | *GNPATP* | 16 | 60665658 | 60743834 | 1.02E-01 | 3.26E-01 |
| 251 | DED-DD | 1q42.2 | rs4333882 | *SLC35F3* | 1 | 234352899 | 234353053 | 6.11E-02 | 1.99E-02 |
| 252 | DED-DD | 2q22.3 | rs28469251 | *ARHGAP15* | 2 | 144274527 | 144503445 | 1.05E-01 | 8.09E-03 |
| 253 | DED-DD | 6q16.1 | rs4839715 | *RP11-436D23.1* | 6 | 98214814 | 98546547 | 2.42E-01 | 7.56E-01 |
| 254 | DED-DD | 7q31.1 | rs2690837 | *FOXP2* | 7 | 113960155 | 114036155 | 2.92E-02 | 1.43E-01 |
| 255 | DED-DD | 10p12.31 | rs11012732 | *MLLT10* | 10 | 21768560 | 22288132 | 8.47E-03 | 4.18E-02 |
| 256 | DED-DD | 11p11.2 | rs3781628 | *PSMC3* | 11 | 47290759 | 48439503 | 1.81E-01 | 2.33E-02 |
| 257 | DED-DD | 18q11.2 | rs12455655 | *RP11-863N1.4* | 18 | 20010871 | 20037597 | NA | NA |
| 258 | DED-DD | 19q13.2 | rs35478630 | *KCNK6* | 19 | 38815217 | 38815217 | 3.40E-02 | 4.23E-03 |
| 259 | DED-DD | 22q13.2 | rs5995893 | *GAPDHP37* | 22 | 40558064 | 41062415 | 2.81E-01 | 1.17E-01 |
| 260 | Keratitis-IBD | 1p31.3 | rs79755370 | *IL23R* | 1 | 67684934 | 67743552 | 1.21E-01 | 7.13E-02 |
| 261 | Keratitis-IBD | 1q32.1 | rs3024493 | *IL10* | 1 | 206939904 | 206968955 | 1.28E-01 | 8.87E-02 |
| 262 | Keratitis-IBD | 7q31.1 | rs990107 | *PIGCP2* | 7 | 107476870 | 107584780 | 9.99E-01 | 2.33E-05 |
| 263 | Keratitis-IBD | 9q31.2 | rs10739246 | *KLF4* | 9 | 110241529 | 110274529 | 4.77E-02 | 7.12E-01 |
| 264 | Keratitis-IBS | 1p34.1 | rs12063694 | *ERI3* | 1 | 44589615 | 44853953 | 1.15E-01 | 5.45E-01 |
| 265 | Keratitis-IBS | 5p15.31 | rs4562016 | *RP11-404K5.3* | 5 | 7188657 | 7270814 | 8.71E-02 | 8.40E-01 |
| 266 | Keratitis-IBS | 13q14.3 | rs5803650 | *AL450423.1* | 13 | 53893310 | 53976774 | 7.59E-02 | 6.87E-02 |
| 267 | Keratitis-DD | 1q21.3 | rs2012674 | *NBPF18P* | 1 | 151836675 | 151996692 | 7.70E-02 | 5.86E-01 |
| 268 | Keratitis-DD | 2q22.3 | rs4372823 | *ARHGAP15:RP11-570L15.2:RP11-570L15.1* | 2 | 144274527 | 144503445 | 3.23E-02 | 2.46E-02 |
| 269 | Keratitis-DD | 3q25.1 | rs6790448 | *MED12L:P2RY12* | 3 | 150977426 | 151079041 | 1.34E-01 | 9.28E-02 |
| 270 | Keratitis-DD | 10p12.1 | rs943985 | *GPR158* | 10 | 25786532 | 25824297 | 7.88E-02 | 2.12E-02 |
| 271 | Keratitis-DD | 10q24.2 | rs35562233 | *snoU13* | 10 | 101357014 | 101485770 | NA | NA |
| 272 | Myopia-GORD | 1p34.1 | rs1319055 | *PIK3R3* | 1 | 46609736 | 46645681 | 4.11E-02 | 1.92E-01 |
| 273 | Myopia-GORD | 1q32.1 | rs6664603 | *ZNF281* | 1 | 200299701 | 200463931 | 5.78E-02 | 8.75E-02 |
| 274 | Myopia-GORD | 4q21.21 | rs77285094 | *BMP3* | 4 | 81951344 | 82002665 | 4.73E-02 | 4.41E-02 |
| 275 | Myopia-GORD | 4q22.1 | rs7660000 | *FAM13A* | 4 | 89745856 | 89779909 | 4.54E-02 | 6.23E-02 |
| 276 | Myopia-GORD | 6p22.3 | rs55775505 | *CASC15* | 6 | 22056923 | 22107290 | 2.02E-01 | 7.74E-02 |
| 277 | Myopia-GORD | 8q12.1 | rs12547193 | *SNORA51* | 8 | 60043089 | 60194032 | 1.12E-01 | 2.41E-02 |
| 278 | Myopia-GORD | 8q12.1 | rs10504295 | *RP11-379I19.3* | 8 | 60485588 | 60954059 | 3.15E-01 | 1.86E-02 |
| 279 | Myopia-GORD | 9p22.2 | rs2791442 | *RP11-570H19.2* | 9 | 18305059 | 18380943 | 1.59E-01 | 2.29E-01 |
| 280 | Myopia-GORD | 9q31.3 | rs113168638 | *TMEM245* | 9 | 111630324 | 111923048 | 9.02E-02 | 5.54E-01 |
| 281 | Myopia-GORD | 10q21.1 | rs12258523 | *BICC1* | 10 | 60253364 | 60375857 | 6.58E-02 | 2.18E-02 |
| 282 | Myopia-GORD | 11q14.1 | rs12281149 | *DLG2* | 11 | 83173973 | 83246281 | 4.69E-02 | 2.29E-01 |
| 283 | Myopia-GORD | 13q32.3 | rs57756661 | *PCCA* | 13 | 100740587 | 100783325 | 6.50E-02 | 3.80E-03 |
| 284 | Myopia-GORD | 14q22.2 | rs12898159 | *MIR5580* | 14 | 54410599 | 54435129 | 4.46E-02 | 3.01E-02 |
| 285 | Myopia-GORD | 16p13.3 | rs8060728 | *RBFOX1* | 16 | 7356555 | 7419121 | 1.80E-01 | 3.87E-02 |
| 286 | Myopia-GORD | 17p12 | rs2969185 | *SHISA6* | 17 | 11395390 | 11487165 | 9.33E-01 | 2.72E-02 |
| 287 | Myopia-GORD | 18q12.3 | rs12455689 | *SLC14A2* | 18 | 42875827 | 42918014 | 6.03E-01 | 3.42E-03 |
| 288 | Myopia-GORD | 19q13.32 | rs429358 | *APOE* | 19 | 45392254 | 45424351 | 3.33E-02 | 8.98E-01 |
| 289 | Myopia-IBD | 1p31.3 | rs7518660 | *C1orf141:IL23R* | 1 | 67669634 | 67730055 | 4.39E-02 | 7.02E-03 |
| 290 | Myopia-IBD | 1q32.1 | rs2790110 | *LINC00862* | 1 | 200271103 | 200421747 | 1.00E+00 | 7.86E-09 |
| 291 | Myopia-IBD | 1q32.1 | rs41267497 | *CACNA1S* | 1 | 200785961 | 201020360 | 1.00E+00 | 7.26E-09 |
| 292 | Myopia-IBD | 2p16.1 | rs11899888 | *EFEMP1* | 2 | 56040035 | 56102744 | 1.59E-01 | 2.60E-02 |
| 293 | Myopia-IBD | 3q23 | rs7636914 | *ZBTB38* | 3 | 141158212 | 141336557 | 1.08E-01 | 1.74E-02 |
| 294 | Myopia-IBD | 5p13.1 | rs7713270 | *AC108105.1* | 5 | 40320006 | 40440063 | 8.81E-02 | 9.58E-02 |
| 295 | Myopia-IBD | 8q12.1 | rs7813733 | *TOX* | 8 | 59798998 | 59925249 | 9.51E-01 | 9.59E-04 |
| 296 | Myopia-IBD | 8q21.12 | rs1001947 | *RP11-38H17.1* | 8 | 78634757 | 78767843 | 1.81E-01 | 5.84E-02 |
| 297 | Myopia-IBD | 10q26.13 | rs2672592 | *HTRA1* | 10 | 124202126 | 124230750 | 1.01E-01 | 4.00E-02 |
| 298 | Myopia-IBD | 11q13.5 | rs7126418 | *RP11-672A2.7* | 11 | 76270683 | 76302073 | 9.60E-02 | 4.17E-01 |
| 299 | Myopia-IBD | 12p13.31 | rs7968679 | *PZP* | 12 | 9212951 | 9313304 | 7.12E-02 | 1.91E-01 |
| 300 | Myopia-IBD | 13q32.3 | rs837323 | *PCCA* | 13 | 101175664 | 101259427 | 1.70E-01 | 1.08E-02 |
| 301 | Myopia-IBD | 14q22.2 | rs2738265 | *BMP4* | 14 | 54419965 | 54435054 | 2.55E-01 | 2.49E-02 |
| 302 | Myopia-IBD | 15q14 | rs4924158 | *GJD2* | 15 | 34982889 | 35010136 | 6.32E-03 | 1.99E-03 |
| 303 | Myopia-IBD | 16p13.3 | rs55977430 | *RBFOX1* | 16 | 7449740 | 7471241 | NA | NA |
| 304 | Myopia-IBD | 18q12.3 | rs9952980 | *SLC14A2* | 18 | 42884026 | 42919925 | NA | NA |
| 305 | Myopia-PUD | 1p31.1 | rs11588959 | *RPL31P12* | 1 | 72749726 | 72845264 | 6.27E-02 | 5.19E-01 |
| 306 | Myopia-PUD | 1q313 | rs7535263 | *CFH* | 1 | 196679455 | 196900709 | 7.93E-02 | 1.85E-01 |
| 307 | Myopia-PUD | 2p25.3 | rs62114494 | *SH3YL1* | 2 | 30762 | 305203 | 1.08E-01 | 4.76E-02 |
| 308 | Myopia-PUD | 2q24.1 | rs3769361 | *GPD2* | 2 | 157307905 | 157477952 | 1.15E-01 | 6.99E-01 |
| 309 | Myopia-PUD | 3q23 | rs6440008 | *ZBTB38* | 3 | 141093285 | 141336557 | 1.19E-01 | 3.10E-02 |
| 310 | Myopia-PUD | 6q13 | rs9446760 | *KCNQ5* | 6 | 73476094 | 73528905 | 8.45E-02 | 5.16E-03 |
| 311 | Myopia-PUD | 8q12.1 | rs3110125 | *SNORA51* | 8 | 60033144 | 60170476 | 6.03E-02 | 8.44E-03 |
| 312 | Myopia-PUD | 19q21.1 | rs7900904 | *BICC1* | 10 | 60228731 | 60374898 | 1.49E-01 | 4.60E-02 |
| 313 | Myopia-PUD | 11p15.4 | rs10500661 | *CCKBR* | 11 | 6214230 | 6289118 | 5.47E-02 | 1.02E-01 |
| 314 | Myopia-PUD | 14q22.2 | rs11623717 | *MIR5580* | 14 | 54410919 | 54418411 | 5.78E-02 | 6.84E-03 |
| 315 | Myopia-PUD | 15q14 | rs649782 | *GJD2* | 15 | 35004749 | 35005068 | 5.96E-02 | 9.67E-03 |
| 316 | Myopia-PUD | 18q22.3 | rs12971120 | *CNDP2* | 18 | 72154931 | 72182965 | 8.33E-02 | 2.37E-01 |
| 317 | Myopia-PUD | 19q13.33 | rs601338 | *FUT2* | 19 | 49168942 | 49250239 | 8.33E-02 | 2.37E-01 |
| 318 | Myopia-PUD | 20p12.3 | rs235766 | *BMP2* | 20 | 6743306 | 6762719 | 6.72E-02 | 1.50E-01 |
| 319 | Myopia-IBS | 1q31.3 | rs17514253 | *F13B* | 1 | 196251806 | 197814623 | 1.30E-01 | 5.77E-03 |
| 320 | Myopia-IBS | 3q25.2 | rs408419 | *ARHGEF26* | 3 | 153758863 | 154088411 | 1.91E-02 | 5.08E-03 |
| 321 | Myopia-IBS | 10q26.13 | rs3887220 | *BTBD16* | 10 | 124064829 | 124298099 | NA | NA |
| 322 | Myopia-IBS | 19p13.3 | rs2279623 | *GPR108* | 19 | 6724444 | 6730855 | 1.59E-02 | 2.56E-03 |
| 323 | Myopia-IBS | 22q13.1 | rs130651 | *PDGFB* | 22 | 39630017 | 39648097 | 2.79E-03 | 1.79E-03 |
| 324 | Myopia-DD | 1q21.3 | rs1979637 | *NBPF18P:AL450992.6* | 1 | 151836675 | 152134136 | 2.24E-01 | 1.04E-01 |
| 325 | Myopia-DD | 1q32.1 | rs34049751 | *RP11-469A15.2* | 1 | 200299701 | 200464803 | 1.39E-02 | 4.96E-03 |
| 326 | Myopia-DD | 1q32.2 | rs6698351 | *RP11-6J21.2* | 1 | 207345819 | 207529576 | 6.61E-02 | 3.69E-04 |
| 327 | Myopia-DD | 1q41 | rs76926608 | *RP11-95P13.2* | 1 | 219268532 | 219704939 | 1.00E+00 | 4.53E-09 |
| 328 | Myopia-DD | 1q41 | rs2784272 | *HLX* | 1 | 221050400 | 221094967 | 4.75E-02 | 5.10E-01 |
| 329 | Myopia-DD | 2p16.1 | rs11899380 | *EFEMP1* | 2 | 55724295 | 56201773 | 2.89E-03 | 9.97E-01 |
| 330 | Myopia-DD | 2q22.3 | rs7597834 | *ARHGAP15:RP11-570L15.2:RP11-570L15.1* | 2 | 144207768 | 144426357 | 1.41E-02 | 1.10E-02 |
| 331 | Myopia-DD | 2q35 | rs72951773 | *NHEJ1:SLC23A3* | 2 | 219943558 | 220046840 | 4.72E-05 | 2.51E-04 |
| 332 | Myopia-DD | 3p25.1 | rs12637948 | *AC090945.1* | 3 | 15928013 | 16080729 | 9.82E-01 | 1.31E-04 |
| 333 | Myopia-DD | 4p16.1 | rs62290573 | *AFAP1* | 4 | 7844754 | 7939008 | 1.14E-01 | 4.14E-01 |
| 334 | Myopia-DD | 4q21.21 | rs1139638 | *ANTXR2* | 4 | 80801388 | 80873617 | 1.33E-03 | 4.35E-04 |
| 335 | Myopia-DD | 5q12.3 | rs10471645 | *CWC27* | 5 | 64239523 | 64330833 | 9.78E-03 | 1.79E-02 |
| 336 | Myopia-DD | 6q22.33 | rs7775030 | *LAMA2* | 6 | 129802971 | 129817167 | 2.52E-03 | 1.60E-03 |
| 337 | Myopia-DD | 7q36.3 | rs62485830 | *VIPR2* | 7 | 158852436 | 158870863 | 3.83E-03 | 5.25E-03 |
| 338 | Myopia-DD | 8q24.12 | rs4870765 | *AC027238.1* | 8 | 122236020 | 122271418 | 4.18E-08 | 6.81E-07 |
| 339 | Myopia-DD | 10p12.33 | rs1888693 | *CACNB2* | 10 | 18427984 | 18472265 | 1.42E-01 | 1.10E-01 |
| 340 | Myopia-DD | 10p12.1 | rs12764415 | *GPR158* | 10 | 25646519 | 25738933 | 2.05E-01 | 6.87E-03 |
| 341 | Myopia-DD | 10q21.1 | rs12262629 | *BICC1* | 10 | 60228731 | 60375857 | NA | NA |
| 342 | Myopia-DD | 10q22.3 | rs11002137 | *KCNMA1:RP11-619F23.2* | 10 | 79043417 | 79159158 | NA | NA |
| 343 | Myopia-DD | 12p12.31 | rs7968679 | *PZP* | 12 | 9212951 | 9313304 | 8.05E-03 | 2.85E-02 |
| 344 | Myopia-DD | 15q14 | rs1915807 | *GJD2* | 15 | 34982578 | 35004765 | 3.12E-03 | 4.32E-04 |
| 345 | Myopia-DD | 15q24.1 | rs2028386 | *LOXL1* | 15 | 74214883 | 74243246 | 6.27E-02 | 5.73E-01 |
| 346 | Myopia-DD | 17p13.1 | rs12942267 | *ZBTB4* | 17 | 7318061 | 7437665 | 7.80E-03 | 9.92E-01 |
| 347 | Myopia-DD | 17q11.2 | rs4605221 | *TMEM98* | 17 | 30963238 | 31251862 | NA | NA |
| 348 | Myopia-DD | 21q22.3 | rs13051496 | *COL6A1* | 21 | 47032395 | 47453019 | 3.58E-11 | 5.53E-08 |
| 349 | Myopia-DD | 22q13.33 | rs742184 | *MAPK12* | 22 | 50697119 | 50698157 | 2.69E-03 | 8.97E-02 |
| 350 | PACG-GORD | 4q24 | rs13135092 | *SLC39A8* | 4 | 103001649 | 103198082 | 2.02E-01 | 3.72E-01 |
| 351 | PACG-GORD | 11q23.2 | rs4456284 | *NCAM1* | 11 | 112829143 | 112910969 | 2.54E-01 | 1.96E-01 |
| 352 | PACG-IBD | 1p36.13 | rs4654897 | *OTUD3* | 1 | 20161288 | 20238860 | 6.30E-01 | 2.48E-02 |
| 353 | PACG-IBD | 7q32.1 | rs11761199 | *IRF5* | 7 | 128573967 | 128584084 | 9.32E-01 | 1.92E-02 |
| 354 | PACG-IBS | 6p22.3 | rs75982572 | *KIF13A* | 6 | 17758145 | 18009727 | 3.42E-01 | 8.62E-02 |
| 355 | PACG-IBS | 15q13.1 | rs6497279 | *HERC2* | 15 | 28344238 | 28573738 | 2.94E-02 | 7.72E-01 |
| 356 | PACG-DD | 1q42.2 | rs4333882 | *SLC35F3* | 1 | 234352899 | 234353053 | 1.24E-01 | 5.17E-02 |
| 357 | PACG-DD | 2p16.1 | rs11125608 | *EFEMP1* | 2 | 55989737 | 56161869 | 9.14E-01 | 6.75E-02 |
| 358 | PACG-DD | 2q22.3 | rs36168014 | *ARHGAP15:RP11-570L15.2:RP11-570L15.1* | 2 | 144275192 | 144363659 | 4.47E-02 | 3.74E-03 |
| 359 | PACG-DD | 3q25.1 | rs9810067 | *MED12L* | 3 | 150946047 | 151067730 | 2.00E-01 | 7.78E-02 |
| 360 | PACG-DD | 5p13.2 | rs1862577 | *WDR70* | 5 | 37683864 | 37751672 | 1.78E-01 | 2.98E-02 |
| 361 | PACG-DD | 5q22.1 | rs59011279 | *STARD4-AS1:NREP* | 5 | 110992642 | 111053222 | 4.08E-04 | 3.84E-04 |
| 362 | PACG-DD | 10q24.2 | rs112381543 | *snoU13* | 10 | 101357014 | 101491236 | NA | NA |
| 363 | PACG-DD | 11p14.1 | rs17309930 | *RP11-587D21.4* | 11 | 27646247 | 27748493 | 2.61E-01 | 6.22E-02 |
| 364 | PACG-DD | 15q13.1 | rs56743903 | *HERC2* | 15 | 28267533 | 28573738 | 3.81E-03 | 2.72E-03 |
| 365 | PACG-DD | 15q24.1 | rs4886778 | *LOXL1* | 15 | 74220599 | 74243246 | 1.27E-01 | 8.58E-02 |
| 366 | PACG-DD | 17q23.2 | rs138760546 | *BCAS3* | 17 | 58898689 | 59031608 | NA | NA |

Note: OR and P value were obtained from single-trait GWAS of corresponding pairwise traits.

**Table S8.** 354 Candidate Pleiotropic Genes Identified by MAGMA based on the mapped genes in FUMA

| **Trait Pair** | **GENE** | **CHR** | **START** | **STOP** | **N_SNPS_** | **N_PARAM_** | **N** | **ZSTAT** | **P** | **entrezID** |
| --- | --- | --- | --- | --- | --- | --- | --- | --- | --- | --- |
| AMD-GORD | *APOE* | 19 | 45409011 | 45412650 | 6 | 3 | 883214 | 5.9936 | 1.03E-09 | 348 |
| AMD-GORD | *ZNF322* | 6 | 26636518 | 26659980 | 69 | 8 | 883214 | 4.9261 | 4.19E-07 | 79692 |
| AMD-GORD | *ZNF311* | 6 | 28962562 | 28973093 | 30 | 8 | 883214 | 4.9129 | 4.49E-07 | 282890 |
| AMD-GORD | *TOMM40* | 19 | 45393826 | 45406946 | 55 | 11 | 883214 | 4.8258 | 6.97E-07 | 10452 |
| AMD-GORD | *ABT1* | 6 | 26597180 | 26600278 | 14 | 4 | 883214 | 4.7827 | 8.65E-07 | 29777 |
| AMD-GORD | *HMGN4* | 6 | 26538633 | 26546482 | 19 | 4 | 883214 | 4.6847 | 1.40E-06 | 10473 |
| AMD-GORD | *APOC1* | 19 | 45417504 | 45422606 | 11 | 5 | 883214 | 4.5926 | 2.19E-06 | 341 |
| AMD-GORD | *OR2J1* | 6 | 29068386 | 29069658 | 8 | 3 | 883214 | 4.5701 | 2.44E-06 | NA |
| AMD-IBD | *CFHR2* | 1 | 196788898 | 196928356 | 38 | 18 | 883214 | 7.0022 | 1.26E-12 | 3080 |
| AMD-IBD | *CSK* | 15 | 75074398 | 75095539 | 39 | 8 | 883214 | 6.6862 | 1.14E-11 | 1445 |
| AMD-IBD | *CFH* | 1 | 196621008 | 196716634 | 25 | 13 | 883214 | 6.3721 | 9.32E-11 | 3075 |
| AMD-IBD | *IRF5* | 7 | 128577666 | 128590089 | 38 | 10 | 883214 | 6.2545 | 1.99E-10 | 3663 |
| AMD-IBD | *CFHR4* | 1 | 196819371 | 196888102 | 16 | 9 | 883214 | 6.1991 | 2.84E-10 | 10877 |
| AMD-IBD | *C1orf106* | 1 | 200860176 | 200884863 | 72 | 15 | 883214 | 6.0548 | 7.03E-10 | 55765 |
| AMD-IBD | *IL23R* | 1 | 67632083 | 67725662 | 264 | 25 | 883214 | 6.0218 | 8.62E-10 | 149233 |
| AMD-IBD | *TNFRSF6B* | 20 | 62328021 | 62330037 | 10 | 2 | 883214 | 5.868 | 2.21E-09 | 8771 |
| AMD-IBD | *ULK3* | 15 | 75128457 | 75135687 | 15 | 3 | 883214 | 5.7243 | 5.19E-09 | 25989 |
| AMD-IBD | *RTEL1-TNFRSF6B* | 20 | 62290653 | 62330037 | 176 | 19 | 883214 | 5.7168 | 5.43E-09 | NA |
| AMD-IBD | *SCAMP2* | 15 | 75136071 | 75165706 | 40 | 9 | 883214 | 5.6365 | 8.67E-09 | 10066 |
| AMD-IBD | *ARFRP1* | 20 | 62329996 | 62339377 | 15 | 2 | 883214 | 5.6254 | 9.25E-09 | 10139 |
| AMD-IBD | *RTEL1* | 20 | 62289163 | 62328416 | 171 | 18 | 883214 | 5.5387 | 1.52E-08 | 51750 |
| AMD-IBD | *LMAN1L* | 15 | 75105057 | 75118099 | 32 | 6 | 883214 | 5.5336 | 1.57E-08 | 79748 |
| AMD-IBD | *COX5A* | 15 | 75212132 | 75230509 | 41 | 6 | 883214 | 5.3978 | 3.37E-08 | 9377 |
| AMD-IBD | *TNPO3* | 7 | 128594948 | 128695198 | 227 | 23 | 883214 | 5.3879 | 3.56E-08 | 23534 |
| AMD-IBD | *KIF21B* | 1 | 200938520 | 200992828 | 133 | 13 | 883214 | 5.3843 | 3.64E-08 | 101929275 |
| AMD-IBD | *MPI* | 15 | 75182346 | 75191798 | 15 | 5 | 883214 | 5.3807 | 3.71E-08 | 4351 |
| AMD-IBD | *ZGPAT* | 20 | 62338817 | 62367494 | 86 | 7 | 883214 | 5.3375 | 4.71E-08 | 84619 |
| AMD-IBD | *RP4-583P15.15* | 20 | 62340216 | 62370456 | 93 | 7 | 883214 | 5.3274 | 4.98E-08 | 54923 |
| AMD-IBD | *CUL1* | 7 | 148395006 | 148498128 | 236 | 21 | 883214 | 5.2654 | 6.99E-08 | 8454 |
| AMD-IBD | *CYP1A2* | 15 | 75041185 | 75048543 | 12 | 4 | 883214 | 5.2524 | 7.51E-08 | 1544 |
| AMD-IBD | *LIME1* | 20 | 62366815 | 62370456 | 10 | 3 | 883214 | 5.1243 | 1.49E-07 | 54923 |
| AMD-IBD | *FAM219B* | 15 | 75192328 | 75199462 | 8 | 3 | 883214 | 5.1067 | 1.64E-07 | 57184 |
| AMD-IBD | *ZBTB41* | 1 | 197122810 | 197169672 | 39 | 5 | 883214 | 4.9292 | 4.13E-07 | 360023 |
| AMD-IBD | *SLC2A4RG* | 20 | 62371214 | 62374858 | 11 | 3 | 883214 | 4.827 | 6.93E-07 | 56731 |
| AMD-IBD | *RP4-583P15.14* | 20 | 62369623 | 62371751 | 5 | 2 | 883214 | 4.8032 | 7.81E-07 | NA |
| AMD-IBD | *CACNA1S* | 1 | 201008642 | 201081694 | 237 | 44 | 883214 | 4.7487 | 1.02E-06 | 779 |
| AMD-IBD | *RPP25* | 15 | 75246757 | 75249805 | 1 | 1 | 883214 | 4.7378 | 1.08E-06 | 54913 |
| AMD-IBD | *F13B* | 1 | 197008321 | 197036397 | 30 | 3 | 883214 | 4.7349 | 1.10E-06 | 2165 |
| AMD-IBD | *STMN3* | 20 | 62271061 | 62284780 | 41 | 8 | 883214 | 4.6286 | 1.84E-06 | 50861 |
| AMD-PUD | *RASIP1* | 19 | 49223844 | 49243978 | 47 | 7 | 883214 | 7.964 | 8.33E-16 | 54922 |
| AMD-PUD | *FUT2* | 19 | 49199228 | 49209207 | 35 | 4 | 883214 | 7.7995 | 3.11E-15 | 2524 |
| AMD-PUD | *CFH* | 1 | 196621008 | 196716634 | 25 | 13 | 883214 | 7.7104 | 6.27E-15 | 3075 |
| AMD-PUD | *CFHR4* | 1 | 196819371 | 196888102 | 16 | 9 | 883214 | 7.7048 | 6.55E-15 | 10877 |
| AMD-PUD | *MAMSTR* | 19 | 49215999 | 49222978 | 24 | 5 | 883214 | 7.6659 | 8.88E-15 | 284358 |
| AMD-PUD | *CFHR2* | 1 | 196788898 | 196928356 | 38 | 18 | 883214 | 7.4057 | 6.52E-14 | 3080 |
| AMD-PUD | *FUT1* | 19 | 49251268 | 49258647 | 16 | 4 | 883214 | 6.6665 | 1.31E-11 | 2523 |
| AMD-PUD | *MAPK10* | 4 | 86936276 | 87515284 | 1692 | 50 | 883214 | 5.8096 | 3.13E-09 | 5602 |
| AMD-PUD | *IZUMO1* | 19 | 49244109 | 49250166 | 27 | 2 | 883214 | 5.5337 | 1.57E-08 | 284359 |
| AMD-PUD | *FOXF1* | 16 | 86544133 | 86548076 | 13 | 6 | 883214 | 4.9779 | 3.21E-07 | 2294 |
| AMD-PUD | *NTN5* | 19 | 49164664 | 49176338 | 22 | 6 | 883214 | 4.8089 | 7.59E-07 | 126147 |
| AMD-DD | *KCTD1* | 18 | 24034874 | 24237365 | 1 | 1 | 877986 | 7.7022 | 6.69E-15 | 284252 |
| AMD-DD | *ELL* | 19 | 18553473 | 18632937 | 1 | 1 | 877986 | 6.3894 | 8.33E-11 | 8178 |
| AMD-DD | *CMTM4* | 16 | 66648653 | 66730610 | 1 | 1 | 877986 | 5.7231 | 5.23E-09 | 146223 |
| AMD-DD | *AGO2* | 8 | 141541264 | 141645718 | 4 | 2 | 877986 | 4.8698 | 5.58E-07 | 27161 |
| DR-GORD | *TCF7L2* | 10 | 114710009 | 114927437 | 313 | 64 | 888536 | 6.4469 | 5.71E-11 | 6934 |
| DR-GORD | *APOE* | 19 | 45409011 | 45412650 | 6 | 3 | 888536 | 6.1728 | 3.36E-10 | 348 |
| DR-GORD | *APOC1* | 19 | 45417504 | 45422606 | 11 | 5 | 888536 | 6.1614 | 3.61E-10 | 341 |
| DR-GORD | *THADA* | 2 | 43393800 | 43823185 | 810 | 48 | 888536 | 6.1006 | 5.28E-10 | 63892 |
| DR-GORD | *MAP3K11* | 11 | 65365226 | 65382853 | 31 | 7 | 888536 | 6.0588 | 6.86E-10 | 4296 |
| DR-GORD | *LTBP3* | 11 | 65306276 | 65326401 | 27 | 7 | 888536 | 5.9972 | 1.00E-09 | 4054 |
| DR-GORD | *CRTC1* | 19 | 18794487 | 18893004 | 260 | 27 | 888536 | 5.9087 | 1.72E-09 | 23373 |
| DR-GORD | *ATP5G1* | 17 | 46970127 | 46973233 | 7 | 2 | 888536 | 5.7671 | 4.03E-09 | 516 |
| DR-GORD | *GIP* | 17 | 47035916 | 47045958 | 44 | 5 | 888536 | 5.7351 | 4.87E-09 | 2695 |
| DR-GORD | *SCYL1* | 11 | 65292548 | 65306175 | 18 | 6 | 888536 | 5.7195 | 5.34E-09 | 57410 |
| DR-GORD | *KANSL1* | 17 | 44107282 | 44302733 | 823 | 4 | 888536 | 5.6682 | 7.22E-09 | 101929776 |
| DR-GORD | *MAPT* | 17 | 43971748 | 44105700 | 748 | 5 | 888536 | 5.5934 | 1.11E-08 | 4137 |
| DR-GORD | *TOMM40* | 19 | 45393826 | 45406946 | 55 | 11 | 888536 | 5.5829 | 1.18E-08 | 10452 |
| DR-GORD | *CRHR1* | 17 | 43699267 | 43913194 | 1025 | 8 | 888536 | 5.5702 | 1.27E-08 | 1394 |
| DR-GORD | *STH* | 17 | 44076616 | 44077060 | 1 | 1 | 888536 | 5.5474 | 1.45E-08 | 246744 |
| DR-GORD | *ARL17B* | 17 | 44352150 | 44439130 | 35 | 3 | 888536 | 5.5425 | 1.49E-08 | 100506084 |
| DR-GORD | *UBE2Z* | 17 | 46985731 | 47006418 | 55 | 4 | 888536 | 5.5072 | 1.82E-08 | 65264 |
| DR-GORD | *SPPL2C* | 17 | 43922256 | 43924438 | 17 | 2 | 888536 | 5.4691 | 2.26E-08 | 162540 |
| DR-GORD | *WNT3* | 17 | 44839872 | 44910520 | 119 | 22 | 888536 | 5.4543 | 2.46E-08 | 101929777 |
| DR-GORD | *EHBP1L1* | 11 | 65343509 | 65360121 | 27 | 8 | 888536 | 5.444 | 2.60E-08 | 254102 |
| DR-GORD | *IGF2BP1* | 17 | 47074774 | 47133012 | 107 | 15 | 888536 | 5.3756 | 3.82E-08 | 10642 |
| DR-GORD | *IGF2BP2* | 3 | 185361527 | 185542844 | 214 | 35 | 888536 | 5.3737 | 3.86E-08 | 10644 |
| DR-GORD | *SNF8* | 17 | 47006678 | 47022479 | 61 | 7 | 888536 | 5.3672 | 4.00E-08 | 11267 |
| DR-GORD | *KCNK7* | 11 | 65360326 | 65363467 | 3 | 2 | 888536 | 5.3543 | 4.29E-08 | 10089 |
| DR-GORD | *ZNF518B* | 4 | 10441498 | 10459034 | 43 | 13 | 888536 | 5.2019 | 9.86E-08 | 85460 |
| DR-GORD | *AP000769.1* | 11 | 65222728 | 65234028 | 29 | 3 | 888536 | 5.1812 | 1.10E-07 | 101927789 |
| DR-GORD | *KDM2B* | 12 | 121866900 | 122018920 | 277 | 22 | 888536 | 5.1742 | 1.14E-07 | 84678 |
| DR-GORD | *TRAIP* | 3 | 49866034 | 49894007 | 40 | 6 | 888536 | 5.0873 | 1.82E-07 | 10293 |
| DR-GORD | *ZMIZ1* | 10 | 80828792 | 81076276 | 739 | 100 | 888536 | 5.0262 | 2.50E-07 | 57178 |
| DR-GORD | *MAB21L1* | 13 | 36047926 | 36050832 | 2 | 1 | 888536 | 4.9708 | 3.33E-07 | 4081 |
| DR-GORD | *TTLL6* | 17 | 46839597 | 46894576 | 158 | 17 | 888536 | 4.9635 | 3.46E-07 | 284076 |
| DR-GORD | *PLEKHM1* | 17 | 43513266 | 43568115 | 140 | 7 | 888536 | 4.9498 | 3.71E-07 | 9842 |
| DR-GORD | *NSF* | 17 | 44668035 | 44834830 | 78 | 7 | 888536 | 4.9125 | 4.50E-07 | 101930324 |
| DR-GORD | *MAU2* | 19 | 19431490 | 19469563 | 79 | 9 | 888536 | 4.9095 | 4.57E-07 | 23383 |
| DR-GORD | *UBA7* | 3 | 49842640 | 49851379 | 9 | 3 | 888536 | 4.8689 | 5.61E-07 | 100847079 |
| DR-GORD | *KCNQ1* | 11 | 2465914 | 2870339 | 1032 | 126 | 888536 | 4.8617 | 5.82E-07 | 3784 |
| DR-GORD | *GATAD2A* | 19 | 19496635 | 19619740 | 189 | 14 | 888536 | 4.8133 | 7.42E-07 | 54815 |
| DR-GORD | *IP6K1* | 3 | 49761727 | 49823975 | 103 | 10 | 888536 | 4.7866 | 8.48E-07 | 9807 |
| DR-GORD | *ARHGAP27* | 17 | 43471275 | 43511787 | 101 | 6 | 888536 | 4.7553 | 9.91E-07 | 201176 |
| DR-GORD | *ANAPC5* | 12 | 121746048 | 121837699 | 235 | 29 | 888536 | 4.6021 | 2.09E-06 | 51433 |
| DR-GORD | *RBM6* | 3 | 49977440 | 50137478 | 292 | 13 | 888536 | 4.5611 | 2.54E-06 | 10180 |
| DR-IBD | *THADA* | 2 | 43393800 | 43823185 | 810 | 48 | 888536 | 6.659 | 1.38E-11 | 63892 |
| DR-IBD | *CARD9* | 9 | 139256355 | 139268133 | 41 | 11 | 888536 | 6.3854 | 8.55E-11 | 728489 |
| DR-IBD | *CDKAL1* | 6 | 20534688 | 21232635 | 2068 | 75 | 888536 | 6.0278 | 8.31E-10 | 54901 |
| DR-IBD | *SDCCAG3* | 9 | 139296377 | 139305061 | 65 | 11 | 888536 | 5.9053 | 1.76E-09 | 10807 |
| DR-IBD | *DNLZ* | 9 | 139253932 | 139258241 | 18 | 6 | 888536 | 5.7354 | 4.86E-09 | 728489 |
| DR-IBD | *SNAPC4* | 9 | 139270029 | 139293249 | 95 | 13 | 888536 | 5.3976 | 3.38E-08 | 6621 |
| DR-IBD | *GPSM1* | 9 | 139221932 | 139254057 | 100 | 18 | 888536 | 5.2904 | 6.10E-08 | 26086 |
| DR-IBD | *UBE3C* | 7 | 156931607 | 157062066 | 419 | 17 | 888536 | 5.2424 | 7.93E-08 | 9690 |
| DR-IBD | *PMPCA* | 9 | 139305110 | 139318213 | 46 | 9 | 888536 | 5.2114 | 9.37E-08 | 23203 |
| DR-IBD | *TRPS1* | 8 | 116420724 | 116821899 | 485 | 25 | 888536 | 5.1592 | 1.24E-07 | 7227 |
| DR-IBD | *INPP5E* | 9 | 139323071 | 139334274 | 48 | 8 | 888536 | 4.9774 | 3.22E-07 | 56623 |
| DR-IBD | *NKX6-3* | 8 | 41502697 | 41508855 | 22 | 6 | 888536 | 4.6923 | 1.35E-06 | 157848 |
| DR-IBD | *PEAK1* | 15 | 77400471 | 77712486 | 477 | 23 | 888536 | 4.6344 | 1.79E-06 | 79834 |
| DR-IBD | *PIK3C2B* | 1 | 204391756 | 204463852 | 177 | 17 | 888536 | 4.6314 | 1.82E-06 | 5287 |
| DR-PUD | *RASIP1* | 19 | 49223844 | 49243978 | 47 | 7 | 888536 | 7.1414 | 4.62E-13 | 54922 |
| DR-PUD | *MAMSTR* | 19 | 49215999 | 49222978 | 24 | 5 | 888536 | 7.1002 | 6.23E-13 | 284358 |
| DR-PUD | *CDKAL1* | 6 | 20534688 | 21232635 | 2068 | 75 | 888536 | 6.9536 | 1.78E-12 | 54901 |
| DR-PUD | *UBE3C* | 7 | 156931607 | 157062066 | 419 | 17 | 888536 | 6.9148 | 2.34E-12 | 9690 |
| DR-PUD | *FUT2* | 19 | 49199228 | 49209207 | 35 | 4 | 888536 | 6.7338 | 8.27E-12 | 2524 |
| DR-PUD | *LAMC1* | 1 | 182992595 | 183114727 | 473 | 12 | 888536 | 6.1643 | 3.54E-10 | 3915 |
| DR-PUD | *IZUMO1* | 19 | 49244109 | 49250166 | 27 | 2 | 888536 | 5.8178 | 2.98E-09 | 284359 |
| DR-PUD | *THADA* | 2 | 43393800 | 43823185 | 810 | 48 | 888536 | 5.7653 | 4.08E-09 | 63892 |
| DR-PUD | *SGCD* | 5 | 155297354 | 156194799 | 2646 | 94 | 888536 | 5.6659 | 7.31E-09 | 6444 |
| DR-PUD | *FBXL20* | 17 | 37415384 | 37558776 | 311 | 11 | 888536 | 5.426 | 2.88E-08 | 84961 |
| DR-PUD | *KCNQ1* | 11 | 2465914 | 2870339 | 1032 | 126 | 888536 | 5.4008 | 3.32E-08 | 3784 |
| DR-PUD | *MED1* | 17 | 37560538 | 37607539 | 85 | 8 | 888536 | 5.2701 | 6.82E-08 | 5469 |
| DR-PUD | *DMWD* | 19 | 46286205 | 46296060 | 20 | 7 | 888536 | 5.1057 | 1.65E-07 | 1762 |
| DR-PUD | *UBE2E2* | 3 | 23244511 | 23633284 | 1370 | 23 | 888536 | 5.1014 | 1.69E-07 | 7325 |
| DR-PUD | *CDK12* | 17 | 37617764 | 37721160 | 211 | 13 | 888536 | 5.0125 | 2.69E-07 | 51755 |
| DR-PUD | *ZMIZ1* | 10 | 80828792 | 81076276 | 739 | 100 | 888536 | 4.9826 | 3.14E-07 | 57178 |
| DR-PUD | *PROX1* | 1 | 214156524 | 214214595 | 138 | 20 | 888536 | 4.9202 | 4.32E-07 | 5629 |
| DR-PUD | *FBXO46* | 19 | 46213887 | 46234162 | 25 | 10 | 888536 | 4.9039 | 4.70E-07 | 23403 |
| DR-PUD | *SIX5* | 19 | 46268043 | 46272484 | 5 | 2 | 888536 | 4.8346 | 6.67E-07 | 147912 |
| DR-PUD | *AC011530.4* | 19 | 46282695 | 46289231 | 10 | 3 | 888536 | 4.6378 | 1.76E-06 | NA |
| DR-PUD | *DMPK* | 19 | 46272975 | 46285810 | 17 | 4 | 888536 | 4.619 | 1.93E-06 | 1760 |
| DR-IBS | *ZMIZ1* | 10 | 80828792 | 81076276 | 893 | 114 | 918810 | 6.2618 | 1.90E-10 | 57178 |
| DR-IBS | *MPV17* | 2 | 27532360 | 27548547 | 23 | 9 | 918810 | 6.1313 | 4.36E-10 | 4358 |
| DR-IBS | *DNAJC5G* | 2 | 27498289 | 27504367 | 10 | 4 | 918810 | 6.0817 | 5.95E-10 | 285126 |
| DR-IBS | *MACF1* | 1 | 39546988 | 39952849 | 926 | 35 | 918810 | 5.9696 | 1.19E-09 | 23499 |
| DR-IBS | *PPP2R3A* | 3 | 135684515 | 135866733 | 338 | 22 | 918810 | 5.9256 | 1.56E-09 | 5523 |
| DR-IBS | *TRIM54* | 2 | 27505260 | 27530307 | 47 | 9 | 918810 | 5.9255 | 1.56E-09 | 57159 |
| DR-IBS | *GCKR* | 2 | 27719709 | 27746554 | 53 | 11 | 918810 | 5.8993 | 1.83E-09 | 2646 |
| DR-IBS | *SNX17* | 2 | 27593389 | 27599995 | 10 | 3 | 918810 | 5.7354 | 4.86E-09 | 9784 |
| DR-IBS | *PABPC4* | 1 | 40026488 | 40042462 | 39 | 10 | 918810 | 5.626 | 9.22E-09 | 100996696 |
| DR-IBS | *KIAA0754* | 1 | 39876151 | 39882154 | 14 | 6 | 918810 | 5.5205 | 1.69E-08 | 643314 |
| DR-IBS | *AP3S2* | 15 | 90373831 | 90437574 | 140 | 16 | 918810 | 5.4887 | 2.02E-08 | 100526783 |
| DR-IBS | *UBE2E2* | 3 | 23244511 | 23633284 | 1459 | 26 | 918810 | 5.4677 | 2.28E-08 | 7325 |
| DR-IBS | *TMEM106A* | 17 | 41363854 | 41372061 | 10 | 4 | 918810 | 5.4583 | 2.40E-08 | 113277 |
| DR-IBS | *VPS33B* | 15 | 91541646 | 91565833 | 80 | 11 | 918810 | 5.3684 | 3.97E-08 | 26276 |
| DR-IBS | *NBR1* | 17 | 41322498 | 41363708 | 84 | 4 | 918810 | 5.3593 | 4.18E-08 | 4077 |
| DR-IBS | *AC074091.13* | 2 | 27928653 | 27938599 | 18 | 7 | 918810 | 5.3118 | 5.43E-08 | NA |
| DR-IBS | *HMGA2* | 12 | 66217911 | 66360075 | 283 | 38 | 918810 | 5.3021 | 5.73E-08 | 8091 |
| DR-IBS | *TRPS1* | 8 | 116420724 | 116821899 | 780 | 35 | 918810 | 5.2981 | 5.85E-08 | 7227 |
| DR-IBS | *BRCA1* | 17 | 41196312 | 41277500 | 178 | 6 | 918810 | 5.2785 | 6.51E-08 | 672 |
| DR-IBS | *MTMR3* | 22 | 30279144 | 30426855 | 407 | 27 | 918810 | 5.2755 | 6.62E-08 | 8897 |
| DR-IBS | *KRTCAP3* | 2 | 27665233 | 27669348 | 5 | 3 | 918810 | 5.2691 | 6.86E-08 | 200634 |
| DR-IBS | *SLC30A3* | 2 | 27476552 | 27498685 | 44 | 9 | 918810 | 5.2178 | 9.05E-08 | 7781 |
| DR-IBS | *SLCO4C1* | 5 | 101569690 | 101632253 | 265 | 25 | 918810 | 5.1986 | 1.00E-07 | 353189 |
| DR-IBS | *MSL2* | 3 | 135867764 | 135916083 | 65 | 16 | 918810 | 5.1792 | 1.11E-07 | 55167 |
| DR-IBS | *PAM* | 5 | 102089685 | 102366809 | 736 | 30 | 918810 | 5.1755 | 1.14E-07 | 5066 |
| DR-IBS | *NCR3LG1* | 11 | 17373273 | 17398888 | 79 | 15 | 918810 | 5.1734 | 1.15E-07 | 374383 |
| DR-IBS | *GTF3C2* | 2 | 27548716 | 27579868 | 35 | 8 | 918810 | 5.1572 | 1.25E-07 | 2976 |
| DR-IBS | *IFT172* | 2 | 27667238 | 27712656 | 81 | 10 | 918810 | 5.1451 | 1.34E-07 | 26160 |
| DR-IBS | *RND2* | 17 | 41177258 | 41184057 | 11 | 2 | 918810 | 5.1089 | 1.62E-07 | 8153 |
| DR-IBS | *VAT1* | 17 | 41166622 | 41177140 | 14 | 4 | 918810 | 5.0996 | 1.70E-07 | 10493 |
| DR-IBS | *PPM1G* | 2 | 27604061 | 27632554 | 46 | 6 | 918810 | 5.0862 | 1.83E-07 | 5496 |
| DR-IBS | *BMP8A* | 1 | 39957318 | 39991607 | 111 | 22 | 918810 | 5.0649 | 2.04E-07 | 353500 |
| DR-IBS | *FNDC4* | 2 | 27714750 | 27718112 | 6 | 2 | 918810 | 5.0464 | 2.25E-07 | 64838 |
| DR-IBS | *C15orf38-AP3S2* | 15 | 90377540 | 90456114 | 189 | 20 | 918810 | 5.0259 | 2.51E-07 | 100526783 |
| DR-IBS | *HORMAD2* | 22 | 30476163 | 30573064 | 221 | 25 | 918810 | 4.9915 | 3.00E-07 | 150280 |
| DR-IBS | *NRBP1* | 2 | 27650657 | 27665126 | 23 | 3 | 918810 | 4.9118 | 4.51E-07 | 29959 |
| DR-IBS | *GIP* | 17 | 47035916 | 47045958 | 51 | 7 | 918810 | 4.8834 | 5.21E-07 | 2695 |
| DR-IBS | *CAD* | 2 | 27440258 | 27466811 | 81 | 13 | 918810 | 4.8633 | 5.77E-07 | 790 |
| DR-IBS | *DENND1A* | 9 | 126141933 | 126692431 | 1519 | 53 | 918810 | 4.8397 | 6.50E-07 | 57706 |
| DR-IBS | *C15orf38* | 15 | 90443159 | 90456188 | 34 | 12 | 918810 | 4.7727 | 9.09E-07 | 348110 |
| DR-IBS | *EIF2B4* | 2 | 27587219 | 27593353 | 15 | 8 | 918810 | 4.7563 | 9.86E-07 | 8890 |
| DR-IBS | *RREB1* | 6 | 7107830 | 7252213 | 309 | 35 | 918810 | 4.7256 | 1.15E-06 | 6239 |
| DR-IBS | *ATP5G1* | 17 | 46970127 | 46973233 | 7 | 2 | 918810 | 4.714 | 1.21E-06 | 516 |
| DR-IBS | *NUCB2* | 11 | 17229700 | 17371521 | 336 | 20 | 918810 | 4.6764 | 1.46E-06 | 4925 |
| DR-IBS | *UQCR10* | 22 | 30163358 | 30166402 | 9 | 3 | 918810 | 4.6569 | 1.61E-06 | 29796 |
| DR-IBS | *GIN1* | 5 | 102421704 | 102455855 | 118 | 7 | 918810 | 4.6537 | 1.63E-06 | 54826 |
| DR-IBS | *C2orf16* | 2 | 27799389 | 27805588 | 9 | 3 | 918810 | 4.6168 | 1.95E-06 | 84226 |
| DR-DD | *TCF7L2* | 10 | 114710009 | 114927437 | 379 | 82 | 886308 | 8.0379 | 4.57E-16 | 6934 |
| DR-DD | *TRPS1* | 8 | 116420724 | 116821899 | 530 | 25 | 886308 | 7.3467 | 1.02E-13 | 7227 |
| DR-DD | *ARHGAP15* | 2 | 143848931 | 144525921 | 1699 | 91 | 886308 | 6.8154 | 4.70E-12 | 101928361 |
| DR-DD | *CDKAL1* | 6 | 20534688 | 21232635 | 2239 | 83 | 886308 | 6.6111 | 1.91E-11 | 54901 |
| DR-DD | *OBFC1* | 10 | 105642300 | 105677963 | 105 | 11 | 886308 | 6.5868 | 2.25E-11 | 79991 |
| DR-DD | *DENND1A* | 9 | 126141933 | 126692431 | 1415 | 54 | 886308 | 6.237 | 2.23E-10 | 57706 |
| DR-DD | *FIBP* | 11 | 65651212 | 65656010 | 11 | 2 | 886308 | 5.7503 | 4.45E-09 | 9158 |
| DR-DD | *FOSL1* | 11 | 65659520 | 65668044 | 25 | 7 | 886308 | 5.3729 | 3.87E-08 | 8061 |
| DR-DD | *THADA* | 2 | 43393800 | 43823185 | 891 | 52 | 886308 | 5.3632 | 4.09E-08 | 63892 |
| DR-DD | *FAM185A* | 7 | 102389418 | 102449672 | 172 | 21 | 886308 | 5.3422 | 4.59E-08 | 222234 |
| DR-DD | *AGBL2* | 11 | 47681143 | 47736941 | 106 | 13 | 886308 | 5.333 | 4.83E-08 | 79841 |
| DR-DD | *DCLK2* | 4 | 150999426 | 151178609 | 562 | 37 | 886308 | 5.2712 | 6.78E-08 | 166614 |
| DR-DD | *TCF4* | 18 | 52889562 | 53332018 | 753 | 50 | 886308 | 5.2159 | 9.15E-08 | 6925 |
| DR-DD | *MRAS* | 3 | 138066539 | 138124375 | 141 | 13 | 886308 | 5.2138 | 9.25E-08 | 22808 |
| DR-DD | *KCNQ1* | 11 | 2465914 | 2870339 | 1248 | 151 | 886308 | 5.1621 | 1.22E-07 | 3784 |
| DR-DD | *JAZF1* | 7 | 27870192 | 28220362 | 968 | 86 | 886308 | 5.1473 | 1.32E-07 | 221895 |
| DR-DD | *BDNF* | 11 | 27676440 | 27743605 | 128 | 18 | 886308 | 5.1442 | 1.34E-07 | 627 |
| DR-DD | *CDC123* | 10 | 12237964 | 12292588 | 158 | 19 | 886308 | 5.1424 | 1.36E-07 | 8872 |
| DR-DD | *HHIP* | 4 | 145567173 | 145666423 | 184 | 14 | 886308 | 5.0819 | 1.87E-07 | 64399 |
| DR-DD | *NUP160* | 11 | 47799639 | 47870107 | 145 | 15 | 886308 | 4.9389 | 3.93E-07 | 23279 |
| DR-DD | *CELF1* | 11 | 47487496 | 47587121 | 143 | 26 | 886308 | 4.8401 | 6.49E-07 | 10658 |
| DR-DD | *PHGR1* | 15 | 40643234 | 40648635 | 18 | 6 | 886308 | 4.8303 | 6.82E-07 | 644844 |
| DR-DD | *CRIP3* | 6 | 43267448 | 43276535 | 13 | 3 | 886308 | 4.7759 | 8.95E-07 | 401262 |
| DR-DD | *LRBA* | 4 | 151185594 | 151936879 | 1585 | 42 | 886308 | 4.7739 | 9.04E-07 | 987 |
| DR-DD | *OASL* | 12 | 121458095 | 121477045 | 55 | 15 | 886308 | 4.7697 | 9.23E-07 | 8638 |
| DR-DD | *C12orf43* | 12 | 121440225 | 121454305 | 61 | 13 | 886308 | 4.7604 | 9.66E-07 | 64897 |
| DR-DD | *FNBP4* | 11 | 47738072 | 47788995 | 99 | 13 | 886308 | 4.735 | 1.10E-06 | 23360 |
| DR-DD | *HNF1A* | 12 | 121416346 | 121440315 | 104 | 16 | 886308 | 4.6908 | 1.36E-06 | 6927 |
| DR-DD | *CTSW* | 11 | 65647280 | 65651212 | 9 | 4 | 886308 | 4.6751 | 1.47E-06 | 1521 |
| DED-GORD | *BTN2A1* | 6 | 26458150 | 26476849 | 66 | 12 | 854963 | 6.0984 | 5.36E-10 | 11120 |
| DED-GORD | *NCAM1* | 11 | 112831997 | 113149158 | 935 | 54 | 854963 | 5.5422 | 1.49E-08 | 4684 |
| DED-GORD | *CRTC1* | 19 | 18794487 | 18893004 | 260 | 27 | 854963 | 5.0115 | 2.70E-07 | 23373 |
| DED-IBD | *OTUD3* | 1 | 20209006 | 20239438 | 82 | 14 | 854963 | 5.7425 | 4.67E-09 | 23252 |
| DED-IBD | *MST1* | 3 | 49721380 | 49726934 | 6 | 3 | 854963 | 5.7413 | 4.70E-09 | 4485 |
| DED-IBD | *BSN* | 3 | 49591922 | 49708978 | 165 | 15 | 854963 | 5.4672 | 2.29E-08 | 8927 |
| DED-IBD | *TCTA* | 3 | 49449639 | 49453908 | 7 | 3 | 854963 | 5.3367 | 4.73E-08 | 6988 |
| DED-IBD | *GPX1* | 3 | 49394609 | 49396033 | 2 | 1 | 854963 | 5.2769 | 6.57E-08 | 2876 |
| DED-IBD | *NICN1* | 3 | 49460379 | 49466759 | 7 | 4 | 854963 | 5.1764 | 1.13E-07 | 84276 |
| DED-IBD | *APEH* | 3 | 49711435 | 49721396 | 8 | 3 | 854963 | 5.0609 | 2.09E-07 | 327 |
| DED-IBD | *DAG1* | 3 | 49506146 | 49573048 | 115 | 12 | 854963 | 4.9775 | 3.22E-07 | 1605 |
| DED-IBD | *P4HA2* | 5 | 131527531 | 131631008 | 237 | 20 | 854963 | 4.9677 | 3.39E-07 | 101927705 |
| DED-IBD | *IRF5* | 7 | 128577666 | 128590089 | 38 | 10 | 854963 | 4.9565 | 3.59E-07 | 3663 |
| DED-IBD | *RNF123* | 3 | 49726932 | 49758962 | 50 | 8 | 854963 | 4.7606 | 9.65E-07 | 63891 |
| DED-IBD | *RHOA* | 3 | 49396578 | 49450431 | 99 | 10 | 854963 | 4.6503 | 1.66E-06 | 387 |
| DED-IBD | *AMT* | 3 | 49454211 | 49460186 | 9 | 2 | 854963 | 4.5766 | 2.36E-06 | 275 |
| DED-PUD | *MAMSTR* | 19 | 49215999 | 49222978 | 24 | 5 | 854963 | 6.1794 | 3.22E-10 | 284358 |
| DED-PUD | *FUT2* | 19 | 49199228 | 49209207 | 35 | 4 | 854963 | 6.0098 | 9.29E-10 | 2524 |
| DED-PUD | *RASIP1* | 19 | 49223844 | 49243978 | 47 | 7 | 854963 | 5.9514 | 1.33E-09 | 54922 |
| DED-PUD | *MLLT10* | 10 | 21823094 | 22032559 | 185 | 27 | 854963 | 5.087 | 1.82E-07 | 8028 |
| DED-PUD | *IZUMO1* | 19 | 49244109 | 49250166 | 27 | 2 | 854963 | 4.7685 | 9.28E-07 | 284359 |
| DED-PUD | *DNAJC1* | 10 | 22045466 | 22292698 | 401 | 21 | 854963 | 4.6865 | 1.39E-06 | 64215 |
| DED-IBS | *NCAM1* | 11 | 112831997 | 113149158 | 1050 | 56 | 885237 | 6.1769 | 3.27E-10 | 4684 |
| DED-DD | *ARHGAP15* | 2 | 143848931 | 144525921 | 1699 | 91 | 849735 | 7.8635 | 1.87E-15 | 101928361 |
| DED-DD | *MLLT10* | 10 | 21823094 | 22032559 | 202 | 31 | 849735 | 6.1094 | 5.00E-10 | 8028 |
| DED-DD | *CELF1* | 11 | 47487496 | 47587121 | 143 | 26 | 849735 | 5.5728 | 1.25E-08 | 10658 |
| DED-DD | *NDUFS3* | 11 | 47586888 | 47606114 | 19 | 7 | 849735 | 5.257 | 7.32E-08 | 4722 |
| DED-DD | *KBTBD4* | 11 | 47593749 | 47600567 | 7 | 4 | 849735 | 5.2497 | 7.62E-08 | 55709 |
| DED-DD | *FOXP2* | 7 | 113726382 | 114333827 | 975 | 51 | 849735 | 4.9302 | 4.11E-07 | 93986 |
| DED-DD | *CASC10* | 10 | 21781587 | 21786191 | 8 | 3 | 849735 | 4.9179 | 4.37E-07 | 399726 |
| DED-DD | *MKL1* | 22 | 40806285 | 41032706 | 417 | 18 | 849735 | 4.7804 | 8.75E-07 | 57591 |
| DED-DD | *SKIDA1* | 10 | 21802407 | 21814611 | 10 | 4 | 849735 | 4.5686 | 2.46E-06 | 387640 |
| Uveitis-IBD | *IL23R* | 1 | 67632083 | 67725662 | 264 | 25 | 904578 | 5.9088 | 1.72E-09 | 149233 |
| Uveitis-IBD | *C1orf106* | 1 | 200860176 | 200884863 | 72 | 15 | 904578 | 5.2591 | 7.24E-08 | 55765 |
| Uveitis-PUD | *RASIP1* | 19 | 49223844 | 49243978 | 47 | 7 | 904578 | 6.3515 | 1.07E-10 | 54922 |
| Uveitis-PUD | *MAMSTR* | 19 | 49215999 | 49222978 | 24 | 5 | 904578 | 6.2465 | 2.10E-10 | 284358 |
| Uveitis-PUD | *FUT2* | 19 | 49199228 | 49209207 | 35 | 4 | 904578 | 5.5907 | 1.13E-08 | 2524 |
| Uveitis-PUD | *IZUMO1* | 19 | 49244109 | 49250166 | 27 | 2 | 904578 | 5.5042 | 1.85E-08 | 284359 |
| Uveitis-DD | *ARHGAP15* | 2 | 143848931 | 144525921 | 1699 | 91 | 899350 | 6.3819 | 8.75E-11 | 101928361 |
| Myopia-GORD | *BMP4* | 14 | 54416454 | 54425479 | 19 | 6 | 855143 | 6.1641 | 3.54E-10 | 652 |
| Myopia-GORD | *APOC1* | 19 | 45417504 | 45422606 | 11 | 5 | 855143 | 5.1861 | 1.07E-07 | 341 |
| Myopia-GORD | *BICC1* | 10 | 60272900 | 60591195 | 423 | 52 | 855143 | 5.1603 | 1.23E-07 | 80114 |
| Myopia-GORD | *SHISA6* | 17 | 11144580 | 11467380 | 1132 | 104 | 855143 | 5.1211 | 1.52E-07 | 388336 |
| Myopia-GORD | *APOE* | 19 | 45409011 | 45412650 | 6 | 3 | 855143 | 5.1124 | 1.59E-07 | 348 |
| Myopia-GORD | *RBFOX1* | 16 | 6069095 | 7763340 | 9963 | 445 | 855143 | 5.0903 | 1.79E-07 | 54715 |
| Myopia-IBD | *TOX* | 8 | 59717977 | 60031767 | 798 | 62 | 855143 | 6.5544 | 2.79E-11 | 9760 |
| Myopia-IBD | *RBFOX1* | 16 | 6069095 | 7763340 | 9963 | 445 | 855143 | 6.3179 | 1.33E-10 | 54715 |
| Myopia-IBD | *IL23R* | 1 | 67632083 | 67725662 | 264 | 25 | 855143 | 5.92 | 1.61E-09 | 149233 |
| Myopia-IBD | *PCCA* | 13 | 100741269 | 101182686 | 867 | 38 | 855143 | 5.4542 | 2.46E-08 | 5095 |
| Myopia-IBD | *BMP4* | 14 | 54416454 | 54425479 | 19 | 6 | 855143 | 5.359 | 4.18E-08 | 652 |
| Myopia-IBD | *ZNF281* | 1 | 200374068 | 200379184 | 2 | 1 | 855143 | 5.3062 | 5.60E-08 | 23528 |
| Myopia-IBD | *GGACT* | 13 | 101183810 | 101241782 | 99 | 9 | 855143 | 4.9451 | 3.81E-07 | 87769 |
| Myopia-IBD | *C1orf106* | 1 | 200860176 | 200884863 | 72 | 15 | 855143 | 4.8717 | 5.53E-07 | 55765 |
| Myopia-IBD | *ARMS2* | 10 | 124214169 | 124216868 | 17 | 3 | 855143 | 4.7094 | 1.24E-06 | 387715 |
| Myopia-PUD | *GPD2* | 2 | 157291802 | 157470247 | 356 | 20 | 855143 | 5.9924 | 1.03E-09 | 2820 |
| Myopia-PUD | *CNDP2* | 18 | 72163051 | 72188366 | 100 | 14 | 855143 | 5.8753 | 2.11E-09 | 55748 |
| Myopia-PUD | *BMP2* | 20 | 6748311 | 6760927 | 31 | 8 | 855143 | 5.711 | 5.62E-09 | 650 |
| Myopia-PUD | *KCNQ5* | 6 | 73331520 | 73908574 | 1376 | 97 | 855143 | 5.5936 | 1.11E-08 | 56479 |
| Myopia-PUD | *TOX* | 8 | 59717977 | 60031767 | 798 | 62 | 855143 | 5.3551 | 4.28E-08 | 9760 |
| Myopia-PUD | *ZBTB38* | 3 | 141043055 | 141168634 | 262 | 22 | 855143 | 5.0979 | 1.72E-07 | 253461 |
| Myopia-PUD | *FAM150B* | 2 | 279558 | 288851 | 17 | 4 | 855143 | 4.7145 | 1.21E-06 | 285016 |
| Myopia-PUD | *ACP1* | 2 | 264140 | 278283 | 24 | 6 | 855143 | 4.7007 | 1.30E-06 | 52 |
| Myopia-PUD | *FUT2* | 19 | 49199228 | 49209207 | 35 | 4 | 855143 | 4.6937 | 1.34E-06 | 2524 |
| Myopia-IBS | *HTRA1* | 10 | 124221041 | 124274424 | 129 | 20 | 913488 | 6.0353 | 7.93E-10 | 5654 |
| Myopia-IBS | *KCNT2* | 1 | 196194909 | 196578355 | 280 | 37 | 913488 | 5.9861 | 1.07E-09 | 343450 |
| Myopia-IBS | *TRIP10* | 19 | 6737936 | 6751537 | 40 | 13 | 913488 | 5.0166 | 2.63E-07 | 9322 |
| Myopia-IBS | *BTBD16* | 10 | 124030821 | 124097677 | 249 | 25 | 913488 | 5.0073 | 2.76E-07 | 118663 |
| Myopia-DD | *ARHGAP15* | 2 | 143848931 | 144525921 | 1699 | 91 | 849915 | 7.9126 | 1.26E-15 | 101928361 |
| Myopia-DD | *CHRNB1* | 17 | 7348380 | 7361026 | 36 | 7 | 849915 | 7.0692 | 7.79E-13 | 1140 |
| Myopia-DD | *POLR2A* | 17 | 7387685 | 7417933 | 132 | 10 | 849915 | 6.829 | 4.27E-12 | 5430 |
| Myopia-DD | *SLC35G6* | 17 | 7384721 | 7386383 | 7 | 2 | 849915 | 6.6843 | 1.16E-11 | 643664 |
| Myopia-DD | *LOXL1* | 15 | 74218330 | 74244478 | 120 | 16 | 849915 | 6.3221 | 1.29E-10 | 4016 |
| Myopia-DD | *EFEMP1* | 2 | 56093102 | 56151274 | 193 | 26 | 849915 | 6.1669 | 3.48E-10 | 2202 |
| Myopia-DD | *NLGN2* | 17 | 7308193 | 7323179 | 40 | 17 | 849915 | 6.1353 | 4.25E-10 | 57555 |
| Myopia-DD | *ZBTB4* | 17 | 7362685 | 7387582 | 69 | 10 | 849915 | 6.1094 | 5.00E-10 | 57659 |
| Myopia-DD | *BICC1* | 10 | 60272900 | 60591195 | 512 | 58 | 849915 | 5.9721 | 1.17E-09 | 80114 |
| Myopia-DD | *AFAP1* | 4 | 7760441 | 7941653 | 1025 | 41 | 849915 | 5.9113 | 1.70E-09 | 60312 |
| Myopia-DD | *C17orf74* | 17 | 7328934 | 7330887 | 3 | 2 | 849915 | 5.7003 | 5.98E-09 | 201243 |
| Myopia-DD | *CD55* | 1 | 207494853 | 207534311 | 47 | 12 | 849915 | 5.6825 | 6.64E-09 | 1604 |
| Myopia-DD | *PCBP3* | 21 | 47063608 | 47362368 | 801 | 21 | 849915 | 5.5081 | 1.81E-08 | 54039 |
| Myopia-DD | *CWC27* | 5 | 64064757 | 64314590 | 507 | 18 | 849915 | 5.4726 | 2.22E-08 | 10283 |
| Myopia-DD | *ANTXR2* | 4 | 80822303 | 81046608 | 592 | 36 | 849915 | 5.4722 | 2.22E-08 | 118429 |
| Myopia-DD | *GPR158* | 10 | 25463991 | 25891155 | 1871 | 54 | 849915 | 5.362 | 4.11E-08 | 57512 |
| Myopia-DD | *CACNB2* | 10 | 18429606 | 18830798 | 1688 | 112 | 849915 | 5.0136 | 2.67E-07 | 783 |
| Myopia-DD | *COL6A1* | 21 | 47401651 | 47424964 | 111 | 20 | 849915 | 5.0096 | 2.73E-07 | 1291 |
| Myopia-DD | *PRED60* | 21 | 47183565 | 47190005 | 10 | 3 | 849915 | 4.9976 | 2.90E-07 | NA |
| Myopia-DD | *FGF11* | 17 | 7341592 | 7348256 | 12 | 4 | 849915 | 4.9368 | 3.97E-07 | 2256 |
| Myopia-DD | *KCNMA1* | 10 | 78629359 | 79398353 | 2233 | 149 | 849915 | 4.9358 | 3.99E-07 | 3778 |
| Myopia-DD | *S100A10* | 1 | 151955391 | 151966866 | 19 | 5 | 849915 | 4.8931 | 4.96E-07 | 6281 |
| Cataractaract-GORD | *RBM6* | 3 | 49977440 | 50137478 | 292 | 13 | 860413 | 5.7988 | 3.34E-09 | 10180 |
| Cataract-GORD | *RBM5* | 3 | 50126341 | 50156454 | 31 | 7 | 860413 | 5.7693 | 3.98E-09 | 10181 |
| Cataract-GORD | *MON1A* | 3 | 49946302 | 49967606 | 27 | 8 | 860413 | 5.7625 | 4.15E-09 | 84315 |
| Cataract-GORD | *BTN2A1* | 6 | 26458150 | 26476849 | 66 | 12 | 860413 | 5.6364 | 8.68E-09 | 11120 |
| Cataract-GORD | *GABRB1* | 4 | 46995740 | 47428461 | 1101 | 68 | 860413 | 5.5898 | 1.14E-08 | 2560 |
| Cataract-GORD | *CAMKV* | 3 | 49895421 | 49907655 | 19 | 5 | 860413 | 5.5578 | 1.37E-08 | 79012 |
| Cataract-GORD | *MST1R* | 3 | 49924435 | 49941299 | 26 | 5 | 860413 | 5.5194 | 1.70E-08 | 4486 |
| Cataract-GORD | *SEMA3F* | 3 | 50192478 | 50226508 | 63 | 10 | 860413 | 5.4239 | 2.92E-08 | 6405 |
| Cataract-GORD | *CTD-2330K9.3* | 3 | 49941278 | 49954370 | 14 | 4 | 860413 | 4.8251 | 7.00E-07 | NA |
| Cataract-GORD | *BTN1A1* | 6 | 26501449 | 26510650 | 21 | 4 | 860413 | 4.7465 | 1.04E-06 | 696 |
| Cataract-GORD | *TRAIP* | 3 | 49866034 | 49894007 | 40 | 6 | 860413 | 4.6372 | 1.77E-06 | 10293 |
| Cataract-IBD | *MST1* | 3 | 49721380 | 49726934 | 6 | 3 | 860413 | 5.5622 | 1.33E-08 | 4485 |
| Cataract-IBD | *BSN* | 3 | 49591922 | 49708978 | 165 | 15 | 860413 | 5.4192 | 2.99E-08 | 8927 |
| Cataract-IBD | *TCTA* | 3 | 49449639 | 49453908 | 7 | 3 | 860413 | 5.1571 | 1.25E-07 | 6988 |
| Cataract-IBD | *IL23R* | 1 | 67632083 | 67725662 | 264 | 25 | 860413 | 5.1411 | 1.37E-07 | 149233 |
| Cataract-IBD | *RNF123* | 3 | 49726932 | 49758962 | 50 | 8 | 860413 | 5.0265 | 2.50E-07 | 63891 |
| Cataract-IBD | *FCGR2A* | 1 | 161475220 | 161493803 | 50 | 8 | 860413 | 4.9477 | 3.75E-07 | 2212 |
| Cataract-IBD | *DLD* | 7 | 107531415 | 107572175 | 75 | 7 | 860413 | 4.8999 | 4.79E-07 | 1738 |
| Cataract-IBD | *APEH* | 3 | 49711435 | 49721396 | 8 | 3 | 860413 | 4.8201 | 7.17E-07 | 327 |
| Cataract-IBD | *GPX1* | 3 | 49394609 | 49396033 | 2 | 1 | 860413 | 4.7765 | 8.92E-07 | 2876 |
| Cataract-IBD | *USP4* | 3 | 49315264 | 49378145 | 75 | 10 | 860413 | 4.7413 | 1.06E-06 | 7375 |
| Cataract-IBD | *RHOA* | 3 | 49396578 | 49450431 | 99 | 10 | 860413 | 4.6893 | 1.37E-06 | 387 |
| Cataract-IBD | *DAG1* | 3 | 49506146 | 49573048 | 115 | 12 | 860413 | 4.6579 | 1.60E-06 | 1605 |
| Cataract-IBD | *NICN1* | 3 | 49460379 | 49466759 | 7 | 4 | 860413 | 4.6063 | 2.05E-06 | 84276 |
| Cataract-IBS | *MACF1* | 1 | 39546988 | 39952849 | 926 | 35 | 890687 | 4.981 | 3.16E-07 | 23499 |
| Cataract-IBS | *NCAM1* | 11 | 112831997 | 113149158 | 1050 | 56 | 890687 | 4.7406 | 1.07E-06 | 4684 |
| Cataract-IBS | *PABPC4* | 1 | 40026488 | 40042462 | 39 | 10 | 890687 | 4.6475 | 1.68E-06 | 100996696 |
| Cataract-IBS | *GLRB* | 4 | 157997209 | 158093242 | 356 | 25 | 890687 | 4.5883 | 2.23E-06 | 2743 |
| Cataract-DD | *ARHGAP15* | 2 | 143848931 | 144525921 | 1699 | 91 | 855185 | 7.3143 | 1.29E-13 | 101928361 |
| Cataract-DD | *OBFC1* | 10 | 105642300 | 105677963 | 105 | 11 | 855185 | 5.6219 | 9.45E-09 | 79991 |
| Cataract-DD | *BDNF* | 11 | 27676440 | 27743605 | 128 | 18 | 855185 | 5.2768 | 6.57E-08 | 627 |
| PACG-IBD | *OTUD3* | 1 | 20209006 | 20239438 | 82 | 14 | 903291 | 6.0727 | 6.29E-10 | 23252 |
| PACG-IBD | *IRF5* | 7 | 128577666 | 128590089 | 38 | 10 | 903291 | 5.2767 | 6.58E-08 | 3663 |
| PACG-IBS | *HERC2* | 15 | 28356186 | 28567298 | 345 | 14 | 933565 | 5.3312 | 4.88E-08 | 101930157 |
| PACG-DD | *ARHGAP15* | 2 | 143848931 | 144525921 | 1699 | 91 | 898063 | 7.1411 | 4.63E-13 | 101928361 |
| PACG-DD | *SLC25A28* | 10 | 101370282 | 101380366 | 21 | 3 | 898063 | 5.8366 | 2.66E-09 | 81894 |
| PACG-DD | *COX15* | 10 | 101471601 | 101491857 | 36 | 6 | 898063 | 5.7949 | 3.42E-09 | 1355 |
| PACG-DD | *CUTC* | 10 | 101462315 | 101515891 | 94 | 17 | 898063 | 5.5502 | 1.43E-08 | 51076 |
| PACG-DD | *ENTPD7* | 10 | 101419263 | 101465997 | 97 | 10 | 898063 | 5.5111 | 1.78E-08 | 57089 |
| PACG-DD | *HERC2* | 15 | 28356186 | 28567298 | 182 | 10 | 898063 | 5.4532 | 2.47E-08 | 101930157 |
| PACG-DD | *BCAS3* | 17 | 58754814 | 59470199 | 1147 | 55 | 898063 | 5.3644 | 4.06E-08 | 54828 |
| PACG-DD | *MED12L* | 3 | 150803484 | 151154860 | 1030 | 48 | 898063 | 5.221 | 8.90E-08 | 116931 |
| PACG-DD | *EFEMP1* | 2 | 56093102 | 56151274 | 195 | 26 | 898063 | 4.7618 | 9.59E-07 | 2202 |
| PACG-DD | *LOXL1* | 15 | 74218330 | 74244478 | 120 | 16 | 898063 | 4.7559 | 9.88E-07 | 4016 |
| PACG-DD | *P2RY14* | 3 | 150929905 | 150996255 | 193 | 21 | 898063 | 4.7466 | 1.03E-06 | 9934 |
| Keratitis-IBD | *IL23R* | 1 | 67632083 | 67725662 | 264 | 25 | 891672 | 5.2033 | 9.79E-08 | 149233 |
| Keratitis-IBD | *DLD* | 7 | 107531415 | 107572175 | 75 | 7 | 891672 | 4.8264 | 6.95E-07 | 1738 |
| Keratitis-IBS | *ERI3* | 1 | 44686742 | 44820932 | 260 | 22 | 921946 | 5.6026 | 1.06E-08 | 79033 |
| Keratitis-IBS | *DMAP1* | 1 | 44679127 | 44686353 | 13 | 5 | 921946 | 4.637 | 1.77E-06 | 55929 |
| Keratitis-IBS | *KLF17* | 1 | 44584522 | 44600812 | 64 | 16 | 921946 | 4.5923 | 2.19E-06 | 128209 |
| Keratitis-DD | *ARHGAP15* | 2 | 143848931 | 144525921 | 1699 | 91 | 886444 | 8.0214 | 5.23E-16 | 101928361 |
| Keratitis-DD | *S100A10* | 1 | 151955391 | 151966866 | 19 | 5 | 886444 | 6.2111 | 2.63E-10 | 6281 |
| Keratitis-DD | *SLC25A28* | 10 | 101370282 | 101380366 | 21 | 3 | 886444 | 5.6771 | 6.85E-09 | 81894 |
| Keratitis-DD | *ENTPD7* | 10 | 101419263 | 101465997 | 97 | 10 | 886444 | 5.4727 | 2.22E-08 | 57089 |
| Keratitis-DD | *COX15* | 10 | 101471601 | 101491857 | 36 | 6 | 886444 | 5.3911 | 3.50E-08 | 1355 |
| Keratitis-DD | *P2RY12* | 3 | 151055168 | 151102600 | 164 | 23 | 886444 | 5.3686 | 3.97E-08 | 64805 |
| Keratitis-DD | *CUTC* | 10 | 101462315 | 101515891 | 94 | 17 | 886444 | 5.2483 | 7.68E-08 | 51076 |
| Keratitis-DD | *MED12L* | 3 | 150803484 | 151154860 | 1030 | 48 | 886444 | 5.1014 | 1.69E-07 | 116931 |
| Keratitis-DD | *GPR158* | 10 | 25463991 | 25891155 | 1871 | 54 | 886444 | 4.934 | 4.03E-07 | 57512 |

**Table S9**. Significant enriched GO and KEGG pathways across the 40 trait pairs

| **Trait pairs** | **ONTOLOGY** | **ID** | **Description** | **FoldEnrichment** | **pvalue** | **p.adjust** | **Count** |
| --- | --- | --- | --- | --- | --- | --- | --- |
| AMD-IBD | CC | GO:0042611 | MHC protein complex | 51.18 | 4.00E-08 | 6.56E-06 | 5 |
| AMD-IBD | MF | GO:0032395 | MHC class II receptor activity | 68.55 | 9.48E-06 | 8.22E-04 | 3 |
| AMD-IBS | BP | GO:0002396 | MHC protein complex assembly | 78.62 | 1.75E-07 | 1.29E-04 | 4 |
| AMD-IBS | BP | GO:0002501 | peptide antigen assembly with MHC protein complex | 78.62 | 1.75E-07 | 1.29E-04 | 4 |
| AMD-IBS | BP | GO:0002399 | MHC class II protein complex assembly | 77.39 | 7.29E-06 | 1.54E-03 | 3 |
| AMD-IBS | BP | GO:0002503 | peptide antigen assembly with MHC class II protein complex | 77.39 | 7.29E-06 | 1.54E-03 | 3 |
| AMD-IBS | BP | GO:0002486 | antigen processing and presentation of endogenous peptide antigen via MHC class I via ER pathway, TAP-independent | 51.59 | 6.74E-04 | 2.63E-02 | 2 |
| AMD-IBS | BP | GO:0016045 | detection of bacterium | 51.59 | 6.74E-04 | 2.63E-02 | 2 |
| AMD-IBS | CC | GO:0042611 | MHC protein complex | 83.17 | 3.33E-09 | 3.96E-07 | 5 |
| AMD-IBS | CC | GO:0098553 | lumenal side of endoplasmic reticulum membrane | 59.40 | 2.00E-08 | 1.19E-06 | 5 |
| AMD-IBS | CC | GO:0042613 | MHC class II protein complex | 73.38 | 8.67E-06 | 1.68E-04 | 3 |
| AMD-IBS | MF | GO:0032395 | MHC class II receptor activity | 114.72 | 1.99E-06 | 1.69E-04 | 3 |
| Cataract-GORD | BP | GO:0002486 | antigen processing and presentation of endogenous peptide antigen via MHC class I via ER pathway, TAP-independent | 87.90 | 2.31E-04 | 4.29E-02 | 2 |
| Cataract-GORD | BP | GO:0002476 | antigen processing and presentation of endogenous peptide antigen via MHC class Ib | 82.73 | 2.61E-04 | 4.29E-02 | 2 |
| Cataract-GORD | BP | GO:0002484 | antigen processing and presentation of endogenous peptide antigen via MHC class I via ER pathway | 82.73 | 2.61E-04 | 4.29E-02 | 2 |
| Cataract-GORD | BP | GO:0002428 | antigen processing and presentation of peptide antigen via MHC class Ib | 78.13 | 2.94E-04 | 4.29E-02 | 2 |
| Cataract-GORD | BP | GO:0019885 | antigen processing and presentation of endogenous peptide antigen via MHC class I | 61.15 | 4.84E-04 | 4.78E-02 | 2 |
| Cataract-GORD | BP | GO:0002475 | antigen processing and presentation via MHC class Ib | 56.25 | 5.73E-04 | 4.78E-02 | 2 |
| Cataract-GORD | BP | GO:0002483 | antigen processing and presentation of endogenous peptide antigen | 56.25 | 5.73E-04 | 4.78E-02 | 2 |
| Cataract-GORD | CC | GO:0042611 | MHC protein complex | 59.14 | 5.19E-04 | 1.37E-02 | 2 |
| Cataract-IBS | BP | GO:0002486 | antigen processing and presentation of endogenous peptide antigen via MHC class I via ER pathway, TAP-independent | 182.56 | 5.17E-05 | 5.10E-03 | 2 |
| Cataract-IBS | BP | GO:0002476 | antigen processing and presentation of endogenous peptide antigen via MHC class Ib | 171.82 | 5.85E-05 | 5.10E-03 | 2 |
| Cataract-IBS | BP | GO:0002484 | antigen processing and presentation of endogenous peptide antigen via MHC class I via ER pathway | 171.82 | 5.85E-05 | 5.10E-03 | 2 |
| Cataract-IBS | BP | GO:0002428 | antigen processing and presentation of peptide antigen via MHC class Ib | 162.27 | 6.58E-05 | 5.10E-03 | 2 |
| Cataract-IBS | BP | GO:0019885 | antigen processing and presentation of endogenous peptide antigen via MHC class I | 127.00 | 1.09E-04 | 5.70E-03 | 2 |
| Cataract-IBS | BP | GO:0002475 | antigen processing and presentation via MHC class Ib | 116.84 | 1.29E-04 | 5.70E-03 | 2 |
| Cataract-IBS | BP | GO:0002483 | antigen processing and presentation of endogenous peptide antigen | 116.84 | 1.29E-04 | 5.70E-03 | 2 |
| Cataract-IBS | BP | GO:0019883 | antigen processing and presentation of endogenous antigen | 91.28 | 2.12E-04 | 8.22E-03 | 2 |
| Cataract-IBS | BP | GO:0002474 | antigen processing and presentation of peptide antigen via MHC class I | 78.94 | 2.84E-04 | 9.79E-03 | 2 |
| Cataract-IBS | BP | GO:0001916 | positive regulation of T cell mediated cytotoxicity | 71.24 | 3.50E-04 | 1.08E-02 | 2 |
| Cataract-IBS | BP | GO:0001914 | regulation of T cell mediated cytotoxicity | 57.27 | 5.41E-04 | 1.34E-02 | 2 |
| Cataract-IBS | BP | GO:0016045 | detection of bacterium | 91.28 | 1.09E-02 | 4.92E-02 | 1 |
| Cataract-IBS | CC | GO:0042611 | MHC protein complex | 122.83 | 1.16E-04 | 3.79E-03 | 2 |
| Cataract-IBS | CC | GO:0098553 | lumenal side of endoplasmic reticulum membrane | 87.74 | 2.30E-04 | 4.99E-03 | 2 |
| Cataract-IBS | CC | GO:0098576 | lumenal side of membrane | 66.76 | 3.99E-04 | 6.48E-03 | 2 |
| Cataract-IBS | MF | GO:0042605 | peptide antigen binding | 66.44 | 4.00E-04 | 1.36E-02 | 2 |
| DR-DD | BP | GO:0002396 | MHC protein complex assembly | 66.48 | 9.88E-09 | 7.89E-06 | 5 |
| DR-DD | BP | GO:0002501 | peptide antigen assembly with MHC protein complex | 66.48 | 9.88E-09 | 7.89E-06 | 5 |
| DR-DD | BP | GO:0002399 | MHC class II protein complex assembly | 69.80 | 2.65E-07 | 8.47E-05 | 4 |
| DR-DD | BP | GO:0002503 | peptide antigen assembly with MHC class II protein complex | 69.80 | 2.65E-07 | 8.47E-05 | 4 |
| DR-DD | BP | GO:0016045 | detection of bacterium | 52.35 | 2.38E-05 | 3.17E-03 | 3 |
| DR-DD | CC | GO:0042611 | MHC protein complex | 64.74 | 3.54E-10 | 3.24E-08 | 6 |
| DR-DD | CC | GO:0042613 | MHC class II protein complex | 63.47 | 3.99E-07 | 1.46E-05 | 4 |
| DR-DD | MF | GO:0032395 | MHC class II receptor activity | 75.96 | 6.96E-06 | 5.24E-04 | 3 |
| DR-GORD | BP | GO:0002399 | MHC class II protein complex assembly | 52.74 | 8.23E-07 | 1.55E-04 | 4 |
| DR-GORD | BP | GO:0002503 | peptide antigen assembly with MHC class II protein complex | 52.74 | 8.23E-07 | 1.55E-04 | 4 |
| DR-GORD | CC | GO:0042611 | MHC protein complex | 52.64 | 1.25E-09 | 1.78E-07 | 6 |
| DR-GORD | CC | GO:0042613 | MHC class II protein complex | 51.61 | 9.19E-07 | 2.42E-05 | 4 |
| DR-GORD | MF | GO:0032395 | MHC class II receptor activity | 85.17 | 9.34E-08 | 1.34E-05 | 4 |
| DR-IBD | BP | GO:0002483 | antigen processing and presentation of endogenous peptide antigen | 76.91 | 6.38E-14 | 3.35E-11 | 8 |
| DR-IBD | BP | GO:0002478 | antigen processing and presentation of exogenous peptide antigen | 52.76 | 7.35E-14 | 3.35E-11 | 9 |
| DR-IBD | BP | GO:0019883 | antigen processing and presentation of endogenous antigen | 60.08 | 6.06E-13 | 1.84E-10 | 8 |
| DR-IBD | BP | GO:0002396 | MHC protein complex assembly | 80.11 | 1.83E-12 | 4.16E-10 | 7 |
| DR-IBD | BP | GO:0002501 | peptide antigen assembly with MHC protein complex | 80.11 | 1.83E-12 | 4.16E-10 | 7 |
| DR-IBD | BP | GO:0002399 | MHC class II protein complex assembly | 90.12 | 3.31E-11 | 4.48E-09 | 6 |
| DR-IBD | BP | GO:0002503 | peptide antigen assembly with MHC class II protein complex | 90.12 | 3.31E-11 | 4.48E-09 | 6 |
| DR-IBD | BP | GO:0002476 | antigen processing and presentation of endogenous peptide antigen via MHC class Ib | 84.82 | 5.10E-11 | 5.81E-09 | 6 |
| DR-IBD | BP | GO:0002484 | antigen processing and presentation of endogenous peptide antigen via MHC class I via ER pathway | 84.82 | 5.10E-11 | 5.81E-09 | 6 |
| DR-IBD | BP | GO:0002428 | antigen processing and presentation of peptide antigen via MHC class Ib | 80.11 | 7.63E-11 | 8.17E-09 | 6 |
| DR-IBD | BP | GO:0019885 | antigen processing and presentation of endogenous peptide antigen via MHC class I | 62.69 | 4.08E-10 | 3.72E-08 | 6 |
| DR-IBD | BP | GO:0002475 | antigen processing and presentation via MHC class Ib | 57.68 | 7.11E-10 | 6.17E-08 | 6 |
| DR-IBD | BP | GO:0002486 | antigen processing and presentation of endogenous peptide antigen via MHC class I via ER pathway, TAP-independent | 75.10 | 4.62E-09 | 3.24E-07 | 5 |
| DR-IBD | CC | GO:0042611 | MHC protein complex | 87.63 | 4.11E-16 | 8.13E-14 | 9 |
| DR-IBD | CC | GO:0098553 | lumenal side of endoplasmic reticulum membrane | 55.64 | 1.23E-12 | 8.12E-11 | 8 |
| DR-IBD | CC | GO:0042613 | MHC class II protein complex | 85.91 | 4.76E-11 | 2.36E-09 | 6 |
| DR-IBD | MF | GO:0042605 | peptide antigen binding | 56.95 | 1.34E-18 | 3.06E-16 | 12 |
| DR-IBD | MF | GO:0032395 | MHC class II receptor activity | 111.53 | 3.97E-10 | 3.03E-08 | 5 |
| DR-IBS | BP | GO:0002396 | MHC protein complex assembly | 91.43 | 2.02E-16 | 2.03E-13 | 9 |
| DR-IBS | BP | GO:0002501 | peptide antigen assembly with MHC protein complex | 91.43 | 2.02E-16 | 2.03E-13 | 9 |
| DR-IBS | BP | GO:0002399 | MHC class II protein complex assembly | 106.66 | 2.11E-15 | 8.46E-13 | 8 |
| DR-IBS | BP | GO:0002503 | peptide antigen assembly with MHC class II protein complex | 106.66 | 2.11E-15 | 8.46E-13 | 8 |
| DR-IBS | BP | GO:0019886 | antigen processing and presentation of exogenous peptide antigen via MHC class II | 55.05 | 1.22E-12 | 3.06E-10 | 8 |
| DR-IBS | BP | GO:0002483 | antigen processing and presentation of endogenous peptide antigen | 51.20 | 1.47E-09 | 2.46E-07 | 6 |
| DR-IBS | BP | GO:0002486 | antigen processing and presentation of endogenous peptide antigen via MHC class I via ER pathway, TAP-independent | 53.33 | 7.86E-07 | 4.64E-05 | 4 |
| DR-IBS | BP | GO:0002476 | antigen processing and presentation of endogenous peptide antigen via MHC class Ib | 50.19 | 1.02E-06 | 5.71E-05 | 4 |
| DR-IBS | BP | GO:0002484 | antigen processing and presentation of endogenous peptide antigen via MHC class I via ER pathway | 50.19 | 1.02E-06 | 5.71E-05 | 4 |
| DR-IBS | CC | GO:0042611 | MHC protein complex | 97.58 | 3.52E-20 | 8.98E-18 | 11 |
| DR-IBS | CC | GO:0098576 | lumenal side of membrane | 53.03 | 9.76E-17 | 1.24E-14 | 11 |
| DR-IBS | CC | GO:0098553 | lumenal side of endoplasmic reticulum membrane | 63.37 | 3.47E-16 | 2.95E-14 | 10 |
| DR-IBS | CC | GO:0042613 | MHC class II protein complex | 104.37 | 2.92E-15 | 1.86E-13 | 8 |
| DR-IBS | MF | GO:0042605 | peptide antigen binding | 54.99 | 2.09E-18 | 5.64E-16 | 12 |
| DR-IBS | MF | GO:0032395 | MHC class II receptor activity | 150.76 | 4.32E-15 | 5.83E-13 | 7 |
| DR-IBS | MF | GO:0023026 | MHC class II protein complex binding | 63.81 | 3.21E-13 | 2.89E-11 | 8 |
| Myopia-GORD | BP | GO:0002486 | antigen processing and presentation of endogenous peptide antigen via MHC class I via ER pathway, TAP-independent | 124.91 | 1.13E-04 | 1.19E-02 | 2 |
| Myopia-GORD | BP | GO:0002476 | antigen processing and presentation of endogenous peptide antigen via MHC class Ib | 117.56 | 1.28E-04 | 1.19E-02 | 2 |
| Myopia-GORD | BP | GO:0002484 | antigen processing and presentation of endogenous peptide antigen via MHC class I via ER pathway | 117.56 | 1.28E-04 | 1.19E-02 | 2 |
| Myopia-GORD | BP | GO:0002428 | antigen processing and presentation of peptide antigen via MHC class Ib | 111.03 | 1.44E-04 | 1.19E-02 | 2 |
| Myopia-GORD | BP | GO:0019885 | antigen processing and presentation of endogenous peptide antigen via MHC class I | 86.89 | 2.37E-04 | 1.77E-02 | 2 |
| Myopia-GORD | BP | GO:0002475 | antigen processing and presentation via MHC class Ib | 79.94 | 2.81E-04 | 1.77E-02 | 2 |
| Myopia-GORD | BP | GO:0002483 | antigen processing and presentation of endogenous peptide antigen | 79.94 | 2.81E-04 | 1.77E-02 | 2 |
| Myopia-GORD | BP | GO:0019883 | antigen processing and presentation of endogenous antigen | 62.45 | 4.62E-04 | 2.04E-02 | 2 |
| Myopia-GORD | BP | GO:0002474 | antigen processing and presentation of peptide antigen via MHC class I | 54.01 | 6.19E-04 | 2.39E-02 | 2 |
| Myopia-GORD | CC | GO:0042611 | MHC protein complex | 79.84 | 2.82E-04 | 5.61E-03 | 2 |
| Myopia-GORD | CC | GO:0098553 | lumenal side of endoplasmic reticulum membrane | 57.03 | 5.56E-04 | 6.74E-03 | 2 |
| Myopia-IBS | BP | GO:0002396 | MHC protein complex assembly | 78.62 | 1.75E-07 | 1.29E-04 | 4 |
| Myopia-IBS | BP | GO:0002501 | peptide antigen assembly with MHC protein complex | 78.62 | 1.75E-07 | 1.29E-04 | 4 |
| Myopia-IBS | BP | GO:0002399 | MHC class II protein complex assembly | 77.39 | 7.29E-06 | 1.54E-03 | 3 |
| Myopia-IBS | BP | GO:0002503 | peptide antigen assembly with MHC class II protein complex | 77.39 | 7.29E-06 | 1.54E-03 | 3 |
| Myopia-IBS | BP | GO:0002486 | antigen processing and presentation of endogenous peptide antigen via MHC class I via ER pathway, TAP-independent | 51.59 | 6.74E-04 | 2.63E-02 | 2 |
| Myopia-IBS | BP | GO:0016045 | detection of bacterium | 51.59 | 6.74E-04 | 2.63E-02 | 2 |
| Myopia-IBS | CC | GO:0042611 | MHC protein complex | 83.17 | 3.33E-09 | 3.96E-07 | 5 |
| Myopia-IBS | CC | GO:0098553 | lumenal side of endoplasmic reticulum membrane | 59.40 | 2.00E-08 | 1.19E-06 | 5 |
| Myopia-IBS | CC | GO:0042613 | MHC class II protein complex | 73.38 | 8.67E-06 | 1.68E-04 | 3 |
| Myopia-IBS | MF | GO:0032395 | MHC class II receptor activity | 114.72 | 1.99E-06 | 1.69E-04 | 3 |
| PACG-GORD | BP | GO:0002486 | antigen processing and presentation of endogenous peptide antigen via MHC class I via ER pathway, TAP-independent | 197.77 | 5.05E-03 | 3.39E-02 | 1 |
| PACG-GORD | BP | GO:0002476 | antigen processing and presentation of endogenous peptide antigen via MHC class Ib | 186.14 | 5.36E-03 | 3.39E-02 | 1 |
| PACG-GORD | BP | GO:0002484 | antigen processing and presentation of endogenous peptide antigen via MHC class I via ER pathway | 186.14 | 5.36E-03 | 3.39E-02 | 1 |
| PACG-GORD | BP | GO:0002428 | antigen processing and presentation of peptide antigen via MHC class Ib | 175.80 | 5.68E-03 | 3.39E-02 | 1 |
| PACG-GORD | BP | GO:0019885 | antigen processing and presentation of endogenous peptide antigen via MHC class I | 137.58 | 7.25E-03 | 3.39E-02 | 1 |
| PACG-GORD | BP | GO:0002475 | antigen processing and presentation via MHC class Ib | 126.57 | 7.88E-03 | 3.39E-02 | 1 |
| PACG-GORD | BP | GO:0002483 | antigen processing and presentation of endogenous peptide antigen | 126.57 | 7.88E-03 | 3.39E-02 | 1 |
| PACG-GORD | BP | GO:0019883 | antigen processing and presentation of endogenous antigen | 98.89 | 1.01E-02 | 3.42E-02 | 1 |
| PACG-GORD | BP | GO:0002474 | antigen processing and presentation of peptide antigen via MHC class I | 85.52 | 1.16E-02 | 3.83E-02 | 1 |
| PACG-GORD | BP | GO:0001916 | positive regulation of T cell mediated cytotoxicity | 77.18 | 1.29E-02 | 3.89E-02 | 1 |
| PACG-GORD | BP | GO:0001914 | regulation of T cell mediated cytotoxicity | 62.05 | 1.60E-02 | 4.08E-02 | 1 |
| PACG-GORD | BP | GO:0001913 | T cell mediated cytotoxicity | 51.87 | 1.91E-02 | 4.54E-02 | 1 |
| PACG-GORD | CC | GO:0042611 | MHC protein complex | 133.07 | 7.49E-03 | 4.75E-02 | 1 |
| PACG-GORD | MF | GO:0042605 | peptide antigen binding | 66.44 | 1.50E-02 | 3.94E-02 | 1 |
| PACG-IBD | BP | GO:0002399 | MHC class II protein complex assembly | 323.63 | 8.07E-08 | 1.73E-05 | 3 |
| PACG-IBD | BP | GO:0002503 | peptide antigen assembly with MHC class II protein complex | 323.63 | 8.07E-08 | 1.73E-05 | 3 |
| PACG-IBD | BP | GO:0002396 | MHC protein complex assembly | 246.57 | 1.91E-07 | 2.06E-05 | 3 |
| PACG-IBD | BP | GO:0002501 | peptide antigen assembly with MHC protein complex | 246.57 | 1.91E-07 | 2.06E-05 | 3 |
| PACG-IBD | BP | GO:0019886 | antigen processing and presentation of exogenous peptide antigen via MHC class II | 167.03 | 6.45E-07 | 5.54E-05 | 3 |
| PACG-IBD | BP | GO:0002495 | antigen processing and presentation of peptide antigen via MHC class II | 147.94 | 9.37E-07 | 6.72E-05 | 3 |
| PACG-IBD | BP | GO:0002504 | antigen processing and presentation of peptide or polysaccharide antigen via MHC class II | 139.95 | 1.11E-06 | 6.83E-05 | 3 |
| PACG-IBD | BP | GO:0002478 | antigen processing and presentation of exogenous peptide antigen | 126.29 | 1.52E-06 | 8.19E-05 | 3 |
| PACG-IBD | BP | GO:0019884 | antigen processing and presentation of exogenous antigen | 103.56 | 2.79E-06 | 1.33E-04 | 3 |
| PACG-IBD | BP | GO:0048002 | antigen processing and presentation of peptide antigen | 71.92 | 8.44E-06 | 3.63E-04 | 3 |
| PACG-IBD | BP | GO:0016045 | detection of bacterium | 107.88 | 9.23E-03 | 4.38E-02 | 1 |
| PACG-IBD | BP | GO:0002483 | antigen processing and presentation of endogenous peptide antigen | 69.04 | 1.44E-02 | 4.84E-02 | 1 |
| PACG-IBD | CC | GO:0042613 | MHC class II protein complex | 320.21 | 8.43E-08 | 3.71E-06 | 3 |
| PACG-IBD | CC | GO:0042611 | MHC protein complex | 217.75 | 2.84E-07 | 6.26E-06 | 3 |
| PACG-IBD | CC | GO:0098553 | lumenal side of endoplasmic reticulum membrane | 155.53 | 8.07E-07 | 1.18E-05 | 3 |
| PACG-IBD | CC | GO:0098576 | lumenal side of membrane | 118.34 | 1.87E-06 | 2.05E-05 | 3 |
| PACG-IBD | CC | GO:0012507 | ER to Golgi transport vesicle membrane | 85.06 | 5.09E-06 | 4.48E-05 | 3 |
| PACG-IBD | CC | GO:0030134 | COPII-coated ER to Golgi transport vesicle | 57.30 | 1.68E-05 | 8.20E-05 | 3 |
| PACG-IBD | MF | GO:0023026 | MHC class II protein complex binding | 189.26 | 4.37E-07 | 2.01E-05 | 3 |
| PACG-IBD | MF | GO:0023023 | MHC protein complex binding | 138.11 | 1.16E-06 | 2.66E-05 | 3 |
| PACG-IBD | MF | GO:0042605 | peptide antigen binding | 108.73 | 2.41E-06 | 3.69E-05 | 3 |
| PACG-IBD | MF | GO:0032395 | MHC class II receptor activity | 340.67 | 1.41E-05 | 1.62E-04 | 2 |
| Uveitis-DD | BP | GO:0016045 | detection of bacterium | 169.52 | 6.02E-05 | 2.29E-02 | 2 |
| Uveitis-DD | BP | GO:0002483 | antigen processing and presentation of endogenous peptide antigen | 108.49 | 1.50E-04 | 2.29E-02 | 2 |
| Uveitis-DD | BP | GO:0019883 | antigen processing and presentation of endogenous antigen | 84.76 | 2.47E-04 | 2.79E-02 | 2 |
| Uveitis-DD | BP | GO:0001916 | positive regulation of T cell mediated cytotoxicity | 66.15 | 4.07E-04 | 2.81E-02 | 2 |
| Uveitis-DD | BP | GO:0001914 | regulation of T cell mediated cytotoxicity | 53.18 | 6.31E-04 | 3.56E-02 | 2 |
| Uveitis-DD | CC | GO:0042611 | MHC protein complex | 114.06 | 1.36E-04 | 6.71E-03 | 2 |
| Uveitis-DD | CC | GO:0098553 | lumenal side of endoplasmic reticulum membrane | 81.47 | 2.68E-04 | 6.71E-03 | 2 |
| Uveitis-DD | CC | GO:0098576 | lumenal side of membrane | 61.99 | 4.65E-04 | 7.74E-03 | 2 |
| Uveitis-DD | MF | GO:0042605 | peptide antigen binding | 56.95 | 5.50E-04 | 3.13E-02 | 2 |
| Uveitis-IBD | BP | GO:0048002 | antigen processing and presentation of peptide antigen | 54.29 | 4.15E-11 | 3.15E-08 | 7 |
| Uveitis-IBD | BP | GO:0001916 | positive regulation of T cell mediated cytotoxicity | 81.72 | 8.90E-11 | 3.38E-08 | 6 |
| Uveitis-IBD | BP | GO:0001914 | regulation of T cell mediated cytotoxicity | 65.70 | 3.52E-10 | 8.92E-08 | 6 |
| Uveitis-IBD | BP | GO:0002483 | antigen processing and presentation of endogenous peptide antigen | 111.68 | 7.01E-10 | 1.33E-07 | 5 |
| Uveitis-IBD | BP | GO:0001913 | T cell mediated cytotoxicity | 54.93 | 1.07E-09 | 1.63E-07 | 6 |
| Uveitis-IBD | BP | GO:0019883 | antigen processing and presentation of endogenous antigen | 87.25 | 2.63E-09 | 2.59E-07 | 5 |
| Uveitis-IBD | BP | GO:0002399 | MHC class II protein complex assembly | 139.60 | 1.54E-08 | 9.73E-07 | 4 |
| Uveitis-IBD | BP | GO:0002503 | peptide antigen assembly with MHC class II protein complex | 139.60 | 1.54E-08 | 9.73E-07 | 4 |
| Uveitis-IBD | BP | GO:0002396 | MHC protein complex assembly | 106.36 | 5.02E-08 | 2.24E-06 | 4 |
| Uveitis-IBD | BP | GO:0002501 | peptide antigen assembly with MHC protein complex | 106.36 | 5.02E-08 | 2.24E-06 | 4 |
| Uveitis-IBD | BP | GO:0019886 | antigen processing and presentation of exogenous peptide antigen via MHC class II | 72.05 | 2.61E-07 | 8.61E-06 | 4 |
| Uveitis-IBD | BP | GO:0002495 | antigen processing and presentation of peptide antigen via MHC class II | 63.82 | 4.31E-07 | 1.31E-05 | 4 |
| Uveitis-IBD | BP | GO:0002504 | antigen processing and presentation of peptide or polysaccharide antigen via MHC class II | 60.37 | 5.43E-07 | 1.59E-05 | 4 |
| Uveitis-IBD | BP | GO:0002478 | antigen processing and presentation of exogenous peptide antigen | 54.48 | 8.28E-07 | 2.33E-05 | 4 |
| Uveitis-IBD | BP | GO:0002486 | antigen processing and presentation of endogenous peptide antigen via MHC class I via ER pathway, TAP-independent | 104.70 | 2.89E-06 | 6.28E-05 | 3 |
| Uveitis-IBD | BP | GO:0002476 | antigen processing and presentation of endogenous peptide antigen via MHC class Ib | 98.54 | 3.51E-06 | 7.20E-05 | 3 |
| Uveitis-IBD | BP | GO:0002484 | antigen processing and presentation of endogenous peptide antigen via MHC class I via ER pathway | 98.54 | 3.51E-06 | 7.20E-05 | 3 |
| Uveitis-IBD | BP | GO:0002428 | antigen processing and presentation of peptide antigen via MHC class Ib | 93.07 | 4.20E-06 | 8.41E-05 | 3 |
| Uveitis-IBD | BP | GO:0019885 | antigen processing and presentation of endogenous peptide antigen via MHC class I | 72.84 | 9.07E-06 | 1.60E-04 | 3 |
| Uveitis-IBD | BP | GO:0002475 | antigen processing and presentation via MHC class Ib | 67.01 | 1.17E-05 | 2.03E-04 | 3 |
| Uveitis-IBD | BP | GO:0016045 | detection of bacterium | 69.80 | 3.68E-04 | 4.05E-03 | 2 |
| Uveitis-IBD | CC | GO:0042611 | MHC protein complex | 114.06 | 6.37E-10 | 5.55E-08 | 5 |
| Uveitis-IBD | CC | GO:0098553 | lumenal side of endoplasmic reticulum membrane | 81.47 | 3.85E-09 | 1.67E-07 | 5 |
| Uveitis-IBD | CC | GO:0098576 | lumenal side of membrane | 61.99 | 1.60E-08 | 4.03E-07 | 5 |
| Uveitis-IBD | CC | GO:0042613 | MHC class II protein complex | 134.18 | 1.85E-08 | 4.03E-07 | 4 |
| Uveitis-IBD | MF | GO:0042605 | peptide antigen binding | 77.52 | 3.09E-12 | 3.03E-10 | 7 |
| Uveitis-IBD | MF | GO:0032395 | MHC class II receptor activity | 208.19 | 2.39E-09 | 1.17E-07 | 4 |
| Uveitis-IBD | MF | GO:0023026 | MHC class II protein complex binding | 77.11 | 1.95E-07 | 4.78E-06 | 4 |
| Uveitis-IBD | MF | GO:0023023 | MHC protein complex binding | 56.27 | 7.24E-07 | 1.18E-05 | 4 |
| Uveitis-IBS | BP | GO:0002483 | antigen processing and presentation of endogenous peptide antigen | 92.05 | 9.32E-08 | 5.42E-05 | 4 |
| Uveitis-IBS | BP | GO:0019883 | antigen processing and presentation of endogenous antigen | 71.92 | 2.63E-07 | 6.38E-05 | 4 |
| Uveitis-IBS | BP | GO:0001916 | positive regulation of T cell mediated cytotoxicity | 56.13 | 7.32E-07 | 1.33E-04 | 4 |
| Uveitis-IBS | BP | GO:0002486 | antigen processing and presentation of endogenous peptide antigen via MHC class I via ER pathway, TAP-independent | 107.88 | 2.64E-06 | 2.54E-04 | 3 |
| Uveitis-IBS | BP | GO:0002476 | antigen processing and presentation of endogenous peptide antigen via MHC class Ib | 101.53 | 3.20E-06 | 2.54E-04 | 3 |
| Uveitis-IBS | BP | GO:0002484 | antigen processing and presentation of endogenous peptide antigen via MHC class I via ER pathway | 101.53 | 3.20E-06 | 2.54E-04 | 3 |
| Uveitis-IBS | BP | GO:0002428 | antigen processing and presentation of peptide antigen via MHC class Ib | 95.89 | 3.83E-06 | 2.54E-04 | 3 |
| Uveitis-IBS | BP | GO:0019885 | antigen processing and presentation of endogenous peptide antigen via MHC class I | 75.04 | 8.27E-06 | 4.38E-04 | 3 |
| Uveitis-IBS | BP | GO:0002475 | antigen processing and presentation via MHC class Ib | 69.04 | 1.07E-05 | 4.88E-04 | 3 |
| Uveitis-IBS | BP | GO:0002399 | MHC class II protein complex assembly | 71.92 | 3.46E-04 | 6.63E-03 | 2 |
| Uveitis-IBS | BP | GO:0002503 | peptide antigen assembly with MHC class II protein complex | 71.92 | 3.46E-04 | 6.63E-03 | 2 |
| Uveitis-IBS | BP | GO:0002396 | MHC protein complex assembly | 54.79 | 6.03E-04 | 9.75E-03 | 2 |
| Uveitis-IBS | BP | GO:0002501 | peptide antigen assembly with MHC protein complex | 54.79 | 6.03E-04 | 9.75E-03 | 2 |
| Uveitis-IBS | CC | GO:0042611 | MHC protein complex | 68.43 | 1.11E-05 | 1.15E-03 | 3 |
| Uveitis-IBS | CC | GO:0042613 | MHC class II protein complex | 67.09 | 4.00E-04 | 7.64E-03 | 2 |
| Uveitis-IBS | MF | GO:0042605 | peptide antigen binding | 55.37 | 2.84E-08 | 3.46E-06 | 5 |
| Uveitis-IBS | MF | GO:0032395 | MHC class II receptor activity | 104.09 | 1.60E-04 | 4.88E-03 | 2 |
| Uveitis-PUD | BP | GO:0002486 | antigen processing and presentation of endogenous peptide antigen via MHC class I via ER pathway, TAP-independent | 139.60 | 8.99E-05 | 7.10E-03 | 2 |
| Uveitis-PUD | BP | GO:0002476 | antigen processing and presentation of endogenous peptide antigen via MHC class Ib | 131.39 | 1.02E-04 | 7.10E-03 | 2 |
| Uveitis-PUD | BP | GO:0002484 | antigen processing and presentation of endogenous peptide antigen via MHC class I via ER pathway | 131.39 | 1.02E-04 | 7.10E-03 | 2 |
| Uveitis-PUD | BP | GO:0002428 | antigen processing and presentation of peptide antigen via MHC class Ib | 124.09 | 1.14E-04 | 7.10E-03 | 2 |
| Uveitis-PUD | BP | GO:0019885 | antigen processing and presentation of endogenous peptide antigen via MHC class I | 97.12 | 1.89E-04 | 7.10E-03 | 2 |
| Uveitis-PUD | BP | GO:0002475 | antigen processing and presentation via MHC class Ib | 89.35 | 2.24E-04 | 7.10E-03 | 2 |
| Uveitis-PUD | BP | GO:0002483 | antigen processing and presentation of endogenous peptide antigen | 89.35 | 2.24E-04 | 7.10E-03 | 2 |
| Uveitis-PUD | BP | GO:0019883 | antigen processing and presentation of endogenous antigen | 69.80 | 3.68E-04 | 8.77E-03 | 2 |
| Uveitis-PUD | BP | GO:0002474 | antigen processing and presentation of peptide antigen via MHC class I | 60.37 | 4.93E-04 | 1.11E-02 | 2 |
| Uveitis-PUD | BP | GO:0001916 | positive regulation of T cell mediated cytotoxicity | 54.48 | 6.06E-04 | 1.28E-02 | 2 |
| AMD-IBS | KEGG | hsa05330 | Allograft rejection | 51.59 | 1.21E-09 | 8.41E-08 | 6 |
| Cataract-IBS | KEGG | hsa05330 | Allograft rejection | 60.19 | 4.63E-04 | 5.56E-03 | 2 |
| Cataract-IBS | KEGG | hsa04940 | Type I diabetes mellitus | 53.35 | 5.90E-04 | 5.56E-03 | 2 |
| Cataract-IBS | KEGG | hsa05332 | Graft-versus-host disease | 52.17 | 6.17E-04 | 5.56E-03 | 2 |
| Myopia-IBS | KEGG | hsa05330 | Allograft rejection | 51.59 | 1.21E-09 | 8.41E-08 | 6 |
| PACG-IBD | KEGG | hsa05330 | Allograft rejection | 103.19 | 2.29E-06 | 1.41E-05 | 3 |
| PACG-IBD | KEGG | hsa04940 | Type I diabetes mellitus | 91.46 | 3.32E-06 | 1.64E-05 | 3 |
| PACG-IBD | KEGG | hsa05332 | Graft-versus-host disease | 89.43 | 3.55E-06 | 1.64E-05 | 3 |
| PACG-IBD | KEGG | hsa05320 | Autoimmune thyroid disease | 74.52 | 6.19E-06 | 2.29E-05 | 3 |
| PACG-IBD | KEGG | hsa05416 | Viral myocarditis | 57.49 | 1.36E-05 | 4.19E-05 | 3 |
| Uveitis-DD | KEGG | hsa05330 | Allograft rejection | 68.79 | 3.48E-04 | 7.12E-03 | 2 |
| Uveitis-DD | KEGG | hsa04940 | Type I diabetes mellitus | 60.97 | 4.44E-04 | 7.12E-03 | 2 |
| Uveitis-DD | KEGG | hsa05332 | Graft-versus-host disease | 59.62 | 4.64E-04 | 7.12E-03 | 2 |
| Uveitis-IBD | KEGG | hsa05330 | Allograft rejection | 60.19 | 1.40E-08 | 7.98E-07 | 5 |
| Uveitis-IBD | KEGG | hsa04940 | Type I diabetes mellitus | 53.35 | 2.63E-08 | 7.98E-07 | 5 |
| Uveitis-IBD | KEGG | hsa05332 | Graft-versus-host disease | 52.17 | 2.96E-08 | 7.98E-07 | 5 |
| Uveitis-PUD | KEGG | hsa04940 | Type I diabetes mellitus | 71.14 | 7.91E-06 | 4.59E-04 | 3 |

**Table S10**. Enriched pathways in GSEA analysis across the 40 trait pairs

| **Trait pairs** | **Category** | **ID** | **Description** | **NES** | ***P*** | PFDR |
| --- | --- | --- | --- | --- | --- | --- |
| DED-DD | CC | GO:0005634 | nucleus | 2.28 | 3.40E-02 | 3.40E-02 |
| DED-DD | CC | GO:0005622 | intracellular anatomical structure | 2.18 | 2.90E-02 | 3.40E-02 |
| DED-DD | CC | GO:0043226 | organelle | 2.18 | 2.90E-02 | 3.40E-02 |
| DED-DD | CC | GO:0043227 | membrane-bounded organelle | 2.18 | 2.90E-02 | 3.40E-02 |
| DED-DD | CC | GO:0043229 | intracellular organelle | 2.18 | 2.90E-02 | 3.40E-02 |
| DED-DD | CC | GO:0043231 | intracellular membrane-bounded organelle | 2.18 | 2.90E-02 | 3.40E-02 |
| DED-IBS | BP | GO:0007275 | multicellular organism development | 2.79 | 1.70E-03 | 2.27E-03 |
| DED-IBS | BP | GO:0032501 | multicellular organismal process | 2.79 | 1.70E-03 | 2.27E-03 |
| DED-IBS | BP | GO:0048856 | anatomical structure development | 2.79 | 1.70E-03 | 2.27E-03 |
| DED-IBS | BP | GO:0032502 | developmental process | 2.73 | 3.98E-03 | 3.98E-03 |
| DR-IBD | BP | GO:0002684 | positive regulation of immune system process | 2.63 | 1.04E-03 | 1.12E-02 |
| DR-IBD | BP | GO:0065003 | protein-containing complex assembly | 2.60 | 1.67E-03 | 1.12E-02 |
| DR-IBD | BP | GO:0050776 | regulation of immune response | 2.57 | 1.52E-03 | 1.12E-02 |
| DR-IBD | BP | GO:0050778 | positive regulation of immune response | 2.57 | 1.52E-03 | 1.12E-02 |
| DR-IBD | BP | GO:0002682 | regulation of immune system process | 2.50 | 2.50E-03 | 1.12E-02 |
| DR-IBD | BP | GO:0043933 | protein-containing complex organization | 2.42 | 3.83E-03 | 1.12E-02 |
| DR-IBD | BP | GO:0002443 | leukocyte mediated immunity | 2.32 | 5.91E-03 | 1.12E-02 |
| DR-IBD | BP | GO:0002449 | lymphocyte mediated immunity | 2.32 | 5.91E-03 | 1.12E-02 |
| DR-IBD | BP | GO:0002697 | regulation of immune effector process | 2.32 | 5.91E-03 | 1.12E-02 |
| DR-IBD | BP | GO:0002699 | positive regulation of immune effector process | 2.32 | 5.91E-03 | 1.12E-02 |
| DR-IBD | BP | GO:0031347 | regulation of defense response | 2.32 | 6.06E-03 | 1.12E-02 |
| DR-IBD | BP | GO:0032101 | regulation of response to external stimulus | 2.32 | 6.06E-03 | 1.12E-02 |
| DR-IBD | BP | GO:0007159 | leukocyte cell-cell adhesion | 2.28 | 9.19E-03 | 1.12E-02 |
| DR-IBD | BP | GO:0022409 | positive regulation of cell-cell adhesion | 2.28 | 9.19E-03 | 1.12E-02 |
| DR-IBD | BP | GO:0045785 | positive regulation of cell adhesion | 2.28 | 9.19E-03 | 1.12E-02 |
| DR-IBD | BP | GO:0050863 | regulation of T cell activation | 2.28 | 9.19E-03 | 1.12E-02 |
| DR-IBD | BP | GO:0050870 | positive regulation of T cell activation | 2.28 | 9.19E-03 | 1.12E-02 |
| DR-IBD | BP | GO:1903037 | regulation of leukocyte cell-cell adhesion | 2.28 | 9.19E-03 | 1.12E-02 |
| DR-IBD | BP | GO:1903039 | positive regulation of leukocyte cell-cell adhesion | 2.28 | 9.19E-03 | 1.12E-02 |
| DR-IBD | BP | GO:0006955 | immune response | 2.28 | 8.44E-03 | 1.12E-02 |
| DR-IBD | BP | GO:0051240 | positive regulation of multicellular organismal process | 2.27 | 8.59E-03 | 1.12E-02 |
| DR-IBD | BP | GO:0048584 | positive regulation of response to stimulus | 2.24 | 4.95E-03 | 1.12E-02 |
| DR-IBD | BP | GO:0002376 | immune system process | 2.24 | 6.28E-03 | 1.12E-02 |
| DR-IBD | BP | GO:0022607 | cellular component assembly | 2.18 | 1.16E-02 | 1.30E-02 |
| DR-IBD | BP | GO:0044085 | cellular component biogenesis | 2.18 | 1.16E-02 | 1.30E-02 |
| DR-IBD | BP | GO:0019882 | antigen processing and presentation | 2.28 | 1.35E-02 | 1.35E-02 |
| DR-IBD | BP | GO:0048002 | antigen processing and presentation of peptide antigen | 2.28 | 1.35E-02 | 1.35E-02 |
| DR-IBD | BP | GO:0002250 | adaptive immune response | 2.24 | 1.31E-02 | 1.35E-02 |
| DR-IBD | CC | GO:0030139 | endocytic vesicle | 2.39 | 4.42E-03 | 8.00E-03 |
| DR-IBD | CC | GO:0032991 | protein-containing complex | 2.35 | 2.09E-03 | 8.00E-03 |
| DR-IBD | CC | GO:0043230 | extracellular organelle | 2.20 | 8.00E-03 | 8.00E-03 |
| DR-IBD | CC | GO:0065010 | extracellular membrane-bounded organelle | 2.20 | 8.00E-03 | 8.00E-03 |
| DR-IBD | CC | GO:0070062 | extracellular exosome | 2.20 | 8.00E-03 | 8.00E-03 |
| DR-IBD | CC | GO:1903561 | extracellular vesicle | 2.20 | 8.00E-03 | 8.00E-03 |
| DR-IBD | MF | GO:0044877 | protein-containing complex binding | 2.95 | 1.51E-04 | 6.03E-04 |
| DR-IBD | MF | GO:0003823 | antigen binding | 2.29 | 6.13E-03 | 6.13E-03 |
| DR-IBD | MF | GO:0042277 | peptide binding | 2.29 | 6.13E-03 | 6.13E-03 |
| DR-IBD | MF | GO:0042605 | peptide antigen binding | 2.29 | 6.13E-03 | 6.13E-03 |
| DR-IBD | KEGG | hsa05166 | Human T-cell leukemia virus 1 infection | 2.48 | 2.90E-03 | 7.74E-03 |
| DR-IBD | KEGG | hsa04145 | Phagosome | 2.41 | 4.42E-03 | 7.74E-03 |
| DR-IBD | KEGG | hsa04612 | Antigen processing and presentation | 2.41 | 4.42E-03 | 7.74E-03 |
| DR-IBD | KEGG | hsa05168 | Herpes simplex virus 1 infection | 2.38 | 4.35E-03 | 7.74E-03 |
| DR-IBD | KEGG | hsa05169 | Epstein-Barr virus infection | 2.19 | 1.04E-02 | 1.45E-02 |
| DR-IBD | KEGG | hsa04514 | Cell adhesion molecules | 2.07 | 2.70E-02 | 2.70E-02 |
| DR-IBD | KEGG | hsa05416 | Viral myocarditis | 2.07 | 2.70E-02 | 2.70E-02 |

**Table S11**. Significant enriched tissues across the 40 gastrointestinal-eye trait pairs in TSEA

| Trait Pairs | Log10PValue | Tissue.Specific.Genes | fold.change | samples | Tissue | *P* | *P_*FDR |
| --- | --- | --- | --- | --- | --- | --- | --- |
| DR-IBS | 3.69 | 8 | 8.25 | 9 | Lung | 2.06E-04 | 7.19E-03 |
| Uveitis-IBS | 2.67 | 5 | 11.87 | 9 | Lung | 2.13E-03 | 7.47E-02 |

**Table S12**. Enriched tissues across the 40 gastrointestinal-eye trait pairs in CSEA

| Trait Pair | Tissue_cell_type_name | Stage | System | Cell_type | Cell_Ontology_ID | Tissue | *P^a^* |
| --- | --- | --- | --- | --- | --- | --- | --- |
| AMD-GORD | TS_Thymus_cd8-positive, alpha-beta cytotoxic t cell | Adult | Lymphatic system | T cell | CL:0000084 | Thymus | 9.11E-08 |
| AMD-GORD | TS_Thymus_naive regulatory t cell | Adult | Lymphatic system | T cell | CL:0000084 | Thymus | 5.65E-07 |
| AMD-GORD | TS_Tongue_epithelial cell | Adult | Digestive system | Epithelial cell | CL:0000066 | Tongue | 4.73E-06 |
| AMD-GORD | TS_Thymus_b cell | Adult | Lymphatic system | B cell | CL:0000236 | Thymus | 1.69E-05 |
| AMD-GORD | TS_Muscle_endothelial cell of artery | Adult | Muscular system | Endothelial cell | CL:0000115 | Muscle organ | 5.67E-06 |
| AMD-GORD | TS_Thymus_t follicular helper cell | Adult | Lymphatic system | T cell | CL:0000084 | Thymus | 1.69E-05 |
| AMD-IBD | FetalLiver_Macrophage | Fetal | Digestive system | Macrophage | CL:0000235 | Liver | 1.35E-07 |
| AMD-IBD | NeonatalAdrenalGland_Macrophage | Neonatal | Endocrine system | Macrophage | CL:0000235 | Adrenal gland | 7.86E-06 |
| AMD-IBD | TS_Muscle_endothelial cell of artery | Adult | Muscular system | Endothelial cell | CL:0000115 | Muscle organ | 8.68E-10 |
| AMD-IBD | FetalHeart_Macrophage | Fetal | Cardiovascular system | Macrophage | CL:0000235 | Heart | 6.49E-06 |
| AMD-IBD | AdultArtery_Macrophage | Adult | Cardiovascular system | Macrophage | CL:0000235 | Artery | 2.31E-05 |
| AMD-IBD | AdultCerebellum_Macrophage | Adult | Nervous system | Microglial cell | CL:0000129 | Cerebellum | 2.89E-05 |
| AMD-IBD | TS_Tongue_epithelial cell | Adult | Digestive system | Epithelial cell | CL:0000066 | Tongue | 4.36E-09 |
| AMD-IBD | AdultLung_M2 Macrophage | Adult | Respiratory system | Macrophage | CL:0000235 | Lung | 2.73E-05 |
| AMD-IBD | FetalFemaleGonad_Macrophage | Fetal | Reproductive system | Macrophage | CL:0000235 | Immature gonad | 3.44E-05 |
| AMD-IBD | TS_Thymus_cd8-positive, alpha-beta cytotoxic t cell | Adult | Lymphatic system | T cell | CL:0000084 | Thymus | 2.09E-08 |
| AMD-IBD | TS_Thymus_b cell | Adult | Lymphatic system | B cell | CL:0000236 | Thymus | 1.19E-07 |
| AMD-IBD | TS_Pancreas_endothelial cell | Adult | Digestive system | Endothelial cell | CL:0000115 | Pancreas | 2.60E-08 |
| AMD-IBD | TS_Bone_Marrow_macrophage | Adult | Lymphatic system | Macrophage | CL:0000235 | Bone marrow | 1.75E-07 |
| AMD-IBD | TS_Thymus_naive regulatory t cell | Adult | Lymphatic system | T cell | CL:0000084 | Thymus | 6.28E-07 |
| AMD-IBD | TS_Lung_macrophage | Adult | Respiratory system | Macrophage | CL:0000235 | Lung | 7.53E-07 |
| AMD-IBD | TS_Pancreas_myeloid cell | Adult | Digestive system | Myeloid cell | CL:0000763 | Pancreas | 7.54E-07 |
| AMD-IBD | TS_Bladder_capillary endothelial cell | Adult | Urinary system | Capillary endothelial cell | CL:0002144 | Bladder organ | 3.65E-06 |
| AMD-IBD | TS_Mammary_vein endothelial cell | Adult | Endocrine system | Endothelial cell | CL:0000115 | Mammary gland | 1.22E-05 |
| AMD-IBD | TS_Lymph_Node_b cell | Adult | Lymphatic system | B cell | CL:0000236 | Lymph node | 1.92E-05 |
| AMD-IBD | TS_Spleen_macrophage | Adult | Lymphatic system | Macrophage | CL:0000235 | Spleen | 1.91E-05 |
| AMD-IBD | TS_Blood_macrophage | Adult | Lymphatic system | Macrophage | CL:0000235 | Blood | 1.92E-05 |
| AMD-IBD | TS_Salivary_Gland_macrophage | Adult | Digestive system | Macrophage | CL:0000235 | Saliva-secreting gland | 2.21E-05 |
| AMD-IBD | TS_Blood_monocyte | Adult | Lymphatic system | Monocyte | CL:0000576 | Blood | 1.92E-05 |
| AMD-IBS | AdultSigmoidColon_B cell | Adult | Digestive system | B cell | CL:0000236 | Sigmoid colon | 3.62E-06 |
| AMD-IBS | AdultCerebellum_Macrophage | Adult | Nervous system | Microglial cell | CL:0000129 | Cerebellum | 6.07E-06 |
| AMD-IBS | AdultTemporalLobe_Macrophage | Adult | Nervous system | Microglial cell | CL:0000129 | Temporal lobe | 7.68E-06 |
| AMD-IBS | FetalLiver_Macrophage | Fetal | Digestive system | Macrophage | CL:0000235 | Liver | 9.37E-06 |
| AMD-IBS | FetalThymus_Dendritic cell | Fetal | Lymphatic system | Dendritic cell | CL:0000451 | Thymus | 1.02E-05 |
| AMD-IBS | AdultPancreas_Macrophage | Adult | Digestive system | Macrophage | CL:0000235 | Pancreas | 3.17E-05 |
| AMD-IBS | FetalLiver_Dendritic cell | Fetal | Digestive system | Dendritic cell | CL:0000451 | Liver | 9.37E-06 |
| AMD-IBS | TS_Muscle_endothelial cell of artery | Adult | Muscular system | Endothelial cell | CL:0000115 | Muscle organ | 6.79E-08 |
| AMD-IBS | AdultArtery_Macrophage | Adult | Cardiovascular system | Macrophage | CL:0000235 | Artery | 1.23E-05 |
| AMD-IBS | AdultPleura_Dendritic cell | Adult | Respiratory system | Dendritic cell | CL:0000451 | Pleura | 1.52E-05 |
| AMD-IBS | NeonatalAdrenalGland_Macrophage | Neonatal | Endocrine system | Macrophage | CL:0000235 | Adrenal gland | 2.00E-05 |
| AMD-IBS | AdultLung_M2 Macrophage | Adult | Respiratory system | Macrophage | CL:0000235 | Lung | 1.90E-05 |
| AMD-IBS | FetalHeart_Macrophage | Fetal | Cardiovascular system | Macrophage | CL:0000235 | Heart | 2.53E-05 |
| AMD-IBS | FetalFemaleGonad_Macrophage | Fetal | Reproductive system | Macrophage | CL:0000235 | Immature gonad | 3.17E-05 |
| AMD-IBS | FetalFemaleGonad_Dendritic cell | Fetal | Reproductive system | Dendritic cell | CL:0000451 | Immature gonad | 3.17E-05 |
| AMD-IBS | TS_Tongue_epithelial cell | Adult | Digestive system | Epithelial cell | CL:0000066 | Tongue | 3.70E-07 |
| AMD-IBS | TS_Lung_macrophage | Adult | Respiratory system | Macrophage | CL:0000235 | Lung | 4.69E-07 |
| AMD-IBS | TS_Pancreas_endothelial cell | Adult | Digestive system | Endothelial cell | CL:0000115 | Pancreas | 2.39E-06 |
| AMD-IBS | TS_Bone_Marrow_macrophage | Adult | Lymphatic system | Macrophage | CL:0000235 | Bone marrow | 9.34E-06 |
| AMD-IBS | TS_Thymus_b cell | Adult | Lymphatic system | B cell | CL:0000236 | Thymus | 1.15E-05 |
| AMD-IBS | TS_Lymph_Node_b cell | Adult | Lymphatic system | B cell | CL:0000236 | Lymph node | 9.37E-06 |
| AMD-IBS | TS_Pancreas_myeloid cell | Adult | Digestive system | Myeloid cell | CL:0000763 | Pancreas | 1.40E-05 |
| AMD-PUD | TS_Muscle_endothelial cell of artery | Adult | Muscular system | Endothelial cell | CL:0000115 | Muscle organ | 1.84E-07 |
| AMD-PUD | TS_Tongue_epithelial cell | Adult | Digestive system | Epithelial cell | CL:0000066 | Tongue | 7.49E-07 |
| AMD-PUD | TS_Thymus_endothelial cell of artery | Adult | Lymphatic system | Endothelial cell | CL:0000115 | Thymus | 3.66E-06 |
| AMD-PUD | TS_Thymus_vein endothelial cell | Adult | Lymphatic system | Endothelial cell | CL:0000115 | Thymus | 3.66E-06 |
| AMD-PUD | TS_Pancreas_endothelial cell | Adult | Digestive system | Endothelial cell | CL:0000115 | Pancreas | 2.48E-05 |
| Cataract-GORD | TS_Kidney_endothelial cell | Adult | Urinary system | Endothelial cell | CL:0000115 | Kidney | 1.57E-07 |
| Cataract-GORD | TS_Kidney_macrophage | Adult | Urinary system | Macrophage | CL:0000235 | Kidney | 1.89E-06 |
| Cataract-GORD | TS_Bladder_capillary endothelial cell | Adult | Urinary system | Capillary endothelial cell | CL:0002144 | Bladder organ | 3.64E-05 |
| Cataract-IBS | Fetal_HCA_Pancreas_Lymphatic endothelial cells | Fetal | Digestive system | Endothelial cell | CL:0000115 | Pancreas | 3.26E-05 |
| Cataract-IBS | Fetal_HCA_Stomach_ENS neurons | Fetal | Digestive system | ENS neuron | CL:0000540 | Stomach | 3.26E-05 |
| Cataract-IBS | TS_Lung_endothelial cell of lymphatic vessel | Adult | Respiratory system | Endothelial cell | CL:0000115 | Lung | 3.30E-05 |
| Cataract-IBS | TS_Uterus_vascular associated smooth muscle cell | Adult | Reproductive system | Smooth muscle cell | CL:0000192 | Uterus | 3.29E-05 |
| Cataract-IBS | TS_Lung_intermediate monocyte | Adult | Respiratory system | Monocyte | CL:0000576 | Lung | 3.30E-05 |
| Cataract-IBS | TS_Tongue_endothelial cell of lymphatic vessel | Adult | Digestive system | Endothelial cell | CL:0000115 | Tongue | 1.95E-06 |
| Cataract-IBS | TS_Bladder_pericyte cell | Adult | Urinary system | Pericyte cell | CL:0000669 | Bladder organ | 3.29E-05 |
| DED-DD | TS_Heart_fibroblast of cardiac tissue | Adult | Cardiovascular system | Fibroblast | CL:0000057 | Heart | 9.65E-06 |
| DED-PUD | Fetal_HCA_Cerebellum_10k_Purkinje neurons | Fetal | Nervous system | Neuron | CL:0000540 | Cerebellum | 1.07E-06 |
| DED-PUD | Fetal_HCA_Cerebrum_10k_SKOR2_NPSR1 positive cells | Fetal | Nervous system | Neuron | CL:0000540 | Cerebral hemisphere | 1.51E-05 |
| DED-PUD | TS_Small_Intestine_duodenum glandular cell | Adult | Digestive system | Duodenum glandular cell | CL:1001589 | Small intestine | 1.51E-05 |
| DED-PUD | TS_Small_Intestine_monocyte | Adult | Digestive system | Monocyte | CL:0000576 | Small intestine | 1.51E-05 |
| DR-DD | TS_Lung_macrophage | Adult | Respiratory system | Macrophage | CL:0000235 | Lung | 1.17E-10 |
| DR-DD | Fetal_HCA_Spleen_Lymphoid cells | Fetal | Lymphatic system | Innate lymphoid cell | CL:0001065 | Spleen | 3.41E-05 |
| DR-DD | TS_Small_Intestine_fibroblast | Adult | Digestive system | Fibroblast | CL:0000057 | Small intestine | 1.36E-09 |
| DR-DD | TS_Thymus_b cell | Adult | Lymphatic system | B cell | CL:0000236 | Thymus | 5.55E-09 |
| DR-DD | TS_Spleen_cd141-positive myeloid dendritic cell | Adult | Lymphatic system | Dendritic cell | CL:0000451 | Spleen | 1.37E-09 |
| DR-DD | VentoTormo_2018_decidua_decidual macrophage (dM2) | Maternal-Fetal | Reproductive system | Macrophage | CL:0000235 | Decidua | 5.20E-08 |
| DR-DD | TS_Salivary_Gland_naive b cell | Adult | Digestive system | B cell | CL:0000236 | Saliva-secreting gland | 3.47E-08 |
| DR-DD | TS_Lymph_Node_cd1c-positive myeloid dendritic cell | Adult | Lymphatic system | Dendritic cell | CL:0000451 | Lymph node | 5.58E-09 |
| DR-DD | TS_Lung_dendritic cell | Adult | Respiratory system | Dendritic cell | CL:0000451 | Lung | 1.09E-06 |
| DR-DD | TS_Pancreas_myeloid cell | Adult | Digestive system | Myeloid cell | CL:0000763 | Pancreas | 4.31E-08 |
| DR-DD | TS_Large_Intestine_fibroblast | Adult | Digestive system | Fibroblast | CL:0000057 | Large intestine | 5.29E-08 |
| DR-DD | TS_Blood_memory b cell | Adult | Lymphatic system | B cell | CL:0000236 | Blood | 5.32E-08 |
| DR-DD | TS_Prostate_endothelial cell | Adult | Reproductive system | Endothelial cell | CL:0000115 | Prostate gland | 2.01E-07 |
| DR-DD | TS_Liver_macrophage | Adult | Digestive system | Macrophage | CL:0000235 | Liver | 2.01E-07 |
| DR-DD | TS_Blood_naive b cell | Adult | Lymphatic system | B cell | CL:0000236 | Blood | 3.00E-07 |
| DR-DD | TS_Eye_retinal blood vessel endothelial cell | Adult | Sensory system | Endothelial cell | CL:0000115 | Eye | 5.33E-08 |
| DR-DD | TS_Vasculature_vein or capillary endothelial cell | Adult | Cardiovascular system | Capillary endothelial cell | CL:0002144 | Vasculature | 2.01E-07 |
| DR-DD | TS_Thymus_dendritic cell | Adult | Lymphatic system | Dendritic cell | CL:0000451 | Thymus | 1.09E-06 |
| DR-DD | TS_Lymph_Node_cd141-positive myeloid dendritic cell | Adult | Lymphatic system | Dendritic cell | CL:0000451 | Lymph node | 2.02E-07 |
| DR-DD | TS_Eye_monocyte | Adult | Sensory system | Monocyte | CL:0000576 | Eye | 3.00E-07 |
| DR-DD | TS_Bladder_macrophage | Adult | Urinary system | Macrophage | CL:0000235 | Bladder organ | 2.98E-07 |
| DR-DD | TS_Bone_Marrow_monocyte | Adult | Lymphatic system | Monocyte | CL:0000576 | Bone marrow | 2.98E-07 |
| DR-DD | TS_Lymph_Node_hematopoietic stem cell | Adult | Lymphatic system | Hematopoietic stem cell | CL:0000037 | Lymph node | 1.09E-06 |
| DR-DD | TS_Salivary_Gland_endothelial cell | Adult | Digestive system | Endothelial cell | CL:0000115 | Saliva-secreting gland | 1.09E-06 |
| DR-DD | TS_Blood_monocyte | Adult | Lymphatic system | Monocyte | CL:0000576 | Blood | 3.00E-07 |
| DR-DD | TS_Bone_Marrow_memory b cell | Adult | Lymphatic system | B cell | CL:0000236 | Bone marrow | 7.60E-06 |
| DR-DD | TS_Large_Intestine_b cell | Adult | Digestive system | B cell | CL:0000236 | Large intestine | 1.56E-06 |
| DR-DD | VentoTormo_2018_decidua_monocyte | Maternal-Fetal | Reproductive system | Monocyte | CL:0000576 | Decidua | 7.51E-06 |
| DR-DD | TS_Lymph_Node_b cell | Adult | Lymphatic system | B cell | CL:0000236 | Lymph node | 1.09E-06 |
| DR-DD | TS_Large_Intestine_monocyte | Adult | Digestive system | Monocyte | CL:0000576 | Large intestine | 1.56E-06 |
| DR-DD | TS_Mammary_endothelial cell of artery | Adult | Endocrine system | Endothelial cell | CL:0000115 | Mammary gland | 5.45E-06 |
| DR-DD | TS_Small_Intestine_b cell | Adult | Digestive system | B cell | CL:0000236 | Small intestine | 1.56E-06 |
| DR-DD | TS_Eye_macrophage | Adult | Sensory system | Macrophage | CL:0000235 | Eye | 1.57E-06 |
| DR-DD | TS_Tongue_epithelial cell | Adult | Digestive system | Epithelial cell | CL:0000066 | Tongue | 1.31E-06 |
| DR-DD | TS_Prostate_macrophage | Adult | Reproductive system | Macrophage | CL:0000235 | Prostate gland | 5.45E-06 |
| DR-DD | TS_Bladder_capillary endothelial cell | Adult | Urinary system | Capillary endothelial cell | CL:0002144 | Bladder organ | 1.56E-06 |
| DR-DD | TS_Liver_liver dendritic cell | Adult | Digestive system | Dendritic cell | CL:0000451 | Liver | 2.53E-05 |
| DR-DD | TS_Eye_microglial cell | Adult | Sensory system | Microglial cell | CL:0000129 | Eye | 7.63E-06 |
| DR-DD | TS_Prostate_basal cell of prostate epithelium | Adult | Reproductive system | Basal cell | CL:0000646 | Prostate gland | 5.45E-06 |
| DR-DD | TS_Eye_corneal keratocyte | Adult | Sensory system | Fibroblast | CL:0000057 | Eye | 1.57E-06 |
| DR-DD | TS_Salivary_Gland_memory b cell | Adult | Digestive system | B cell | CL:0000236 | Saliva-secreting gland | 5.47E-06 |
| DR-DD | TS_Trachea_macrophage | Adult | Respiratory system | Macrophage | CL:0000235 | Trachea | 6.42E-06 |
| DR-DD | TS_Spleen_cd1c-positive myeloid dendritic cell | Adult | Lymphatic system | Dendritic cell | CL:0000451 | Spleen | 7.61E-06 |
| DR-DD | TS_Skin_macrophage | Adult | Integumentary system | Macrophage | CL:0000235 | Skin of body | 2.53E-05 |
| DR-DD | TS_Uterus_macrophage | Adult | Reproductive system | Macrophage | CL:0000235 | Uterus | 2.16E-05 |
| DR-DD | TS_Thymus_fibroblast | Adult | Lymphatic system | Fibroblast | CL:0000057 | Thymus | 5.46E-06 |
| DR-DD | TS_Thymus_vein endothelial cell | Adult | Lymphatic system | Endothelial cell | CL:0000115 | Thymus | 5.46E-06 |
| DR-DD | TS_Bone_Marrow_macrophage | Adult | Lymphatic system | Macrophage | CL:0000235 | Bone marrow | 7.60E-06 |
| DR-DD | TS_Salivary_Gland_adventitial cell | Adult | Digestive system | Adventitial cell | CL:0002503 | Saliva-secreting gland | 2.54E-05 |
| DR-DD | TS_Uterus_endothelial cell of lymphatic vessel | Adult | Reproductive system | Endothelial cell | CL:0000115 | Uterus | 2.16E-05 |
| DR-DD | TS_Trachea_endothelial cell | Adult | Respiratory system | Endothelial cell | CL:0000115 | Trachea | 2.94E-05 |
| DR-DD | TS_Prostate_myeloid cell | Adult | Reproductive system | Myeloid cell | CL:0000763 | Prostate gland | 2.53E-05 |
| DR-DD | TS_Liver_monocyte | Adult | Digestive system | Monocyte | CL:0000576 | Liver | 2.53E-05 |
| DR-DD | TS_Uterus_endothelial cell | Adult | Reproductive system | Endothelial cell | CL:0000115 | Uterus | 2.16E-05 |
| DR-DD | TS_Lymph_Node_endothelial cell | Adult | Lymphatic system | Endothelial cell | CL:0000115 | Lymph node | 2.54E-05 |
| DR-DD | TS_Blood_classical monocyte | Adult | Lymphatic system | Monocyte | CL:0000576 | Blood | 7.62E-06 |
| DR-DD | TS_Bone_Marrow_hematopoietic stem cell | Adult | Lymphatic system | Hematopoietic stem cell | CL:0000037 | Bone marrow | 3.42E-05 |
| DR-DD | TS_Lymph_Node_intermediate monocyte | Adult | Lymphatic system | Monocyte | CL:0000576 | Lymph node | 2.54E-05 |
| DR-DD | TS_Tongue_basal cell | Adult | Digestive system | Basal cell | CL:0000646 | Tongue | 2.96E-05 |
| DR-DD | TS_Thymus_endothelial cell of artery | Adult | Lymphatic system | Endothelial cell | CL:0000115 | Thymus | 2.53E-05 |
| DR-DD | TS_Spleen_memory b cell | Adult | Lymphatic system | B cell | CL:0000236 | Spleen | 3.42E-05 |
| DR-DD | TS_Small_Intestine_paneth cell of epithelium of small intestine | Adult | Digestive system | Paneth cell | CL:0000510 | Small intestine | 3.42E-05 |
| DR-DD | TS_Pancreas_endothelial cell | Adult | Digestive system | Endothelial cell | CL:0000115 | Pancreas | 2.95E-05 |
| DR-DD | TS_Muscle_endothelial cell of vascular tree | Adult | Muscular system | Endothelial cell | CL:0000115 | Muscle organ | 3.42E-05 |
| DR-DD | TS_Muscle_mesenchymal stem cell | Adult | Muscular system | Mesenchymal stem cell | CL:0000134 | Muscle organ | 3.42E-05 |
| DR-DD | TS_Spleen_classical monocyte | Adult | Lymphatic system | Monocyte | CL:0000576 | Spleen | 3.42E-05 |
| DR-DD | TS_Spleen_macrophage | Adult | Lymphatic system | Macrophage | CL:0000235 | Spleen | 3.42E-05 |
| DR-DD | TS_Eye_corneal epithelial cell | Adult | Sensory system | Epithelial cell | CL:0000066 | Eye | 3.43E-05 |
| DR-GORD | TS_Lymph_Node_b cell | Adult | Lymphatic system | B cell | CL:0000236 | Lymph node | 8.44E-11 |
| DR-GORD | TS_Large_Intestine_b cell | Adult | Digestive system | B cell | CL:0000236 | Large intestine | 6.76E-10 |
| DR-GORD | Fetal_HCA_Eye_Vascular endothelial cells | Fetal | Sensory system | Endothelial cell | CL:0000115 | Eye | 2.08E-05 |
| DR-GORD | TS_Kidney_b cell | Adult | Urinary system | B cell | CL:0000236 | Kidney | 1.25E-06 |
| DR-GORD | TS_Bone_Marrow_memory b cell | Adult | Lymphatic system | B cell | CL:0000236 | Bone marrow | 1.89E-08 |
| DR-GORD | TS_Trachea_macrophage | Adult | Respiratory system | Macrophage | CL:0000235 | Trachea | 2.63E-09 |
| DR-GORD | TS_Liver_liver dendritic cell | Adult | Digestive system | Dendritic cell | CL:0000451 | Liver | 5.73E-08 |
| DR-GORD | TS_Lung_macrophage | Adult | Respiratory system | Macrophage | CL:0000235 | Lung | 3.30E-09 |
| DR-GORD | TS_Eye_microglial cell | Adult | Sensory system | Microglial cell | CL:0000129 | Eye | 2.81E-08 |
| DR-GORD | TS_Prostate_myeloid cell | Adult | Reproductive system | Myeloid cell | CL:0000763 | Prostate gland | 2.29E-08 |
| DR-GORD | TS_Blood_classical monocyte | Adult | Lymphatic system | Monocyte | CL:0000576 | Blood | 3.31E-09 |
| DR-GORD | TS_Small_Intestine_b cell | Adult | Digestive system | B cell | CL:0000236 | Small intestine | 2.30E-08 |
| DR-GORD | TS_Thymus_b cell | Adult | Lymphatic system | B cell | CL:0000236 | Thymus | 1.22E-07 |
| DR-GORD | TS_Lymph_Node_memory b cell | Adult | Lymphatic system | B cell | CL:0000236 | Lymph node | 2.43E-06 |
| DR-GORD | TS_Salivary_Gland_memory b cell | Adult | Digestive system | B cell | CL:0000236 | Saliva-secreting gland | 2.80E-08 |
| DR-GORD | TS_Kidney_macrophage | Adult | Urinary system | Macrophage | CL:0000235 | Kidney | 2.51E-07 |
| DR-GORD | VentoTormo_2018_decidua_monocyte | Maternal-Fetal | Reproductive system | Monocyte | CL:0000576 | Decidua | 8.34E-07 |
| DR-GORD | TS_Thymus_memory b cell | Adult | Lymphatic system | B cell | CL:0000236 | Thymus | 2.83E-06 |
| DR-GORD | TS_Liver_macrophage | Adult | Digestive system | Macrophage | CL:0000235 | Liver | 3.02E-07 |
| DR-GORD | TS_Large_Intestine_plasma cell | Adult | Digestive system | Plasma cell | CL:0000786 | Large intestine | 6.07E-07 |
| DR-GORD | TS_Blood_monocyte | Adult | Lymphatic system | Monocyte | CL:0000576 | Blood | 1.02E-07 |
| DR-GORD | TS_Bone_Marrow_macrophage | Adult | Lymphatic system | Macrophage | CL:0000235 | Bone marrow | 5.13E-07 |
| DR-GORD | TS_Spleen_cd141-positive myeloid dendritic cell | Adult | Lymphatic system | Dendritic cell | CL:0000451 | Spleen | 5.14E-07 |
| DR-GORD | TS_Skin_endothelial cell | Adult | Integumentary system | Endothelial cell | CL:0000115 | Skin of body | 4.30E-07 |
| DR-GORD | TS_Thymus_innate lymphoid cell | Adult | Lymphatic system | Innate lymphoid cell | CL:0001065 | Thymus | 2.83E-06 |
| DR-GORD | TS_Bladder_capillary endothelial cell | Adult | Urinary system | Capillary endothelial cell | CL:0002144 | Bladder organ | 6.08E-07 |
| DR-GORD | TS_Salivary_Gland_monocyte | Adult | Digestive system | Monocyte | CL:0000576 | Saliva-secreting gland | 7.21E-07 |
| DR-GORD | TS_Trachea_b cell | Adult | Respiratory system | B cell | CL:0000236 | Trachea | 9.20E-06 |
| DR-GORD | TS_Pancreas_myeloid cell | Adult | Digestive system | Myeloid cell | CL:0000763 | Pancreas | 6.10E-07 |
| DR-GORD | TS_Lymph_Node_classical monocyte | Adult | Lymphatic system | Monocyte | CL:0000576 | Lymph node | 2.43E-06 |
| DR-GORD | TS_Bone_Marrow_monocyte | Adult | Lymphatic system | Monocyte | CL:0000576 | Bone marrow | 2.43E-06 |
| DR-GORD | TS_Lymph_Node_cd1c-positive myeloid dendritic cell | Adult | Lymphatic system | Dendritic cell | CL:0000451 | Lymph node | 2.43E-06 |
| DR-GORD | TS_Lymph_Node_intermediate monocyte | Adult | Lymphatic system | Monocyte | CL:0000576 | Lymph node | 2.43E-06 |
| DR-GORD | TS_Spleen_classical monocyte | Adult | Lymphatic system | Monocyte | CL:0000576 | Spleen | 2.43E-06 |
| DR-GORD | TS_Vasculature_vein or capillary endothelial cell | Adult | Cardiovascular system | Capillary endothelial cell | CL:0002144 | Vasculature | 2.83E-06 |
| DR-GORD | TS_Eye_monocyte | Adult | Sensory system | Monocyte | CL:0000576 | Eye | 3.33E-06 |
| DR-GORD | TS_Fat_fibroblast | Adult | Integumentary system | Fibroblast | CL:0000057 | Perirenal fat | 2.83E-06 |
| DR-GORD | TS_Liver_endothelial cell | Adult | Digestive system | Endothelial cell | CL:0000115 | Liver | 6.84E-06 |
| DR-GORD | TS_Kidney_endothelial cell | Adult | Urinary system | Endothelial cell | CL:0000115 | Kidney | 2.54E-05 |
| DR-GORD | TS_Salivary_Gland_macrophage | Adult | Digestive system | Macrophage | CL:0000235 | Saliva-secreting gland | 3.32E-06 |
| DR-GORD | TS_Bladder_macrophage | Adult | Urinary system | Macrophage | CL:0000235 | Bladder organ | 2.84E-06 |
| DR-GORD | TS_Liver_monocyte | Adult | Digestive system | Monocyte | CL:0000576 | Liver | 6.84E-06 |
| DR-GORD | TS_Tongue_epithelial cell | Adult | Digestive system | Epithelial cell | CL:0000066 | Tongue | 3.32E-06 |
| DR-GORD | TS_Pancreas_nk cell | Adult | Digestive system | Natural killer cell | CL:0000623 | Pancreas | 1.24E-05 |
| DR-GORD | TS_Salivary_Gland_naive b cell | Adult | Digestive system | B cell | CL:0000236 | Saliva-secreting gland | 1.43E-05 |
| DR-GORD | TS_Prostate_macrophage | Adult | Reproductive system | Macrophage | CL:0000235 | Prostate gland | 1.23E-05 |
| DR-GORD | TS_Muscle_endothelial cell of artery | Adult | Muscular system | Endothelial cell | CL:0000115 | Muscle organ | 3.86E-06 |
| DR-GORD | TS_Lymph_Node_cd141-positive myeloid dendritic cell | Adult | Lymphatic system | Dendritic cell | CL:0000451 | Lymph node | 1.07E-05 |
| DR-GORD | TS_Muscle_mesenchymal stem cell | Adult | Muscular system | Mesenchymal stem cell | CL:0000134 | Muscle organ | 3.86E-06 |
| DR-GORD | TS_Blood_memory b cell | Adult | Lymphatic system | B cell | CL:0000236 | Blood | 1.07E-05 |
| DR-GORD | TS_Thymus_naive regulatory t cell | Adult | Lymphatic system | T cell | CL:0000084 | Thymus | 1.23E-05 |
| DR-GORD | TS_Lymph_Node_plasma cell | Adult | Lymphatic system | Plasma cell | CL:0000786 | Lymph node | 1.07E-05 |
| DR-GORD | TS_Blood_cd8-positive, alpha-beta cytokine secreting effector t cell | Adult | Lymphatic system | T cell | CL:0000084 | Blood | 1.07E-05 |
| DR-GORD | TS_Salivary_Gland_fibroblast | Adult | Digestive system | Fibroblast | CL:0000057 | Saliva-secreting gland | 1.43E-05 |
| DR-GORD | TS_Muscle_macrophage | Adult | Muscular system | Macrophage | CL:0000235 | Muscle organ | 1.64E-05 |
| DR-GORD | TS_Muscle_endothelial cell of vascular tree | Adult | Muscular system | Endothelial cell | CL:0000115 | Muscle organ | 1.64E-05 |
| DR-GORD | TS_Eye_corneal keratocyte | Adult | Sensory system | Fibroblast | CL:0000057 | Eye | 1.43E-05 |
| DR-IBD | Fetal_HCA_Liver_Lymphoid cells | Fetal | Digestive system | Innate lymphoid cell | CL:0001065 | Liver | 2.31E-07 |
| DR-IBD | TS_Pancreas_endothelial cell | Adult | Digestive system | Endothelial cell | CL:0000115 | Pancreas | 4.03E-11 |
| DR-IBD | TS_Lung_dendritic cell | Adult | Respiratory system | Dendritic cell | CL:0000451 | Lung | 6.72E-09 |
| DR-IBD | AdultKidney_Endothelial cell (APC) | Adult | Urinary system | Endothelial cell | CL:0000115 | Kidney | 7.16E-07 |
| DR-IBD | TS_Lymph_Node_b cell | Adult | Lymphatic system | B cell | CL:0000236 | Lymph node | 2.13E-10 |
| DR-IBD | AdultRectum_B cell | Adult | Digestive system | B cell | CL:0000236 | Rectum | 8.12E-07 |
| DR-IBD | AdultProstate_Endothelial cell (APC) | Adult | Reproductive system | Endothelial cell | CL:0000115 | Prostate gland | 3.50E-06 |
| DR-IBD | TS_Kidney_b cell | Adult | Urinary system | B cell | CL:0000236 | Kidney | 6.24E-07 |
| DR-IBD | Fetal_HCA_Lung_Lymphoid cells | Fetal | Respiratory system | Innate lymphoid cell | CL:0001065 | Lung | 5.01E-07 |
| DR-IBD | Madissoon_2020_oesophagus_B_CD27pos | Adult | Digestive system | B cell | CL:0000236 | Esophagus | 8.47E-07 |
| DR-IBD | TS_Bone_Marrow_memory b cell | Adult | Lymphatic system | B cell | CL:0000236 | Bone marrow | 1.03E-08 |
| DR-IBD | TS_Bladder_capillary endothelial cell | Adult | Urinary system | Capillary endothelial cell | CL:0002144 | Bladder organ | 3.42E-10 |
| DR-IBD | Madissoon_2020_spleen_B_mature | Adult | Lymphatic system | B cell | CL:0000236 | Spleen | 1.36E-05 |
| DR-IBD | Madissoon_2020_oesophagus_B_CD27neg | Adult | Digestive system | B cell | CL:0000236 | Esophagus | 4.09E-06 |
| DR-IBD | Lake_2017_CerebellarHemisphere_Mic | Adult | Nervous system | Microglial cell | CL:0000129 | Cerebellar hemisphere | 8.73E-06 |
| DR-IBD | TS_Kidney_macrophage | Adult | Urinary system | Macrophage | CL:0000235 | Kidney | 3.43E-09 |
| DR-IBD | TS_Eye_b cell | Adult | Sensory system | B cell | CL:0000236 | Eye | 1.77E-06 |
| DR-IBD | Fetal_HCA_Heart_Myeloid cells | Fetal | Cardiovascular system | Myeloid cell | CL:0000763 | Heart | 5.20E-06 |
| DR-IBD | TS_Trachea_b cell | Adult | Respiratory system | B cell | CL:0000236 | Trachea | 1.25E-08 |
| DR-IBD | TS_Muscle_endothelial cell of artery | Adult | Muscular system | Endothelial cell | CL:0000115 | Muscle organ | 3.43E-10 |
| DR-IBD | FetalMuscle_Dendritic cell | Fetal | Muscular system | Dendritic cell | CL:0000451 | Muscle organ | 3.12E-06 |
| DR-IBD | AdultSigmoidColon_B cell | Adult | Digestive system | B cell | CL:0000236 | Sigmoid colon | 2.70E-05 |
| DR-IBD | FetalIntestine_Dendritic cell | Fetal | Digestive system | Dendritic cell | CL:0000451 | Intestine | 3.70E-06 |
| DR-IBD | TS_Tongue_immune cell | Adult | Digestive system | Immune cell | NA | Tongue | 1.03E-08 |
| DR-IBD | TS_Lymph_Node_memory b cell | Adult | Lymphatic system | B cell | CL:0000236 | Lymph node | 2.54E-07 |
| DR-IBD | FetalLiver_Dendritic cell | Fetal | Digestive system | Dendritic cell | CL:0000451 | Liver | 4.48E-06 |
| DR-IBD | TS_Salivary_Gland_memory b cell | Adult | Digestive system | B cell | CL:0000236 | Saliva-secreting gland | 1.72E-09 |
| DR-IBD | AdultStomach_Macrophage | Adult | Digestive system | Macrophage | CL:0000235 | Stomach | 1.10E-05 |
| DR-IBD | FetalThymus_Dendritic cell | Fetal | Lymphatic system | Dendritic cell | CL:0000451 | Thymus | 1.94E-05 |
| DR-IBD | TS_Lung_macrophage | Adult | Respiratory system | Macrophage | CL:0000235 | Lung | 1.09E-09 |
| DR-IBD | TS_Salivary_Gland_naive b cell | Adult | Digestive system | B cell | CL:0000236 | Saliva-secreting gland | 1.03E-08 |
| DR-IBD | Madissoon_2020_oesophagus_Dendritic_Cells | Adult | Digestive system | Dendritic cell | CL:0000451 | Esophagus | 1.84E-05 |
| DR-IBD | FetalKidney_Macrophage | Fetal | Urinary system | Macrophage | CL:0000235 | Kidney | 1.53E-05 |
| DR-IBD | NeonatalAdrenalGland_Dendritic cell | Neonatal | Endocrine system | Dendritic cell | CL:0000451 | Adrenal gland | 2.72E-05 |
| DR-IBD | TS_Eye_microglial cell | Adult | Sensory system | Microglial cell | CL:0000129 | Eye | 1.27E-08 |
| DR-IBD | TS_Thymus_b cell | Adult | Lymphatic system | B cell | CL:0000236 | Thymus | 3.88E-08 |
| DR-IBD | FetalHeart_Macrophage | Fetal | Cardiovascular system | Macrophage | CL:0000235 | Heart | 2.72E-05 |
| DR-IBD | TS_Pancreas_myeloid cell | Adult | Digestive system | Myeloid cell | CL:0000763 | Pancreas | 1.03E-08 |
| DR-IBD | TS_Prostate_myeloid cell | Adult | Reproductive system | Myeloid cell | CL:0000763 | Prostate gland | 4.72E-08 |
| DR-IBD | TS_Large_Intestine_b cell | Adult | Digestive system | B cell | CL:0000236 | Large intestine | 6.96E-08 |
| DR-IBD | TS_Liver_macrophage | Adult | Digestive system | Macrophage | CL:0000235 | Liver | 4.72E-08 |
| DR-IBD | TS_Eye_retinal blood vessel endothelial cell | Adult | Sensory system | Endothelial cell | CL:0000115 | Eye | 1.27E-08 |
| DR-IBD | TS_Eye_monocyte | Adult | Sensory system | Monocyte | CL:0000576 | Eye | 7.02E-08 |
| DR-IBD | TS_Small_Intestine_b cell | Adult | Digestive system | B cell | CL:0000236 | Small intestine | 6.98E-08 |
| DR-IBD | TS_Thymus_innate lymphoid cell | Adult | Lymphatic system | Innate lymphoid cell | CL:0001065 | Thymus | 1.06E-06 |
| DR-IBD | TS_Trachea_endothelial cell | Adult | Respiratory system | Endothelial cell | CL:0000115 | Trachea | 3.60E-07 |
| DR-IBD | TS_Uterus_endothelial cell | Adult | Reproductive system | Endothelial cell | CL:0000115 | Uterus | 2.09E-07 |
| DR-IBD | TS_Skin_endothelial cell | Adult | Integumentary system | Endothelial cell | CL:0000115 | Skin of body | 1.74E-07 |
| DR-IBD | TS_Bladder_b cell | Adult | Urinary system | B cell | CL:0000236 | Bladder organ | 7.97E-06 |
| DR-IBD | TS_Salivary_Gland_endothelial cell | Adult | Digestive system | Endothelial cell | CL:0000115 | Saliva-secreting gland | 3.04E-07 |
| DR-IBD | TS_Blood_memory b cell | Adult | Lymphatic system | B cell | CL:0000236 | Blood | 3.04E-07 |
| DR-IBD | TS_Skin_cd1c-positive myeloid dendritic cell | Adult | Integumentary system | Myeloid cell | CL:0000763 | Skin of body | 1.91E-05 |
| DR-IBD | TS_Spleen_cd141-positive myeloid dendritic cell | Adult | Lymphatic system | Dendritic cell | CL:0000451 | Spleen | 3.63E-07 |
| DR-IBD | TS_Pancreas_nk cell | Adult | Digestive system | Natural killer cell | CL:0000623 | Pancreas | 1.50E-06 |
| DR-IBD | TS_Thymus_memory b cell | Adult | Lymphatic system | B cell | CL:0000236 | Thymus | 2.21E-05 |
| DR-IBD | TS_Lung_capillary aerocyte | Adult | Respiratory system | Alveolar capillary type 1 endothelial cell | CL:4028002 | Lung | 2.21E-05 |
| DR-IBD | TS_Liver_monocyte | Adult | Digestive system | Monocyte | CL:0000576 | Liver | 1.26E-06 |
| DR-IBD | TS_Thymus_cd8-positive, alpha-beta cytotoxic t cell | Adult | Lymphatic system | T cell | CL:0000084 | Thymus | 1.06E-06 |
| DR-IBD | VentoTormo_2018_decidua_monocyte | Maternal-Fetal | Reproductive system | Monocyte | CL:0000576 | Decidua | 7.87E-06 |
| DR-IBD | TS_Prostate_endothelial cell | Adult | Reproductive system | Endothelial cell | CL:0000115 | Prostate gland | 1.26E-06 |
| DR-IBD | TS_Thymus_dendritic cell | Adult | Lymphatic system | Dendritic cell | CL:0000451 | Thymus | 5.03E-06 |
| DR-IBD | TS_Bone_Marrow_macrophage | Adult | Lymphatic system | Macrophage | CL:0000235 | Bone marrow | 1.49E-06 |
| DR-IBD | TS_Uterus_macrophage | Adult | Reproductive system | Macrophage | CL:0000235 | Uterus | 5.01E-06 |
| DR-IBD | TS_Mammary_macrophage | Adult | Endocrine system | Macrophage | CL:0000235 | Mammary gland | 2.20E-05 |
| DR-IBD | TS_Spleen_memory b cell | Adult | Lymphatic system | B cell | CL:0000236 | Spleen | 1.76E-06 |
| DR-IBD | TS_Bladder_macrophage | Adult | Urinary system | Macrophage | CL:0000235 | Bladder organ | 1.76E-06 |
| DR-IBD | TS_Mammary_endothelial cell of lymphatic vessel | Adult | Endocrine system | Endothelial cell | CL:0000115 | Mammary gland | 2.20E-05 |
| DR-IBD | TS_Heart_cardiac endothelial cell | Adult | Cardiovascular system | Endothelial cell | CL:0000115 | Heart | 6.81E-06 |
| DR-IBD | TS_Liver_liver dendritic cell | Adult | Digestive system | Dendritic cell | CL:0000451 | Liver | 2.54E-05 |
| DR-IBD | TS_Blood_classical monocyte | Adult | Lymphatic system | Monocyte | CL:0000576 | Blood | 1.50E-06 |
| DR-IBD | TS_Mammary_vein endothelial cell | Adult | Endocrine system | Endothelial cell | CL:0000115 | Mammary gland | 5.02E-06 |
| DR-IBD | TS_Salivary_Gland_monocyte | Adult | Digestive system | Monocyte | CL:0000576 | Saliva-secreting gland | 6.87E-06 |
| DR-IBD | TS_Muscle_capillary endothelial cell | Adult | Muscular system | Capillary endothelial cell | CL:0002144 | Muscle organ | 7.98E-06 |
| DR-IBD | TS_Thymus_endothelial cell of artery | Adult | Lymphatic system | Endothelial cell | CL:0000115 | Thymus | 5.03E-06 |
| DR-IBD | TS_Salivary_Gland_macrophage | Adult | Digestive system | Macrophage | CL:0000235 | Saliva-secreting gland | 6.87E-06 |
| DR-IBD | TS_Trachea_cd8-positive, alpha-beta t cell | Adult | Respiratory system | T cell | CL:0000084 | Trachea | 3.35E-05 |
| DR-IBD | TS_Eye_macrophage | Adult | Sensory system | Macrophage | CL:0000235 | Eye | 8.01E-06 |
| DR-IBD | TS_Trachea_macrophage | Adult | Respiratory system | Macrophage | CL:0000235 | Trachea | 7.93E-06 |
| DR-IBD | TS_Mammary_endothelial cell of artery | Adult | Endocrine system | Endothelial cell | CL:0000115 | Mammary gland | 2.20E-05 |
| DR-IBD | TS_Blood_cd8-positive, alpha-beta cytokine secreting effector t cell | Adult | Lymphatic system | T cell | CL:0000084 | Blood | 6.88E-06 |
| DR-IBD | TS_Thymus_capillary endothelial cell | Adult | Lymphatic system | Capillary endothelial cell | CL:0002144 | Thymus | 2.21E-05 |
| DR-IBD | TS_Prostate_macrophage | Adult | Reproductive system | Macrophage | CL:0000235 | Prostate gland | 2.54E-05 |
| DR-IBD | TS_Blood_naive b cell | Adult | Lymphatic system | B cell | CL:0000236 | Blood | 2.94E-05 |
| DR-IBD | TS_Thymus_naive regulatory t cell | Adult | Lymphatic system | T cell | CL:0000084 | Thymus | 2.21E-05 |
| DR-IBD | TS_Vasculature_artery endothelial cell | Adult | Cardiovascular system | Endothelial cell | CL:0000115 | Vasculature | 2.93E-05 |
| DR-IBD | TS_Tongue_capillary endothelial cell | Adult | Digestive system | Capillary endothelial cell | CL:0002144 | Tongue | 2.94E-05 |
| DR-IBD | TS_Thymus_vein endothelial cell | Adult | Lymphatic system | Endothelial cell | CL:0000115 | Thymus | 2.21E-05 |
| DR-IBD | TS_Blood_macrophage | Adult | Lymphatic system | Macrophage | CL:0000235 | Blood | 2.94E-05 |
| DR-IBD | TS_Salivary_Gland_endothelial cell of lymphatic vessel | Adult | Digestive system | Endothelial cell | CL:0000115 | Saliva-secreting gland | 2.94E-05 |
| DR-IBD | TS_Fat_endothelial cell | Adult | Integumentary system | Endothelial cell | CL:0000115 | Perirenal fat | 2.93E-05 |
| DR-IBD | TS_Large_Intestine_cd8-positive, alpha-beta t cell | Adult | Digestive system | T cell | CL:0000084 | Large intestine | 3.36E-05 |
| DR-IBD | TS_Blood_nk cell | Adult | Lymphatic system | Natural killer cell | CL:0000623 | Blood | 2.94E-05 |
| DR-IBS | Braga_2020_lung_Macrophages | Adult | Respiratory system | Macrophage | CL:0000235 | Lung | 2.58E-06 |
| DR-IBS | Fetal_HCA_Heart_Myeloid cells | Fetal | Cardiovascular system | Myeloid cell | CL:0000763 | Heart | 1.62E-07 |
| DR-IBS | TS_Lung_macrophage | Adult | Respiratory system | Macrophage | CL:0000235 | Lung | 2.51E-15 |
| DR-IBS | FetalMuscle_Dendritic cell | Fetal | Muscular system | Dendritic cell | CL:0000451 | Muscle organ | 1.12E-07 |
| DR-IBS | TS_Liver_macrophage | Adult | Digestive system | Macrophage | CL:0000235 | Liver | 4.83E-12 |
| DR-IBS | AdultBoneMarrow_Antigen presenting cell (RPS high) | Adult | Lymphatic system | Professional antigen presenting cell | CL:0000145 | Bone marrow | 1.23E-06 |
| DR-IBS | FetalLiver_CB CD34+ | Fetal | Digestive system | Hematopoietic stem cell | CL:0000037 | Liver | 1.08E-05 |
| DR-IBS | Madissoon_2020_lung_Macrophage_MARCOneg | Adult | Respiratory system | Melanocyte | CL:0000148 | Lung | 3.17E-06 |
| DR-IBS | MacParland_2018_liver_Kupffer cell | Adult | Digestive system | Kupffer cell | CL:0000091 | Liver | 6.47E-06 |
| DR-IBS | AdultLung_B cell | Adult | Respiratory system | B cell | CL:0000236 | Lung | 1.59E-05 |
| DR-IBS | AdultFallopiantube_Dendritic cell | Adult | Reproductive system | Dendritic cell | CL:0000451 | Fallopian tube | 2.09E-06 |
| DR-IBS | Madissoon_2020_oesophagus_B_CD27pos | Adult | Digestive system | B cell | CL:0000236 | Esophagus | 5.72E-07 |
| DR-IBS | AdultPleura_Macrophage | Adult | Respiratory system | Macrophage | CL:0000235 | Pleura | 1.48E-06 |
| DR-IBS | TS_Bladder_macrophage | Adult | Urinary system | Macrophage | CL:0000235 | Bladder organ | 5.34E-11 |
| DR-IBS | Madissoon_2020_oesophagus_B_CD27neg | Adult | Digestive system | B cell | CL:0000236 | Esophagus | 2.69E-06 |
| DR-IBS | FetalLiver_Dendritic cell | Fetal | Digestive system | Dendritic cell | CL:0000451 | Liver | 3.87E-07 |
| DR-IBS | AdultArtery_Macrophage | Adult | Cardiovascular system | Macrophage | CL:0000235 | Artery | 2.12E-06 |
| DR-IBS | FetalIntestine_Dendritic cell | Fetal | Digestive system | Dendritic cell | CL:0000451 | Intestine | 7.06E-07 |
| DR-IBS | AdultRectum_B cell | Adult | Digestive system | B cell | CL:0000236 | Rectum | 3.09E-06 |
| DR-IBS | AdultBoneMarrow_Dendritic cell | Adult | Lymphatic system | Dendritic cell | CL:0000451 | Bone marrow | 6.11E-06 |
| DR-IBS | AdultEsophagus_Macrophage | Adult | Digestive system | Macrophage | CL:0000235 | Esophagus | 6.19E-06 |
| DR-IBS | AdultEsophagus_Dendritic cell | Adult | Digestive system | Dendritic cell | CL:0000451 | Esophagus | 6.19E-06 |
| DR-IBS | FetalStomach_Dendritic cell | Fetal | Digestive system | Dendritic cell | CL:0000451 | Stomach | 3.71E-06 |
| DR-IBS | TS_Tongue_immune cell | Adult | Digestive system | Immune cell | NA | Tongue | 9.22E-09 |
| DR-IBS | AdultLung_M2 Macrophage | Adult | Respiratory system | Macrophage | CL:0000235 | Lung | 3.49E-06 |
| DR-IBS | TS_Large_Intestine_b cell | Adult | Digestive system | B cell | CL:0000236 | Large intestine | 1.61E-09 |
| DR-IBS | TS_Pancreas_myeloid cell | Adult | Digestive system | Myeloid cell | CL:0000763 | Pancreas | 2.70E-10 |
| DR-IBS | van_Zyl_eye_2020_Macrophage | Adult | Sensory system | Macrophage | CL:0000235 | Eye | 1.03E-05 |
| DR-IBS | TS_Skin_langerhans cell | Adult | Integumentary system | Langerhans cell | CL:0000453 | Skin of body | 4.06E-08 |
| DR-IBS | AdultBladder_Dendritic cell | Adult | Urinary system | Dendritic cell | CL:0000451 | Bladder organ | 2.82E-05 |
| DR-IBS | AdultEsophagus_B cell | Adult | Digestive system | B cell | CL:0000236 | Esophagus | 6.19E-06 |
| DR-IBS | FetalLiver_B cell | Fetal | Digestive system | B cell | CL:0000236 | Liver | 1.08E-05 |
| DR-IBS | AdultStomach_Dendritic cell | Adult | Digestive system | Dendritic cell | CL:0000451 | Stomach | 8.57E-06 |
| DR-IBS | AdultHeart_Dendritic cell | Adult | Cardiovascular system | Dendritic cell | CL:0000451 | Heart | 2.70E-05 |
| DR-IBS | Madissoon_2020_lung_DC_2 | Adult | Respiratory system | Dendritic cell | CL:0000451 | Lung | 1.37E-05 |
| DR-IBS | VentoTormo_2018_decidua_monocyte | Maternal-Fetal | Reproductive system | Monocyte | CL:0000576 | Decidua | 5.77E-08 |
| DR-IBS | TS_Blood_classical monocyte | Adult | Lymphatic system | Monocyte | CL:0000576 | Blood | 2.70E-10 |
| DR-IBS | AdultStomach_Macrophage | Adult | Digestive system | Macrophage | CL:0000235 | Stomach | 8.57E-06 |
| DR-IBS | TS_Prostate_myeloid cell | Adult | Reproductive system | Myeloid cell | CL:0000763 | Prostate gland | 7.48E-09 |
| DR-IBS | FetalThymus_Dendritic cell | Fetal | Lymphatic system | Dendritic cell | CL:0000451 | Thymus | 1.11E-05 |
| DR-IBS | VentoTormo_2018_decidua_decidual macrophage (dM2) | Maternal-Fetal | Reproductive system | Macrophage | CL:0000235 | Decidua | 5.77E-08 |
| DR-IBS | AdultLung_Dendritic cell | Adult | Respiratory system | Dendritic cell | CL:0000451 | Lung | 1.59E-05 |
| DR-IBS | NeonatalAdrenalGland_Macrophage | Neonatal | Endocrine system | Macrophage | CL:0000235 | Adrenal gland | 1.79E-05 |
| DR-IBS | NeonatalAdrenalGland_Dendritic cell | Neonatal | Endocrine system | Dendritic cell | CL:0000451 | Adrenal gland | 1.79E-05 |
| DR-IBS | FetalPancreas_Dendritic cell | Fetal | Digestive system | Dendritic cell | CL:0000451 | Pancreas | 1.36E-05 |
| DR-IBS | TS_Trachea_macrophage | Adult | Respiratory system | Macrophage | CL:0000235 | Trachea | 9.09E-09 |
| DR-IBS | TS_Bone_Marrow_macrophage | Adult | Lymphatic system | Macrophage | CL:0000235 | Bone marrow | 9.18E-09 |
| DR-IBS | Fetal_HCA_Pancreas_Myeloid cells | Fetal | Digestive system | Myeloid cell | CL:0000763 | Pancreas | 2.11E-05 |
| DR-IBS | FetalKidney_Macrophage | Fetal | Urinary system | Macrophage | CL:0000235 | Kidney | 1.84E-05 |
| DR-IBS | AdultCerebellum_Macrophage | Adult | Nervous system | Microglial cell | CL:0000129 | Cerebellum | 2.85E-05 |
| DR-IBS | TS_Trachea_b cell | Adult | Respiratory system | B cell | CL:0000236 | Trachea | 2.46E-07 |
| DR-IBS | TS_Eye_microglial cell | Adult | Sensory system | Microglial cell | CL:0000129 | Eye | 5.94E-08 |
| DR-IBS | TS_Bone_Marrow_memory b cell | Adult | Lymphatic system | B cell | CL:0000236 | Bone marrow | 2.48E-07 |
| DR-IBS | TS_Eye_b cell | Adult | Sensory system | B cell | CL:0000236 | Eye | 2.47E-05 |
| DR-IBS | FetalHeart_Macrophage | Fetal | Cardiovascular system | Macrophage | CL:0000235 | Heart | 2.84E-05 |
| DR-IBS | TS_Small_Intestine_b cell | Adult | Digestive system | B cell | CL:0000236 | Small intestine | 4.91E-08 |
| DR-IBS | TS_Spleen_cd141-positive myeloid dendritic cell | Adult | Lymphatic system | Dendritic cell | CL:0000451 | Spleen | 4.92E-08 |
| DR-IBS | TS_Blood_monocyte | Adult | Lymphatic system | Monocyte | CL:0000576 | Blood | 9.22E-09 |
| DR-IBS | TS_Eye_monocyte | Adult | Sensory system | Monocyte | CL:0000576 | Eye | 5.94E-08 |
| DR-IBS | TS_Thymus_b cell | Adult | Lymphatic system | B cell | CL:0000236 | Thymus | 2.47E-07 |
| DR-IBS | TS_Prostate_macrophage | Adult | Reproductive system | Macrophage | CL:0000235 | Prostate gland | 2.07E-07 |
| DR-IBS | TS_Uterus_macrophage | Adult | Reproductive system | Macrophage | CL:0000235 | Uterus | 2.46E-07 |
| DR-IBS | TS_Lymph_Node_b cell | Adult | Lymphatic system | B cell | CL:0000236 | Lymph node | 2.09E-07 |
| DR-IBS | TS_Lymph_Node_cd1c-positive myeloid dendritic cell | Adult | Lymphatic system | Dendritic cell | CL:0000451 | Lymph node | 2.09E-07 |
| DR-IBS | TS_Lung_dendritic cell | Adult | Respiratory system | Dendritic cell | CL:0000451 | Lung | 6.01E-06 |
| DR-IBS | TS_Eye_macrophage | Adult | Sensory system | Macrophage | CL:0000235 | Eye | 2.95E-07 |
| DR-IBS | TS_Liver_liver dendritic cell | Adult | Digestive system | Dendritic cell | CL:0000451 | Liver | 4.47E-06 |
| DR-IBS | TS_Liver_endothelial cell | Adult | Digestive system | Endothelial cell | CL:0000115 | Liver | 9.95E-07 |
| DR-IBS | TS_Salivary_Gland_macrophage | Adult | Digestive system | Macrophage | CL:0000235 | Saliva-secreting gland | 2.94E-07 |
| DR-IBS | TS_Bone_Marrow_granulocyte | Adult | Lymphatic system | Granulocyte | CL:0000094 | Bone marrow | 2.48E-07 |
| DR-IBS | TS_Mammary_macrophage | Adult | Endocrine system | Macrophage | CL:0000235 | Mammary gland | 5.98E-06 |
| DR-IBS | TS_Spleen_macrophage | Adult | Lymphatic system | Macrophage | CL:0000235 | Spleen | 2.48E-07 |
| DR-IBS | TS_Spleen_classical monocyte | Adult | Lymphatic system | Monocyte | CL:0000576 | Spleen | 2.48E-07 |
| DR-IBS | TS_Spleen_cd1c-positive myeloid dendritic cell | Adult | Lymphatic system | Dendritic cell | CL:0000451 | Spleen | 1.17E-06 |
| DR-IBS | TS_Eye_retinal blood vessel endothelial cell | Adult | Sensory system | Endothelial cell | CL:0000115 | Eye | 2.95E-07 |
| DR-IBS | TS_Lymph_Node_intermediate monocyte | Adult | Lymphatic system | Monocyte | CL:0000576 | Lymph node | 1.00E-06 |
| DR-IBS | VentoTormo_2018_decidua_decidual macrophage (dM1) | Maternal-Fetal | Reproductive system | Macrophage | CL:0000235 | Decidua | 5.92E-06 |
| DR-IBS | TS_Kidney_macrophage | Adult | Urinary system | Macrophage | CL:0000235 | Kidney | 3.83E-06 |
| DR-IBS | TS_Skin_endothelial cell | Adult | Integumentary system | Endothelial cell | CL:0000115 | Skin of body | 9.94E-07 |
| DR-IBS | TS_Blood_macrophage | Adult | Lymphatic system | Macrophage | CL:0000235 | Blood | 1.18E-06 |
| DR-IBS | TS_Small_Intestine_neutrophil | Adult | Digestive system | Neutrophil | CL:0000775 | Small intestine | 5.19E-06 |
| DR-IBS | TS_Muscle_macrophage | Adult | Muscular system | Macrophage | CL:0000235 | Muscle organ | 1.37E-06 |
| DR-IBS | TS_Pancreas_plasma cell | Adult | Digestive system | Plasma cell | CL:0000786 | Pancreas | 5.21E-06 |
| DR-IBS | TS_Pancreas_endothelial cell | Adult | Digestive system | Endothelial cell | CL:0000115 | Pancreas | 1.17E-06 |
| DR-IBS | TS_Liver_monocyte | Adult | Digestive system | Monocyte | CL:0000576 | Liver | 4.47E-06 |
| DR-IBS | TS_Salivary_Gland_naive b cell | Adult | Digestive system | B cell | CL:0000236 | Saliva-secreting gland | 6.01E-06 |
| DR-IBS | TS_Prostate_endothelial cell | Adult | Reproductive system | Endothelial cell | CL:0000115 | Prostate gland | 4.47E-06 |
| DR-IBS | TS_Lymph_Node_cd141-positive myeloid dendritic cell | Adult | Lymphatic system | Dendritic cell | CL:0000451 | Lymph node | 4.49E-06 |
| DR-IBS | VentoTormo_2018_decidua_Hofbauer cell (HB) | Maternal-Fetal | Reproductive system | Hofbauer cell | CL:3000001 | Decidua | 2.43E-05 |
| DR-IBS | TS_Thymus_innate lymphoid cell | Adult | Lymphatic system | Innate lymphoid cell | CL:0001065 | Thymus | 2.15E-05 |
| DR-IBS | TS_Blood_memory b cell | Adult | Lymphatic system | B cell | CL:0000236 | Blood | 5.21E-06 |
| DR-IBS | TS_Lymph_Node_mature nk t cell | Adult | Lymphatic system | Natural killer cell | CL:0000623 | Lymph node | 1.88E-05 |
| DR-IBS | TS_Bone_Marrow_monocyte | Adult | Lymphatic system | Monocyte | CL:0000576 | Bone marrow | 5.19E-06 |
| DR-IBS | TS_Salivary_Gland_monocyte | Adult | Digestive system | Monocyte | CL:0000576 | Saliva-secreting gland | 6.01E-06 |
| DR-IBS | TS_Salivary_Gland_memory b cell | Adult | Digestive system | B cell | CL:0000236 | Saliva-secreting gland | 6.01E-06 |
| DR-IBS | TS_Tongue_epithelial cell | Adult | Digestive system | Epithelial cell | CL:0000066 | Tongue | 5.21E-06 |
| DR-IBS | TS_Skin_macrophage | Adult | Integumentary system | Macrophage | CL:0000235 | Skin of body | 1.87E-05 |
| DR-IBS | TS_Trachea_endothelial cell | Adult | Respiratory system | Endothelial cell | CL:0000115 | Trachea | 2.14E-05 |
| DR-IBS | TS_Lymph_Node_classical monocyte | Adult | Lymphatic system | Monocyte | CL:0000576 | Lymph node | 1.88E-05 |
| DR-IBS | TS_Large_Intestine_gut endothelial cell | Adult | Digestive system | Endothelial cell | CL:0000115 | Large intestine | 2.15E-05 |
| DR-IBS | TS_Thymus_cd8-positive, alpha-beta cytotoxic t cell | Adult | Lymphatic system | T cell | CL:0000084 | Thymus | 2.15E-05 |
| DR-IBS | TS_Uterus_endothelial cell | Adult | Reproductive system | Endothelial cell | CL:0000115 | Uterus | 2.14E-05 |
| DR-IBS | TS_Spleen_nk cell | Adult | Lymphatic system | Natural killer cell | CL:0000623 | Spleen | 2.15E-05 |
| DR-IBS | TS_Mammary_luminal epithelial cell of mammary gland | Adult | Endocrine system | Epithelial cell | CL:0000066 | Mammary gland | 2.45E-05 |
| DR-IBS | TS_Eye_ocular surface cell | Adult | Sensory system | Epithelial cell | CL:0000066 | Eye | 2.47E-05 |
| DR-IBS | TS_Spleen_plasma cell | Adult | Lymphatic system | Plasma cell | CL:0000786 | Spleen | 2.15E-05 |
| DR-IBS | TS_Thymus_vein endothelial cell | Adult | Lymphatic system | Endothelial cell | CL:0000115 | Thymus | 2.15E-05 |
| DR-IBS | TS_Bladder_capillary endothelial cell | Adult | Urinary system | Capillary endothelial cell | CL:0002144 | Bladder organ | 2.46E-05 |
| DR-PUD | TS_Bladder_capillary endothelial cell | Adult | Urinary system | Capillary endothelial cell | CL:0002144 | Bladder organ | 1.13E-07 |
| DR-PUD | TS_Bladder_fibroblast | Adult | Urinary system | Fibroblast | CL:0000057 | Bladder organ | 1.13E-07 |
| DR-PUD | TS_Eye_retinal blood vessel endothelial cell | Adult | Sensory system | Endothelial cell | CL:0000115 | Eye | 1.13E-07 |
| DR-PUD | TS_Thymus_capillary endothelial cell | Adult | Lymphatic system | Capillary endothelial cell | CL:0002144 | Thymus | 5.93E-07 |
| DR-PUD | TS_Muscle_endothelial cell of artery | Adult | Muscular system | Endothelial cell | CL:0000115 | Muscle organ | 1.45E-07 |
| DR-PUD | TS_Tongue_capillary endothelial cell | Adult | Digestive system | Capillary endothelial cell | CL:0002144 | Tongue | 7.49E-07 |
| DR-PUD | TS_Trachea_endothelial cell | Adult | Respiratory system | Endothelial cell | CL:0000115 | Trachea | 2.95E-06 |
| DR-PUD | TS_Eye_corneal keratocyte | Adult | Sensory system | Fibroblast | CL:0000057 | Eye | 7.49E-07 |
| DR-PUD | TS_Muscle_mesenchymal stem cell | Adult | Muscular system | Mesenchymal stem cell | CL:0000134 | Muscle organ | 9.34E-07 |
| DR-PUD | TS_Lung_capillary aerocyte | Adult | Respiratory system | Alveolar capillary type 1 endothelial cell | CL:4028002 | Lung | 2.06E-05 |
| DR-PUD | TS_Large_Intestine_gut endothelial cell | Adult | Digestive system | Endothelial cell | CL:0000115 | Large intestine | 3.66E-06 |
| DR-PUD | TS_Blood_classical monocyte | Adult | Lymphatic system | Monocyte | CL:0000576 | Blood | 2.39E-06 |
| DR-PUD | TS_Skin_endothelial cell | Adult | Integumentary system | Endothelial cell | CL:0000115 | Skin of body | 3.66E-06 |
| DR-PUD | VentoTormo_2018_decidua_decidual perivascular cell (dP1) | Maternal-Fetal | Reproductive system | Pericyte cell | CL:0000669 | Decidua | 2.95E-05 |
| DR-PUD | TS_Tongue_endothelial cell of lymphatic vessel | Adult | Digestive system | Endothelial cell | CL:0000115 | Tongue | 4.52E-06 |
| DR-PUD | TS_Thymus_vein endothelial cell | Adult | Lymphatic system | Endothelial cell | CL:0000115 | Thymus | 3.66E-06 |
| DR-PUD | TS_Prostate_endothelial cell | Adult | Reproductive system | Endothelial cell | CL:0000115 | Prostate gland | 4.50E-06 |
| DR-PUD | TS_Mammary_endothelial cell of lymphatic vessel | Adult | Endocrine system | Endothelial cell | CL:0000115 | Mammary gland | 2.47E-05 |
| DR-PUD | TS_Salivary_Gland_endothelial cell | Adult | Digestive system | Endothelial cell | CL:0000115 | Saliva-secreting gland | 4.51E-06 |
| DR-PUD | TS_Pancreas_pancreatic stellate cell | Adult | Digestive system | Pancreatic stellate cell | CL:0002410 | Pancreas | 3.67E-06 |
| DR-PUD | TS_Pancreas_endothelial cell | Adult | Digestive system | Endothelial cell | CL:0000115 | Pancreas | 3.67E-06 |
| DR-PUD | TS_Blood_type i nk t cell | Adult | Lymphatic system | T cell | CL:0000084 | Blood | 1.40E-05 |
| DR-PUD | TS_Blood_cd8-positive, alpha-beta cytokine secreting effector t cell | Adult | Lymphatic system | T cell | CL:0000084 | Blood | 1.40E-05 |
| DR-PUD | TS_Lymph_Node_endothelial cell | Adult | Lymphatic system | Endothelial cell | CL:0000115 | Lymph node | 2.06E-05 |
| DR-PUD | TS_Thymus_endothelial cell of artery | Adult | Lymphatic system | Endothelial cell | CL:0000115 | Thymus | 2.06E-05 |
| DR-PUD | TS_Thymus_fibroblast | Adult | Lymphatic system | Fibroblast | CL:0000057 | Thymus | 2.06E-05 |
| DR-PUD | TS_Vasculature_pericyte cell | Adult | Cardiovascular system | Pericyte cell | CL:0000669 | Vasculature | 2.48E-05 |
| DR-PUD | TS_Salivary_Gland_fibroblast | Adult | Digestive system | Fibroblast | CL:0000057 | Saliva-secreting gland | 2.48E-05 |
| DR-PUD | TS_Salivary_Gland_endothelial cell of lymphatic vessel | Adult | Digestive system | Endothelial cell | CL:0000115 | Saliva-secreting gland | 2.48E-05 |
| DR-PUD | TS_Eye_ocular surface cell | Adult | Sensory system | Epithelial cell | CL:0000066 | Eye | 2.48E-05 |
| DR-PUD | TS_Muscle_pericyte cell | Adult | Muscular system | Pericyte cell | CL:0000669 | Muscle organ | 2.97E-05 |
| Keratitis-DD | Voigt_2019_retina_2_Fibroblasts | Adult | Sensory system | Fibroblast | CL:0000057 | Retina | 1.10E-05 |
| Myopia-DD | AdultIleum_Stromal cell | Adult | Digestive system | Stromal cell | CL:0000499 | Ileum | 4.51E-09 |
| Myopia-DD | AdultStomach_Fibroblast | Adult | Digestive system | Fibroblast | CL:0000057 | Stomach | 1.23E-05 |
| Myopia-DD | AdultArtery_Fibroblast | Adult | Cardiovascular system | Fibroblast | CL:0000057 | Artery | 4.04E-08 |
| Myopia-DD | van_Zyl_eye_2020_Fibroblast | Adult | Sensory system | Fibroblast | CL:0000057 | Eye | 1.27E-07 |
| Myopia-DD | Fetal_HCA_Stomach_Mesothelial cells | Fetal | Digestive system | Mesothelial cell | CL:0000077 | Stomach | 2.31E-06 |
| Myopia-DD | AdultThyroid_Stromal cell | Adult | Endocrine system | Stromal cell | CL:0000499 | Thyroid gland | 7.72E-06 |
| Myopia-DD | Voigt_2019_retina_2_Fibroblasts | Adult | Sensory system | Fibroblast | CL:0000057 | Retina | 1.26E-07 |
| Myopia-DD | AdultTrachea_Fibroblast | Adult | Respiratory system | Fibroblast | CL:0000057 | Trachea | 1.41E-06 |
| Myopia-DD | Fetal_HCA_Muscle_Stromal cells | Fetal | Muscular system | Stromal cell | CL:0000499 | Muscle organ | 2.41E-07 |
| Myopia-DD | Fetal_HCA_Heart_Epicardial fat cells | Fetal | Cardiovascular system | Epicardial adipocyte | CL:1000309 | Heart | 2.32E-05 |
| Myopia-DD | AdultEsophagus_Fibroblast | Adult | Digestive system | Fibroblast | CL:0000057 | Esophagus | 1.85E-07 |
| Myopia-DD | AdultPleura_Fibroblast | Adult | Respiratory system | Fibroblast | CL:0000057 | Pleura | 4.22E-07 |
| Myopia-DD | AdultOmentum_Stromal cell | Adult | Integumentary system | Stromal cell | CL:0000499 | Omentum | 1.40E-06 |
| Myopia-DD | Braga_2020_lung_Fibroblast | Adult | Respiratory system | Fibroblast | CL:0000057 | Lung | 4.70E-06 |
| Myopia-DD | Fetal_HCA_Heart_Stromal cells | Fetal | Cardiovascular system | Stromal cell | CL:0000499 | Heart | 3.76E-06 |
| Myopia-DD | Fetal_HCA_Lung_Mesothelial cells | Fetal | Respiratory system | Mesothelial cell | CL:0000077 | Lung | 3.81E-06 |
| Myopia-DD | FetalMuscle_Fetal stromal cell | Fetal | Muscular system | Stromal cell | CL:0000499 | Muscle organ | 5.52E-07 |
| Myopia-DD | TS_Lung_adventitial cell | Adult | Respiratory system | Adventitial cell | CL:0002503 | Lung | 9.44E-07 |
| Myopia-DD | Fetal_HCA_Liver_Mesothelial cells | Fetal | Digestive system | Mesothelial cell | CL:0000077 | Liver | 3.78E-06 |
| Myopia-DD | Madissoon_2020_oesophagus_Fibroblast | Adult | Digestive system | Fibroblast | CL:0000057 | Esophagus | 4.74E-06 |
| Myopia-DD | TS_Eye_corneal keratocyte | Adult | Sensory system | Fibroblast | CL:0000057 | Eye | 1.19E-14 |
| Myopia-DD | Fetal_HCA_Liver_Stellate cells | Fetal | Digestive system | Hepatic stellate cell | CL:0000632 | Liver | 2.33E-05 |
| Myopia-DD | Fetal_HCA_Adrenal_Stromal cells | Fetal | Endocrine system | Stromal cell | CL:0000499 | Adrenal gland | 3.78E-06 |
| Myopia-DD | TS_Lung_alveolar fibroblast | Adult | Respiratory system | Fibroblast | CL:0000057 | Lung | 9.44E-07 |
| Myopia-DD | TS_Bladder_fibroblast | Adult | Urinary system | Fibroblast | CL:0000057 | Bladder organ | 1.53E-13 |
| Myopia-DD | Fetal_HCA_Intestine_Stromal cells | Fetal | Digestive system | Stromal cell | CL:0000499 | Intestine | 2.34E-05 |
| Myopia-DD | TS_Vasculature_fibroblast | Adult | Cardiovascular system | Fibroblast | CL:0000057 | Vasculature | 1.84E-12 |
| Myopia-DD | TS_Trachea_fibroblast | Adult | Respiratory system | Fibroblast | CL:0000057 | Trachea | 2.04E-11 |
| Myopia-DD | TS_Trachea_connective tissue cell | Adult | Respiratory system | Connective tissue cell | CL:0002320 | Trachea | 1.95E-09 |
| Myopia-DD | TS_Fat_fibroblast | Adult | Integumentary system | Fibroblast | CL:0000057 | Perirenal fat | 2.05E-11 |
| Myopia-DD | TS_Salivary_Gland_adventitial cell | Adult | Digestive system | Adventitial cell | CL:0002503 | Saliva-secreting gland | 1.97E-09 |
| Myopia-DD | Lake_2017_VisualCortex_Ex3a | Adult | Nervous system | Excitatory neuron | CL:0008030 | Visual cortex | 1.24E-05 |
| Myopia-DD | Fetal_HCA_Cerebrum_10k_Astrocytes | Fetal | Nervous system | Astrocyte | CL:0000127 | Cerebral hemisphere | 2.89E-05 |
| Myopia-DD | TS_Muscle_mesenchymal stem cell | Adult | Muscular system | Mesenchymal stem cell | CL:0000134 | Muscle organ | 2.06E-11 |
| Myopia-DD | TS_Tongue_fibroblast | Adult | Digestive system | Fibroblast | CL:0000057 | Tongue | 1.69E-08 |
| Myopia-DD | TS_Fat_mesenchymal stem cell | Adult | Integumentary system | Mesenchymal stem cell | CL:0000134 | Perirenal fat | 1.96E-09 |
| Myopia-DD | TS_Salivary_Gland_fibroblast | Adult | Digestive system | Fibroblast | CL:0000057 | Saliva-secreting gland | 1.97E-09 |
| Myopia-DD | TS_Liver_fibroblast | Adult | Digestive system | Fibroblast | CL:0000057 | Liver | 1.00E-07 |
| Myopia-DD | TS_Eye_fibroblast | Adult | Sensory system | Fibroblast | CL:0000057 | Eye | 1.69E-08 |
| Myopia-DD | TS_Lung_type i pneumocyte | Adult | Respiratory system | Pneumocyte | CL:0000322 | Lung | 1.69E-08 |
| Myopia-DD | TS_Thymus_fibroblast | Adult | Lymphatic system | Fibroblast | CL:0000057 | Thymus | 1.68E-08 |
| Myopia-DD | Enge_2017_Pancreas_mesenchymal cell | Adult | Digestive system | Mesenchymal stem cell | CL:0000134 | Pancreas | 3.53E-05 |
| Myopia-DD | TS_Mammary_fibroblast of breast | Adult | Endocrine system | Fibroblast | CL:0000057 | Mammary gland | 1.32E-07 |
| Myopia-DD | TS_Uterus_fibroblast | Adult | Reproductive system | Fibroblast | CL:0000057 | Uterus | 1.32E-07 |
| Myopia-DD | TS_Pancreas_fibroblast | Adult | Digestive system | Fibroblast | CL:0000057 | Pancreas | 9.46E-07 |
| Myopia-DD | TS_Prostate_fibroblast | Adult | Reproductive system | Fibroblast | CL:0000057 | Prostate gland | 9.42E-07 |
| Myopia-DD | TS_Lung_vascular associated smooth muscle cell | Adult | Respiratory system | Smooth muscle cell | CL:0000192 | Lung | 6.11E-06 |
| Myopia-DD | TS_Skin_stromal cell | Adult | Integumentary system | Stromal cell | CL:0000499 | Skin of body | 9.42E-07 |
| Myopia-DD | TS_Prostate_endothelial cell | Adult | Reproductive system | Endothelial cell | CL:0000115 | Prostate gland | 9.42E-07 |
| Myopia-DD | TS_Lung_pericyte cell | Adult | Respiratory system | Pericyte cell | CL:0000669 | Lung | 3.56E-05 |
| Myopia-DD | TS_Large_Intestine_monocyte | Adult | Digestive system | Monocyte | CL:0000576 | Large intestine | 9.42E-07 |
| Myopia-DD | TS_Small_Intestine_fibroblast | Adult | Digestive system | Fibroblast | CL:0000057 | Small intestine | 9.43E-07 |
| Myopia-DD | TS_Lymph_Node_stromal cell | Adult | Lymphatic system | Stromal cell | CL:0000499 | Lymph node | 9.46E-07 |
| Myopia-DD | TS_Large_Intestine_fibroblast | Adult | Digestive system | Fibroblast | CL:0000057 | Large intestine | 9.42E-07 |
| Myopia-DD | TS_Bladder_myofibroblast cell | Adult | Urinary system | Myofibroblast cell | CL:0000186 | Bladder organ | 9.43E-07 |
| Myopia-DD | TS_Pancreas_pancreatic ductal cell | Adult | Digestive system | Pancreatic ductal cell | CL:0002079 | Pancreas | 9.46E-07 |
| Myopia-DD | VentoTormo_2018_decidua_decidual stromal cell (dS2) | Maternal-Fetal | Reproductive system | Stromal cell | CL:0000499 | Decidua | 3.54E-05 |
| Myopia-DD | TS_Prostate_basal cell of prostate epithelium | Adult | Reproductive system | Basal cell | CL:0000646 | Prostate gland | 6.09E-06 |
| Myopia-DD | VentoTormo_2018_decidua_decidual stromal cell (dS1) | Maternal-Fetal | Reproductive system | Stromal cell | CL:0000499 | Decidua | 3.54E-05 |
| Myopia-DD | TS_Small_Intestine_monocyte | Adult | Digestive system | Monocyte | CL:0000576 | Small intestine | 6.10E-06 |
| Myopia-DD | TS_Bladder_smooth muscle cell | Adult | Urinary system | Smooth muscle cell | CL:0000192 | Bladder organ | 6.10E-06 |
| Myopia-DD | TS_Vasculature_smooth muscle cell | Adult | Cardiovascular system | Smooth muscle cell | CL:0000192 | Vasculature | 6.10E-06 |
| Myopia-DD | TS_Liver_endothelial cell of hepatic sinusoid | Adult | Digestive system | Endothelial cell | CL:0000115 | Liver | 2.93E-05 |
| Myopia-DD | TS_Eye_limbal stem cell | Adult | Sensory system | Stem cell | CL:0000034 | Eye | 6.12E-06 |
| Myopia-DD | TS_Prostate_smooth muscle cell | Adult | Reproductive system | Smooth muscle cell | CL:0000192 | Prostate gland | 3.56E-05 |
| Myopia-DD | TS_Spleen_endothelial cell | Adult | Lymphatic system | Endothelial cell | CL:0000115 | Spleen | 3.56E-05 |
| Myopia-DD | TS_Blood_nk cell | Adult | Lymphatic system | Natural killer cell | CL:0000623 | Blood | 3.57E-05 |
| Myopia-DD | TS_Pancreas_pancreatic stellate cell | Adult | Digestive system | Pancreatic stellate cell | CL:0002410 | Pancreas | 3.57E-05 |
| Myopia-GORD | AdultTransverseColon_Stromal cell | Adult | Digestive system | Stromal cell | CL:0000499 | Transverse colon | 2.26E-05 |
| Myopia-GORD | Enge_2017_Pancreas_mesenchymal cell | Adult | Digestive system | Mesenchymal stem cell | CL:0000134 | Pancreas | 2.82E-06 |
| Myopia-GORD | TS_Large_Intestine_fibroblast | Adult | Digestive system | Fibroblast | CL:0000057 | Large intestine | 1.95E-07 |
| Myopia-GORD | TS_Prostate_fibroblast | Adult | Reproductive system | Fibroblast | CL:0000057 | Prostate gland | 2.83E-06 |
| Myopia-GORD | TS_Fat_fibroblast | Adult | Integumentary system | Fibroblast | CL:0000057 | Perirenal fat | 2.83E-06 |
| Myopia-GORD | TS_Small_Intestine_fibroblast | Adult | Digestive system | Fibroblast | CL:0000057 | Small intestine | 2.83E-06 |
| Myopia-GORD | TS_Lymph_Node_stromal cell | Adult | Lymphatic system | Stromal cell | CL:0000499 | Lymph node | 2.84E-06 |
| Myopia-GORD | TS_Trachea_connective tissue cell | Adult | Respiratory system | Connective tissue cell | CL:0002320 | Trachea | 3.37E-05 |
| Myopia-GORD | TS_Trachea_fibroblast | Adult | Respiratory system | Fibroblast | CL:0000057 | Trachea | 3.37E-05 |
| Myopia-GORD | TS_Tongue_tongue muscle cell | Adult | Digestive system | Muscle cell | CL:0000187 | Tongue | 3.38E-05 |
| Myopia-GORD | TS_Salivary_Gland_adventitial cell | Adult | Digestive system | Adventitial cell | CL:0002503 | Saliva-secreting gland | 3.38E-05 |
| Myopia-GORD | TS_Fat_mesenchymal stem cell | Adult | Integumentary system | Mesenchymal stem cell | CL:0000134 | Perirenal fat | 3.37E-05 |
| Myopia-GORD | TS_Thymus_fibroblast | Adult | Lymphatic system | Fibroblast | CL:0000057 | Thymus | 3.37E-05 |
| Myopia-GORD | TS_Lymph_Node_cd8-positive alpha-beta t cell | Adult | Lymphatic system | T cell | CL:0000084 | Lymph node | 3.38E-05 |
| Myopia-GORD | TS_Bladder_myofibroblast cell | Adult | Urinary system | Myofibroblast cell | CL:0000186 | Bladder organ | 3.37E-05 |
| Myopia-GORD | TS_Salivary_Gland_fibroblast | Adult | Digestive system | Fibroblast | CL:0000057 | Saliva-secreting gland | 3.38E-05 |
| Myopia-IBD | TS_Lung_type i pneumocyte | Adult | Respiratory system | Pneumocyte | CL:0000322 | Lung | 2.11E-08 |
| Myopia-IBD | TS_Prostate_basal cell of prostate epithelium | Adult | Reproductive system | Basal cell | CL:0000646 | Prostate gland | 2.24E-06 |
| Myopia-IBD | TS_Small_Intestine_duodenum glandular cell | Adult | Digestive system | Duodenum glandular cell | CL:1001589 | Small intestine | 2.25E-06 |
| Myopia-IBD | TS_Spleen_nk cell | Adult | Lymphatic system | Natural killer cell | CL:0000623 | Spleen | 1.42E-05 |
| Myopia-IBD | TS_Pancreas_pancreatic ductal cell | Adult | Digestive system | Pancreatic ductal cell | CL:0002079 | Pancreas | 1.43E-05 |
| Myopia-IBD | TS_Bladder_bladder urothelial cell | Adult | Urinary system | Urothelial cell | CL:0000731 | Bladder organ | 1.89E-05 |
| Myopia-IBD | TS_Lung_basal cell | Adult | Respiratory system | Basal cell | CL:0000646 | Lung | 1.89E-05 |
| Myopia-IBD | TS_Lung_respiratory goblet cell | Adult | Respiratory system | Goblet cell | CL:0000160 | Lung | 1.89E-05 |
| Myopia-IBD | TS_Salivary_Gland_basal cell | Adult | Digestive system | Basal cell | CL:0000646 | Saliva-secreting gland | 1.90E-05 |
| Myopia-IBS | AdultSigmoidColon_B cell | Adult | Digestive system | B cell | CL:0000236 | Sigmoid colon | 3.62E-06 |
| Myopia-IBS | AdultCerebellum_Macrophage | Adult | Nervous system | Microglial cell | CL:0000129 | Cerebellum | 6.07E-06 |
| Myopia-IBS | AdultTemporalLobe_Macrophage | Adult | Nervous system | Microglial cell | CL:0000129 | Temporal lobe | 7.68E-06 |
| Myopia-IBS | FetalLiver_Macrophage | Fetal | Digestive system | Macrophage | CL:0000235 | Liver | 9.37E-06 |
| Myopia-IBS | FetalThymus_Dendritic cell | Fetal | Lymphatic system | Dendritic cell | CL:0000451 | Thymus | 1.02E-05 |
| Myopia-IBS | AdultPancreas_Macrophage | Adult | Digestive system | Macrophage | CL:0000235 | Pancreas | 3.17E-05 |
| Myopia-IBS | FetalLiver_Dendritic cell | Fetal | Digestive system | Dendritic cell | CL:0000451 | Liver | 9.37E-06 |
| Myopia-IBS | TS_Muscle_endothelial cell of artery | Adult | Muscular system | Endothelial cell | CL:0000115 | Muscle organ | 6.79E-08 |
| Myopia-IBS | AdultArtery_Macrophage | Adult | Cardiovascular system | Macrophage | CL:0000235 | Artery | 1.23E-05 |
| Myopia-IBS | AdultPleura_Dendritic cell | Adult | Respiratory system | Dendritic cell | CL:0000451 | Pleura | 1.52E-05 |
| Myopia-IBS | NeonatalAdrenalGland_Macrophage | Neonatal | Endocrine system | Macrophage | CL:0000235 | Adrenal gland | 2.00E-05 |
| Myopia-IBS | AdultLung_M2 Macrophage | Adult | Respiratory system | Macrophage | CL:0000235 | Lung | 1.90E-05 |
| Myopia-IBS | FetalHeart_Macrophage | Fetal | Cardiovascular system | Macrophage | CL:0000235 | Heart | 2.53E-05 |
| Myopia-IBS | FetalFemaleGonad_Macrophage | Fetal | Reproductive system | Macrophage | CL:0000235 | Immature gonad | 3.17E-05 |
| Myopia-IBS | FetalFemaleGonad_Dendritic cell | Fetal | Reproductive system | Dendritic cell | CL:0000451 | Immature gonad | 3.17E-05 |
| Myopia-IBS | TS_Tongue_epithelial cell | Adult | Digestive system | Epithelial cell | CL:0000066 | Tongue | 3.70E-07 |
| Myopia-IBS | TS_Lung_macrophage | Adult | Respiratory system | Macrophage | CL:0000235 | Lung | 4.69E-07 |
| Myopia-IBS | TS_Pancreas_endothelial cell | Adult | Digestive system | Endothelial cell | CL:0000115 | Pancreas | 2.39E-06 |
| Myopia-IBS | TS_Bone_Marrow_macrophage | Adult | Lymphatic system | Macrophage | CL:0000235 | Bone marrow | 9.34E-06 |
| Myopia-IBS | TS_Thymus_b cell | Adult | Lymphatic system | B cell | CL:0000236 | Thymus | 1.15E-05 |
| Myopia-IBS | TS_Lymph_Node_b cell | Adult | Lymphatic system | B cell | CL:0000236 | Lymph node | 9.37E-06 |
| Myopia-IBS | TS_Pancreas_myeloid cell | Adult | Digestive system | Myeloid cell | CL:0000763 | Pancreas | 1.40E-05 |
| Myopia-PUD | TS_Fat_myofibroblast cell | Adult | Integumentary system | Myofibroblast cell | CL:0000186 | Perirenal fat | 1.94E-05 |
| Myopia-PUD | TS_Vasculature_pericyte cell | Adult | Cardiovascular system | Pericyte cell | CL:0000669 | Vasculature | 1.94E-05 |
| Myopia-PUD | TS_Bladder_smooth muscle cell | Adult | Urinary system | Smooth muscle cell | CL:0000192 | Bladder organ | 1.94E-05 |
| Myopia-PUD | TS_Salivary_Gland_basal cell | Adult | Digestive system | Basal cell | CL:0000646 | Saliva-secreting gland | 1.95E-05 |
| PACG-DD | Voigt_2019_retina_2_Fibroblasts | Adult | Sensory system | Fibroblast | CL:0000057 | Retina | 1.86E-06 |
| PACG-DD | TS_Eye_corneal keratocyte | Adult | Sensory system | Fibroblast | CL:0000057 | Eye | 2.44E-07 |
| PACG-DD | TS_Bladder_smooth muscle cell | Adult | Urinary system | Smooth muscle cell | CL:0000192 | Bladder organ | 2.77E-06 |
| PACG-DD | TS_Trachea_connective tissue cell | Adult | Respiratory system | Connective tissue cell | CL:0002320 | Trachea | 1.94E-05 |
| PACG-DD | TS_Lung_vascular associated smooth muscle cell | Adult | Respiratory system | Smooth muscle cell | CL:0000192 | Lung | 2.68E-05 |
| PACG-DD | TS_Vasculature_fibroblast | Adult | Cardiovascular system | Fibroblast | CL:0000057 | Vasculature | 1.94E-05 |
| PACG-DD | TS_Large_Intestine_fibroblast | Adult | Digestive system | Fibroblast | CL:0000057 | Large intestine | 2.68E-05 |
| PACG-DD | TS_Fat_fibroblast | Adult | Integumentary system | Fibroblast | CL:0000057 | Perirenal fat | 2.68E-05 |
| PACG-DD | TS_Thymus_fibroblast | Adult | Lymphatic system | Fibroblast | CL:0000057 | Thymus | 2.68E-05 |
| PACG-DD | TS_Bladder_fibroblast | Adult | Urinary system | Fibroblast | CL:0000057 | Bladder organ | 2.68E-05 |
| PACG-IBD | Voigt_2019_retina_1_Macrophage | Adult | Sensory system | Macrophage | CL:0000235 | Retina | 5.75E-06 |
| PACG-IBD | TS_Trachea_serous cell of epithelium of trachea | Adult | Respiratory system | Serous cell of epithelium of trachea | CL:1000330 | Trachea | 5.77E-06 |
| PACG-IBD | AdultArtery_Macrophage | Adult | Cardiovascular system | Macrophage | CL:0000235 | Artery | 2.72E-06 |
| PACG-IBD | TS_Skin_cd1c-positive myeloid dendritic cell | Adult | Integumentary system | Myeloid cell | CL:0000763 | Skin of body | 5.77E-06 |
| PACG-IBD | VentoTormo_2018_decidua_decidual macrophage (dM1) | Maternal-Fetal | Reproductive system | Macrophage | CL:0000235 | Decidua | 5.76E-06 |
| PACG-IBD | TS_Tongue_immune cell | Adult | Digestive system | Immune cell | NA | Tongue | 5.78E-06 |
| PACG-IBD | TS_Skin_macrophage | Adult | Integumentary system | Macrophage | CL:0000235 | Skin of body | 5.77E-06 |
| PACG-IBD | TS_Uterus_macrophage | Adult | Reproductive system | Macrophage | CL:0000235 | Uterus | 5.77E-06 |
| PACG-IBD | TS_Muscle_macrophage | Adult | Muscular system | Macrophage | CL:0000235 | Muscle organ | 5.77E-06 |
| PACG-IBD | TS_Pancreas_myeloid cell | Adult | Digestive system | Myeloid cell | CL:0000763 | Pancreas | 5.78E-06 |
| Uveitis-DD | TS_Muscle_endothelial cell of artery | Adult | Muscular system | Endothelial cell | CL:0000115 | Muscle organ | 3.47E-07 |
| Uveitis-DD | TS_Liver_monocyte | Adult | Digestive system | Monocyte | CL:0000576 | Liver | 3.50E-06 |
| Uveitis-IBD | FetalThymus_Dendritic cell | Fetal | Lymphatic system | Dendritic cell | CL:0000451 | Thymus | 3.82E-06 |
| Uveitis-IBD | FetalSpinalCord_Dendritic cell | Fetal | Nervous system | Dendritic cell | CL:0000451 | Spinal cord | 5.48E-06 |
| Uveitis-IBD | TS_Eye_b cell | Adult | Sensory system | B cell | CL:0000236 | Eye | 4.86E-06 |
| Uveitis-IBD | AdultSigmoidColon_B cell | Adult | Digestive system | B cell | CL:0000236 | Sigmoid colon | 1.85E-05 |
| Uveitis-IBD | FetalLung_Macrophage | Fetal | Respiratory system | Macrophage | CL:0000235 | Lung | 1.40E-05 |
| Uveitis-IBD | TS_Muscle_endothelial cell of artery | Adult | Muscular system | Endothelial cell | CL:0000115 | Muscle organ | 5.57E-12 |
| Uveitis-IBD | FetalFemaleGonad_Dendritic cell | Fetal | Reproductive system | Dendritic cell | CL:0000451 | Immature gonad | 1.04E-05 |
| Uveitis-IBD | AdultRectum_B cell | Adult | Digestive system | B cell | CL:0000236 | Rectum | 1.40E-05 |
| Uveitis-IBD | FetalLung_Dendritic cell | Fetal | Respiratory system | Dendritic cell | CL:0000451 | Lung | 1.40E-05 |
| Uveitis-IBD | AdultBoneMarrow_Antigen presenting cell (RPS high) | Adult | Lymphatic system | Professional antigen presenting cell | CL:0000145 | Bone marrow | 2.43E-05 |
| Uveitis-IBD | TS_Bladder_capillary endothelial cell | Adult | Urinary system | Capillary endothelial cell | CL:0002144 | Bladder organ | 5.58E-08 |
| Uveitis-IBD | TS_Pancreas_endothelial cell | Adult | Digestive system | Endothelial cell | CL:0000115 | Pancreas | 4.85E-07 |
| Uveitis-IBD | TS_Thymus_endothelial cell of artery | Adult | Lymphatic system | Endothelial cell | CL:0000115 | Thymus | 6.46E-07 |
| Uveitis-IBD | TS_Vasculature_artery endothelial cell | Adult | Cardiovascular system | Endothelial cell | CL:0000115 | Vasculature | 3.74E-06 |
| Uveitis-IBD | TS_Thymus_b cell | Adult | Lymphatic system | B cell | CL:0000236 | Thymus | 4.85E-06 |
| Uveitis-IBD | TS_Liver_monocyte | Adult | Digestive system | Monocyte | CL:0000576 | Liver | 1.25E-05 |
| Uveitis-IBD | TS_Lymph_Node_b cell | Adult | Lymphatic system | B cell | CL:0000236 | Lymph node | 1.61E-05 |
| Uveitis-IBD | TS_Skin_endothelial cell | Adult | Integumentary system | Endothelial cell | CL:0000115 | Skin of body | 2.04E-05 |
| Uveitis-IBD | TS_Eye_monocyte | Adult | Sensory system | Monocyte | CL:0000576 | Eye | 3.25E-05 |
| Uveitis-IBD | TS_Pancreas_myeloid cell | Adult | Digestive system | Myeloid cell | CL:0000763 | Pancreas | 2.59E-05 |
| Uveitis-IBD | TS_Lung_macrophage | Adult | Respiratory system | Macrophage | CL:0000235 | Lung | 3.24E-05 |
| Uveitis-IBD | TS_Eye_retinal blood vessel endothelial cell | Adult | Sensory system | Endothelial cell | CL:0000115 | Eye | 3.25E-05 |
| Uveitis-IBS | FetalLiver_Macrophage | Fetal | Digestive system | Macrophage | CL:0000235 | Liver | 3.87E-06 |
| Uveitis-IBS | AdultLung_M2 Macrophage | Adult | Respiratory system | Macrophage | CL:0000235 | Lung | 1.86E-05 |
| Uveitis-IBS | TS_Tongue_epithelial cell | Adult | Digestive system | Epithelial cell | CL:0000066 | Tongue | 2.88E-06 |
| Uveitis-IBS | TS_Lung_macrophage | Adult | Respiratory system | Macrophage | CL:0000235 | Lung | 3.75E-06 |
| Uveitis-IBS | TS_Bone_Marrow_macrophage | Adult | Lymphatic system | Macrophage | CL:0000235 | Bone marrow | 9.63E-06 |
| Uveitis-IBS | TS_Muscle_endothelial cell of artery | Adult | Muscular system | Endothelial cell | CL:0000115 | Muscle organ | 2.58E-05 |
| Uveitis-PUD | TS_Kidney_endothelial cell | Adult | Urinary system | Endothelial cell | CL:0000115 | Kidney | 1.51E-05 |
| Uveitis-PUD | TS_Muscle_capillary endothelial cell | Adult | Muscular system | Capillary endothelial cell | CL:0002144 | Muscle organ | 2.83E-06 |
| Uveitis-PUD | TS_Muscle_endothelial cell of artery | Adult | Muscular system | Endothelial cell | CL:0000115 | Muscle organ | 2.83E-06 |
| Uveitis-PUD | TS_Bladder_capillary endothelial cell | Adult | Urinary system | Capillary endothelial cell | CL:0002144 | Bladder organ | 3.37E-05 |
| Uveitis-PUD | TS_Muscle_pericyte cell | Adult | Muscular system | Pericyte cell | CL:0000669 | Muscle organ | 3.37E-05 |

Note：^a^Significant results with P reaching the Bonferroni-corrected threshold 3.69×10-5 (0.05/1,355)

**Table S13.** Significant TWAS results based on candidate pleiotropic genes

| **Trait-pairs** | **GENE** | **EQTL.R2** | **MODELCV.R2** | **MODELCV.PV** | **TWAS.Z** | **TWAS.P** | **TWAS.FDR** | **GTEx v8 Tissuea** | **Sample** |
| --- | --- | --- | --- | --- | --- | --- | --- | --- | --- |
| AMD-GORD | *HMGN4* | 6.61E-02 | 6.60E-02 | 1.00E-06 | 1.27E+01 | 8.49E-37 | 4.29E-34 | Whole Blood | 558 |
| AMD-IBD | *IRF5* | 5.93E-02 | 9.00E-02 | 1.20E-08 | 1.91E+01 | 3.89E-81 | 1.57E-78 | Whole Blood | 558 |
| AMD-IBD | *TNPO3* | 1.13E-01 | 1.20E-01 | 4.90E-11 | -5.47E+00 | 4.53E-08 | 2.41E-06 | Whole Blood | 558 |
| AMD-IBD | *ULK3* | 5.60E-02 | 5.60E-02 | 6.70E-06 | -1.46E+01 | 1.49E-48 | 5.02E-46 | Whole Blood | 558 |
| AMD-IBD | *MPI* | 4.48E-02 | 4.50E-02 | 5.30E-05 | 1.34E+01 | 3.26E-41 | 9.42E-39 | Whole Blood | 558 |
| AMD-IBD | *STMN3* | -1.20E-03 | 4.20E-03 | 1.20E-01 | 1.16E+01 | 6.60E-31 | 1.33E-28 | Whole Blood | 558 |
| AMD-PUD | *NTN5* | -1.06E-03 | -7.90E-04 | 3.90E-01 | 1.25E+01 | 5.98E-36 | 1.21E-32 | Whole Blood | 558 |
| DR-GORD | *UBA7* | 3.91E-02 | 3.90E-02 | 1.50E-04 | -1.12E+01 | 3.11E-29 | 3.70E-27 | Whole Blood | 558 |
| DR-GORD | *RBM6* | 1.90E-01 | 1.90E-01 | 3.10E-17 | -1.26E+01 | 2.16E-36 | 3.12E-34 | Whole Blood | 558 |
| DR-GORD | *ARL17B* | 1.53E-02 | 8.30E-02 | 4.30E-08 | 1.26E+01 | 2.05E-36 | 3.12E-34 | Whole Blood | 558 |
| DR-GORD | *UBE2Z* | 1.14E-02 | 2.00E-02 | 4.90E-03 | -1.64E+01 | 1.54E-60 | 5.19E-58 | Whole Blood | 558 |
| DR-GORD | *GATAD2A* | 1.58E-02 | 2.60E-02 | 1.70E-03 | 8.94E+00 | 3.85E-19 | 3.11E-17 | Whole Blood | 558 |
| DR-IBD | *DNLZ* | 2.08E-01 | 2.60E-01 | 8.00E-24 | -4.22E+00 | 2.42E-05 | 5.62E-04 | Whole Blood | 558 |
| DR-IBD | *CARD9* | 7.22E-02 | 7.20E-02 | 3.30E-07 | 1.60E+01 | 1.77E-57 | 4.47E-55 | Whole Blood | 558 |
| DR-IBD | *SDCCAG3* | 3.49E-02 | 3.50E-02 | 3.30E-04 | -4.09E+00 | 4.36E-05 | 9.09E-04 | Whole Blood | 558 |
| DR-PUD | *LAMC1* | 4.57E-02 | 4.60E-02 | 4.50E-05 | 1.32E+01 | 6.47E-40 | 1.31E-36 | Whole Blood | 558 |
| DR-PUD | *DMPK* | 3.70E-02 | 3.70E-02 | 2.20E-04 | -8.94E+00 | 3.98E-19 | 5.75E-17 | Whole Blood | 558 |
| DR-IBS | *KRTCAP3* | 4.59E-02 | 5.40E-02 | 9.90E-06 | -3.82E+00 | 1.35E-04 | 3.11E-03 | Whole Blood | 558 |
| DR-IBS | *PAM* | 2.75E-01 | 2.70E-01 | 2.20E-25 | 9.01E+00 | 2.05E-19 | 4.16E-17 | Whole Blood | 558 |
| DR-IBS | *AP3S2* | 5.76E-02 | 8.50E-02 | 2.80E-08 | -1.70E+01 | 4.46E-65 | 4.53E-62 | Whole Blood | 558 |
| DR-DD | *DCLK2* | 5.27E-02 | 5.30E-02 | 1.20E-05 | 1.39E+01 | 5.91E-44 | 2.40E-41 | Whole Blood | 558 |
| DR-DD | *CTSW* | 1.25E-02 | 4.50E-02 | 5.40E-05 | -1.55E+01 | 2.29E-54 | 1.55E-51 | Whole Blood | 558 |
| Myopia-DD | *CD55* | 8.55E-02 | 1.10E-01 | 6.20E-10 | -9.99E+00 | 1.70E-23 | 3.14E-21 | Whole Blood | 558 |
| Myopia-DD | *AFAP1* | 4.80E-01 | 5.00E-01 | 1.30E-52 | -1.36E+01 | 3.05E-42 | 6.19E-39 | Whole Blood | 558 |
| Myopia-DD | *KCNMA1* | 1.00E-01 | 1.00E-01 | 1.80E-09 | 3.05E+00 | 2.31E-03 | 2.77E-02 | Whole Blood | 558 |
| Cataract-GORD | *RBM6* | 1.90E-01 | 1.90E-01 | 3.10E-17 | -1.86E+01 | 7.96E-77 | 1.61E-73 | Whole Blood | 558 |
| Cataract-GORD | *BTN2A1* | 1.65E-01 | 1.70E-01 | 4.50E-15 | 4.52E+00 | 6.11E-06 | 2.38E-04 | Whole Blood | 558 |
| Cataract-IBD | *GPX1* | 5.60E-02 | 5.60E-02 | 6.80E-06 | -7.64E+00 | 2.10E-14 | 2.50E-12 | Whole Blood | 558 |
| PACG-IBD | *IRF5* | 5.93E-02 | 9.00E-02 | 1.20E-08 | 1.40E+01 | 1.50E-44 | 3.03E-41 | Whole Blood | 558 |
| PACG-IBS | *HERC2* | 2.81E-01 | 2.80E-01 | 5.20E-26 | -1.49E+01 | 2.42E-50 | 4.91E-47 | Whole Blood | 558 |
| PACG-DD | *HERC2* | 2.81E-01 | 2.80E-01 | 5.20E-26 | -1.32E+01 | 1.68E-39 | 1.70E-36 | Whole Blood | 558 |
| DED-IBD | *GPX1* | 5.60E-02 | 5.60E-02 | 6.80E-06 | -4.34E+00 | 1.42E-05 | 5.63E-04 | Whole Blood | 558 |
| DED-IBD | *IRF5* | 5.93E-02 | 9.00E-02 | 1.20E-08 | 1.00E+01 | 1.56E-23 | 7.89E-21 | Whole Blood | 558 |
| AMD-GORD | *ZNF322* | 2.96E-01 | 4.20E-01 | 6.40E-33 | -8.94E+00 | 3.79E-19 | 9.81E-17 | Colon Sigmoid | 266 |
| AMD-GORD | *HMGN4* | 6.49E-02 | 6.50E-02 | 1.50E-05 | 1.27E+01 | 1.06E-36 | 8.57E-34 | Colon Sigmoid | 266 |
| AMD-IBD | *IRF5* | 1.61E-01 | 1.90E-01 | 7.10E-14 | 1.92E+01 | 1.85E-82 | 1.20E-79 | Colon Sigmoid | 266 |
| AMD-IBD | *SCAMP2* | 1.10E-02 | 1.50E-02 | 2.70E-02 | 4.51E+00 | 6.50E-06 | 3.34E-04 | Colon Sigmoid | 266 |
| AMD-IBD | *LIME1* | 1.19E-01 | 1.20E-01 | 4.00E-09 | -1.16E+01 | 2.56E-31 | 7.20E-29 | Colon Sigmoid | 266 |
| AMD-IBD | *TNFRSF6B* | 1.24E-02 | 2.80E-02 | 3.80E-03 | 7.73E+00 | 1.10E-14 | 1.55E-12 | Colon Sigmoid | 266 |
| AMD-IBD | *RTEL1* | 6.00E-02 | 8.50E-02 | 8.00E-07 | 9.77E+00 | 1.56E-22 | 3.26E-20 | Colon Sigmoid | 266 |
| AMD-PUD | *RASIP1* | 1.59E-01 | 1.60E-01 | 8.20E-12 | 3.18E+01 | 1.81E-221 | 1.17E-217 | Colon Sigmoid | 266 |
| AMD-PUD | *MAPK10* | 6.75E-02 | 1.20E-01 | 3.00E-09 | 3.15E+00 | 1.65E-03 | 3.75E-02 | Colon Sigmoid | 266 |
| AMD-PUD | *NTN5* | 8.17E-02 | 1.70E-01 | 2.70E-12 | 2.48E+01 | 4.20E-136 | 9.06E-133 | Colon Sigmoid | 266 |
| AMD-PUD | *MAMSTR* | 4.50E-02 | 5.80E-02 | 4.40E-05 | 3.12E+01 | 1.94E-213 | 6.28E-210 | Colon Sigmoid | 266 |
| AMD-PUD | *IZUMO1* | 8.29E-02 | 1.60E-01 | 1.00E-11 | 8.02E+00 | 1.03E-15 | 3.92E-13 | Colon Sigmoid | 266 |
| DR-GORD | *RBM6* | 4.44E-01 | 4.40E-01 | 1.00E-35 | -1.23E+01 | 4.98E-35 | 1.24E-32 | Colon Sigmoid | 266 |
| DR-GORD | *WNT3* | 1.47E-01 | 1.50E-01 | 5.60E-11 | -1.37E+01 | 1.89E-42 | 5.32E-40 | Colon Sigmoid | 266 |
| DR-GORD | *KANSL1* | 2.00E-01 | 2.00E-01 | 6.00E-15 | -3.52E+00 | 4.25E-04 | 6.09E-03 | Colon Sigmoid | 266 |
| DR-GORD | *CRHR1* | 3.98E-02 | 4.00E-02 | 6.30E-04 | -1.37E+01 | 6.72E-43 | 1.98E-40 | Colon Sigmoid | 266 |
| DR-GORD | *TTLL6* | 6.19E-02 | 7.10E-02 | 6.40E-06 | -3.19E+00 | 1.44E-03 | 1.66E-02 | Colon Sigmoid | 266 |
| DR-GORD | *KCNK7* | 1.06E-01 | 1.10E-01 | 2.20E-08 | -2.20E+01 | 4.16E-107 | 5.38E-104 | Colon Sigmoid | 266 |
| DR-GORD | *IP6K1* | 1.94E-02 | 2.40E-02 | 6.50E-03 | 8.28E+00 | 1.27E-16 | 1.42E-14 | Colon Sigmoid | 266 |
| DR-GORD | *ZNF518B* | 1.08E-01 | 1.10E-01 | 2.60E-08 | 8.00E+00 | 1.28E-15 | 1.31E-13 | Colon Sigmoid | 266 |
| DR-GORD | *UBA7* | 5.69E-02 | 5.70E-02 | 5.10E-05 | -1.00E+01 | 9.38E-24 | 1.73E-21 | Colon Sigmoid | 266 |
| DR-GORD | *MAPT* | 2.81E-01 | 3.20E-01 | 6.90E-24 | 1.54E+01 | 1.67E-53 | 6.00E-51 | Colon Sigmoid | 266 |
| DR-IBD | *INPP5E* | 1.15E-01 | 1.10E-01 | 8.80E-09 | -1.27E+01 | 4.79E-37 | 1.94E-34 | Colon Sigmoid | 266 |
| DR-IBD | *GPSM1* | 3.34E-02 | 3.60E-02 | 1.10E-03 | -1.04E+01 | 3.75E-25 | 1.28E-22 | Colon Sigmoid | 266 |
| DR-IBD | *DNLZ* | 2.53E-01 | 3.00E-01 | 3.70E-22 | -8.12E+00 | 4.54E-16 | 6.68E-14 | Colon Sigmoid | 266 |
| DR-PUD | *RASIP1* | 1.59E-01 | 1.60E-01 | 8.20E-12 | 2.37E+01 | 6.77E-124 | 1.46E-120 | Colon Sigmoid | 266 |
| DR-PUD | *FBXL20* | 2.28E-02 | 4.20E-02 | 4.60E-04 | 1.41E+01 | 5.20E-45 | 6.73E-42 | Colon Sigmoid | 266 |
| DR-PUD | *LAMC1* | 5.40E-02 | 7.80E-02 | 2.20E-06 | -1.43E+01 | 2.47E-46 | 4.00E-43 | Colon Sigmoid | 266 |
| DR-PUD | *MAMSTR* | 4.50E-02 | 5.80E-02 | 4.40E-05 | 2.73E+01 | 5.98E-164 | 3.87E-160 | Colon Sigmoid | 266 |
| DR-PUD | *IZUMO1* | 8.29E-02 | 1.60E-01 | 1.00E-11 | 5.77E+00 | 7.74E-09 | 5.44E-07 | Colon Sigmoid | 266 |
| DR-PUD | *DMWD* | 4.98E-02 | 8.30E-02 | 1.00E-06 | -9.24E+00 | 2.56E-20 | 4.87E-18 | Colon Sigmoid | 266 |
| DR-IBS | *MTMR3* | 5.93E-02 | 5.90E-02 | 3.50E-05 | 6.33E+00 | 2.38E-10 | 2.60E-08 | Colon Sigmoid | 266 |
| DR-IBS | *AP3S2* | 3.86E-01 | 4.20E-01 | 8.70E-34 | 3.04E+00 | 2.35E-03 | 4.99E-02 | Colon Sigmoid | 266 |
| DR-IBS | *BMP8A* | 3.68E-02 | 3.70E-02 | 9.80E-04 | -1.88E+01 | 7.35E-79 | 1.19E-75 | Colon Sigmoid | 266 |
| DR-IBS | *TMEM106A* | 1.02E-01 | 1.00E-01 | 6.00E-08 | -1.36E+01 | 2.86E-42 | 2.32E-39 | Colon Sigmoid | 266 |
| DR-IBS | *NBR1* | 7.07E-02 | 7.10E-02 | 6.60E-06 | 1.52E+01 | 4.34E-52 | 5.64E-49 | Colon Sigmoid | 266 |
| DR-DD | *NUP160* | 1.51E-02 | 1.50E-02 | 2.50E-02 | 5.97E+00 | 2.43E-09 | 1.03E-07 | Colon Sigmoid | 266 |
| DED-IBD | *P4HA2* | 2.74E-02 | 4.30E-02 | 3.70E-04 | 6.30E+00 | 3.02E-10 | 4.55E-08 | Colon Sigmoid | 266 |
| DED-IBD | *IRF5* | 1.61E-01 | 1.90E-01 | 7.10E-14 | 1.43E+01 | 2.42E-46 | 3.92E-43 | Colon Sigmoid | 266 |
| DED-IBD | *AMT* | 3.82E-01 | 3.80E-01 | 1.10E-29 | 3.57E+00 | 3.53E-04 | 1.02E-02 | Colon Sigmoid | 266 |
| DED-IBD | *NICN1* | 2.24E-01 | 2.30E-01 | 1.00E-16 | 5.40E+00 | 6.58E-08 | 5.65E-06 | Colon Sigmoid | 266 |
| DED-IBD | *RNF123* | 2.01E-01 | 2.00E-01 | 8.50E-15 | -9.75E+00 | 1.79E-22 | 7.72E-20 | Colon Sigmoid | 266 |
| DED-IBD | *MST1* | 3.99E-02 | 1.00E-01 | 6.60E-08 | 7.02E+00 | 2.22E-12 | 4.59E-10 | Colon Sigmoid | 266 |
| DED-PUD | *RASIP1* | 1.59E-01 | 1.60E-01 | 8.20E-12 | 1.82E+01 | 2.54E-74 | 8.22E-71 | Colon Sigmoid | 266 |
| DED-PUD | *MAMSTR* | 4.50E-02 | 5.80E-02 | 4.40E-05 | 2.01E+01 | 1.65E-89 | 1.07E-85 | Colon Sigmoid | 266 |
| DED-DD | *ARHGAP15* | 9.59E-02 | 9.60E-02 | 1.50E-07 | -4.30E+00 | 1.70E-05 | 5.61E-04 | Colon Sigmoid | 266 |
| Myopia-GORD | *BICC1* | 1.72E-01 | 1.80E-01 | 1.30E-13 | 5.30E+00 | 1.17E-07 | 7.21E-06 | Colon Sigmoid | 266 |
| Myopia-IBD | *ARMS2* | 1.59E-01 | 2.40E-01 | 2.10E-17 | -2.97E+00 | 3.01E-03 | 4.49E-02 | Colon Sigmoid | 266 |
| Myopia-PUD | *ACP1* | 3.09E-02 | 4.20E-02 | 4.60E-04 | -1.23E+01 | 1.23E-34 | 2.65E-31 | Colon Sigmoid | 266 |
| Myopia-DD | *ARHGAP15* | 9.59E-02 | 9.60E-02 | 1.50E-07 | -9.22E+00 | 2.86E-20 | 4.65E-18 | Colon Sigmoid | 266 |
| Myopia-DD | *BICC1* | 1.72E-01 | 1.80E-01 | 1.30E-13 | 3.20E+00 | 1.40E-03 | 1.77E-02 | Colon Sigmoid | 266 |
| Myopia-DD | *GPR158* | 1.09E-01 | 1.10E-01 | 1.70E-08 | -8.17E+00 | 3.21E-16 | 4.01E-14 | Colon Sigmoid | 266 |
| Myopia-DD | *ANTXR2* | 1.47E-02 | 4.40E-02 | 3.50E-04 | 5.07E+00 | 3.90E-07 | 1.46E-05 | Colon Sigmoid | 266 |
| Myopia-DD | *CHRNB1* | 2.05E-01 | 2.90E-01 | 1.10E-21 | -1.22E+01 | 3.77E-34 | 4.90E-31 | Colon Sigmoid | 266 |
| Myopia-DD | *PCBP3* | 1.83E-01 | 1.80E-01 | 1.60E-13 | -8.45E+00 | 3.03E-17 | 4.19E-15 | Colon Sigmoid | 266 |
| Myopia-DD | *CD55* | 6.67E-02 | 1.10E-01 | 9.10E-09 | 1.18E+01 | 2.57E-32 | 1.86E-29 | Colon Sigmoid | 266 |
| Myopia-DD | *S100A10* | 1.20E-01 | 1.30E-01 | 1.60E-09 | 4.20E+00 | 2.71E-05 | 6.32E-04 | Colon Sigmoid | 266 |
| Uveitis-PUD | *RASIP1* | 1.59E-01 | 1.60E-01 | 8.20E-12 | 1.40E+01 | 2.25E-44 | 4.85E-41 | Colon Sigmoid | 266 |
| Uveitis-PUD | *MAMSTR* | 4.50E-02 | 5.80E-02 | 4.40E-05 | 1.78E+01 | 3.24E-71 | 2.10E-67 | Colon Sigmoid | 266 |
| Uveitis-PUD | *IZUMO1* | 8.29E-02 | 1.60E-01 | 1.00E-11 | 6.43E+00 | 1.31E-10 | 4.46E-08 | Colon Sigmoid | 266 |
| Uveitis-DD | *ARHGAP15* | 9.59E-02 | 9.60E-02 | 1.50E-07 | -9.41E+00 | 5.13E-21 | 6.67E-18 | Colon Sigmoid | 266 |
| Cataract-GORD | *RBM6* | 4.44E-01 | 4.40E-01 | 1.00E-35 | -1.80E+01 | 2.23E-72 | 1.44E-68 | Colon Sigmoid | 266 |
| Cataract-GORD | *MST1R* | 2.98E-02 | 4.00E-02 | 5.90E-04 | 1.24E+01 | 2.14E-35 | 3.46E-32 | Colon Sigmoid | 266 |
| Cataract-IBD | *NICN1* | 2.24E-01 | 2.30E-01 | 1.00E-16 | 8.01E+00 | 1.17E-15 | 2.61E-13 | Colon Sigmoid | 266 |
| Cataract-IBD | *RNF123* | 2.01E-01 | 2.00E-01 | 8.50E-15 | -1.13E+01 | 1.45E-29 | 9.38E-27 | Colon Sigmoid | 266 |
| Cataract-IBD | *MST1* | 3.99E-02 | 1.00E-01 | 6.60E-08 | 9.43E+00 | 4.07E-21 | 1.46E-18 | Colon Sigmoid | 266 |
| Cataract-IBD | *GPX1* | 1.82E-02 | 2.00E-02 | 1.10E-02 | -3.97E+00 | 7.32E-05 | 2.72E-03 | Colon Sigmoid | 266 |
| Cataract-DD | *ARHGAP15* | 9.59E-02 | 9.60E-02 | 1.50E-07 | -7.61E+00 | 2.82E-14 | 8.33E-12 | Colon Sigmoid | 266 |
| PACG-IBD | *IRF5* | 1.61E-01 | 1.90E-01 | 7.10E-14 | 1.52E+01 | 1.69E-52 | 1.09E-48 | Colon Sigmoid | 266 |
| PACG-DD | *COX15* | 4.71E-02 | 6.30E-02 | 2.00E-05 | -1.42E+01 | 1.36E-45 | 8.84E-42 | Colon Sigmoid | 266 |
| PACG-DD | *ARHGAP15* | 9.59E-02 | 9.60E-02 | 1.50E-07 | -5.02E+00 | 5.07E-07 | 3.33E-05 | Colon Sigmoid | 266 |
| Keratitis-DD | *COX15* | 4.71E-02 | 6.30E-02 | 2.00E-05 | -1.01E+01 | 5.45E-24 | 7.09E-21 | Colon Sigmoid | 266 |
| Keratitis-DD | *GPR158* | 1.09E-01 | 1.10E-01 | 1.70E-08 | -1.59E+01 | 7.89E-57 | 5.13E-53 | Colon Sigmoid | 266 |
| Keratitis-DD | *S100A10* | 1.20E-01 | 1.30E-01 | 1.60E-09 | 7.59E+00 | 3.11E-14 | 1.56E-11 | Colon Sigmoid | 266 |
| AMD-GORD | *ABT1* | 6.46E-02 | 6.50E-02 | 6.10E-06 | 4.70E+00 | 2.55E-06 | 1.11E-04 | Colon Transverse | 294 |
| AMD-GORD | *ZNF322* | 3.01E-01 | 3.30E-01 | 9.70E-28 | -4.23E+00 | 2.34E-05 | 7.74E-04 | Colon Transverse | 294 |
| AMD-PUD | *RASIP1* | 2.20E-02 | 5.50E-02 | 2.90E-05 | 2.93E+01 | 1.60E-188 | 3.49E-185 | Colon Transverse | 294 |
| AMD-PUD | *MAPK10* | 1.36E-02 | 1.40E-02 | 2.50E-02 | 5.60E+00 | 2.18E-08 | 3.15E-06 | Colon Transverse | 294 |
| AMD-PUD | *NTN5* | 7.37E-02 | 7.60E-02 | 9.40E-07 | 2.95E+01 | 1.06E-191 | 3.47E-188 | Colon Transverse | 294 |
| AMD-PUD | *FUT2* | 2.60E-01 | 2.60E-01 | 4.10E-21 | -3.26E+01 | 1.20E-233 | 7.86E-230 | Colon Transverse | 294 |
| AMD-IBS | *HTRA1* | 2.14E-02 | 4.10E-02 | 2.80E-04 | 5.61E+00 | 2.05E-08 | 2.42E-06 | Colon Transverse | 294 |
| AMD-IBD | *IRF5* | 2.82E-01 | 2.90E-01 | 2.70E-23 | 1.73E+01 | 1.05E-66 | 4.91E-64 | Colon Transverse | 294 |
| AMD-IBD | *RPP25* | 3.55E-02 | 5.40E-02 | 3.60E-05 | -1.51E+01 | 1.82E-51 | 6.27E-49 | Colon Transverse | 294 |
| AMD-IBD | *FAM219B* | 7.56E-02 | 8.20E-02 | 3.60E-07 | 1.62E+01 | 1.02E-58 | 3.93E-56 | Colon Transverse | 294 |
| AMD-IBD | *LIME1* | 8.32E-02 | 9.30E-02 | 5.80E-08 | -6.36E+00 | 1.99E-10 | 1.59E-08 | Colon Transverse | 294 |
| DR-GORD | *RBM6* | 5.64E-01 | 5.60E-01 | 9.20E-55 | -1.25E+01 | 5.08E-36 | 1.11E-33 | Colon Transverse | 294 |
| DR-GORD | *KDM2B* | 7.20E-02 | 7.80E-02 | 6.50E-07 | 1.11E+01 | 7.25E-29 | 1.36E-26 | Colon Transverse | 294 |
| DR-GORD | *WNT3* | 1.31E-01 | 1.40E-01 | 2.50E-11 | -1.48E+01 | 1.38E-49 | 4.76E-47 | Colon Transverse | 294 |
| DR-GORD | *KANSL1* | 1.51E-01 | 2.00E-01 | 6.80E-16 | -5.23E+00 | 1.70E-07 | 5.46E-06 | Colon Transverse | 294 |
| DR-GORD | *SNF8* | 2.54E-03 | 1.50E-02 | 1.80E-02 | 1.73E+01 | 2.31E-67 | 1.38E-64 | Colon Transverse | 294 |
| DR-GORD | *TTLL6* | 5.70E-02 | 8.20E-02 | 3.40E-07 | -9.26E+00 | 1.97E-20 | 2.58E-18 | Colon Transverse | 294 |
| DR-GORD | *KCNK7* | 1.05E-01 | 1.00E-01 | 8.00E-09 | -2.14E+01 | 5.12E-102 | 1.12E-98 | Colon Transverse | 294 |
| DR-GORD | *UBA7* | 2.62E-01 | 2.60E-01 | 2.80E-21 | -1.00E+01 | 9.38E-24 | 1.43E-21 | Colon Transverse | 294 |
| DR-GORD | *MAPT* | 1.00E-01 | 1.20E-01 | 1.30E-09 | 7.20E+00 | 6.06E-13 | 4.41E-11 | Colon Transverse | 294 |
| DR-IBD | *CDKAL1* | 4.30E-03 | 1.40E-02 | 2.40E-02 | 4.00E+00 | 6.26E-05 | 1.43E-03 | Colon Transverse | 294 |
| DR-IBD | *INPP5E* | 9.53E-02 | 9.80E-02 | 2.50E-08 | -1.42E+01 | 1.48E-45 | 5.39E-43 | Colon Transverse | 294 |
| DR-IBD | *GPSM1* | 2.19E-02 | 2.70E-02 | 2.60E-03 | -1.96E+01 | 1.01E-85 | 5.52E-83 | Colon Transverse | 294 |
| DR-IBD | *PEAK1* | 1.41E-01 | 1.40E-01 | 1.50E-11 | 2.73E+00 | 6.43E-03 | 6.56E-02 | Colon Transverse | 294 |
| DR-IBD | *DNLZ* | 3.71E-01 | 3.90E-01 | 1.10E-33 | -5.62E+00 | 1.87E-08 | 9.01E-07 | Colon Transverse | 294 |
| DR-PUD | *DMPK* | 4.39E-02 | 4.40E-02 | 1.70E-04 | -9.56E+00 | 1.20E-21 | 2.91E-19 | Colon Transverse | 294 |
| DR-PUD | *RASIP1* | 2.20E-02 | 5.50E-02 | 2.90E-05 | 2.51E+01 | 6.25E-139 | 1.37E-135 | Colon Transverse | 294 |
| DR-PUD | *FBXL20* | 1.58E-01 | 1.60E-01 | 6.60E-13 | 1.43E+01 | 1.17E-46 | 1.92E-43 | Colon Transverse | 294 |
| DR-PUD | *LAMC1* | 1.52E-03 | 4.60E-02 | 1.30E-04 | -1.30E+01 | 7.64E-39 | 1.00E-35 | Colon Transverse | 294 |
| DR-PUD | *CDKAL1* | 4.30E-03 | 1.40E-02 | 2.40E-02 | -7.06E+00 | 1.65E-12 | 2.08E-10 | Colon Transverse | 294 |
| DR-PUD | *FUT2* | 2.60E-01 | 2.60E-01 | 4.10E-21 | -2.84E+01 | 5.34E-177 | 3.50E-173 | Colon Transverse | 294 |
| DR-PUD | *DMWD* | 2.79E-02 | 5.30E-02 | 3.80E-05 | -9.99E+00 | 1.71E-23 | 4.48E-21 | Colon Transverse | 294 |
| DR-IBS | *NRBP1* | 1.83E-01 | 1.80E-01 | 1.00E-14 | -2.05E+01 | 2.98E-93 | 6.55E-90 | Colon Transverse | 294 |
| DR-IBS | *DENND1A* | 3.97E-02 | 4.00E-02 | 3.50E-04 | -7.82E+00 | 5.09E-15 | 7.99E-13 | Colon Transverse | 294 |
| DR-IBS | *GIN1* | 3.73E-02 | 6.70E-02 | 4.20E-06 | 1.11E+01 | 1.48E-28 | 8.14E-26 | Colon Transverse | 294 |
| DR-IBS | *BMP8A* | 4.41E-02 | 5.10E-02 | 5.20E-05 | -4.66E+00 | 3.09E-06 | 1.49E-04 | Colon Transverse | 294 |
| DR-IBS | *NBR1* | 2.44E-02 | 3.20E-02 | 1.10E-03 | -7.48E+00 | 7.20E-14 | 9.69E-12 | Colon Transverse | 294 |
| DED-IBD | *P4HA2* | 5.85E-02 | 5.80E-02 | 1.70E-05 | 1.07E+01 | 1.05E-26 | 7.65E-24 | Colon Transverse | 294 |
| DED-IBD | *IRF5* | 2.82E-01 | 2.90E-01 | 2.70E-23 | 1.29E+01 | 3.15E-38 | 4.13E-35 | Colon Transverse | 294 |
| DED-IBD | *AMT* | 2.56E-01 | 2.70E-01 | 8.40E-22 | 4.35E+00 | 1.36E-05 | 6.65E-04 | Colon Transverse | 294 |
| DED-IBD | *NICN1* | 8.37E-02 | 1.00E-01 | 1.00E-08 | 4.06E+00 | 4.92E-05 | 1.97E-03 | Colon Transverse | 294 |
| DED-IBD | *RNF123* | 1.13E-01 | 1.60E-01 | 1.10E-12 | -8.94E+00 | 3.80E-19 | 1.31E-16 | Colon Transverse | 294 |
| DED-IBD | *MST1* | 7.09E-03 | 4.50E-02 | 1.40E-04 | 5.95E+00 | 2.66E-09 | 3.42E-07 | Colon Transverse | 294 |
| DED-PUD | *RASIP1* | 2.20E-02 | 5.50E-02 | 2.90E-05 | 1.69E+01 | 7.42E-64 | 1.62E-60 | Colon Transverse | 294 |
| DED-PUD | *FUT2* | 2.60E-01 | 2.60E-01 | 4.10E-21 | -1.99E+01 | 2.72E-88 | 1.78E-84 | Colon Transverse | 294 |
| Uveitis-PUD | *RASIP1* | 2.20E-02 | 5.50E-02 | 2.90E-05 | 1.91E+01 | 1.27E-81 | 8.32E-78 | Colon Transverse | 294 |
| Uveitis-PUD | *FUT2* | 2.60E-01 | 2.60E-01 | 4.10E-21 | -1.83E+01 | 5.50E-75 | 1.20E-71 | Colon Transverse | 294 |
| Myopia-GORD | *BICC1* | 3.45E-02 | 6.50E-02 | 6.00E-06 | 7.00E+00 | 2.51E-12 | 3.38E-10 | Colon Transverse | 294 |
| Myopia-IBD | *GGACT* | 2.09E-01 | 2.50E-01 | 5.70E-20 | -3.77E+00 | 1.62E-04 | 4.25E-03 | Colon Transverse | 294 |
| Myopia-IBD | *ARMS2* | 8.97E-02 | 9.00E-02 | 8.80E-08 | -3.07E+00 | 2.18E-03 | 3.45E-02 | Colon Transverse | 294 |
| Myopia-PUD | *GPD2* | 7.25E-02 | 7.30E-02 | 1.70E-06 | -2.17E+01 | 1.95E-104 | 1.28E-100 | Colon Transverse | 294 |
| Myopia-PUD | *FUT2* | 2.60E-01 | 2.60E-01 | 4.10E-21 | -1.59E+01 | 3.07E-57 | 6.71E-54 | Colon Transverse | 294 |
| Myopia-DD | *BICC1* | 3.45E-02 | 6.50E-02 | 6.00E-06 | 7.71E+00 | 1.24E-14 | 2.10E-12 | Colon Transverse | 294 |
| Myopia-DD | *CHRNB1* | 9.47E-02 | 1.10E-01 | 4.80E-09 | -4.08E+01 | 0.00E+00 | 0.00E+00 | Colon Transverse | 294 |
| Cataract-GORD | *RBM6* | 5.64E-01 | 5.60E-01 | 9.20E-55 | -1.81E+01 | 3.27E-73 | 1.07E-69 | Colon Transverse | 294 |
| Cataract-GORD | *MST1R* | 5.40E-02 | 5.40E-02 | 3.40E-05 | 1.96E+01 | 2.49E-85 | 1.63E-81 | Colon Transverse | 294 |
| Cataract-IBD | *NICN1* | 8.37E-02 | 1.00E-01 | 1.00E-08 | 6.85E+00 | 7.29E-12 | 1.33E-09 | Colon Transverse | 294 |
| Cataract-IBD | *RNF123* | 1.13E-01 | 1.60E-01 | 1.10E-12 | -9.82E+00 | 9.03E-23 | 4.23E-20 | Colon Transverse | 294 |
| Cataract-IBD | *MST1* | 7.09E-03 | 4.50E-02 | 1.40E-04 | 7.77E+00 | 7.62E-15 | 1.92E-12 | Colon Transverse | 294 |
| PACG-IBD | *IRF5* | 2.82E-01 | 2.90E-01 | 2.70E-23 | 1.33E+01 | 4.00E-40 | 2.62E-36 | Colon Transverse | 294 |
| Keratitis-IBS | *DMAP1* | 8.58E-02 | 8.60E-02 | 1.90E-07 | 1.36E+01 | 7.31E-42 | 4.82E-38 | Colon Transverse | 294 |
| AMD-IBD | *ULK3* | 1.77E-01 | 1.90E-01 | 1.10E-06 | -1.38E+01 | 1.78E-43 | 6.25E-41 | EBV-transformed | 113 |
| AMD-IBD | *MPI* | 7.84E-02 | 1.20E-01 | 7.30E-05 | 1.47E+01 | 4.23E-49 | 1.70E-46 | EBV-transformed | 113 |
| DR-GORD | *RBM6* | 7.49E-02 | 1.10E-01 | 1.90E-04 | -1.26E+01 | 3.36E-36 | 4.49E-34 | EBV-transformed | 113 |
| DR-GORD | *NSF* | 1.95E-01 | 2.10E-01 | 1.80E-07 | -3.67E+00 | 2.47E-04 | 3.30E-03 | EBV-transformed | 113 |
| DR-GORD | *WNT3* | 4.48E-02 | 6.20E-02 | 4.40E-03 | -1.43E+01 | 4.04E-46 | 7.09E-44 | EBV-transformed | 113 |
| DR-GORD | *KANSL1* | 2.89E-01 | 3.40E-01 | 9.50E-12 | -6.16E+00 | 7.34E-10 | 3.44E-08 | EBV-transformed | 113 |
| DR-GORD | *UBE2Z* | 1.13E-01 | 1.80E-01 | 2.00E-06 | -1.14E+01 | 7.31E-30 | 8.56E-28 | EBV-transformed | 113 |
| DR-GORD | *ARHGAP27* | 7.56E-02 | 2.10E-01 | 1.40E-07 | 1.77E+01 | 3.22E-70 | 1.00E-67 | EBV-transformed | 113 |
| DR-GORD | *ARL17B* | 1.63E-01 | 3.70E-01 | 4.50E-13 | -1.02E+01 | 2.07E-24 | 2.24E-22 | EBV-transformed | 113 |
| DR-IBD | *INPP5E* | -8.74E-03 | 2.30E-02 | 6.00E-02 | -5.93E+00 | 3.01E-09 | 1.39E-07 | EBV-transformed | 113 |
| DR-IBD | *DNLZ* | 1.06E-01 | 2.50E-01 | 1.10E-08 | -1.21E+01 | 8.25E-34 | 2.11E-31 | EBV-transformed | 113 |
| DR-IBS | *NUCB2* | 2.66E-01 | 2.70E-01 | 3.00E-09 | -3.99E+00 | 6.74E-05 | 2.41E-03 | EBV-transformed | 113 |
| DR-IBS | *NRBP1* | 2.34E-01 | 2.60E-01 | 6.10E-09 | -1.12E+01 | 6.47E-29 | 2.61E-26 | EBV-transformed | 113 |
| DR-IBS | *AP3S2* | 3.01E-01 | 3.30E-01 | 2.20E-11 | 9.88E+00 | 5.33E-23 | 1.37E-20 | EBV-transformed | 113 |
| DED-IBD | *AMT* | 7.71E-02 | 1.10E-01 | 2.30E-04 | 5.11E+00 | 3.21E-07 | 1.88E-05 | EBV-transformed | 113 |
| DED-IBD | *RNF123* | 3.37E-02 | 5.00E-02 | 9.90E-03 | -6.70E+00 | 2.04E-11 | 2.73E-09 | EBV-transformed | 113 |
| Myopia-PUD | *CNDP2* | 2.40E-01 | 2.70E-01 | 2.60E-09 | -1.36E+01 | 2.04E-42 | 5.73E-39 | EBV-transformed | 113 |
| Cataract-GORD | *RBM6* | 7.49E-02 | 1.10E-01 | 1.90E-04 | -1.83E+01 | 1.07E-74 | 3.01E-71 | EBV-transformed | 113 |
| Cataract-IBD | *RNF123* | 3.37E-02 | 5.00E-02 | 9.90E-03 | -1.01E+01 | 7.60E-24 | 7.12E-21 | EBV-transformed | 113 |
| AMD-GORD | *ZNF322* | 1.72E-01 | 2.30E-01 | 6.60E-12 | -1.39E+01 | 3.78E-44 | 1.38E-41 | Liver | 178 |
| AMD-GORD | *HMGN4* | 1.44E-01 | 1.40E-01 | 1.00E-07 | 1.57E+01 | 3.09E-55 | 1.62E-52 | Liver | 178 |
| AMD-IBD | *ARFRP1* | 2.14E-02 | 2.70E-02 | 1.60E-02 | 1.54E+01 | 3.38E-53 | 7.28E-51 | Liver | 178 |
| AMD-IBD | *SLC2A4RG* | 1.66E-01 | 1.70E-01 | 9.80E-09 | 1.76E+01 | 2.01E-69 | 6.69E-67 | Liver | 178 |
| AMD-IBD | *IRF5* | 2.36E-01 | 2.50E-01 | 5.60E-13 | 1.56E+01 | 1.48E-54 | 3.39E-52 | Liver | 178 |
| AMD-IBD | *ULK3* | 1.69E-01 | 1.70E-01 | 7.10E-09 | -1.22E+01 | 5.20E-34 | 8.27E-32 | Liver | 178 |
| AMD-IBD | *RPP25* | 2.65E-02 | 6.10E-02 | 5.00E-04 | -1.56E+01 | 8.40E-55 | 2.05E-52 | Liver | 178 |
| DR-IBD | *TRPS1* | 5.13E-02 | 5.10E-02 | 1.40E-03 | -1.66E+01 | 4.66E-62 | 1.07E-59 | Liver | 178 |
| DR-IBD | *PMPCA* | 2.58E-01 | 2.60E-01 | 2.70E-13 | 1.37E+01 | 1.01E-42 | 2.06E-40 | Liver | 178 |
| DR-IBD | *DNLZ* | 2.45E-01 | 2.40E-01 | 1.30E-12 | 4.25E+00 | 2.10E-05 | 5.66E-04 | Liver | 178 |
| DR-PUD | *DMPK* | 1.12E-01 | 1.10E-01 | 3.10E-06 | -7.92E+00 | 2.38E-15 | 2.29E-13 | Liver | 178 |
| DR-IBS | *BRCA1* | 2.44E-01 | 2.40E-01 | 1.60E-12 | -1.55E+01 | 5.04E-54 | 2.06E-51 | Liver | 178 |
| DR-IBS | *PPP2R3A* | 5.07E-02 | 6.00E-02 | 5.60E-04 | 3.82E+00 | 1.36E-04 | 3.77E-03 | Liver | 178 |
| DR-IBS | *TRPS1* | 5.13E-02 | 5.10E-02 | 1.40E-03 | -1.73E+01 | 6.95E-67 | 3.66E-64 | Liver | 178 |
| DR-IBS | *AP3S2* | 4.32E-01 | 4.40E-01 | 3.50E-24 | -1.30E+01 | 1.00E-38 | 2.83E-36 | Liver | 178 |
| DED-IBD | *IRF5* | 2.36E-01 | 2.50E-01 | 5.60E-13 | 9.94E+00 | 2.82E-23 | 1.15E-20 | Liver | 178 |
| Myopia-DD | *COL6A1* | 3.75E-02 | 6.10E-02 | 5.30E-04 | -1.35E+01 | 2.79E-41 | 2.06E-38 | Liver | 178 |
| Myopia-DD | *S100A10* | 2.64E-01 | 2.60E-01 | 1.40E-13 | -1.63E+01 | 1.39E-59 | 2.56E-56 | Liver | 178 |
| Cataract-GORD | *RBM6* | 2.82E-01 | 2.80E-01 | 1.60E-14 | -1.81E+01 | 3.27E-73 | 1.20E-69 | Liver | 178 |
| PACG-IBD | *IRF5* | 2.36E-01 | 2.50E-01 | 5.60E-13 | 1.26E+01 | 1.70E-36 | 5.82E-33 | Liver | 178 |
| Keratitis-DD | *S100A10* | 2.64E-01 | 2.60E-01 | 1.40E-13 | -1.72E+01 | 1.87E-66 | 3.44E-63 | Liver | 178 |
| AMD-GORD | *ABT1* | 4.20E-02 | 4.70E-02 | 1.10E-06 | 3.86E+00 | 1.12E-04 | 3.15E-03 | Adipose-Subcutaneous | 479 |
| AMD-GORD | *ZNF322* | 3.15E-01 | 4.30E-01 | 6.70E-60 | -6.13E+00 | 8.83E-10 | 7.13E-08 | Adipose-Subcutaneous | 479 |
| AMD-IBD | *CSK* | 6.05E-02 | 6.80E-02 | 4.20E-09 | -1.83E+01 | 9.03E-75 | 5.52E-72 | Adipose-Subcutaneous | 479 |
| AMD-IBD | *IRF5* | 1.37E-01 | 1.40E-01 | 3.10E-17 | 1.73E+01 | 9.40E-67 | 5.10E-64 | Adipose-Subcutaneous | 479 |
| AMD-IBD | *ULK3* | 1.15E-01 | 1.30E-01 | 7.10E-17 | -1.36E+01 | 4.21E-42 | 1.79E-39 | Adipose-Subcutaneous | 479 |
| AMD-IBD | *SCAMP2* | 1.55E-02 | 5.60E-02 | 1.00E-07 | 1.03E+01 | 8.29E-25 | 1.93E-22 | Adipose-Subcutaneous | 479 |
| AMD-IBD | *RPP25* | 3.26E-01 | 3.30E-01 | 5.10E-43 | -1.19E+01 | 7.36E-33 | 2.66E-30 | Adipose-Subcutaneous | 479 |
| AMD-IBD | *FAM219B* | 6.69E-02 | 7.10E-02 | 1.80E-09 | 1.67E+01 | 1.95E-62 | 9.53E-60 | Adipose-Subcutaneous | 479 |
| AMD-IBD | *MPI* | 5.05E-02 | 5.00E-02 | 4.00E-07 | 1.17E+01 | 8.61E-32 | 2.90E-29 | Adipose-Subcutaneous | 479 |
| AMD-IBD | *LIME1* | 8.22E-03 | 2.40E-02 | 3.60E-04 | -8.20E+00 | 2.36E-16 | 4.12E-14 | Adipose-Subcutaneous | 479 |
| AMD-IBD | *RTEL1* | 7.93E-02 | 1.00E-01 | 4.50E-13 | 1.46E+01 | 1.60E-48 | 7.45E-46 | Adipose-Subcutaneous | 479 |
| AMD-PUD | *NTN5* | 1.96E-03 | 7.20E-02 | 1.40E-09 | 1.27E+01 | 3.50E-37 | 1.14E-33 | Adipose-Subcutaneous | 479 |
| AMD-PUD | *FUT1* | 1.11E-01 | 1.10E-01 | 4.10E-14 | -1.99E+01 | 3.28E-88 | 3.21E-84 | Adipose-Subcutaneous | 479 |
| AMD-PUD | *IZUMO1* | 2.20E-01 | 3.50E-01 | 2.40E-46 | 8.51E+00 | 1.71E-17 | 9.28E-15 | Adipose-Subcutaneous | 479 |
| DR-GORD | *RBM6* | 5.63E-01 | 5.60E-01 | 2.00E-88 | -1.23E+01 | 4.89E-35 | 1.23E-32 | Adipose-Subcutaneous | 479 |
| DR-GORD | *KDM2B* | 7.67E-02 | 7.70E-02 | 4.20E-10 | 7.85E+00 | 4.30E-15 | 4.38E-13 | Adipose-Subcutaneous | 479 |
| DR-GORD | *WNT3* | 5.84E-02 | 1.30E-01 | 8.50E-17 | -1.21E+01 | 6.23E-34 | 1.49E-31 | Adipose-Subcutaneous | 479 |
| DR-GORD | *THADA* | 8.82E-03 | 2.20E-02 | 6.70E-04 | -1.43E+01 | 3.98E-46 | 1.34E-43 | Adipose-Subcutaneous | 479 |
| DR-GORD | *CRHR1* | 1.16E-01 | 1.20E-01 | 1.20E-14 | -1.84E+01 | 2.83E-75 | 3.07E-72 | Adipose-Subcutaneous | 479 |
| DR-GORD | *ARHGAP27* | 1.06E-02 | 2.00E-02 | 1.20E-03 | 1.55E+01 | 3.81E-54 | 1.55E-51 | Adipose-Subcutaneous | 479 |
| DR-GORD | *LTBP3* | 1.26E-01 | 1.60E-01 | 3.40E-20 | 1.23E+01 | 1.04E-34 | 2.54E-32 | Adipose-Subcutaneous | 479 |
| DR-GORD | *TTLL6* | 7.19E-02 | 8.30E-02 | 8.50E-11 | -8.87E+00 | 7.13E-19 | 9.29E-17 | Adipose-Subcutaneous | 479 |
| DR-GORD | *KCNK7* | 5.73E-02 | 5.70E-02 | 6.80E-08 | -2.14E+01 | 5.12E-102 | 8.34E-99 | Adipose-Subcutaneous | 479 |
| DR-GORD | *EHBP1L1* | 9.03E-03 | 9.00E-03 | 2.10E-02 | 4.45E+00 | 8.55E-06 | 2.21E-04 | Adipose-Subcutaneous | 479 |
| DR-GORD | *IP6K1* | 2.68E-02 | 2.70E-02 | 1.90E-04 | 8.81E+00 | 1.30E-18 | 1.67E-16 | Adipose-Subcutaneous | 479 |
| DR-GORD | *UBA7* | 8.71E-03 | 1.20E-02 | 9.10E-03 | -1.10E+01 | 4.55E-28 | 8.55E-26 | Adipose-Subcutaneous | 479 |
| DR-GORD | *MAPT* | 2.87E-02 | 2.90E-02 | 1.10E-04 | -1.37E+01 | 6.72E-43 | 2.12E-40 | Adipose-Subcutaneous | 479 |
| DR-GORD | *PLEKHM1* | 6.68E-02 | 6.70E-02 | 5.60E-09 | -1.37E+01 | 6.72E-43 | 2.12E-40 | Adipose-Subcutaneous | 479 |
| DR-GORD | *ARL17B* | 5.46E-02 | 1.90E-01 | 7.90E-24 | 4.55E+00 | 5.47E-06 | 1.54E-04 | Adipose-Subcutaneous | 479 |
| DR-IBD | *THADA* | 8.82E-03 | 2.20E-02 | 6.70E-04 | -2.02E+01 | 2.06E-90 | 1.18E-87 | Adipose-Subcutaneous | 479 |
| DR-IBD | *GPSM1* | 3.52E-02 | 1.90E-01 | 2.60E-23 | -8.41E+00 | 3.97E-17 | 6.93E-15 | Adipose-Subcutaneous | 479 |
| DR-IBD | *DNLZ* | 3.07E-01 | 4.40E-01 | 6.70E-62 | -8.71E+00 | 3.08E-18 | 5.79E-16 | Adipose-Subcutaneous | 479 |
| DR-PUD | *DMPK* | 8.00E-02 | 8.00E-02 | 1.80E-10 | -1.37E+01 | 7.45E-43 | 2.43E-39 | Adipose-Subcutaneous | 479 |
| DR-PUD | *FBXL20* | 6.26E-02 | 7.30E-02 | 1.00E-09 | 1.44E+01 | 3.91E-47 | 3.82E-43 | Adipose-Subcutaneous | 479 |
| DR-PUD | *THADA* | 8.82E-03 | 2.20E-02 | 6.70E-04 | -9.94E+00 | 2.74E-23 | 8.93E-21 | Adipose-Subcutaneous | 479 |
| DR-PUD | *IZUMO1* | 2.20E-01 | 3.50E-01 | 2.40E-46 | 8.35E+00 | 7.04E-17 | 1.30E-14 | Adipose-Subcutaneous | 479 |
| DR-PUD | *DMWD* | 5.31E-02 | 6.30E-02 | 1.60E-08 | -5.72E+00 | 1.08E-08 | 7.65E-07 | Adipose-Subcutaneous | 479 |
| DR-IBS | *PPP2R3A* | 7.40E-03 | 7.40E-03 | 3.30E-02 | -1.71E+01 | 2.46E-65 | 2.69E-62 | Adipose-Subcutaneous | 479 |
| DR-IBS | *PABPC4* | 1.51E-01 | 1.50E-01 | 2.90E-19 | -6.13E+00 | 8.61E-10 | 8.29E-08 | Adipose-Subcutaneous | 479 |
| DR-IBS | *MTMR3* | 1.05E-01 | 1.20E-01 | 7.90E-15 | 6.76E+00 | 1.40E-11 | 1.79E-09 | Adipose-Subcutaneous | 479 |
| DR-IBS | *VAT1* | 7.14E-02 | 9.70E-02 | 1.90E-12 | -3.82E+00 | 1.32E-04 | 4.41E-03 | Adipose-Subcutaneous | 479 |
| DR-IBS | *NRBP1* | 1.31E-01 | 1.40E-01 | 9.20E-18 | 3.22E+00 | 1.30E-03 | 3.02E-02 | Adipose-Subcutaneous | 479 |
| DR-IBS | *PPM1G* | 8.07E-02 | 8.10E-02 | 1.40E-10 | 1.24E+01 | 3.61E-35 | 2.36E-32 | Adipose-Subcutaneous | 479 |
| DR-IBS | *DENND1A* | 5.70E-02 | 5.70E-02 | 7.40E-08 | 5.32E+00 | 1.03E-07 | 6.88E-06 | Adipose-Subcutaneous | 479 |
| DR-IBS | *GIN1* | 4.12E-02 | 4.10E-02 | 4.50E-06 | 1.04E+01 | 2.27E-25 | 8.58E-23 | Adipose-Subcutaneous | 479 |
| DR-IBS | *AP3S2* | 4.84E-01 | 5.10E-01 | 4.70E-76 | 3.16E+00 | 1.57E-03 | 3.50E-02 | Adipose-Subcutaneous | 479 |
| DR-IBS | *VPS33B* | 5.82E-02 | 1.10E-01 | 1.60E-14 | 6.17E+00 | 6.71E-10 | 6.59E-08 | Adipose-Subcutaneous | 479 |
| DR-IBS | *TMEM106A* | 5.04E-02 | 5.10E-02 | 3.50E-07 | -4.61E+00 | 3.95E-06 | 1.93E-04 | Adipose-Subcutaneous | 479 |
| DR-IBS | *NCR3LG1* | 1.49E-01 | 1.50E-01 | 1.10E-18 | -1.21E+01 | 1.25E-33 | 6.93E-31 | Adipose-Subcutaneous | 479 |
| DED-IBD | *IRF5* | 1.37E-01 | 1.40E-01 | 3.10E-17 | 1.29E+01 | 2.45E-38 | 5.99E-35 | Adipose-Subcutaneous | 479 |
| DED-IBD | *AMT* | 4.35E-01 | 4.40E-01 | 8.90E-63 | 4.87E+00 | 1.09E-06 | 8.07E-05 | Adipose-Subcutaneous | 479 |
| DED-IBD | *TCTA* | 3.48E-02 | 4.20E-02 | 3.50E-06 | 4.17E+00 | 3.02E-05 | 1.39E-03 | Adipose-Subcutaneous | 479 |
| DED-IBD | *NICN1* | 1.83E-01 | 2.00E-01 | 3.60E-25 | 8.14E+00 | 3.91E-16 | 1.66E-13 | Adipose-Subcutaneous | 479 |
| DED-IBD | *APEH* | 9.60E-02 | 9.60E-02 | 2.50E-12 | 1.42E+01 | 9.85E-46 | 9.63E-42 | Adipose-Subcutaneous | 479 |
| DED-IBD | *RNF123* | 2.05E-01 | 2.40E-01 | 4.80E-31 | -9.36E+00 | 7.66E-21 | 5.76E-18 | Adipose-Subcutaneous | 479 |
| DED-IBD | *MST1* | 6.81E-02 | 7.30E-02 | 1.10E-09 | 8.09E+00 | 5.85E-16 | 2.38E-13 | Adipose-Subcutaneous | 479 |
| DED-IBD | *GPX1* | 1.90E-02 | 2.10E-02 | 8.00E-04 | 4.18E+00 | 2.96E-05 | 1.37E-03 | Adipose-Subcutaneous | 479 |
| DED-PUD | *IZUMO1* | 2.20E-01 | 3.50E-01 | 2.40E-46 | 4.67E+00 | 2.97E-06 | 2.82E-04 | Adipose-Subcutaneous | 479 |
| DED-DD | *FOXP2* | 5.99E-02 | 7.30E-02 | 1.10E-09 | -1.38E+01 | 1.70E-43 | 1.67E-39 | Adipose-Subcutaneous | 479 |
| Uveitis-PUD | *IZUMO1* | 2.20E-01 | 3.50E-01 | 2.40E-46 | 8.57E+00 | 1.02E-17 | 1.42E-14 | Adipose-Subcutaneous | 479 |
| Myopia-IBD | *GGACT* | 1.46E-01 | 1.50E-01 | 3.70E-19 | -3.20E+00 | 1.36E-03 | 2.46E-02 | Adipose-Subcutaneous | 479 |
| Myopia-IBD | *ARMS2* | 4.62E-02 | 5.30E-02 | 2.10E-07 | -3.31E+00 | 9.45E-04 | 1.83E-02 | Adipose-Subcutaneous | 479 |
| Myopia-PUD | *GPD2* | 1.06E-01 | 1.10E-01 | 8.30E-14 | -7.16E+00 | 7.81E-13 | 2.18E-10 | Adipose-Subcutaneous | 479 |
| Myopia-PUD | *ACP1* | 1.40E-02 | 1.90E-02 | 1.40E-03 | -1.15E+01 | 2.15E-30 | 5.26E-27 | Adipose-Subcutaneous | 479 |
| Myopia-PUD | *ZBTB38* | 8.04E-02 | 8.00E-02 | 1.60E-10 | -1.52E+01 | 2.80E-52 | 2.74E-48 | Adipose-Subcutaneous | 479 |
| Myopia-DD | *ARHGAP15* | 8.34E-03 | 1.40E-02 | 5.80E-03 | 6.17E+00 | 6.81E-10 | 5.11E-08 | Adipose-Subcutaneous | 479 |
| Myopia-DD | *EFEMP1* | 8.58E-03 | 3.60E-02 | 1.60E-05 | -1.53E+01 | 4.02E-53 | 3.95E-49 | Adipose-Subcutaneous | 479 |
| Myopia-DD | *COL6A1* | 6.62E-02 | 6.60E-02 | 6.70E-09 | -7.27E+00 | 3.61E-13 | 3.85E-11 | Adipose-Subcutaneous | 479 |
| Myopia-DD | *KCNMA1* | 1.45E-01 | 1.40E-01 | 3.80E-18 | 3.57E+00 | 3.52E-04 | 6.21E-03 | Adipose-Subcutaneous | 479 |
| Myopia-DD | *ANTXR2* | 9.97E-02 | 1.00E-01 | 9.10E-13 | 1.18E+01 | 2.26E-32 | 2.02E-29 | Adipose-Subcutaneous | 479 |
| Myopia-DD | *CHRNB1* | 2.48E-01 | 3.00E-01 | 4.90E-39 | -1.27E+01 | 1.12E-36 | 2.20E-33 | Adipose-Subcutaneous | 479 |
| Myopia-DD | *CD55* | 3.51E-02 | 3.60E-02 | 1.90E-05 | 7.90E+00 | 2.68E-15 | 4.18E-13 | Adipose-Subcutaneous | 479 |
| PACG-IBD | *IRF5* | 1.37E-01 | 1.40E-01 | 3.10E-17 | 1.32E+01 | 5.07E-40 | 4.96E-36 | Adipose-Subcutaneous | 479 |
| PACG-DD | *COX15* | 1.72E-02 | 6.80E-02 | 4.20E-09 | -1.44E+01 | 2.59E-47 | 2.54E-43 | Adipose-Subcutaneous | 479 |
| PACG-DD | *ARHGAP15* | 8.34E-03 | 1.40E-02 | 5.80E-03 | 3.35E+00 | 8.21E-04 | 1.70E-02 | Adipose-Subcutaneous | 479 |
| PACG-DD | *EFEMP1* | 8.58E-03 | 3.60E-02 | 1.60E-05 | -6.78E+00 | 1.18E-11 | 2.58E-09 | Adipose-Subcutaneous | 479 |
| PACG-DD | *BCAS3* | 2.16E-02 | 2.20E-02 | 7.30E-04 | 4.51E+00 | 6.47E-06 | 3.19E-04 | Adipose-Subcutaneous | 479 |
| PACG-DD | *MED12L* | 4.28E-02 | 4.30E-02 | 2.90E-06 | -6.55E+00 | 5.76E-11 | 1.07E-08 | Adipose-Subcutaneous | 479 |
| Keratitis-DD | *COX15* | 1.72E-02 | 6.80E-02 | 4.20E-09 | -1.08E+01 | 2.10E-27 | 6.88E-24 | Adipose-Subcutaneous | 479 |
| Keratitis-DD | *ARHGAP15* | 8.34E-03 | 1.40E-02 | 5.80E-03 | 7.25E+00 | 4.24E-13 | 2.78E-10 | Adipose-Subcutaneous | 479 |
| Keratitis-DD | *S100A10* | 8.89E-02 | 9.60E-02 | 2.60E-12 | 4.75E+00 | 2.06E-06 | 2.47E-04 | Adipose-Subcutaneous | 479 |
| Cataract-GORD | *SEMA3F* | 9.98E-03 | 3.10E-02 | 7.10E-05 | -1.68E+01 | 3.92E-63 | 1.92E-59 | Adipose-Subcutaneous | 479 |
| Cataract-GORD | *RBM6* | 5.63E-01 | 5.60E-01 | 2.00E-88 | -1.80E+01 | 3.15E-72 | 3.08E-68 | Adipose-Subcutaneous | 479 |
| Cataract-GORD | *BTN2A1* | 1.47E-01 | 1.50E-01 | 1.90E-18 | 4.52E+00 | 6.11E-06 | 3.05E-04 | Adipose-Subcutaneous | 479 |
| Cataract-GORD | *MST1R* | 8.52E-02 | 8.50E-02 | 4.40E-11 | 1.65E+01 | 3.95E-61 | 1.29E-57 | Adipose-Subcutaneous | 479 |
| Cataract-IBD | *TCTA* | 3.48E-02 | 4.20E-02 | 3.50E-06 | 6.97E+00 | 3.13E-12 | 5.67E-10 | Adipose-Subcutaneous | 479 |
| Cataract-IBD | *NICN1* | 1.83E-01 | 2.00E-01 | 3.60E-25 | 1.02E+01 | 2.52E-24 | 1.64E-21 | Adipose-Subcutaneous | 479 |
| Cataract-IBD | *APEH* | 9.60E-02 | 9.60E-02 | 2.50E-12 | 1.34E+01 | 7.46E-41 | 7.29E-37 | Adipose-Subcutaneous | 479 |
| Cataract-IBD | *RNF123* | 2.05E-01 | 2.40E-01 | 4.80E-31 | -1.03E+01 | 8.91E-25 | 6.22E-22 | Adipose-Subcutaneous | 479 |
| Cataract-IBD | *MST1* | 6.81E-02 | 7.30E-02 | 1.10E-09 | 1.10E+01 | 2.71E-28 | 5.30E-25 | Adipose-Subcutaneous | 479 |
| Cataract-IBD | *GPX1* | 1.90E-02 | 2.10E-02 | 8.00E-04 | 6.30E+00 | 2.98E-10 | 3.99E-08 | Adipose-Subcutaneous | 479 |
| Cataract-IBS | *PABPC4* | 1.51E-01 | 1.50E-01 | 2.90E-19 | -4.09E+00 | 4.35E-05 | 2.93E-03 | Adipose-Subcutaneous | 479 |
| AMD-GORD | *ABT1* | 1.93E-02 | 4.70E-02 | 9.00E-06 | 6.34E+00 | 2.34E-10 | 1.92E-08 | Adipose-Visceral | 393 |
| AMD-GORD | *ZNF322* | 3.22E-01 | 3.40E-01 | 2.20E-37 | -1.48E+01 | 2.99E-49 | 7.77E-46 | Adipose-Visceral | 393 |
| AMD-GORD | *HMGN4* | 2.56E-02 | 5.20E-02 | 2.70E-06 | 1.34E+01 | 3.15E-41 | 3.51E-38 | Adipose-Visceral | 393 |
| AMD-IBD | *IRF5* | 1.49E-01 | 1.50E-01 | 1.20E-15 | 1.73E+01 | 9.40E-67 | 6.11E-64 | Adipose-Visceral | 393 |
| AMD-IBD | *ULK3* | 2.01E-01 | 2.00E-01 | 3.90E-21 | -1.72E+01 | 1.14E-66 | 6.84E-64 | Adipose-Visceral | 393 |
| AMD-IBD | *RPP25* | 1.58E-01 | 1.60E-01 | 8.50E-17 | -1.19E+01 | 1.64E-32 | 4.74E-30 | Adipose-Visceral | 393 |
| AMD-IBD | *FAM219B* | 4.48E-03 | 5.90E-03 | 6.90E-02 | 1.14E+01 | 6.29E-30 | 1.64E-27 | Adipose-Visceral | 393 |
| AMD-IBD | *MPI* | 1.03E-01 | 1.20E-01 | 9.80E-13 | 1.54E+01 | 1.63E-53 | 6.69E-51 | Adipose-Visceral | 393 |
| AMD-PUD | *IZUMO1* | 2.34E-01 | 4.00E-01 | 7.70E-45 | 1.05E+01 | 5.37E-26 | 8.38E-23 | Adipose-Visceral | 393 |
| DR-GORD | *RBM6* | 5.44E-01 | 5.40E-01 | 6.10E-69 | -1.21E+01 | 6.35E-34 | 1.42E-31 | Adipose-Visceral | 393 |
| DR-GORD | *WNT3* | 2.08E-03 | 6.30E-02 | 3.00E-07 | -9.99E+00 | 1.72E-23 | 2.49E-21 | Adipose-Visceral | 393 |
| DR-GORD | *KANSL1* | 1.17E-01 | 2.00E-01 | 3.10E-21 | -3.27E+00 | 1.07E-03 | 1.40E-02 | Adipose-Visceral | 393 |
| DR-GORD | *CRHR1* | 1.82E-01 | 1.80E-01 | 2.60E-19 | -1.78E+01 | 1.01E-70 | 8.19E-68 | Adipose-Visceral | 393 |
| DR-GORD | *GATAD2A* | -2.50E-03 | 1.20E-02 | 1.70E-02 | 5.87E+00 | 4.37E-09 | 2.12E-07 | Adipose-Visceral | 393 |
| DR-GORD | *LTBP3* | 5.10E-02 | 5.10E-02 | 3.60E-06 | 1.46E+01 | 3.41E-48 | 1.16E-45 | Adipose-Visceral | 393 |
| DR-GORD | *TTLL6* | 3.47E-02 | 7.00E-02 | 6.80E-08 | -9.49E+00 | 2.21E-21 | 3.08E-19 | Adipose-Visceral | 393 |
| DR-GORD | *KCNK7* | 4.58E-02 | 5.70E-02 | 1.10E-06 | -1.86E+01 | 5.94E-77 | 7.73E-74 | Adipose-Visceral | 393 |
| DR-GORD | *IP6K1* | 4.75E-02 | 4.70E-02 | 7.80E-06 | 7.78E+00 | 7.17E-15 | 6.91E-13 | Adipose-Visceral | 393 |
| DR-GORD | *MAPT* | 4.82E-02 | 4.80E-02 | 6.60E-06 | -1.76E+01 | 3.41E-69 | 2.05E-66 | Adipose-Visceral | 393 |
| DR-GORD | *PLEKHM1* | 2.82E-02 | 4.20E-02 | 2.30E-05 | -1.76E+01 | 1.06E-69 | 7.52E-67 | Adipose-Visceral | 393 |
| DR-IBD | *INPP5E* | 1.03E-01 | 1.00E-01 | 4.40E-11 | -3.51E+00 | 4.41E-04 | 8.44E-03 | Adipose-Visceral | 393 |
| DR-IBD | *GPSM1* | 3.66E-02 | 8.60E-02 | 1.70E-09 | -7.81E+00 | 5.78E-15 | 7.52E-13 | Adipose-Visceral | 393 |
| DR-IBD | *PEAK1* | 6.45E-02 | 1.10E-01 | 2.80E-11 | 3.17E+00 | 1.51E-03 | 2.22E-02 | Adipose-Visceral | 393 |
| DR-IBD | *CARD9* | 1.95E-02 | 2.00E-02 | 3.20E-03 | 1.73E+01 | 5.18E-67 | 2.69E-64 | Adipose-Visceral | 393 |
| DR-IBD | *DNLZ* | 2.81E-01 | 4.10E-01 | 9.40E-47 | -8.45E+00 | 2.86E-17 | 5.06E-15 | Adipose-Visceral | 393 |
| DR-PUD | *DMPK* | 7.46E-02 | 7.80E-02 | 1.10E-08 | -8.76E+00 | 2.02E-18 | 4.64E-16 | Adipose-Visceral | 393 |
| DR-PUD | *IZUMO1* | 2.34E-01 | 4.00E-01 | 7.70E-45 | 7.96E+00 | 1.76E-15 | 3.19E-13 | Adipose-Visceral | 393 |
| DR-PUD | *DMWD* | 1.13E-01 | 1.10E-01 | 4.20E-12 | -3.86E+00 | 1.14E-04 | 3.00E-03 | Adipose-Visceral | 393 |
| DR-IBS | *PPP2R3A* | 6.17E-02 | 6.20E-02 | 3.70E-07 | 1.57E+01 | 2.29E-55 | 3.00E-52 | Adipose-Visceral | 393 |
| DR-IBS | *PABPC4* | 1.17E-01 | 1.20E-01 | 1.90E-12 | 2.34E+01 | 2.51E-121 | 9.85E-118 | Adipose-Visceral | 393 |
| DR-IBS | *PPM1G* | 2.85E-02 | 2.80E-02 | 4.60E-04 | 1.17E+01 | 1.13E-31 | 4.22E-29 | Adipose-Visceral | 393 |
| DR-IBS | *DENND1A* | 2.42E-02 | 2.40E-02 | 1.20E-03 | 8.19E+00 | 2.59E-16 | 5.08E-14 | Adipose-Visceral | 393 |
| DR-IBS | *KRTCAP3* | 5.42E-02 | 6.90E-02 | 7.00E-08 | 4.24E+00 | 2.21E-05 | 9.37E-04 | Adipose-Visceral | 393 |
| DR-IBS | *TMEM106A* | -1.05E-03 | 2.60E-02 | 7.80E-04 | -5.97E+00 | 2.42E-09 | 2.16E-07 | Adipose-Visceral | 393 |
| DR-IBS | *NCR3LG1* | 2.62E-02 | 2.60E-02 | 7.50E-04 | -1.36E+01 | 2.36E-42 | 1.42E-39 | Adipose-Visceral | 393 |
| DED-IBD | *IRF5* | 1.49E-01 | 1.50E-01 | 1.20E-15 | 1.29E+01 | 2.45E-38 | 3.82E-35 | Adipose-Visceral | 393 |
| DED-IBD | *AMT* | 4.57E-01 | 4.60E-01 | 7.60E-55 | 4.60E+00 | 4.21E-06 | 2.49E-04 | Adipose-Visceral | 393 |
| DED-IBD | *NICN1* | 2.02E-01 | 2.20E-01 | 4.70E-23 | 8.79E+00 | 1.51E-18 | 6.93E-16 | Adipose-Visceral | 393 |
| DED-IBD | *APEH* | 2.64E-02 | 3.70E-02 | 7.70E-05 | 1.51E+01 | 9.10E-52 | 3.55E-48 | Adipose-Visceral | 393 |
| DED-IBD | *RNF123* | 1.37E-01 | 2.00E-01 | 1.30E-20 | -9.05E+00 | 1.40E-19 | 6.83E-17 | Adipose-Visceral | 393 |
| DED-IBD | *MST1* | 3.86E-02 | 5.80E-02 | 8.20E-07 | 9.87E+00 | 5.41E-23 | 4.22E-20 | Adipose-Visceral | 393 |
| DED-PUD | *IZUMO1* | 2.34E-01 | 4.00E-01 | 7.70E-45 | 4.67E+00 | 2.98E-06 | 2.77E-04 | Adipose-Visceral | 393 |
| DED-DD | *ARHGAP15* | 5.88E-02 | 5.90E-02 | 6.90E-07 | -3.02E+00 | 2.53E-03 | 3.92E-02 | Adipose-Visceral | 393 |
| DED-DD | *FOXP2* | 1.60E-02 | 1.60E-02 | 6.90E-03 | -8.25E+00 | 1.65E-16 | 7.62E-14 | Adipose-Visceral | 393 |
| Uveitis-PUD | *IZUMO1* | 2.34E-01 | 4.00E-01 | 7.70E-45 | 8.50E+00 | 1.97E-17 | 1.54E-14 | Adipose-Visceral | 393 |
| Uveitis-DD | *ARHGAP15* | 5.88E-02 | 5.90E-02 | 6.90E-07 | -5.15E+00 | 2.66E-07 | 3.03E-05 | Adipose-Visceral | 393 |
| Myopia-IBD | *GGACT* | 1.21E-01 | 1.30E-01 | 9.70E-14 | -3.64E+00 | 2.67E-04 | 6.59E-03 | Adipose-Visceral | 393 |
| Myopia-IBD | *ARMS2* | 5.75E-02 | 6.60E-02 | 1.50E-07 | -3.22E+00 | 1.28E-03 | 2.33E-02 | Adipose-Visceral | 393 |
| Myopia-PUD | *GPD2* | 4.46E-02 | 4.50E-02 | 1.40E-05 | -7.13E+00 | 1.01E-12 | 3.28E-10 | Adipose-Visceral | 393 |
| Myopia-PUD | *CNDP2* | 5.77E-02 | 9.10E-02 | 5.90E-10 | 4.53E+00 | 5.78E-06 | 3.99E-04 | Adipose-Visceral | 393 |
| Myopia-DD | *EFEMP1* | 3.04E-02 | 3.00E-02 | 3.00E-04 | -4.06E+01 | 0.00E+00 | 0.00E+00 | Adipose-Visceral | 393 |
| Myopia-DD | *COL6A1* | 8.66E-02 | 9.40E-02 | 3.00E-10 | -7.87E+00 | 3.50E-15 | 5.84E-13 | Adipose-Visceral | 393 |
| Myopia-DD | *GPR158* | 8.14E-03 | 2.00E-02 | 3.00E-03 | -6.95E+00 | 3.66E-12 | 4.29E-10 | Adipose-Visceral | 393 |
| Myopia-DD | *ANTXR2* | 5.98E-02 | 6.00E-02 | 5.60E-07 | 1.07E+01 | 8.85E-27 | 3.31E-24 | Adipose-Visceral | 393 |
| Myopia-DD | *CHRNB1* | 1.71E-01 | 2.50E-01 | 1.10E-26 | -3.79E+01 | 0.00E+00 | 0.00E+00 | Adipose-Visceral | 393 |
| Myopia-DD | *PCBP3* | 1.55E-01 | 1.80E-01 | 1.90E-18 | -7.73E+00 | 1.11E-14 | 1.81E-12 | Adipose-Visceral | 393 |
| Myopia-DD | *CD55* | 1.34E-01 | 1.30E-01 | 3.80E-14 | -1.19E+01 | 6.87E-33 | 4.90E-30 | Adipose-Visceral | 393 |
| Cataract-GORD | *SEMA3F* | 5.76E-02 | 5.80E-02 | 8.90E-07 | -1.81E+01 | 2.28E-73 | 8.90E-70 | Adipose-Visceral | 393 |
| Cataract-GORD | *RBM5* | 1.15E-02 | 2.10E-02 | 2.50E-03 | -1.78E+01 | 7.60E-71 | 1.98E-67 | Adipose-Visceral | 393 |
| Cataract-GORD | *RBM6* | 5.44E-01 | 5.40E-01 | 6.10E-69 | -1.77E+01 | 1.97E-70 | 3.84E-67 | Adipose-Visceral | 393 |
| Cataract-GORD | *MST1R* | 2.87E-02 | 3.00E-02 | 3.30E-04 | 1.85E+01 | 1.25E-76 | 9.76E-73 | Adipose-Visceral | 393 |
| Cataract-IBD | *NICN1* | 2.02E-01 | 2.20E-01 | 4.70E-23 | 1.09E+01 | 1.06E-27 | 1.03E-24 | Adipose-Visceral | 393 |
| Cataract-IBD | *APEH* | 2.64E-02 | 3.70E-02 | 7.70E-05 | 1.24E+01 | 1.52E-35 | 2.37E-32 | Adipose-Visceral | 393 |
| Cataract-IBD | *RNF123* | 1.37E-01 | 2.00E-01 | 1.30E-20 | -1.00E+01 | 1.43E-23 | 9.30E-21 | Adipose-Visceral | 393 |
| Cataract-IBD | *MST1* | 3.86E-02 | 5.80E-02 | 8.20E-07 | 1.20E+01 | 5.08E-33 | 6.61E-30 | Adipose-Visceral | 393 |
| Cataract-IBS | *PABPC4* | 1.17E-01 | 1.20E-01 | 1.90E-12 | 1.28E+01 | 2.84E-37 | 1.11E-33 | Adipose-Visceral | 393 |
| PACG-IBD | *IRF5* | 1.49E-01 | 1.50E-01 | 1.20E-15 | 1.32E+01 | 5.07E-40 | 1.98E-36 | Adipose-Visceral | 393 |
| PACG-IBD | *OTUD3* | 1.96E-01 | 2.80E-01 | 2.40E-30 | 3.13E+00 | 1.76E-03 | 4.14E-02 | Adipose-Visceral | 393 |
| PACG-DD | *EFEMP1* | 3.04E-02 | 3.00E-02 | 3.00E-04 | -1.50E+01 | 3.53E-51 | 2.77E-47 | Adipose-Visceral | 393 |
| Keratitis-DD | *ARHGAP15* | 5.88E-02 | 5.90E-02 | 6.90E-07 | -3.61E+00 | 3.01E-04 | 1.14E-02 | Adipose-Visceral | 393 |
| Keratitis-DD | *GPR158* | 8.14E-03 | 2.00E-02 | 3.00E-03 | -5.66E+00 | 1.48E-08 | 3.04E-06 | Adipose-Visceral | 393 |
| AMD-GORD | *ABT1* | 7.94E-02 | 8.50E-02 | 9.20E-10 | 5.64E+00 | 1.70E-08 | 1.26E-06 | Esophagus-Mucosa | 411 |
| AMD-GORD | *ZNF322* | 3.24E-01 | 4.30E-01 | 3.00E-52 | -8.76E+00 | 1.99E-18 | 4.65E-16 | Esophagus-Mucosa | 411 |
| AMD-GORD | *HMGN4* | 9.38E-02 | 9.40E-02 | 1.30E-10 | 4.77E+00 | 1.85E-06 | 8.61E-05 | Esophagus-Mucosa | 411 |
| AMD-IBD | *IRF5* | 2.52E-01 | 2.70E-01 | 3.20E-30 | 1.72E+01 | 1.88E-66 | 1.22E-63 | Esophagus-Mucosa | 411 |
| AMD-IBD | *ULK3* | 4.32E-02 | 1.00E-01 | 1.10E-11 | 3.30E+00 | 9.76E-04 | 2.11E-02 | Esophagus-Mucosa | 411 |
| AMD-IBD | *SCAMP2* | 1.58E-01 | 2.50E-01 | 8.20E-28 | 1.48E+01 | 8.18E-50 | 3.39E-47 | Esophagus-Mucosa | 411 |
| AMD-IBD | *LMAN1L* | 1.63E-01 | 1.80E-01 | 3.80E-20 | 1.60E+01 | 7.97E-58 | 4.04E-55 | Esophagus-Mucosa | 411 |
| AMD-IBD | *RPP25* | 6.11E-03 | 1.40E-02 | 9.80E-03 | -1.42E+01 | 5.50E-46 | 2.18E-43 | Esophagus-Mucosa | 411 |
| AMD-IBD | *FAM219B* | 7.31E-02 | 7.30E-02 | 1.50E-08 | 1.17E+01 | 8.61E-32 | 2.45E-29 | Esophagus-Mucosa | 411 |
| AMD-IBD | *MPI* | 7.05E-02 | 1.00E-01 | 1.10E-11 | 1.17E+01 | 1.15E-31 | 3.08E-29 | Esophagus-Mucosa | 411 |
| AMD-IBD | *RTEL1* | 5.48E-02 | 5.50E-02 | 9.50E-07 | 1.14E+01 | 2.83E-30 | 6.97E-28 | Esophagus-Mucosa | 411 |
| AMD-PUD | *RASIP1* | 1.72E-01 | 2.00E-01 | 4.60E-22 | 2.72E+01 | 4.15E-163 | 1.89E-159 | Esophagus-Mucosa | 411 |
| AMD-PUD | *MAPK10* | 1.50E-02 | 5.10E-02 | 2.20E-06 | 3.93E+00 | 8.41E-05 | 3.41E-03 | Esophagus-Mucosa | 411 |
| AMD-PUD | *NTN5* | 4.69E-02 | 1.30E-01 | 1.10E-14 | 2.10E+01 | 4.44E-98 | 1.35E-94 | Esophagus-Mucosa | 411 |
| AMD-PUD | *FUT1* | 9.53E-02 | 9.50E-02 | 9.60E-11 | -1.99E+01 | 3.28E-88 | 7.48E-85 | Esophagus-Mucosa | 411 |
| AMD-PUD | *FUT2* | 6.95E-01 | 7.00E-01 | 5.50E-108 | -3.33E+01 | 1.78E-243 | 1.62E-239 | Esophagus-Mucosa | 411 |
| AMD-PUD | *IZUMO1* | 2.24E-01 | 4.20E-01 | 9.30E-51 | 1.17E+01 | 1.13E-31 | 1.14E-28 | Esophagus-Mucosa | 411 |
| DR-GORD | *RBM6* | 5.43E-01 | 5.50E-01 | 1.50E-72 | -1.24E+01 | 1.83E-35 | 4.51E-33 | Esophagus-Mucosa | 411 |
| DR-GORD | *NSF* | 8.46E-02 | 1.10E-01 | 4.20E-12 | 1.13E+01 | 1.32E-29 | 2.87E-27 | Esophagus-Mucosa | 411 |
| DR-GORD | *KDM2B* | 9.45E-02 | 9.90E-02 | 3.70E-11 | 1.98E+01 | 1.96E-87 | 4.47E-84 | Esophagus-Mucosa | 411 |
| DR-GORD | *KANSL1* | 1.93E-01 | 2.50E-01 | 8.00E-28 | -6.25E+00 | 4.20E-10 | 2.21E-08 | Esophagus-Mucosa | 411 |
| DR-GORD | *CRHR1* | 1.14E-02 | 1.30E-02 | 1.30E-02 | 4.67E+00 | 3.06E-06 | 8.75E-05 | Esophagus-Mucosa | 411 |
| DR-GORD | *TCF7L2* | 1.01E-02 | 1.90E-02 | 3.00E-03 | 4.22E+00 | 2.48E-05 | 5.54E-04 | Esophagus-Mucosa | 411 |
| DR-GORD | *UBE2Z* | 6.94E-03 | 6.90E-03 | 5.00E-02 | 1.53E+01 | 4.07E-53 | 1.69E-50 | Esophagus-Mucosa | 411 |
| DR-GORD | *SNF8* | 4.07E-02 | 4.10E-02 | 2.20E-05 | 1.46E+01 | 2.54E-48 | 9.65E-46 | Esophagus-Mucosa | 411 |
| DR-GORD | *ARHGAP27* | 1.14E-01 | 1.10E-01 | 1.30E-12 | 1.76E+01 | 3.41E-69 | 2.07E-66 | Esophagus-Mucosa | 411 |
| DR-GORD | *GATAD2A* | 3.17E-02 | 4.20E-02 | 1.70E-05 | 1.40E+01 | 1.08E-44 | 3.40E-42 | Esophagus-Mucosa | 411 |
| DR-GORD | *IP6K1* | -1.11E-03 | 1.50E-02 | 8.00E-03 | 7.02E+00 | 2.16E-12 | 1.52E-10 | Esophagus-Mucosa | 411 |
| DR-GORD | *UBA7* | 2.46E-01 | 2.50E-01 | 4.30E-27 | -1.09E+01 | 1.35E-27 | 2.68E-25 | Esophagus-Mucosa | 411 |
| DR-GORD | *MAPT* | 4.39E-01 | 4.50E-01 | 5.00E-55 | -1.76E+01 | 1.48E-69 | 1.23E-66 | Esophagus-Mucosa | 411 |
| DR-GORD | *PLEKHM1* | 1.61E-01 | 2.10E-01 | 8.80E-23 | 1.67E+01 | 6.90E-63 | 3.70E-60 | Esophagus-Mucosa | 411 |
| DR-IBD | *PIK3C2B* | 1.12E-01 | 1.20E-01 | 2.10E-13 | 7.38E+00 | 1.63E-13 | 1.93E-11 | Esophagus-Mucosa | 411 |
| DR-IBD | *INPP5E* | 1.65E-01 | 1.70E-01 | 7.50E-19 | -1.37E+01 | 1.71E-42 | 8.21E-40 | Esophagus-Mucosa | 411 |
| DR-IBD | *GPSM1* | -3.24E-05 | 1.30E-02 | 1.20E-02 | -8.98E+00 | 2.80E-19 | 5.67E-17 | Esophagus-Mucosa | 411 |
| DR-IBD | *DNLZ* | 3.52E-01 | 5.00E-01 | 4.90E-63 | -6.64E+00 | 3.16E-11 | 2.94E-09 | Esophagus-Mucosa | 411 |
| DR-PUD | *DMPK* | 4.24E-02 | 6.10E-02 | 2.60E-07 | -4.38E+00 | 1.21E-05 | 4.28E-04 | Esophagus-Mucosa | 411 |
| DR-PUD | *RASIP1* | 1.72E-01 | 2.00E-01 | 4.60E-22 | 2.19E+01 | 1.97E-106 | 5.99E-103 | Esophagus-Mucosa | 411 |
| DR-PUD | *LAMC1* | 7.80E-02 | 7.80E-02 | 4.60E-09 | -1.37E+01 | 8.78E-43 | 1.60E-39 | Esophagus-Mucosa | 411 |
| DR-PUD | *FUT2* | 6.95E-01 | 7.00E-01 | 5.50E-108 | -2.93E+01 | 4.13E-188 | 3.77E-184 | Esophagus-Mucosa | 411 |
| DR-PUD | *SIX5* | 7.16E-02 | 7.20E-02 | 2.20E-08 | 8.94E+00 | 3.98E-19 | 8.44E-17 | Esophagus-Mucosa | 411 |
| DR-PUD | *UBE2E2* | 1.34E-02 | 2.30E-02 | 1.30E-03 | -3.56E+00 | 3.68E-04 | 7.84E-03 | Esophagus-Mucosa | 411 |
| DR-PUD | *IZUMO1* | 2.24E-01 | 4.20E-01 | 9.30E-51 | 8.75E+00 | 2.11E-18 | 4.09E-16 | Esophagus-Mucosa | 411 |
| DR-IBS | *PPP2R3A* | 6.64E-02 | 6.60E-02 | 7.10E-08 | -1.70E+01 | 1.24E-64 | 2.84E-61 | Esophagus-Mucosa | 411 |
| DR-IBS | *CAD* | 5.82E-02 | 6.00E-02 | 3.30E-07 | -5.91E+00 | 3.51E-09 | 2.77E-07 | Esophagus-Mucosa | 411 |
| DR-IBS | *PABPC4* | 1.43E-01 | 1.60E-01 | 2.10E-17 | 3.93E+00 | 8.32E-05 | 3.09E-03 | Esophagus-Mucosa | 411 |
| DR-IBS | *VAT1* | 5.26E-02 | 1.50E-01 | 5.80E-16 | 7.33E+00 | 2.30E-13 | 2.93E-11 | Esophagus-Mucosa | 411 |
| DR-IBS | *RND2* | 2.41E-01 | 2.40E-01 | 8.00E-27 | 1.49E+01 | 3.11E-50 | 2.59E-47 | Esophagus-Mucosa | 411 |
| DR-IBS | *NRBP1* | 1.23E-01 | 1.20E-01 | 1.30E-13 | 1.80E+01 | 1.23E-72 | 3.76E-69 | Esophagus-Mucosa | 411 |
| DR-IBS | *DENND1A* | 6.05E-02 | 6.00E-02 | 2.70E-07 | 1.44E+01 | 3.20E-47 | 2.26E-44 | Esophagus-Mucosa | 411 |
| DR-IBS | *GIN1* | 2.54E-02 | 2.50E-02 | 6.90E-04 | 1.16E+01 | 2.82E-31 | 1.08E-28 | Esophagus-Mucosa | 411 |
| DR-IBS | *PAM* | 6.48E-02 | 6.80E-02 | 5.30E-08 | -5.18E+00 | 2.22E-07 | 1.35E-05 | Esophagus-Mucosa | 411 |
| DR-IBS | *AP3S2* | 5.20E-01 | 5.70E-01 | 1.50E-77 | 3.73E+00 | 1.90E-04 | 6.38E-03 | Esophagus-Mucosa | 411 |
| DR-IBS | *UBE2E2* | 1.34E-02 | 2.30E-02 | 1.30E-03 | -5.31E+00 | 1.11E-07 | 7.02E-06 | Esophagus-Mucosa | 411 |
| DR-IBS | *NCR3LG1* | 3.31E-01 | 4.60E-01 | 4.60E-57 | -8.93E+00 | 4.31E-19 | 9.19E-17 | Esophagus-Mucosa | 411 |
| DED-IBD | *P4HA2* | 1.94E-01 | 2.00E-01 | 1.70E-21 | 1.09E+01 | 6.81E-28 | 6.21E-25 | Esophagus-Mucosa | 411 |
| DED-IBD | *IRF5* | 2.52E-01 | 2.70E-01 | 3.20E-30 | 1.28E+01 | 1.26E-37 | 2.87E-34 | Esophagus-Mucosa | 411 |
| DED-IBD | *AMT* | 4.06E-01 | 4.10E-01 | 6.70E-49 | 4.52E+00 | 6.28E-06 | 4.03E-04 | Esophagus-Mucosa | 411 |
| DED-IBD | *NICN1* | 8.41E-02 | 8.40E-02 | 1.20E-09 | 6.19E+00 | 5.86E-10 | 1.01E-07 | Esophagus-Mucosa | 411 |
| DED-IBD | *APEH* | 1.54E-01 | 1.60E-01 | 4.40E-17 | 1.30E+01 | 9.73E-39 | 2.96E-35 | Esophagus-Mucosa | 411 |
| DED-IBD | *RNF123* | 5.59E-02 | 5.90E-02 | 3.60E-07 | -1.15E+01 | 1.00E-30 | 1.14E-27 | Esophagus-Mucosa | 411 |
| DED-IBD | *OTUD3* | 9.16E-02 | 1.70E-01 | 4.70E-18 | 3.14E+00 | 1.70E-03 | 4.06E-02 | Esophagus-Mucosa | 411 |
| DED-IBD | *MST1* | 9.52E-02 | 2.00E-01 | 6.70E-22 | 1.15E+01 | 1.82E-30 | 1.84E-27 | Esophagus-Mucosa | 411 |
| DED-PUD | *RASIP1* | 1.72E-01 | 2.00E-01 | 4.60E-22 | 1.46E+01 | 2.78E-48 | 1.27E-44 | Esophagus-Mucosa | 411 |
| DED-PUD | *FUT2* | 6.95E-01 | 7.00E-01 | 5.50E-108 | -1.99E+01 | 2.18E-88 | 1.99E-84 | Esophagus-Mucosa | 411 |
| DED-PUD | *IZUMO1* | 2.24E-01 | 4.20E-01 | 9.30E-51 | 4.82E+00 | 1.45E-06 | 1.47E-04 | Esophagus-Mucosa | 411 |
| Uveitis-PUD | *RASIP1* | 1.72E-01 | 2.00E-01 | 4.60E-22 | 1.84E+01 | 5.85E-76 | 2.67E-72 | Esophagus-Mucosa | 411 |
| Uveitis-PUD | *FUT2* | 6.95E-01 | 7.00E-01 | 5.50E-108 | -1.89E+01 | 1.13E-79 | 1.03E-75 | Esophagus-Mucosa | 411 |
| Uveitis-PUD | *IZUMO1* | 2.24E-01 | 4.20E-01 | 9.30E-51 | 9.28E+00 | 1.73E-20 | 2.25E-17 | Esophagus-Mucosa | 411 |
| Myopia-IBD | *GGACT* | 1.62E-01 | 1.70E-01 | 1.10E-18 | -3.65E+00 | 2.67E-04 | 6.29E-03 | Esophagus-Mucosa | 411 |
| Myopia-PUD | *CNDP2* | 2.28E-01 | 2.90E-01 | 3.50E-32 | 1.24E+01 | 4.29E-35 | 1.30E-31 | Esophagus-Mucosa | 411 |
| Myopia-PUD | *FUT2* | 6.95E-01 | 7.00E-01 | 5.50E-108 | -1.68E+01 | 4.39E-63 | 4.00E-59 | Esophagus-Mucosa | 411 |
| Myopia-PUD | *ZBTB38* | 4.98E-02 | 5.00E-02 | 2.90E-06 | 7.42E+00 | 1.17E-13 | 3.68E-11 | Esophagus-Mucosa | 411 |
| Myopia-IBS | *KCNT2* | 5.15E-02 | 5.20E-02 | 2.00E-06 | -5.91E+00 | 3.52E-09 | 6.09E-07 | Esophagus-Mucosa | 411 |
| Myopia-DD | *CACNB2* | 8.53E-03 | 8.70E-03 | 3.30E-02 | -6.30E+00 | 3.00E-10 | 2.55E-08 | Esophagus-Mucosa | 411 |
| Myopia-DD | *CHRNB1* | 1.68E-01 | 1.90E-01 | 4.10E-21 | -2.51E+01 | 4.66E-139 | 4.27E-135 | Esophagus-Mucosa | 411 |
| Myopia-DD | *ZBTB4* | 1.47E-01 | 1.50E-01 | 4.60E-16 | -2.27E+01 | 3.08E-114 | 1.41E-110 | Esophagus-Mucosa | 411 |
| Myopia-DD | *PCBP3* | 1.95E-01 | 2.20E-01 | 1.80E-24 | -7.71E+00 | 1.22E-14 | 2.03E-12 | Esophagus-Mucosa | 411 |
| Myopia-DD | *CD55* | 1.12E-01 | 1.20E-01 | 1.10E-13 | -9.42E+00 | 4.32E-21 | 1.28E-18 | Esophagus-Mucosa | 411 |
| Myopia-DD | *AFAP1* | 5.16E-01 | 5.20E-01 | 9.10E-68 | -1.25E+01 | 1.30E-35 | 1.49E-32 | Esophagus-Mucosa | 411 |
| Myopia-DD | *S100A10* | 3.85E-02 | 3.80E-02 | 3.70E-05 | -1.47E+01 | 1.31E-48 | 2.00E-45 | Esophagus-Mucosa | 411 |
| Cataract-GORD | *RBM5* | 1.16E-02 | 1.60E-02 | 5.90E-03 | -1.73E+01 | 3.14E-67 | 9.55E-64 | Esophagus-Mucosa | 411 |
| Cataract-GORD | *RBM6* | 5.43E-01 | 5.50E-01 | 1.50E-72 | -1.79E+01 | 1.02E-71 | 4.65E-68 | Esophagus-Mucosa | 411 |
| Cataract-GORD | *MST1R* | 2.74E-02 | 3.50E-02 | 7.70E-05 | 1.85E+01 | 2.11E-76 | 1.92E-72 | Esophagus-Mucosa | 411 |
| Cataract-IBD | *NICN1* | 8.41E-02 | 8.40E-02 | 1.20E-09 | 9.01E+00 | 2.03E-19 | 7.41E-17 | Esophagus-Mucosa | 411 |
| Cataract-IBD | *APEH* | 1.54E-01 | 1.60E-01 | 4.40E-17 | 1.52E+01 | 2.07E-52 | 9.44E-49 | Esophagus-Mucosa | 411 |
| Cataract-IBD | *RNF123* | 5.59E-02 | 5.90E-02 | 3.60E-07 | -1.20E+01 | 4.03E-33 | 6.13E-30 | Esophagus-Mucosa | 411 |
| Cataract-IBD | *MST1* | 9.52E-02 | 2.00E-01 | 6.70E-22 | 1.44E+01 | 8.45E-47 | 2.57E-43 | Esophagus-Mucosa | 411 |
| PACG-IBD | *IRF5* | 2.52E-01 | 2.70E-01 | 3.20E-30 | 1.31E+01 | 4.85E-39 | 4.42E-35 | Esophagus-Mucosa | 411 |
| PACG-IBD | *OTUD3* | 9.16E-02 | 1.70E-01 | 4.70E-18 | 4.10E+00 | 4.15E-05 | 1.85E-03 | Esophagus-Mucosa | 411 |
| Keratitis-DD | *S100A10* | 3.85E-02 | 3.80E-02 | 3.70E-05 | -2.12E+01 | 2.06E-99 | 1.89E-95 | Esophagus-Mucosa | 411 |
| AMD-GORD | *ABT1* | 3.30E-02 | 3.50E-02 | 1.30E-04 | 5.55E+00 | 2.94E-08 | 2.10E-06 | Esophagus-Muscularis | 385 |
| AMD-GORD | *ZNF322* | 3.95E-01 | 4.80E-01 | 3.00E-56 | -4.29E+00 | 1.78E-05 | 6.43E-04 | Esophagus-Muscularis | 385 |
| AMD-GORD | *HMGN4* | 2.54E-02 | 3.60E-02 | 9.80E-05 | 1.30E+01 | 2.23E-38 | 2.47E-35 | Esophagus-Muscularis | 385 |
| AMD-IBD | *CSK* | 1.26E-02 | 3.30E-02 | 2.10E-04 | -1.64E+01 | 1.21E-60 | 6.70E-58 | Esophagus-Muscularis | 385 |
| AMD-IBD | *KIF21B* | 1.02E-01 | 1.10E-01 | 1.80E-11 | 1.49E+01 | 2.60E-50 | 1.00E-47 | Esophagus-Muscularis | 385 |
| AMD-IBD | *SLC2A4RG* | 2.55E-03 | 7.50E-03 | 4.80E-02 | 1.02E+01 | 1.45E-24 | 3.21E-22 | Esophagus-Muscularis | 385 |
| AMD-IBD | *IRF5* | 1.23E-01 | 1.20E-01 | 8.00E-13 | 1.73E+01 | 9.40E-67 | 6.40E-64 | Esophagus-Muscularis | 385 |
| AMD-IBD | *SCAMP2* | 2.81E-02 | 2.80E-02 | 5.60E-04 | 1.13E+01 | 1.44E-29 | 3.75E-27 | Esophagus-Muscularis | 385 |
| AMD-IBD | *RPP25* | 5.31E-02 | 5.60E-02 | 1.70E-06 | -1.28E+01 | 2.27E-37 | 7.44E-35 | Esophagus-Muscularis | 385 |
| AMD-IBD | *FAM219B* | 1.30E-02 | 1.50E-02 | 8.40E-03 | 1.54E+01 | 2.55E-53 | 1.08E-50 | Esophagus-Muscularis | 385 |
| AMD-IBD | *MPI* | 7.31E-02 | 8.50E-02 | 3.70E-09 | 1.50E+01 | 9.35E-51 | 3.76E-48 | Esophagus-Muscularis | 385 |
| AMD-IBD | *STMN3* | 3.79E-02 | 5.20E-02 | 3.50E-06 | -6.83E+00 | 8.76E-12 | 1.16E-09 | Esophagus-Muscularis | 385 |
| AMD-IBD | *LIME1* | 1.25E-01 | 1.50E-01 | 1.50E-15 | -3.02E+00 | 2.56E-03 | 4.94E-02 | Esophagus-Muscularis | 385 |
| AMD-IBD | *RTEL1* | 1.01E-01 | 1.30E-01 | 1.00E-13 | 1.55E+01 | 5.85E-54 | 2.73E-51 | Esophagus-Muscularis | 385 |
| AMD-PUD | *RASIP1* | 7.56E-02 | 7.60E-02 | 2.50E-08 | 3.26E+01 | 1.20E-233 | 1.06E-229 | Esophagus-Muscularis | 385 |
| AMD-PUD | *MAPK10* | 3.00E-02 | 3.30E-02 | 1.80E-04 | 5.57E+00 | 2.60E-08 | 3.71E-06 | Esophagus-Muscularis | 385 |
| AMD-PUD | *NTN5* | 6.90E-02 | 1.30E-01 | 4.30E-13 | 1.55E+01 | 4.02E-54 | 1.19E-50 | Esophagus-Muscularis | 385 |
| AMD-PUD | *MAMSTR* | 6.28E-02 | 7.40E-02 | 3.30E-08 | 2.98E+01 | 5.97E-195 | 2.64E-191 | Esophagus-Muscularis | 385 |
| DR-GORD | *RBM6* | 5.64E-01 | 5.60E-01 | 2.70E-71 | -1.21E+01 | 6.35E-34 | 1.61E-31 | Esophagus-Muscularis | 385 |
| DR-GORD | *NSF* | 4.47E-02 | 6.80E-02 | 1.20E-07 | 8.14E+00 | 4.08E-16 | 4.46E-14 | Esophagus-Muscularis | 385 |
| DR-GORD | *WNT3* | 1.19E-01 | 1.50E-01 | 1.50E-15 | -1.34E+01 | 6.66E-41 | 1.84E-38 | Esophagus-Muscularis | 385 |
| DR-GORD | *CRHR1* | 8.27E-02 | 8.30E-02 | 5.50E-09 | -1.76E+01 | 3.41E-69 | 2.16E-66 | Esophagus-Muscularis | 385 |
| DR-GORD | *SNF8* | 1.14E-02 | 1.50E-02 | 8.70E-03 | 1.51E+01 | 2.40E-51 | 9.24E-49 | Esophagus-Muscularis | 385 |
| DR-GORD | *LTBP3* | 3.29E-03 | 1.60E-02 | 7.00E-03 | 4.72E+00 | 2.33E-06 | 6.41E-05 | Esophagus-Muscularis | 385 |
| DR-GORD | *TTLL6* | 4.57E-02 | 6.30E-02 | 3.80E-07 | -9.73E+00 | 2.16E-22 | 3.68E-20 | Esophagus-Muscularis | 385 |
| DR-GORD | *KCNK7* | 7.24E-02 | 7.20E-02 | 4.90E-08 | -2.16E+01 | 8.23E-104 | 1.82E-100 | Esophagus-Muscularis | 385 |
| DR-GORD | *EHBP1L1* | 1.31E-03 | 1.40E-02 | 1.20E-02 | 7.00E+00 | 2.64E-12 | 1.93E-10 | Esophagus-Muscularis | 385 |
| DR-GORD | *IP6K1* | 3.64E-02 | 3.60E-02 | 9.70E-05 | 7.93E+00 | 2.17E-15 | 2.29E-13 | Esophagus-Muscularis | 385 |
| DR-GORD | *ZNF518B* | 1.13E-01 | 1.10E-01 | 5.80E-12 | 7.86E+00 | 3.91E-15 | 3.98E-13 | Esophagus-Muscularis | 385 |
| DR-GORD | *UBA7* | 6.31E-02 | 6.30E-02 | 3.60E-07 | -1.09E+01 | 1.35E-27 | 2.78E-25 | Esophagus-Muscularis | 385 |
| DR-GORD | *MAPT* | 1.58E-02 | 6.10E-02 | 5.60E-07 | 6.00E+00 | 1.92E-09 | 9.50E-08 | Esophagus-Muscularis | 385 |
| DR-GORD | *PLEKHM1* | 6.39E-02 | 6.40E-02 | 3.00E-07 | -1.37E+01 | 6.72E-43 | 1.98E-40 | Esophagus-Muscularis | 385 |
| DR-IBD | *UBE3C* | 4.24E-02 | 4.20E-02 | 2.70E-05 | 5.25E+00 | 1.56E-07 | 7.47E-06 | Esophagus-Muscularis | 385 |
| DR-IBD | *INPP5E* | 1.42E-01 | 1.40E-01 | 1.20E-14 | -3.51E+00 | 4.41E-04 | 8.12E-03 | Esophagus-Muscularis | 385 |
| DR-IBD | *GPSM1* | 1.81E-02 | 3.20E-02 | 2.50E-04 | -1.27E+01 | 5.61E-37 | 3.31E-34 | Esophagus-Muscularis | 385 |
| DR-IBD | *SNAPC4* | 1.18E-01 | 1.70E-01 | 6.10E-18 | 7.49E+00 | 6.82E-14 | 9.02E-12 | Esophagus-Muscularis | 385 |
| DR-PUD | *DMPK* | 2.45E-02 | 2.90E-02 | 4.90E-04 | -7.82E+00 | 5.45E-15 | 7.79E-13 | Esophagus-Muscularis | 385 |
| DR-PUD | *RASIP1* | 7.56E-02 | 7.60E-02 | 2.50E-08 | 2.84E+01 | 5.34E-177 | 4.73E-173 | Esophagus-Muscularis | 385 |
| DR-PUD | *FBXL20* | 1.84E-02 | 4.90E-02 | 7.10E-06 | 9.92E+00 | 3.46E-23 | 8.76E-21 | Esophagus-Muscularis | 385 |
| DR-PUD | *LAMC1* | 8.09E-02 | 9.10E-02 | 9.00E-10 | -1.57E+01 | 7.44E-56 | 1.65E-52 | Esophagus-Muscularis | 385 |
| DR-PUD | *MAMSTR* | 6.28E-02 | 7.40E-02 | 3.30E-08 | 2.54E+01 | 3.40E-142 | 1.51E-138 | Esophagus-Muscularis | 385 |
| DR-PUD | *IZUMO1* | 1.04E-01 | 1.40E-01 | 4.80E-14 | 5.26E+00 | 1.43E-07 | 8.12E-06 | Esophagus-Muscularis | 385 |
| DR-IBS | *BRCA1* | 3.50E-02 | 3.70E-02 | 9.50E-05 | 1.24E+01 | 2.42E-35 | 1.35E-32 | Esophagus-Muscularis | 385 |
| DR-IBS | *PABPC4* | 4.81E-02 | 4.80E-02 | 8.40E-06 | 2.34E+01 | 2.51E-121 | 5.58E-118 | Esophagus-Muscularis | 385 |
| DR-IBS | *MTMR3* | 1.25E-01 | 1.20E-01 | 5.70E-13 | 6.33E+00 | 2.38E-10 | 2.52E-08 | Esophagus-Muscularis | 385 |
| DR-IBS | *VAT1* | 6.48E-02 | 6.50E-02 | 2.50E-07 | 1.48E+01 | 2.61E-49 | 2.32E-46 | Esophagus-Muscularis | 385 |
| DR-IBS | *RND2* | 4.54E-02 | 4.50E-02 | 1.50E-05 | -1.47E+01 | 3.98E-49 | 3.22E-46 | Esophagus-Muscularis | 385 |
| DR-IBS | *SNX17* | 6.22E-03 | 1.80E-02 | 4.50E-03 | 3.90E+00 | 9.63E-05 | 3.40E-03 | Esophagus-Muscularis | 385 |
| DR-IBS | *DENND1A* | 6.23E-02 | 6.20E-02 | 4.20E-07 | 1.29E+01 | 3.80E-38 | 2.25E-35 | Esophagus-Muscularis | 385 |
| DR-IBS | *BMP8A* | 7.67E-02 | 8.00E-02 | 1.10E-08 | 3.31E+00 | 9.44E-04 | 2.47E-02 | Esophagus-Muscularis | 385 |
| DR-IBS | *TMEM106A* | 5.84E-02 | 5.80E-02 | 9.60E-07 | -1.60E+01 | 7.48E-58 | 8.32E-55 | Esophagus-Muscularis | 385 |
| DR-IBS | *NCR3LG1* | 6.67E-02 | 6.70E-02 | 1.60E-07 | -1.09E+01 | 1.02E-27 | 4.13E-25 | Esophagus-Muscularis | 385 |
| DR-IBS | *NBR1* | 5.03E-02 | 5.00E-02 | 5.30E-06 | 1.55E+01 | 5.43E-54 | 5.37E-51 | Esophagus-Muscularis | 385 |
| DED-IBD | *P4HA2* | 8.64E-02 | 9.00E-02 | 1.10E-09 | 1.18E+01 | 3.50E-32 | 4.43E-29 | Esophagus-Muscularis | 385 |
| DED-IBD | *IRF5* | 1.23E-01 | 1.20E-01 | 8.00E-13 | 1.29E+01 | 2.45E-38 | 4.34E-35 | Esophagus-Muscularis | 385 |
| DED-IBD | *AMT* | 4.35E-01 | 4.60E-01 | 8.30E-54 | 3.66E+00 | 2.54E-04 | 8.79E-03 | Esophagus-Muscularis | 385 |
| DED-IBD | *TCTA* | 4.97E-02 | 5.80E-02 | 1.10E-06 | 4.24E+00 | 2.20E-05 | 1.10E-03 | Esophagus-Muscularis | 385 |
| DED-IBD | *NICN1* | 1.57E-01 | 1.60E-01 | 8.10E-17 | 5.26E+00 | 1.41E-07 | 1.40E-05 | Esophagus-Muscularis | 385 |
| DED-IBD | *APEH* | -2.54E-03 | 1.80E-02 | 5.10E-03 | 5.87E+00 | 4.29E-09 | 6.44E-07 | Esophagus-Muscularis | 385 |
| DED-IBD | *RNF123* | 1.64E-01 | 1.70E-01 | 8.90E-18 | -1.12E+01 | 4.82E-29 | 5.34E-26 | Esophagus-Muscularis | 385 |
| DED-IBD | *MST1* | 4.23E-02 | 8.70E-02 | 2.10E-09 | 7.50E+00 | 6.33E-14 | 2.08E-11 | Esophagus-Muscularis | 385 |
| DED-PUD | *RASIP1* | 7.56E-02 | 7.60E-02 | 2.50E-08 | 1.99E+01 | 2.72E-88 | 1.20E-84 | Esophagus-Muscularis | 385 |
| DED-PUD | *MAMSTR* | 6.28E-02 | 7.40E-02 | 3.30E-08 | 2.21E+01 | 2.95E-108 | 2.61E-104 | Esophagus-Muscularis | 385 |
| DED-DD | *ARHGAP15* | 7.98E-02 | 8.40E-02 | 3.80E-09 | -4.24E+00 | 2.24E-05 | 8.27E-04 | Esophagus-Muscularis | 385 |
| Uveitis-PUD | *RASIP1* | 7.56E-02 | 7.60E-02 | 2.50E-08 | 1.83E+01 | 5.50E-75 | 2.44E-71 | Esophagus-Muscularis | 385 |
| Uveitis-PUD | *MAMSTR* | 6.28E-02 | 7.40E-02 | 3.30E-08 | 1.66E+01 | 1.01E-61 | 2.98E-58 | Esophagus-Muscularis | 385 |
| Uveitis-PUD | *IZUMO1* | 1.04E-01 | 1.40E-01 | 4.80E-14 | 7.37E+00 | 1.66E-13 | 9.80E-11 | Esophagus-Muscularis | 385 |
| Uveitis-DD | *ARHGAP15* | 7.98E-02 | 8.40E-02 | 3.80E-09 | -8.99E+00 | 2.42E-19 | 4.31E-16 | Esophagus-Muscularis | 385 |
| Myopia-PUD | *CNDP2* | 5.90E-02 | 1.20E-01 | 3.20E-12 | 4.07E+00 | 4.64E-05 | 2.05E-03 | Esophagus-Muscularis | 385 |
| Myopia-PUD | *ZBTB38* | 3.90E-02 | 4.20E-02 | 3.00E-05 | -8.53E+00 | 1.42E-17 | 8.99E-15 | Esophagus-Muscularis | 385 |
| Myopia-PUD | *KCNQ5* | 1.66E-01 | 2.20E-01 | 3.00E-22 | -6.23E+00 | 4.72E-10 | 9.29E-08 | Esophagus-Muscularis | 385 |
| Myopia-DD | *ARHGAP15* | 7.98E-02 | 8.40E-02 | 3.80E-09 | -9.45E+00 | 3.29E-21 | 1.05E-18 | Esophagus-Muscularis | 385 |
| Myopia-DD | *COL6A1* | 1.43E-01 | 1.60E-01 | 3.90E-16 | -3.00E+00 | 2.68E-03 | 3.38E-02 | Esophagus-Muscularis | 385 |
| Myopia-DD | *GPR158* | 6.85E-02 | 6.90E-02 | 1.10E-07 | -8.25E+00 | 1.62E-16 | 3.20E-14 | Esophagus-Muscularis | 385 |
| Myopia-DD | *ANTXR2* | 5.53E-03 | 4.60E-02 | 1.30E-05 | 9.14E+00 | 6.35E-20 | 1.82E-17 | Esophagus-Muscularis | 385 |
| Myopia-DD | *CHRNB1* | 1.68E-01 | 1.80E-01 | 1.60E-18 | -1.30E+01 | 8.65E-39 | 1.10E-35 | Esophagus-Muscularis | 385 |
| Myopia-DD | *ZBTB4* | 1.74E-02 | 2.80E-02 | 5.40E-04 | -7.02E+00 | 2.29E-12 | 2.61E-10 | Esophagus-Muscularis | 385 |
| Myopia-DD | *PCBP3* | 1.99E-01 | 2.40E-01 | 7.60E-25 | -8.70E+00 | 3.20E-18 | 7.49E-16 | Esophagus-Muscularis | 385 |
| Myopia-DD | *CD55* | 1.58E-01 | 1.60E-01 | 2.30E-16 | 6.77E+00 | 1.27E-11 | 1.31E-09 | Esophagus-Muscularis | 385 |
| Myopia-DD | *S100A10* | 6.99E-02 | 1.20E-01 | 6.50E-13 | 4.58E+00 | 4.61E-06 | 1.51E-04 | Esophagus-Muscularis | 385 |
| Cataract-GORD | *SEMA3F* | 1.82E-02 | 2.80E-02 | 5.30E-04 | -1.15E+01 | 1.26E-30 | 5.87E-28 | Esophagus-Muscularis | 385 |
| Cataract-GORD | *RBM6* | 5.64E-01 | 5.60E-01 | 2.70E-71 | -1.77E+01 | 1.97E-70 | 5.82E-67 | Esophagus-Muscularis | 385 |
| Cataract-GORD | *MON1A* | 1.04E-02 | 1.50E-02 | 9.70E-03 | -1.85E+01 | 3.60E-76 | 3.19E-72 | Esophagus-Muscularis | 385 |
| Cataract-GORD | *MST1R* | 5.86E-02 | 6.50E-02 | 2.30E-07 | 1.81E+01 | 6.91E-73 | 3.06E-69 | Esophagus-Muscularis | 385 |
| Cataract-IBD | *TCTA* | 4.97E-02 | 5.80E-02 | 1.10E-06 | 7.08E+00 | 1.48E-12 | 2.62E-10 | Esophagus-Muscularis | 385 |
| Cataract-IBD | *NICN1* | 1.57E-01 | 1.60E-01 | 8.10E-17 | 7.74E+00 | 1.00E-14 | 2.21E-12 | Esophagus-Muscularis | 385 |
| Cataract-IBD | *APEH* | -2.54E-03 | 1.80E-02 | 5.10E-03 | 6.54E+00 | 6.13E-11 | 8.90E-09 | Esophagus-Muscularis | 385 |
| Cataract-IBD | *RNF123* | 1.64E-01 | 1.70E-01 | 8.90E-18 | -1.30E+01 | 8.64E-39 | 1.91E-35 | Esophagus-Muscularis | 385 |
| Cataract-IBD | *MST1* | 4.23E-02 | 8.70E-02 | 2.10E-09 | 9.29E+00 | 1.50E-20 | 6.99E-18 | Esophagus-Muscularis | 385 |
| Cataract-IBS | *PABPC4* | 4.81E-02 | 4.80E-02 | 8.40E-06 | 1.28E+01 | 2.84E-37 | 6.32E-34 | Esophagus-Muscularis | 385 |
| Cataract-DD | *ARHGAP15* | 7.98E-02 | 8.40E-02 | 3.80E-09 | -6.93E+00 | 4.35E-12 | 1.11E-09 | Esophagus-Muscularis | 385 |
| Cataract-DD | *ARHGAP15* | 7.98E-02 | 8.40E-02 | 3.80E-09 | -4.90E+00 | 9.64E-07 | 6.13E-05 | Esophagus-Muscularis | 385 |
| Keratitis-IBS | *ERI3* | 5.25E-03 | 2.10E-02 | 2.70E-03 | -3.46E+00 | 5.46E-04 | 4.42E-02 | Esophagus-Muscularis | 385 |
| Keratitis-DD | *GPR158* | 6.85E-02 | 6.90E-02 | 1.10E-07 | -1.22E+01 | 4.31E-34 | 3.83E-30 | Esophagus-Muscularis | 385 |
| Keratitis-DD | *S100A10* | 6.99E-02 | 1.20E-01 | 6.50E-13 | 7.84E+00 | 4.59E-15 | 5.10E-12 | Esophagus-Muscularis | 385 |
| AMD-GORD | *ZNF322* | 2.99E-01 | 4.40E-01 | 1.30E-36 | -9.19E+00 | 3.79E-20 | 7.95E-18 | EGJ | 275 |
| AMD-IBD | *IRF5* | 1.17E-01 | 1.20E-01 | 3.20E-09 | 1.97E+01 | 3.89E-86 | 2.61E-83 | EGJ | 275 |
| AMD-IBD | *ULK3* | 9.08E-02 | 9.10E-02 | 2.10E-07 | -1.74E+01 | 5.55E-68 | 3.10E-65 | EGJ | 275 |
| AMD-IBD | *RPP25* | 7.32E-02 | 7.30E-02 | 3.20E-06 | -1.13E+01 | 1.44E-29 | 3.72E-27 | EGJ | 275 |
| AMD-IBD | *FAM219B* | 3.00E-02 | 3.00E-02 | 2.30E-03 | 1.78E+01 | 3.26E-71 | 1.99E-68 | EGJ | 275 |
| AMD-IBD | *MPI* | 9.01E-02 | 9.00E-02 | 2.40E-07 | 1.48E+01 | 2.94E-49 | 1.23E-46 | EGJ | 275 |
| AMD-IBD | *RTEL1* | 1.75E-01 | 2.10E-01 | 5.00E-16 | 1.66E+01 | 3.69E-62 | 1.90E-59 | EGJ | 275 |
| AMD-PUD | *RASIP1* | 7.75E-02 | 1.10E-01 | 1.80E-08 | 3.00E+01 | 1.64E-197 | 1.10E-193 | EGJ | 275 |
| AMD-PUD | *NTN5* | 6.88E-02 | 1.60E-01 | 2.80E-12 | 2.52E+01 | 1.38E-140 | 3.09E-137 | EGJ | 275 |
| AMD-PUD | *MAMSTR* | 1.33E-01 | 1.50E-01 | 1.20E-11 | 2.82E+01 | 8.94E-175 | 3.00E-171 | EGJ | 275 |
| AMD-PUD | *IZUMO1* | 1.38E-01 | 1.60E-01 | 3.20E-12 | 1.60E+01 | 7.99E-58 | 1.34E-54 | EGJ | 275 |
| DR-GORD | *RBM6* | 5.13E-01 | 5.10E-01 | 1.00E-44 | -1.25E+01 | 5.08E-36 | 1.03E-33 | EGJ | 275 |
| DR-GORD | *NSF* | 3.30E-02 | 4.80E-02 | 1.40E-04 | 1.00E+01 | 1.15E-23 | 1.48E-21 | EGJ | 275 |
| DR-GORD | *KDM2B* | 5.87E-02 | 5.90E-02 | 2.60E-05 | 1.60E+01 | 1.15E-57 | 4.54E-55 | EGJ | 275 |
| DR-GORD | *WNT3* | 1.67E-01 | 1.80E-01 | 1.00E-13 | -1.44E+01 | 3.68E-47 | 9.88E-45 | EGJ | 275 |
| DR-GORD | *CRHR1* | 5.06E-02 | 5.20E-02 | 7.70E-05 | -1.76E+01 | 1.98E-69 | 1.11E-66 | EGJ | 275 |
| DR-GORD | *SNF8* | 8.35E-02 | 8.40E-02 | 6.50E-07 | 1.69E+01 | 9.09E-64 | 4.36E-61 | EGJ | 275 |
| DR-GORD | *TTLL6* | 3.87E-02 | 1.00E-01 | 3.60E-08 | -1.28E+01 | 3.04E-37 | 6.38E-35 | EGJ | 275 |
| DR-GORD | *KCNK7* | 1.55E-02 | 2.30E-02 | 7.00E-03 | -2.16E+01 | 6.18E-104 | 1.38E-100 | EGJ | 275 |
| DR-GORD | *MAPT* | 1.99E-02 | 2.20E-02 | 8.10E-03 | 7.87E+00 | 3.64E-15 | 2.98E-13 | EGJ | 275 |
| DR-IBD | *INPP5E* | 2.65E-02 | 3.00E-02 | 2.30E-03 | -5.03E+00 | 4.87E-07 | 2.35E-05 | EGJ | 275 |
| DR-PUD | *RASIP1* | 7.75E-02 | 1.10E-01 | 1.80E-08 | 2.53E+01 | 1.38E-141 | 9.27E-138 | EGJ | 275 |
| DR-PUD | *MAMSTR* | 1.33E-01 | 1.50E-01 | 1.20E-11 | 2.44E+01 | 8.29E-132 | 2.78E-128 | EGJ | 275 |
| DR-PUD | *IZUMO1* | 1.38E-01 | 1.60E-01 | 3.20E-12 | 1.22E+01 | 5.08E-34 | 4.87E-31 | EGJ | 275 |
| DR-IBS | *BRCA1* | 3.50E-02 | 3.70E-02 | 9.50E-05 | 1.24E+01 | 2.42E-35 | 1.35E-32 | EGJ | 275 |
| DR-IBS | *PABPC4* | 4.81E-02 | 4.80E-02 | 8.40E-06 | 2.34E+01 | 2.51E-121 | 5.58E-118 | EGJ | 275 |
| DR-IBS | *MTMR3* | 1.25E-01 | 1.20E-01 | 5.70E-13 | 6.33E+00 | 2.38E-10 | 2.52E-08 | EGJ | 275 |
| DR-IBS | *VAT1* | 6.48E-02 | 6.50E-02 | 2.50E-07 | 1.48E+01 | 2.61E-49 | 2.32E-46 | EGJ | 275 |
| DR-IBS | *RND2* | 4.54E-02 | 4.50E-02 | 1.50E-05 | -1.47E+01 | 3.98E-49 | 3.22E-46 | EGJ | 275 |
| DR-IBS | *SNX17* | 6.22E-03 | 1.80E-02 | 4.50E-03 | 3.90E+00 | 9.63E-05 | 3.40E-03 | EGJ | 275 |
| DR-IBS | *DENND1A* | 6.23E-02 | 6.20E-02 | 4.20E-07 | 1.29E+01 | 3.80E-38 | 2.25E-35 | EGJ | 275 |
| DR-IBS | *BMP8A* | 7.67E-02 | 8.00E-02 | 1.10E-08 | 3.31E+00 | 9.44E-04 | 2.47E-02 | EGJ | 275 |
| DR-IBS | *TMEM106A* | 5.84E-02 | 5.80E-02 | 9.60E-07 | -1.60E+01 | 7.48E-58 | 8.32E-55 | EGJ | 275 |
| DR-IBS | *NCR3LG1* | 6.67E-02 | 6.70E-02 | 1.60E-07 | -1.09E+01 | 1.02E-27 | 4.13E-25 | EGJ | 275 |
| DR-IBS | *NBR1* | 5.03E-02 | 5.00E-02 | 5.30E-06 | 1.55E+01 | 5.43E-54 | 5.37E-51 | EGJ | 275 |
| DED-IBD | *P4HA2* | 9.37E-02 | 9.50E-02 | 1.10E-07 | 1.10E+01 | 2.34E-28 | 3.14E-25 | EGJ | 275 |
| DED-IBD | *IRF5* | 1.17E-01 | 1.20E-01 | 3.20E-09 | 1.45E+01 | 7.37E-48 | 1.65E-44 | EGJ | 275 |
| DED-IBD | *AMT* | 3.67E-01 | 3.70E-01 | 1.50E-29 | 3.98E+00 | 6.96E-05 | 2.70E-03 | EGJ | 275 |
| DED-IBD | *NICN1* | 1.83E-01 | 1.80E-01 | 7.10E-14 | 4.03E+00 | 5.66E-05 | 2.28E-03 | EGJ | 275 |
| DED-IBD | *RNF123* | 8.98E-02 | 1.50E-01 | 1.40E-11 | -9.51E+00 | 1.88E-21 | 1.58E-18 | EGJ | 275 |
| DED-IBD | *MST1* | 4.58E-02 | 4.60E-02 | 2.10E-04 | 7.76E+00 | 8.33E-15 | 3.11E-12 | EGJ | 275 |
| DED-PUD | *RASIP1* | 7.75E-02 | 1.10E-01 | 1.80E-08 | 2.01E+01 | 1.13E-89 | 3.79E-86 | EGJ | 275 |
| DED-PUD | *MAMSTR* | 1.33E-01 | 1.50E-01 | 1.20E-11 | 2.17E+01 | 1.55E-104 | 1.04E-100 | EGJ | 275 |
| DED-PUD | *IZUMO1* | 1.38E-01 | 1.60E-01 | 3.20E-12 | 6.64E+00 | 3.20E-11 | 9.34E-09 | EGJ | 275 |
| DED-DD | *ARHGAP15* | 1.09E-01 | 1.20E-01 | 2.20E-09 | -4.13E+00 | 3.61E-05 | 1.24E-03 | EGJ | 275 |
| Uveitis-PUD | *RASIP1* | 7.75E-02 | 1.10E-01 | 1.80E-08 | 1.69E+01 | 4.13E-64 | 2.77E-60 | EGJ | 275 |
| Uveitis-PUD | *MAMSTR* | 1.33E-01 | 1.50E-01 | 1.20E-11 | 1.61E+01 | 1.19E-58 | 3.99E-55 | EGJ | 275 |
| Uveitis-PUD | *IZUMO1* | 1.38E-01 | 1.60E-01 | 3.20E-12 | 1.27E+01 | 8.40E-37 | 1.13E-33 | EGJ | 275 |
| Uveitis-DD | *ARHGAP15* | 1.09E-01 | 1.20E-01 | 2.20E-09 | -9.20E+00 | 3.56E-20 | 4.81E-17 | EGJ | 275 |
| Myopia-IBD | *GGACT* | 6.62E-02 | 2.80E-01 | 3.90E-21 | -3.50E+00 | 4.73E-04 | 1.10E-02 | EGJ | 275 |
| Myopia-IBD | *ARMS2* | 3.26E-01 | 3.40E-01 | 5.50E-27 | -2.97E+00 | 2.96E-03 | 4.81E-02 | EGJ | 275 |
| Myopia-PUD | *GPD2* | 3.50E-02 | 4.80E-02 | 1.40E-04 | -7.04E+00 | 1.93E-12 | 4.32E-10 | EGJ | 275 |
| Myopia-PUD | *CNDP2* | 9.19E-02 | 1.10E-01 | 6.00E-09 | 5.07E+00 | 3.98E-07 | 3.30E-05 | EGJ | 275 |
| Myopia-PUD | *ACP1* | 6.85E-02 | 6.80E-02 | 6.50E-06 | -1.02E+01 | 1.44E-24 | 1.93E-21 | EGJ | 275 |
| Myopia-PUD | *ZBTB38* | 7.64E-02 | 7.60E-02 | 1.90E-06 | -1.30E+01 | 8.86E-39 | 5.95E-35 | EGJ | 275 |
| Myopia-PUD | *KCNQ5* | 1.30E-01 | 1.30E-01 | 4.20E-10 | -9.03E+00 | 1.80E-19 | 9.30E-17 | EGJ | 275 |
| Myopia-DD | *ARHGAP15* | 1.09E-01 | 1.20E-01 | 2.20E-09 | -1.06E+01 | 3.03E-26 | 1.28E-23 | EGJ | 275 |
| Myopia-DD | *BICC1* | 1.38E-01 | 1.40E-01 | 4.70E-11 | 3.95E+00 | 7.92E-05 | 1.76E-03 | EGJ | 275 |
| Myopia-DD | *COL6A1* | 1.84E-01 | 1.90E-01 | 3.40E-14 | -3.11E+00 | 1.84E-03 | 2.34E-02 | EGJ | 275 |
| Myopia-DD | *ANTXR2* | 1.97E-02 | 2.00E-02 | 1.10E-02 | 1.53E+01 | 1.12E-52 | 7.56E-49 | EGJ | 275 |
| Myopia-DD | *CHRNB1* | 1.05E-01 | 1.70E-01 | 7.20E-13 | -1.10E+01 | 6.01E-28 | 3.69E-25 | EGJ | 275 |
| Myopia-DD | *PCBP3* | 1.15E-01 | 1.40E-01 | 1.00E-10 | -8.70E+00 | 3.43E-18 | 6.62E-16 | EGJ | 275 |
| Myopia-DD | *CD55* | 1.13E-01 | 1.10E-01 | 6.60E-09 | 7.52E+00 | 5.56E-14 | 7.51E-12 | EGJ | 275 |
| Myopia-DD | *S100A10* | 9.16E-02 | 9.30E-02 | 1.50E-07 | 1.50E+01 | 1.18E-50 | 3.98E-47 | EGJ | 275 |
| Cataract-GORD | *RBM6* | 5.13E-01 | 5.10E-01 | 1.00E-44 | -1.81E+01 | 3.27E-73 | 2.20E-69 | EGJ | 275 |
| Cataract-GORD | *MST1R* | 8.25E-03 | 1.30E-02 | 3.40E-02 | 1.77E+01 | 2.48E-70 | 8.33E-67 | EGJ | 275 |
| Cataract-IBD | *NICN1* | 1.83E-01 | 1.80E-01 | 7.10E-14 | 6.51E+00 | 7.29E-11 | 8.44E-09 | EGJ | 275 |
| Cataract-IBD | *RNF123* | 8.98E-02 | 1.50E-01 | 1.40E-11 | -1.10E+01 | 3.21E-28 | 4.31E-25 | EGJ | 275 |
| Cataract-IBD | *MST1* | 4.58E-02 | 4.60E-02 | 2.10E-04 | 1.16E+01 | 4.76E-31 | 1.07E-27 | EGJ | 275 |
| Cataract-IBS | *PABPC4* | 7.83E-02 | 7.80E-02 | 1.50E-06 | -1.19E+01 | 2.03E-32 | 6.85E-29 | EGJ | 275 |
| Cataract-DD | *ARHGAP15* | 1.09E-01 | 1.20E-01 | 2.20E-09 | -6.52E+00 | 7.15E-11 | 1.38E-08 | EGJ | 275 |
| PACG-IBD | *IRF5* | 1.17E-01 | 1.20E-01 | 3.20E-09 | 1.60E+01 | 6.17E-58 | 4.14E-54 | EGJ | 275 |
| PACG-DD | *ARHGAP15* | 1.09E-01 | 1.20E-01 | 2.20E-09 | -5.23E+00 | 1.71E-07 | 1.37E-05 | EGJ | 275 |
| Keratitis-DD | *S100A10* | 9.16E-02 | 9.30E-02 | 1.50E-07 | 1.65E+01 | 2.94E-61 | 1.98E-57 | EGJ | 275 |
| AMD-GORD | *ABT1* | 1.99E-02 | 5.40E-02 | 4.90E-07 | 5.00E+00 | 5.72E-07 | 3.12E-05 | Nerve-Tibial | 438 |
| AMD-GORD | *ZNF322* | 4.20E-01 | 4.90E-01 | 7.80E-67 | -3.96E+00 | 7.57E-05 | 2.36E-03 | Nerve-Tibial | 438 |
| AMD-GORD | *HMGN4* | 1.14E-01 | 1.60E-01 | 9.10E-19 | 6.42E+00 | 1.33E-10 | 1.26E-08 | Nerve-Tibial | 438 |
| AMD-IBD | *IRF5* | 1.57E-01 | 2.00E-01 | 9.10E-23 | 1.94E+01 | 4.23E-84 | 3.97E-81 | Nerve-Tibial | 438 |
| AMD-IBD | *ULK3* | 1.40E-02 | 4.80E-02 | 2.30E-06 | -1.17E+01 | 1.87E-31 | 6.20E-29 | Nerve-Tibial | 438 |
| AMD-IBD | *SCAMP2* | 4.50E-03 | 1.90E-02 | 2.50E-03 | 1.50E+01 | 7.55E-51 | 4.26E-48 | Nerve-Tibial | 438 |
| AMD-IBD | *RPP25* | 3.61E-01 | 3.70E-01 | 1.90E-46 | -1.31E+01 | 2.16E-39 | 9.74E-37 | Nerve-Tibial | 438 |
| AMD-IBD | *MPI* | 1.42E-01 | 1.50E-01 | 1.20E-17 | 1.41E+01 | 2.01E-45 | 1.03E-42 | Nerve-Tibial | 438 |
| AMD-IBD | *RTEL1* | 3.28E-02 | 3.30E-02 | 8.10E-05 | 1.13E+01 | 9.42E-30 | 2.95E-27 | Nerve-Tibial | 438 |
| AMD-PUD | *MAPK10* | 7.25E-02 | 1.60E-01 | 1.90E-18 | 5.56E+00 | 2.64E-08 | 4.13E-06 | Nerve-Tibial | 438 |
| AMD-PUD | *NTN5* | 1.30E-01 | 2.50E-01 | 5.40E-29 | 2.04E+01 | 1.44E-92 | 1.62E-88 | Nerve-Tibial | 438 |
| AMD-PUD | *IZUMO1* | 1.39E-01 | 1.70E-01 | 4.20E-20 | 6.56E+00 | 5.37E-11 | 1.33E-08 | Nerve-Tibial | 438 |
| DR-GORD | *RBM6* | 5.81E-01 | 5.90E-01 | 5.00E-86 | -1.23E+01 | 8.63E-35 | 2.56E-32 | Nerve-Tibial | 438 |
| DR-GORD | *IGF2BP2* | 8.53E-02 | 8.50E-02 | 2.80E-10 | -5.30E+00 | 1.13E-07 | 4.52E-06 | Nerve-Tibial | 438 |
| DR-GORD | *NSF* | 1.66E-02 | 1.90E-02 | 2.10E-03 | 6.53E+00 | 6.69E-11 | 4.49E-09 | Nerve-Tibial | 438 |
| DR-GORD | *KDM2B* | 9.78E-02 | 1.10E-01 | 2.10E-12 | 9.45E+00 | 3.50E-21 | 5.81E-19 | Nerve-Tibial | 438 |
| DR-GORD | *CRTC1* | 2.34E-02 | 5.70E-02 | 2.50E-07 | -4.62E+00 | 3.80E-06 | 1.04E-04 | Nerve-Tibial | 438 |
| DR-GORD | *WNT3* | 1.55E-01 | 1.60E-01 | 3.20E-18 | -1.51E+01 | 1.90E-51 | 7.94E-49 | Nerve-Tibial | 438 |
| DR-GORD | *THADA* | 2.33E-02 | 3.30E-02 | 7.70E-05 | -1.23E+01 | 1.38E-34 | 3.99E-32 | Nerve-Tibial | 438 |
| DR-GORD | *CRHR1* | 8.04E-02 | 8.10E-02 | 7.30E-10 | -1.35E+01 | 2.98E-41 | 9.61E-39 | Nerve-Tibial | 438 |
| DR-GORD | *MAU2* | 1.19E-02 | 2.60E-02 | 4.40E-04 | -5.79E+00 | 7.06E-09 | 3.72E-07 | Nerve-Tibial | 438 |
| DR-GORD | *SNF8* | 7.96E-04 | 6.30E-03 | 5.20E-02 | 1.80E+01 | 1.77E-72 | 2.85E-69 | Nerve-Tibial | 438 |
| DR-GORD | *LTBP3* | 1.58E-01 | 1.60E-01 | 8.00E-19 | 2.36E+01 | 1.74E-123 | 4.91E-120 | Nerve-Tibial | 438 |
| DR-GORD | *TTLL6* | 2.42E-01 | 2.70E-01 | 2.30E-31 | -8.28E+00 | 1.21E-16 | 1.42E-14 | Nerve-Tibial | 438 |
| DR-GORD | *IP6K1* | 1.20E-04 | 3.40E-02 | 6.30E-05 | 1.32E+01 | 5.39E-40 | 1.64E-37 | Nerve-Tibial | 438 |
| DR-GORD | *ZNF518B* | 4.96E-02 | 5.80E-02 | 2.20E-07 | 4.56E+00 | 5.06E-06 | 1.30E-04 | Nerve-Tibial | 438 |
| DR-GORD | *UBA7* | 1.40E-01 | 1.40E-01 | 3.40E-16 | -1.15E+01 | 1.11E-30 | 2.85E-28 | Nerve-Tibial | 438 |
| DR-IBD | *THADA* | 2.33E-02 | 3.30E-02 | 7.70E-05 | -1.29E+01 | 8.13E-38 | 4.37E-35 | Nerve-Tibial | 438 |
| DR-IBD | *INPP5E* | 6.36E-02 | 8.80E-02 | 1.50E-10 | -9.98E+00 | 1.92E-23 | 5.42E-21 | Nerve-Tibial | 438 |
| DR-IBD | *GPSM1* | 1.05E-01 | 1.70E-01 | 1.80E-19 | -1.11E+01 | 8.46E-29 | 3.18E-26 | Nerve-Tibial | 438 |
| DR-IBD | *DNLZ* | 1.53E-01 | 3.70E-01 | 1.00E-45 | -1.02E+01 | 1.20E-24 | 3.87E-22 | Nerve-Tibial | 438 |
| DR-PUD | *DMPK* | 1.18E-01 | 1.30E-01 | 2.70E-15 | -8.24E+00 | 1.70E-16 | 4.26E-14 | Nerve-Tibial | 438 |
| DR-PUD | *FBXL20* | 3.99E-02 | 6.40E-02 | 4.70E-08 | 5.79E+00 | 7.10E-09 | 6.36E-07 | Nerve-Tibial | 438 |
| DR-PUD | *THADA* | 2.33E-02 | 3.30E-02 | 7.70E-05 | -5.36E+00 | 8.17E-08 | 5.59E-06 | Nerve-Tibial | 438 |
| DR-PUD | *LAMC1* | 1.45E-01 | 1.50E-01 | 5.40E-17 | 1.64E+01 | 2.37E-60 | 1.34E-56 | Nerve-Tibial | 438 |
| DR-PUD | *SIX5* | 3.35E-02 | 3.40E-02 | 6.30E-05 | 2.96E+00 | 3.10E-03 | 4.57E-02 | Nerve-Tibial | 438 |
| DR-PUD | *IZUMO1* | 1.39E-01 | 1.70E-01 | 4.20E-20 | 4.85E+00 | 1.23E-06 | 6.17E-05 | Nerve-Tibial | 438 |
| DR-IBS | *NUCB2* | 9.26E-02 | 9.30E-02 | 4.60E-11 | -4.84E+00 | 1.32E-06 | 8.31E-05 | Nerve-Tibial | 438 |
| DR-IBS | *PPP2R3A* | 4.36E-02 | 6.40E-02 | 4.40E-08 | 1.14E+01 | 7.18E-30 | 4.28E-27 | Nerve-Tibial | 438 |
| DR-IBS | *PABPC4* | 1.33E-01 | 1.30E-01 | 1.90E-15 | 2.34E+01 | 2.51E-121 | 9.49E-118 | Nerve-Tibial | 438 |
| DR-IBS | *MTMR3* | 9.21E-02 | 1.20E-01 | 4.30E-14 | 5.01E+00 | 5.31E-07 | 3.67E-05 | Nerve-Tibial | 438 |
| DR-IBS | *RND2* | 2.13E-01 | 2.20E-01 | 8.80E-26 | 4.97E+00 | 6.69E-07 | 4.57E-05 | Nerve-Tibial | 438 |
| DR-IBS | *NRBP1* | 7.41E-02 | 7.40E-02 | 4.20E-09 | -1.42E+01 | 9.67E-46 | 1.22E-42 | Nerve-Tibial | 438 |
| DR-IBS | *TRIM54* | 3.58E-02 | 3.60E-02 | 4.00E-05 | -1.53E+01 | 6.28E-53 | 8.90E-50 | Nerve-Tibial | 438 |
| DR-IBS | *GIN1* | 3.93E-02 | 4.60E-02 | 3.60E-06 | 3.23E+00 | 1.23E-03 | 3.11E-02 | Nerve-Tibial | 438 |
| DR-IBS | *PAM* | 3.77E-02 | 6.10E-02 | 8.80E-08 | 3.19E+00 | 1.40E-03 | 3.47E-02 | Nerve-Tibial | 438 |
| DR-IBS | *AP3S2* | 4.76E-01 | 4.90E-01 | 1.00E-66 | -3.91E+00 | 9.39E-05 | 3.67E-03 | Nerve-Tibial | 438 |
| DR-IBS | *VPS33B* | 7.25E-02 | 1.40E-01 | 5.50E-16 | 5.84E+00 | 5.36E-09 | 5.06E-07 | Nerve-Tibial | 438 |
| DR-IBS | *TMEM106A* | 3.67E-02 | 5.40E-02 | 5.50E-07 | -3.87E+00 | 1.08E-04 | 4.11E-03 | Nerve-Tibial | 438 |
| DR-IBS | *NCR3LG1* | 2.09E-01 | 2.10E-01 | 2.40E-24 | -1.27E+01 | 4.49E-37 | 3.39E-34 | Nerve-Tibial | 438 |
| DR-IBS | *NBR1* | 1.74E-01 | 1.80E-01 | 1.80E-20 | -5.97E+00 | 2.36E-09 | 2.33E-07 | Nerve-Tibial | 438 |
| DED-GORD | *NCAM1* | 7.35E-02 | 1.20E-01 | 1.20E-13 | -3.95E+00 | 7.69E-05 | 2.75E-03 | Nerve-Tibial | 438 |
| DED-IBD | *RHOA* | 1.77E-03 | 3.70E-03 | 1.10E-01 | -3.67E+00 | 2.40E-04 | 8.62E-03 | Nerve-Tibial | 438 |
| DED-IBD | *P4HA2* | 2.73E-01 | 2.80E-01 | 4.60E-33 | 9.84E+00 | 7.79E-23 | 6.76E-20 | Nerve-Tibial | 438 |
| DED-IBD | *IRF5* | 1.57E-01 | 2.00E-01 | 9.10E-23 | 1.16E+01 | 3.98E-31 | 8.98E-28 | Nerve-Tibial | 438 |
| DED-IBD | *AMT* | 5.20E-01 | 5.40E-01 | 2.10E-75 | 6.24E+00 | 4.49E-10 | 6.85E-08 | Nerve-Tibial | 438 |
| DED-IBD | *NICN1* | 2.41E-01 | 2.90E-01 | 8.40E-35 | 1.01E+01 | 4.42E-24 | 5.54E-21 | Nerve-Tibial | 438 |
| DED-IBD | *APEH* | 6.21E-02 | 1.10E-01 | 3.20E-13 | 1.43E+01 | 1.33E-46 | 7.50E-43 | Nerve-Tibial | 438 |
| DED-IBD | *RNF123* | 2.22E-01 | 2.20E-01 | 8.10E-26 | -9.99E+00 | 1.65E-23 | 1.69E-20 | Nerve-Tibial | 438 |
| DED-IBD | *MST1* | 9.72E-02 | 2.10E-01 | 1.70E-24 | 7.91E+00 | 2.51E-15 | 8.58E-13 | Nerve-Tibial | 438 |
| DED-IBD | *GPX1* | 4.11E-02 | 5.40E-02 | 5.30E-07 | 3.45E+00 | 5.70E-04 | 1.75E-02 | Nerve-Tibial | 438 |
| DED-IBS | *NCAM1* | 7.35E-02 | 1.20E-01 | 1.20E-13 | 4.91E+00 | 9.19E-07 | 1.27E-04 | Nerve-Tibial | 438 |
| DED-DD | *ARHGAP15* | 6.46E-02 | 6.50E-02 | 4.10E-08 | -4.30E+00 | 1.70E-05 | 7.19E-04 | Nerve-Tibial | 438 |
| Uveitis-PUD | *IZUMO1* | 1.39E-01 | 1.70E-01 | 4.20E-20 | 6.51E+00 | 7.31E-11 | 5.89E-08 | Nerve-Tibial | 438 |
| Uveitis-DD | *ARHGAP15* | 6.46E-02 | 6.50E-02 | 4.10E-08 | -9.41E+00 | 5.13E-21 | 5.81E-18 | Nerve-Tibial | 438 |
| Myopia-GORD | *SHISA6* | 2.75E-02 | 8.90E-02 | 1.30E-10 | -6.25E+00 | 3.98E-10 | 4.08E-08 | Nerve-Tibial | 438 |
| Myopia-IBD | *GGACT* | 2.83E-02 | 9.90E-02 | 9.40E-12 | -3.74E+00 | 1.87E-04 | 5.13E-03 | Nerve-Tibial | 438 |
| Myopia-PUD | *GPD2* | 1.89E-01 | 1.90E-01 | 8.20E-22 | -7.13E+00 | 1.01E-12 | 3.80E-10 | Nerve-Tibial | 438 |
| Myopia-PUD | *CNDP2* | 2.94E-01 | 3.40E-01 | 3.60E-42 | 6.16E+00 | 7.06E-10 | 1.31E-07 | Nerve-Tibial | 438 |
| Myopia-PUD | *ACP1* | 9.31E-03 | 1.80E-02 | 2.70E-03 | -9.60E+00 | 7.99E-22 | 6.44E-19 | Nerve-Tibial | 438 |
| Myopia-PUD | *ZBTB38* | 2.62E-02 | 2.90E-02 | 1.80E-04 | -1.48E+01 | 1.60E-49 | 9.03E-46 | Nerve-Tibial | 438 |
| Myopia-DD | *ARHGAP15* | 6.46E-02 | 6.50E-02 | 4.10E-08 | -9.22E+00 | 2.86E-20 | 7.90E-18 | Nerve-Tibial | 438 |
| Myopia-DD | *BICC1* | 1.96E-01 | 2.00E-01 | 3.10E-23 | 3.58E+00 | 3.49E-04 | 6.57E-03 | Nerve-Tibial | 438 |
| Myopia-DD | *COL6A1* | 2.29E-01 | 2.40E-01 | 5.20E-28 | -3.41E+00 | 6.59E-04 | 1.10E-02 | Nerve-Tibial | 438 |
| Myopia-DD | *ANTXR2* | 5.97E-02 | 1.00E-01 | 8.50E-12 | 5.78E+00 | 7.61E-09 | 4.87E-07 | Nerve-Tibial | 438 |
| Myopia-DD | *CHRNB1* | 3.04E-01 | 3.20E-01 | 1.40E-38 | -8.97E+00 | 3.08E-19 | 7.76E-17 | Nerve-Tibial | 438 |
| Myopia-DD | *ZBTB4* | 5.02E-02 | 5.00E-02 | 1.30E-06 | -2.21E+01 | 3.58E-108 | 2.03E-104 | Nerve-Tibial | 438 |
| Myopia-DD | *POLR2A* | -1.73E-03 | 9.20E-04 | 2.40E-01 | 3.48E+01 | 5.52E-265 | 6.26E-261 | Nerve-Tibial | 438 |
| Myopia-DD | *PCBP3* | 3.60E-01 | 4.00E-01 | 3.30E-50 | -6.85E+00 | 7.59E-12 | 8.27E-10 | Nerve-Tibial | 438 |
| Myopia-DD | *CD55* | 1.68E-02 | 2.30E-02 | 8.70E-04 | 6.41E+00 | 1.42E-10 | 1.25E-08 | Nerve-Tibial | 438 |
| Myopia-DD | *S100A10* | 3.74E-02 | 3.80E-02 | 2.40E-05 | 1.40E+01 | 2.16E-44 | 4.90E-41 | Nerve-Tibial | 438 |
| Cataract-GORD | *SEMA3F* | 3.77E-02 | 1.20E-01 | 7.40E-14 | -8.59E+00 | 8.47E-18 | 2.73E-15 | Nerve-Tibial | 438 |
| Cataract-GORD | *RBM6* | 5.81E-01 | 5.90E-01 | 5.00E-86 | -1.81E+01 | 5.77E-73 | 6.51E-69 | Nerve-Tibial | 438 |
| Cataract-GORD | *BTN2A1* | 7.30E-02 | 7.30E-02 | 5.50E-09 | 4.52E+00 | 6.11E-06 | 3.21E-04 | Nerve-Tibial | 438 |
| Cataract-GORD | *BTN1A1* | 1.31E-02 | 1.30E-02 | 9.50E-03 | 1.40E+01 | 1.12E-44 | 3.16E-41 | Nerve-Tibial | 438 |
| Cataract-GORD | *MST1R* | 1.09E-01 | 1.10E-01 | 7.10E-13 | 1.55E+01 | 1.90E-54 | 7.15E-51 | Nerve-Tibial | 438 |
| Cataract-IBD | *RHOA* | 1.77E-03 | 3.70E-03 | 1.10E-01 | -5.50E+00 | 3.71E-08 | 3.77E-06 | Nerve-Tibial | 438 |
| Cataract-IBD | *NICN1* | 2.41E-01 | 2.90E-01 | 8.40E-35 | 1.15E+01 | 2.17E-30 | 4.90E-27 | Nerve-Tibial | 438 |
| Cataract-IBD | *APEH* | 6.21E-02 | 1.10E-01 | 3.20E-13 | 1.20E+01 | 5.75E-33 | 1.62E-29 | Nerve-Tibial | 438 |
| Cataract-IBD | *RNF123* | 2.22E-01 | 2.20E-01 | 8.10E-26 | -9.95E+00 | 2.49E-23 | 2.01E-20 | Nerve-Tibial | 438 |
| Cataract-IBD | *MST1* | 9.72E-02 | 2.10E-01 | 1.70E-24 | 9.64E+00 | 5.59E-22 | 3.71E-19 | Nerve-Tibial | 438 |
| Cataract-IBD | *GPX1* | 4.11E-02 | 5.40E-02 | 5.30E-07 | 4.94E+00 | 7.70E-07 | 5.83E-05 | Nerve-Tibial | 438 |
| Cataract-IBS | *PABPC4* | 1.33E-01 | 1.30E-01 | 1.90E-15 | 1.28E+01 | 2.84E-37 | 8.05E-34 | Nerve-Tibial | 438 |
| Cataract-IBS | *NCAM1* | 7.35E-02 | 1.20E-01 | 1.20E-13 | 4.47E+00 | 7.80E-06 | 8.50E-04 | Nerve-Tibial | 438 |
| Cataract-DD | *ARHGAP15* | 6.46E-02 | 6.50E-02 | 4.10E-08 | -7.61E+00 | 2.82E-14 | 1.39E-11 | Nerve-Tibial | 438 |
| PACG-IBD | *IRF5* | 1.57E-01 | 2.00E-01 | 9.10E-23 | 1.53E+01 | 6.02E-53 | 6.79E-49 | Nerve-Tibial | 438 |
| PACG-DD | *ARHGAP15* | 6.46E-02 | 6.50E-02 | 4.10E-08 | -5.02E+00 | 5.07E-07 | 3.68E-05 | Nerve-Tibial | 438 |
| PACG-DD | *P2RY14* | 1.99E-02 | 2.00E-02 | 1.80E-03 | 3.04E+00 | 2.39E-03 | 4.09E-02 | Nerve-Tibial | 438 |
| Keratitis-IBD | *DLD* | 9.32E-02 | 9.70E-02 | 1.70E-11 | -6.26E+00 | 3.82E-10 | 1.11E-07 | Nerve-Tibial | 438 |
| Keratitis-DD | *S100A10* | 3.74E-02 | 3.80E-02 | 2.40E-05 | 1.84E+01 | 2.56E-75 | 1.45E-71 | Nerve-Tibial | 438 |
| AMD-GORD | *ABT1* | -1.06E-03 | -1.10E-03 | 3.60E-01 | 4.60E+00 | 4.29E-06 | 1.44E-04 | SI-TI | 141 |
| AMD-GORD | *ZNF322* | 3.51E-01 | 4.20E-01 | 1.40E-18 | -8.77E+00 | 1.85E-18 | 3.33E-16 | SI-TI | 141 |
| AMD-GORD | *HMGN4* | 1.61E-01 | 2.40E-01 | 5.40E-10 | 3.41E+00 | 6.50E-04 | 1.16E-02 | SI-TI | 141 |
| AMD-IBD | *IRF5* | 1.47E-01 | 1.80E-01 | 1.40E-07 | 1.87E+01 | 1.05E-77 | 3.99E-75 | SI-TI | 141 |
| AMD-PUD | *NTN5* | 1.28E-02 | 5.50E-02 | 3.10E-03 | 2.67E+01 | 1.51E-157 | 2.58E-154 | SI-TI | 141 |
| AMD-PUD | *FUT2* | 3.77E-01 | 3.80E-01 | 3.30E-16 | -3.26E+01 | 1.20E-233 | 4.10E-230 | SI-TI | 141 |
| AMD-PUD | *IZUMO1* | 1.90E-02 | 6.80E-02 | 1.10E-03 | 7.84E+00 | 4.56E-15 | 1.42E-12 | SI-TI | 141 |
| AMD-IBS | *BTBD16* | 3.28E-01 | 3.30E-01 | 7.30E-14 | 4.88E+00 | 1.09E-06 | 6.35E-05 | SI-TI | 141 |
| DR-GORD | *RBM6* | 3.58E-01 | 3.70E-01 | 9.50E-16 | -1.32E+01 | 1.45E-39 | 2.25E-37 | SI-TI | 141 |
| DR-GORD | *WNT3* | 4.19E-02 | 6.60E-02 | 1.20E-03 | -1.39E+01 | 1.06E-43 | 1.81E-41 | SI-TI | 141 |
| DR-GORD | *KANSL1* | 8.18E-02 | 1.10E-01 | 2.50E-05 | -1.13E+01 | 1.90E-29 | 2.32E-27 | SI-TI | 141 |
| DR-GORD | *MAP3K11* | 1.22E-01 | 1.60E-01 | 5.80E-07 | -4.59E+00 | 4.53E-06 | 9.80E-05 | SI-TI | 141 |
| DR-GORD | *UBA7* | 1.24E-01 | 1.50E-01 | 9.70E-07 | -1.06E+01 | 3.42E-26 | 3.90E-24 | SI-TI | 141 |
| DR-GORD | *ARL17B* | 1.69E-01 | 1.90E-01 | 4.70E-08 | -4.51E+00 | 6.37E-06 | 1.34E-04 | SI-TI | 141 |
| DR-IBD | *DNLZ* | 3.84E-01 | 4.50E-01 | 4.20E-20 | 3.07E+00 | 2.14E-03 | 2.70E-02 | SI-TI | 141 |
| DR-PUD | *DMPK* | 7.03E-03 | 2.50E-02 | 3.40E-02 | -8.44E+00 | 3.17E-17 | 3.87E-15 | SI-TI | 141 |
| DR-PUD | *FBXL20* | 2.43E-01 | 2.40E-01 | 3.20E-10 | 1.28E+01 | 1.98E-37 | 1.13E-34 | SI-TI | 141 |
| DR-PUD | *FUT2* | 3.77E-01 | 3.80E-01 | 3.30E-16 | -2.84E+01 | 5.34E-177 | 1.83E-173 | SI-TI | 141 |
| DR-PUD | *IZUMO1* | 1.90E-02 | 6.80E-02 | 1.10E-03 | 6.68E+00 | 2.34E-11 | 1.82E-09 | SI-TI | 141 |
| DR-IBS | *NUCB2* | 2.28E-02 | 5.80E-02 | 2.30E-03 | -3.14E+00 | 1.70E-03 | 3.46E-02 | SI-TI | 141 |
| DR-IBS | *PABPC4* | 4.23E-02 | 4.90E-02 | 5.00E-03 | 5.38E+00 | 7.45E-08 | 4.27E-06 | SI-TI | 141 |
| DR-IBS | *AP3S2* | 4.05E-01 | 4.10E-01 | 1.30E-17 | 1.85E+01 | 8.45E-77 | 1.45E-73 | SI-TI | 141 |
| DED-IBD | *IRF5* | 1.47E-01 | 1.80E-01 | 1.40E-07 | 1.38E+01 | 1.92E-43 | 6.42E-40 | SI-TI | 141 |
| DED-IBD | *AMT* | 1.60E-01 | 1.60E-01 | 5.00E-07 | 4.66E+00 | 3.17E-06 | 1.66E-04 | SI-TI | 141 |
| DED-IBD | *RNF123* | 7.81E-02 | 7.80E-02 | 4.60E-04 | -5.06E+00 | 4.27E-07 | 2.90E-05 | SI-TI | 141 |
| DED-PUD | *FUT2* | 3.77E-01 | 3.80E-01 | 3.30E-16 | -1.99E+01 | 2.72E-88 | 9.30E-85 | SI-TI | 141 |
| DED-PUD | *IZUMO1* | 1.90E-02 | 6.80E-02 | 1.10E-03 | 4.35E+00 | 1.39E-05 | 1.01E-03 | SI-TI | 141 |
| Cataract-GORD | *RBM6* | 3.58E-01 | 3.70E-01 | 9.50E-16 | -1.85E+01 | 1.17E-76 | 4.00E-73 | SI-TI | 141 |
| Cataract-GORD | *CAMKV* | 1.87E-02 | 7.00E-02 | 8.60E-04 | 9.83E+00 | 8.51E-23 | 2.24E-20 | SI-TI | 141 |
| Cataract-IBD | *RNF123* | 7.81E-02 | 7.80E-02 | 4.60E-04 | -5.41E+00 | 6.47E-08 | 5.03E-06 | SI-TI | 141 |
| Cataract-IBS | *PABPC4* | 4.23E-02 | 4.90E-02 | 5.00E-03 | 4.29E+00 | 1.77E-05 | 1.19E-03 | SI-TI | 141 |
| PACG-IBD | *IRF5* | 1.47E-01 | 1.80E-01 | 1.40E-07 | 1.64E+01 | 8.48E-61 | 2.90E-57 | SI-TI | 141 |
| AMD-GORD | *ABT1* | 3.37E-02 | 3.90E-02 | 4.80E-03 | 5.69E+00 | 1.30E-08 | 8.11E-07 | Spleen | 179 |
| AMD-GORD | *ZNF322* | 3.73E-01 | 4.60E-01 | 8.10E-26 | -8.15E+00 | 3.51E-16 | 5.75E-14 | Spleen | 179 |
| AMD-GORD | *HMGN4* | 1.75E-01 | 2.40E-01 | 2.20E-12 | 7.58E+00 | 3.49E-14 | 4.65E-12 | Spleen | 179 |
| AMD-IBD | *CSK* | 6.32E-02 | 6.30E-02 | 4.00E-04 | -2.05E+01 | 1.97E-93 | 1.18E-90 | Spleen | 179 |
| AMD-IBD | *IRF5* | 7.26E-02 | 9.70E-02 | 1.20E-05 | 1.91E+01 | 1.08E-81 | 5.79E-79 | Spleen | 179 |
| AMD-IBD | *ULK3* | 5.41E-02 | 8.50E-02 | 4.30E-05 | -1.52E+01 | 3.88E-52 | 1.22E-49 | Spleen | 179 |
| AMD-IBD | *MPI* | 1.44E-01 | 2.20E-01 | 1.30E-11 | 1.55E+01 | 3.69E-54 | 1.30E-51 | Spleen | 179 |
| AMD-IBD | *STMN3* | 4.55E-02 | 6.20E-02 | 4.80E-04 | 1.55E+01 | 4.18E-54 | 1.39E-51 | Spleen | 179 |
| AMD-IBD | *LIME1* | 1.70E-01 | 1.70E-01 | 5.80E-09 | -1.02E+01 | 1.24E-24 | 2.12E-22 | Spleen | 179 |
| AMD-PUD | *NTN5* | 1.12E-01 | 1.10E-01 | 2.70E-06 | 2.86E+01 | 4.87E-180 | 2.92E-176 | Spleen | 179 |
| AMD-PUD | *FUT1* | 1.78E-01 | 1.90E-01 | 4.90E-10 | -1.49E+01 | 4.58E-50 | 1.37E-46 | Spleen | 179 |
| AMD-PUD | *IZUMO1* | 3.43E-01 | 4.10E-01 | 5.70E-22 | 6.14E+00 | 8.34E-10 | 1.32E-07 | Spleen | 179 |
| DR-GORD | *RBM6* | 4.90E-01 | 4.90E-01 | 7.00E-28 | -1.14E+01 | 6.36E-30 | 1.09E-27 | Spleen | 179 |
| DR-GORD | *WNT3* | 1.03E-01 | 1.00E-01 | 7.40E-06 | -1.50E+01 | 7.41E-51 | 1.78E-48 | Spleen | 179 |
| DR-GORD | *UBE2Z* | 2.13E-01 | 2.20E-01 | 2.60E-11 | -1.53E+01 | 6.47E-53 | 1.62E-50 | Spleen | 179 |
| DR-GORD | *LTBP3* | 6.21E-02 | 6.20E-02 | 4.50E-04 | 1.76E+01 | 3.96E-69 | 1.40E-66 | Spleen | 179 |
| DR-GORD | *UBA7* | 2.32E-01 | 2.30E-01 | 4.20E-12 | -1.03E+01 | 7.10E-25 | 1.01E-22 | Spleen | 179 |
| DR-GORD | *MAPT* | 9.39E-02 | 1.10E-01 | 4.80E-06 | -1.77E+01 | 4.40E-70 | 1.76E-67 | Spleen | 179 |
| DR-PUD | *DMPK* | 2.66E-01 | 2.70E-01 | 9.30E-14 | -8.17E+00 | 3.03E-16 | 5.68E-14 | Spleen | 179 |
| DR-PUD | *LAMC1* | 6.56E-02 | 7.40E-02 | 1.40E-04 | 1.78E+01 | 3.40E-71 | 1.02E-67 | Spleen | 179 |
| DR-PUD | *IZUMO1* | 3.43E-01 | 4.10E-01 | 5.70E-22 | 6.94E+00 | 3.97E-12 | 4.33E-10 | Spleen | 179 |
| DR-PUD | *DMWD* | 8.12E-02 | 9.90E-02 | 1.00E-05 | -6.77E+00 | 1.33E-11 | 1.35E-09 | Spleen | 179 |
| DR-IBS | *GCKR* | 3.16E-01 | 3.20E-01 | 1.50E-16 | 6.23E+00 | 4.64E-10 | 7.98E-08 | Spleen | 179 |
| DR-IBS | *KRTCAP3* | 1.82E-01 | 1.90E-01 | 8.70E-10 | -7.78E+00 | 7.35E-15 | 1.92E-12 | Spleen | 179 |
| DR-IBD | *INPP5E* | 1.32E-01 | 1.30E-01 | 3.40E-07 | -3.51E+00 | 4.41E-04 | 7.62E-03 | Spleen | 179 |
| DR-IBD | *SNAPC4* | -4.92E-03 | 7.50E-02 | 1.20E-04 | 3.76E+00 | 1.68E-04 | 3.32E-03 | Spleen | 179 |
| DR-IBD | *PEAK1* | 8.73E-02 | 1.20E-01 | 1.30E-06 | 4.12E+00 | 3.86E-05 | 9.24E-04 | Spleen | 179 |
| DR-IBD | *DNLZ* | 1.07E-01 | 2.10E-01 | 1.10E-10 | -6.22E+00 | 5.11E-10 | 3.52E-08 | Spleen | 179 |
| DED-GORD | *BTN2A1* | 8.35E-03 | 2.30E-02 | 2.50E-02 | -1.51E+01 | 1.13E-51 | 6.77E-48 | Spleen | 179 |
| DED-IBD | *P4HA2* | 8.62E-02 | 1.20E-01 | 1.90E-06 | 4.97E+00 | 6.57E-07 | 4.38E-05 | Spleen | 179 |
| DED-IBD | *IRF5* | 7.26E-02 | 9.70E-02 | 1.20E-05 | 8.71E+00 | 3.13E-18 | 8.53E-16 | Spleen | 179 |
| DED-IBD | *AMT* | 3.79E-01 | 4.00E-01 | 1.80E-21 | 3.72E+00 | 2.01E-04 | 5.91E-03 | Spleen | 179 |
| DED-IBD | *TCTA* | 1.49E-02 | 3.50E-02 | 6.90E-03 | 1.54E+01 | 1.55E-53 | 4.65E-50 | Spleen | 179 |
| DED-IBD | *APEH* | 7.36E-02 | 8.30E-02 | 5.50E-05 | 9.08E+00 | 1.06E-19 | 3.18E-17 | Spleen | 179 |
| DED-IBD | *RNF123* | 6.82E-02 | 1.00E-01 | 6.40E-06 | -1.00E+01 | 1.12E-23 | 6.10E-21 | Spleen | 179 |
| DED-IBD | *OTUD3* | -9.75E-04 | 8.50E-02 | 4.30E-05 | 5.94E+00 | 2.81E-09 | 3.18E-07 | Spleen | 179 |
| DED-PUD | *IZUMO1* | 3.43E-01 | 4.10E-01 | 5.70E-22 | 3.22E+00 | 1.29E-03 | 3.75E-02 | Spleen | 179 |
| DED-PUD | *IZUMO1* | 3.43E-01 | 4.10E-01 | 5.70E-22 | 7.75E+00 | 8.97E-15 | 4.89E-12 | Spleen | 179 |
| DED-DD | *ARHGAP15* | 1.62E-02 | 3.70E-02 | 5.70E-03 | -5.17E+00 | 2.34E-07 | 3.15E-05 | Spleen | 179 |
| Myopia-PUD | *CNDP2* | 2.65E-01 | 2.60E-01 | 1.00E-13 | -1.76E+01 | 2.54E-69 | 1.52E-65 | Spleen | 179 |
| Myopia-PUD | *ZBTB38* | 4.21E-02 | 6.90E-02 | 2.20E-04 | -1.12E+01 | 2.78E-29 | 3.33E-26 | Spleen | 179 |
| Myopia-DD | *ARHGAP15* | 1.62E-02 | 3.70E-02 | 5.70E-03 | -4.50E+00 | 6.88E-06 | 2.21E-04 | Spleen | 179 |
| Myopia-DD | *CACNB2* | -3.74E-03 | -1.80E-03 | 4.10E-01 | -4.25E+00 | 2.14E-05 | 5.89E-04 | Spleen | 179 |
| Myopia-DD | *CHRNB1* | 5.47E-02 | 5.50E-02 | 9.50E-04 | -1.19E+01 | 1.71E-32 | 1.47E-29 | Spleen | 179 |
| Myopia-DD | *CD55* | 1.23E-01 | 1.20E-01 | 8.80E-07 | -1.16E+01 | 6.23E-31 | 4.70E-28 | Spleen | 179 |
| Myopia-DD | *S100A10* | 3.68E-01 | 3.70E-01 | 1.40E-19 | -1.14E+01 | 5.67E-30 | 3.80E-27 | Spleen | 179 |
| Cataract-GORD | *RBM6* | 4.90E-01 | 4.90E-01 | 7.00E-28 | -1.84E+01 | 1.78E-75 | 1.07E-71 | Spleen | 179 |
| Cataract-GORD | *BTN2A1* | 8.35E-03 | 2.30E-02 | 2.50E-02 | -9.60E+00 | 7.98E-22 | 2.28E-19 | Spleen | 179 |
| Cataract-IBD | *TCTA* | 1.49E-02 | 3.50E-02 | 6.90E-03 | 1.70E+01 | 9.21E-65 | 5.52E-61 | Spleen | 179 |
| Cataract-IBD | *APEH* | 7.36E-02 | 8.30E-02 | 5.50E-05 | 1.28E+01 | 1.23E-37 | 2.46E-34 | Spleen | 179 |
| Cataract-IBD | *RNF123* | 6.82E-02 | 1.00E-01 | 6.40E-06 | -1.12E+01 | 2.72E-29 | 1.81E-26 | Spleen | 179 |
| PACG-IBD | *IRF5* | 7.26E-02 | 9.70E-02 | 1.20E-05 | 1.30E+01 | 1.77E-38 | 1.06E-34 | Spleen | 179 |
| PACG-IBD | *OTUD3* | -9.75E-04 | 8.50E-02 | 4.30E-05 | 4.39E+00 | 1.14E-05 | 5.42E-04 | Spleen | 179 |
| Keratitis-DD | *ARHGAP15* | 1.62E-02 | 3.70E-02 | 5.70E-03 | -3.16E+00 | 1.57E-03 | 4.00E-02 | Spleen | 179 |
| Keratitis-DD | *P2RY12* | 1.76E-01 | 2.40E-01 | 2.90E-12 | -3.39E+00 | 6.93E-04 | 2.12E-02 | Spleen | 179 |
| Keratitis-DD | *S100A10* | 3.68E-01 | 3.70E-01 | 1.40E-19 | -8.43E+00 | 3.57E-17 | 6.66E-14 | Spleen | 179 |
| AMD-GORD | *ZNF322* | 2.08E-01 | 2.60E-01 | 1.60E-18 | -1.25E+01 | 1.17E-35 | 1.02E-32 | Stomach | 260 |
| AMD-IBD | *IRF5* | 3.24E-01 | 3.20E-01 | 6.30E-24 | 1.73E+01 | 7.74E-67 | 4.50E-64 | Stomach | 260 |
| AMD-IBD | *ULK3* | 1.14E-01 | 1.20E-01 | 6.30E-09 | -1.43E+01 | 2.51E-46 | 1.01E-43 | Stomach | 260 |
| AMD-IBD | *RPP25* | 1.77E-01 | 1.90E-01 | 4.70E-14 | -1.01E+01 | 4.96E-24 | 1.08E-21 | Stomach | 260 |
| AMD-IBD | *MPI* | 4.29E-02 | 6.20E-02 | 2.80E-05 | 1.50E+01 | 1.32E-50 | 5.75E-48 | Stomach | 260 |
| AMD-IBD | *STMN3* | 2.03E-03 | 1.90E-02 | 1.60E-02 | -7.31E+00 | 2.67E-13 | 3.40E-11 | Stomach | 260 |
| AMD-PUD | *RASIP1* | 1.97E-01 | 3.50E-01 | 4.10E-26 | 1.01E+01 | 4.00E-24 | 3.49E-21 | Stomach | 260 |
| AMD-PUD | *MAMSTR* | 7.90E-04 | 2.60E-02 | 4.90E-03 | -1.60E+01 | 1.20E-57 | 3.14E-54 | Stomach | 260 |
| AMD-PUD | *FUT2* | 2.67E-01 | 2.90E-01 | 1.80E-21 | -3.25E+01 | 3.92E-231 | 2.05E-227 | Stomach | 260 |
| AMD-PUD | *IZUMO1* | 3.62E-01 | 4.10E-01 | 5.30E-32 | 3.07E+00 | 2.15E-03 | 4.26E-02 | Stomach | 260 |
| AMD-IBS | *BTBD16* | 3.83E-01 | 4.10E-01 | 3.20E-31 | -3.61E+00 | 3.06E-04 | 1.34E-02 | Stomach | 260 |
| DR-GORD | *RBM6* | 4.62E-01 | 4.80E-01 | 1.30E-38 | -1.24E+01 | 3.16E-35 | 6.61E-33 | Stomach | 260 |
| DR-GORD | *ANAPC5* | 1.23E-02 | 7.20E-02 | 6.90E-06 | 3.18E+00 | 1.46E-03 | 1.67E-02 | Stomach | 260 |
| DR-GORD | *KDM2B* | 9.24E-02 | 9.20E-02 | 3.50E-07 | 7.48E+00 | 7.39E-14 | 5.37E-12 | Stomach | 260 |
| DR-GORD | *WNT3* | 7.78E-02 | 9.90E-02 | 1.20E-07 | -1.45E+01 | 1.27E-47 | 3.02E-45 | Stomach | 260 |
| DR-GORD | *THADA* | 3.71E-02 | 4.30E-02 | 4.40E-04 | -1.12E+01 | 3.88E-29 | 6.34E-27 | Stomach | 260 |
| DR-GORD | *SNF8* | 1.16E-01 | 1.30E-01 | 7.20E-10 | 1.54E+01 | 8.33E-54 | 2.42E-51 | Stomach | 260 |
| DR-GORD | *ARHGAP27* | 2.43E-02 | 2.40E-02 | 6.80E-03 | 1.79E+01 | 1.90E-71 | 1.10E-68 | Stomach | 260 |
| DR-GORD | *EHBP1L1* | 6.74E-02 | 6.70E-02 | 1.30E-05 | 1.18E+01 | 5.40E-32 | 1.01E-29 | Stomach | 260 |
| DR-GORD | *UBA7* | 1.99E-01 | 2.10E-01 | 7.70E-15 | -1.02E+01 | 1.43E-24 | 2.02E-22 | Stomach | 260 |
| DR-IBD | *UBE3C* | 1.29E-04 | 3.10E-03 | 1.80E-01 | 1.35E+01 | 2.82E-41 | 8.68E-39 | Stomach | 260 |
| DR-IBD | *THADA* | 3.71E-02 | 4.30E-02 | 4.40E-04 | -1.15E+01 | 7.47E-31 | 1.63E-28 | Stomach | 260 |
| DR-IBD | *INPP5E* | 1.80E-02 | 3.70E-02 | 1.10E-03 | -5.44E+00 | 5.46E-08 | 2.23E-06 | Stomach | 260 |
| DR-IBD | *PEAK1* | 1.31E-02 | 1.30E-02 | 3.60E-02 | -4.77E+00 | 1.84E-06 | 6.21E-05 | Stomach | 260 |
| DR-IBD | *DNLZ* | 2.66E-01 | 3.10E-01 | 3.90E-23 | -8.25E+00 | 1.64E-16 | 2.04E-14 | Stomach | 260 |
| DR-PUD | *UBE3C* | 1.29E-04 | 3.10E-03 | 1.80E-01 | -1.82E+01 | 4.52E-74 | 7.88E-71 | Stomach | 260 |
| DR-PUD | *RASIP1* | 1.97E-01 | 3.50E-01 | 4.10E-26 | 8.07E+00 | 6.83E-16 | 1.16E-13 | Stomach | 260 |
| DR-PUD | *THADA* | 3.71E-02 | 4.30E-02 | 4.40E-04 | -4.08E+00 | 4.43E-05 | 1.32E-03 | Stomach | 260 |
| DR-PUD | *SGCD* | 1.54E-01 | 1.60E-01 | 8.60E-12 | 7.60E+00 | 3.04E-14 | 4.18E-12 | Stomach | 260 |
| DR-PUD | *MAMSTR* | 7.90E-04 | 2.60E-02 | 4.90E-03 | -1.30E+01 | 1.70E-38 | 1.78E-35 | Stomach | 260 |
| DR-PUD | *FUT2* | 2.67E-01 | 2.90E-01 | 1.80E-21 | -2.58E+01 | 3.18E-146 | 1.66E-142 | Stomach | 260 |
| DR-PUD | *SIX5* | 4.64E-02 | 4.60E-02 | 2.70E-04 | 4.76E+00 | 1.92E-06 | 8.51E-05 | Stomach | 260 |
| DR-PUD | *IZUMO1* | 3.62E-01 | 4.10E-01 | 5.30E-32 | 3.85E+00 | 1.19E-04 | 3.05E-03 | Stomach | 260 |
| DR-IBS | *PPP2R3A* | 5.97E-02 | 7.20E-02 | 6.50E-06 | -8.70E+00 | 3.18E-18 | 6.43E-16 | Stomach | 260 |
| DR-IBS | *MTMR3* | 3.08E-02 | 3.10E-02 | 2.60E-03 | 5.22E+00 | 1.80E-07 | 1.00E-05 | Stomach | 260 |
| DR-IBS | *PAM* | 1.72E-02 | 1.70E-02 | 1.90E-02 | 1.29E+01 | 2.44E-38 | 1.43E-35 | Stomach | 260 |
| DR-IBS | *AP3S2* | 5.13E-01 | 5.10E-01 | 1.90E-42 | 2.16E+01 | 2.92E-103 | 7.68E-100 | Stomach | 260 |
| DR-IBS | *KRTCAP3* | 2.50E-02 | 4.80E-02 | 2.10E-04 | 3.40E+00 | 6.68E-04 | 1.58E-02 | Stomach | 260 |
| DR-IBS | *BMP8A* | 2.46E-02 | 3.00E-02 | 3.20E-03 | -5.08E+00 | 3.74E-07 | 1.93E-05 | Stomach | 260 |
| DR-IBS | *VPS33B* | -3.48E-03 | -5.80E-05 | 3.20E-01 | -3.71E+00 | 2.10E-04 | 5.72E-03 | Stomach | 260 |
| DED-IBD | *IRF5* | 3.24E-01 | 3.20E-01 | 6.30E-24 | 1.30E+01 | 2.21E-38 | 3.85E-35 | Stomach | 260 |
| DED-IBD | *AMT* | 2.62E-01 | 2.60E-01 | 5.70E-19 | 4.06E+00 | 4.91E-05 | 2.01E-03 | Stomach | 260 |
| DED-IBD | *NICN1* | 6.61E-02 | 7.50E-02 | 4.20E-06 | 4.34E+00 | 1.45E-05 | 7.95E-04 | Stomach | 260 |
| DED-IBD | *MST1* | 8.61E-02 | 8.60E-02 | 8.80E-07 | 6.94E+00 | 3.89E-12 | 7.82E-10 | Stomach | 260 |
| DED-PUD | *RASIP1* | 1.97E-01 | 3.50E-01 | 4.10E-26 | 4.34E+00 | 1.45E-05 | 9.60E-04 | Stomach | 260 |
| DED-PUD | *MAMSTR* | 7.90E-04 | 2.60E-02 | 4.90E-03 | -9.60E+00 | 7.78E-22 | 1.36E-18 | Stomach | 260 |
| DED-PUD | *FUT2* | 2.67E-01 | 2.90E-01 | 1.80E-21 | -2.03E+01 | 1.53E-91 | 8.00E-88 | Stomach | 260 |
| DED-DD | *SKIDA1* | -3.88E-03 | 3.30E-02 | 2.00E-03 | 1.32E+01 | 1.30E-39 | 6.83E-36 | Stomach | 260 |
| Uveitis-PUD | *RASIP1* | 1.97E-01 | 3.50E-01 | 4.10E-26 | 8.49E+00 | 2.13E-17 | 1.59E-14 | Stomach | 260 |
| Uveitis-PUD | *MAMSTR* | 7.90E-04 | 2.60E-02 | 4.90E-03 | -1.31E+01 | 2.11E-39 | 3.68E-36 | Stomach | 260 |
| Uveitis-PUD | *FUT2* | 2.67E-01 | 2.90E-01 | 1.80E-21 | -1.62E+01 | 2.94E-59 | 1.54E-55 | Stomach | 260 |
| Uveitis-PUD | *IZUMO1* | 3.62E-01 | 4.10E-01 | 5.30E-32 | 4.87E+00 | 1.14E-06 | 1.99E-04 | Stomach | 260 |
| Myopia-IBD | *GGACT* | 1.76E-01 | 1.80E-01 | 3.00E-13 | -4.86E+00 | 1.16E-06 | 6.00E-05 | Stomach | 260 |
| Myopia-PUD | *FUT2* | 2.67E-01 | 2.90E-01 | 1.80E-21 | -8.70E+00 | 3.43E-18 | 3.59E-15 | Stomach | 260 |
| Myopia-DD | *ARHGAP15* | 1.16E-02 | 2.80E-02 | 3.70E-03 | -3.83E+00 | 1.27E-04 | 2.61E-03 | Stomach | 260 |
| Myopia-DD | *CHRNB1* | 1.30E-01 | 2.20E-01 | 4.60E-16 | -2.21E+01 | 7.25E-108 | 3.81E-104 | Stomach | 260 |
| Myopia-DD | *PCBP3* | 1.36E-01 | 1.60E-01 | 1.70E-11 | -7.07E+00 | 1.56E-12 | 1.91E-10 | Stomach | 260 |
| Myopia-DD | *S100A10* | 8.39E-02 | 8.40E-02 | 1.20E-06 | -1.14E+01 | 5.67E-30 | 4.97E-27 | Stomach | 260 |
| Cataract-GORD | *RBM6* | 4.62E-01 | 4.80E-01 | 1.30E-38 | -1.80E+01 | 8.90E-73 | 4.65E-69 | Stomach | 260 |
| Cataract-GORD | *CAMKV* | 2.44E-01 | 2.40E-01 | 1.30E-17 | 1.71E+01 | 1.03E-65 | 2.69E-62 | Stomach | 260 |
| Cataract-IBD | *NICN1* | 6.61E-02 | 7.50E-02 | 4.20E-06 | 7.28E+00 | 3.44E-13 | 6.00E-11 | Stomach | 260 |
| Cataract-IBD | *MST1* | 8.61E-02 | 8.60E-02 | 8.80E-07 | 1.05E+01 | 6.51E-26 | 4.26E-23 | Stomach | 260 |
| Cataract-IBD | *GPX1* | 2.66E-02 | 2.70E-02 | 4.80E-03 | 3.10E+00 | 1.91E-03 | 4.06E-02 | Stomach | 260 |
| PACG-IBD | *IRF5* | 3.24E-01 | 3.20E-01 | 6.30E-24 | 1.33E+01 | 4.40E-40 | 2.30E-36 | Stomach | 260 |
| PACG-DD | *ENTPD7* | 6.68E-02 | 8.30E-02 | 1.40E-06 | 1.88E+01 | 1.25E-78 | 6.57E-75 | Stomach | 260 |
| Keratitis-DD | *S100A10* | 8.39E-02 | 8.40E-02 | 1.20E-06 | -8.43E+00 | 3.57E-17 | 3.75E-14 | Stomach | 260 |
| Keratitis-DD | *ENTPD7* | 6.68E-02 | 8.30E-02 | 1.40E-06 | 1.82E+01 | 3.22E-74 | 1.69E-70 | Stomach | 260 |

Note: Esophagus-Gastroesophageal Junction: EGJ; Small Intestine-Terminal Ileum: SI-TI

**Table S14.** Significant PWAS results based on candidate pleiotropic genes

| **Trait-pairs** | **GENE** | **EQTL.R2** | **MODELCV.R2** | **MODELCV.PV** | **TWAS.Z** | **TWAS.P** | **TWAS.FDR** |
| --- | --- | --- | --- | --- | --- | --- | --- |
| AMD-GORD | *APOE* | 8.81E-02 | 1.00E-01 | 2.10E-79 | -5.90E+00 | 3.59E-09 | 2.68E-07 |
| AMD-GORD | *APOE* | 1.48E-02 | 3.10E-02 | 5.80E-25 | -1.35E+01 | 1.84E-41 | 9.26E-39 |
| AMD-IBD | *CSK* | 2.51E-03 | 3.20E-03 | 6.80E-04 | -2.10E+01 | 8.09E-98 | 4.07E-95 |
| AMD-IBD | *TNFRSF6B* | 1.28E-02 | 1.60E-02 | 3.40E-13 | -7.78E+00 | 7.26E-15 | 7.30E-13 |
| Cataract-IBD | *IL23R* | 2.94E-02 | 5.60E-02 | 1.40E-43 | 8.24E+00 | 1.69E-16 | 4.26E-14 |
| Cataract-IBD | *FCGR2A* | 7.89E-01 | 7.90E-01 | 0.00E+00 | -1.67E+01 | 2.37E-62 | 2.39E-59 |
| Cataract-IBD | *DLD* | 4.36E-03 | 4.70E-03 | 4.50E-05 | -7.77E+00 | 8.02E-15 | 1.62E-12 |
| DR-GORD | *NSF* | 2.29E-03 | 2.30E-03 | 3.40E-03 | -1.41E+01 | 2.27E-45 | 3.82E-43 |
| DR-GORD | *APOE* | 1.48E-02 | 3.10E-02 | 5.80E-25 | -1.56E+01 | 1.06E-54 | 2.14E-52 |
| DR-IBS | *GCKR* | 4.28E-03 | 4.30E-03 | 9.90E-05 | -2.32E+01 | 2.78E-119 | 2.82E-116 |
| Keratitis-IBD | *IL23R* | 2.94E-02 | 5.60E-02 | 1.40E-43 | 1.29E+01 | 5.62E-38 | 5.67E-35 |
| Keratitis-IBD | *DLD* | 4.36E-03 | 4.70E-03 | 4.50E-05 | -1.21E+01 | 1.59E-33 | 8.02E-31 |
| Myopia-DD | *CD55* | 1.27E-01 | 1.50E-01 | 4.30E-116 | 2.02E+01 | 2.13E-90 | 2.16E-87 |
| Myopia-DD | *COL6A1* | 4.76E-02 | 5.30E-02 | 4.30E-41 | -1.39E+01 | 1.24E-43 | 4.20E-41 |
| Myopia-GORD | *APOE* | 8.81E-02 | 1.00E-01 | 2.10E-79 | -3.12E+00 | 1.82E-03 | 3.11E-02 |
| Myopia-GORD | *APOE* | 1.48E-02 | 3.10E-02 | 5.80E-25 | -1.21E+01 | 7.66E-34 | 7.73E-31 |
| Myopia-PUD | *ACP1* | 6.80E-01 | 6.80E-01 | 0.00E+00 | 1.03E+01 | 9.96E-25 | 5.02E-22 |

**Table S15.** Data field of Modifiable exposure for G×E analysis in UK Biobank

| **Data field** | **Catgory** | **Modifiable exposure** | **Description** |
| --- | --- | --- | --- |
| 24003 | Air Pollutants | Nitrogen dioxide air pollution | Continuous variable |
| 24004 | Air Pollutants | Nitrogen oxides air pollution | Continuous variable |
| 24005 | Air Pollutants | Particulate matter air pollution (pm10) | Continuous variable |
| 24006 | Air Pollutants | Particulate matter air pollution (pm2.5) | Continuous variable |
| 21067 | Antibiotic exposure | Long-term/recurrent antibiotics as child or teenager | No VS Yes |
| 6154 | Aspirin exposure | Medication for pain relief, constipation, heartburn: Aspirin | No VS Yes |
| 1329 | Diet | Oily fish intake | No VS ≤1 time/week VS >1 time/week |
| 1369 | Diet | Beef intake | No VS ≤1 time/week VS >1 time/week |
| 1379 | Diet | Lamb/mutton intake | No VS ≤1 time/week VS >1 time/week |
| 1389 | Diet | Pork intake | No VS ≤1 time/week VS >1 time/week |
| 1349 | Diet | Processed meat intake | No VS ≤1 time/week VS >1 time/week |
| 6179 | Diet | Mineral and other dietary supplements: Calcium | No VS Yes |
| 1408 | Diet | Cheese intake | No VS ≤1 time/week VS >1 time/week |
| 1309 | Diet | Fresh fruit intake | Continuous variable |
| 1319 | Diet | Dried fruit intake | Continuous variable |
| 1289 | Diet | Cooked vegetable intake | Continuous variable |
| 1299 | Diet | Salad / raw vegetable intake | Continuous variable |
| 1558 | Lifestyles | Alcohol intake frequency | No VS ≤1 time/week VS >1 time/week |
| 20117 | Lifestyles | Alcohol drinker status | Never VS Previous VS Current |
| 20116 | Lifestyles | Smoking status | Never VS Previous VS Current |
| 864 | Lifestyles | Number of days/week walked 10+ minutes | Continuous variable |
| 884 | Lifestyles | Number of days/week of moderate physical activity 10+ minutes | Continuous variable |
| 904 | Lifestyles | Number of days/week of vigorous physical activity 10+ minutes | Continuous variable |
| 1269 | Lifestyles | Exposure to tobacco smoke at home | Continuous variable |
| 1279 | Lifestyles | Exposure to tobacco smoke outside home | Continuous variable |
| 1239 | Lifestyles | Current tobacco smoking | No or Occasionally VS Most or all days |
| 21001 | Lifestyles | Body mass index (BMI, Physical measures) | Continuous variable |
| 23104 | Lifestyles | Body mass index (BMI, Body composition) | Continuous variable |
| 1920 | Mental health | Mood swings | No VS Yes |
| 1930 | Mental health | Miserableness | No VS Yes |
| 1940 | Mental health | Irritability | No VS Yes |
| 1950 | Mental health | Sensitivity / hurt feelings | No VS Yes |
| 1960 | Mental health | Fed-up feelings | No VS Yes |
| 1970 | Mental health | Nervous feelings | No VS Yes |
| 1980 | Mental health | Worrier / anxious feelings | No VS Yes |
| 1990 | Mental health | Tense / 'highly strung' | No VS Yes |
| 2000 | Mental health | Worry too long after embarrassment | No VS Yes |
| 2010 | Mental health | Suffer from 'nerves' | No VS Yes |
| 2020 | Mental health | Loneliness, isolation | No VS Yes |
| 2030 | Mental health | Guilty feelings | No VS Yes |
| 2040 | Mental health | Risk taking | No VS Yes |
| 2050 | Mental health | Frequency of depressed mood in last 2 weeks | No VS Several day VS Every day |
| 2060 | Mental health | Frequency of unenthusiasm / disinterest in last 2 weeks | No VS Several day VS Every day |
| 2070 | Mental health | Frequency of tenseness / restlessness in last 2 weeks | No VS Several day VS Every day |
| 2080 | Mental health | Frequency of tiredness / lethargy in last 2 weeks | No VS Several day VS Every day |
| 2090 | Mental health | Seen doctor (GP) for nerves, anxiety, tension or depression | No VS Yes |
| 2100 | Mental health | Seen a psychiatrist for nerves, anxiety, tension or depression | No VS Yes |
| 4526 | Mental health | Happiness | No VS Yes |
| 4537 | Mental health | Work/job satisfaction | No VS Yes |
| 4548 | Mental health | Health satisfaction | No VS Yes |
| 4559 | Mental health | Family relationship satisfaction | No VS Yes |
| 4570 | Mental health | Friendships satisfaction | No VS Yes |
| 4581 | Mental health | Financial situation satisfaction | No VS Yes |
| 4598 | Mental health | Ever depressed for a whole week | No VS Yes |
| 4631 | Mental health | Ever unenthusiastic/disinterested for a whole week | No VS Yes |
| 4642 | Mental health | Ever manic/hyper for 2 days | No VS Yes |
| 4653 | Mental health | Ever highly irritable/argumentative for 2 days | No VS Yes |
| 5663 | Mental health | Length of longest manic/irritable episode | ＜1w VS ≥1w |
| 5674 | Mental health | Severity of manic/irritable episodes | No VS Yes |
| 4620 | Mental health | Number of depression episodes | Continuous variable |
| 5386 | Mental health | Number of unenthusiastic/disinterested episodes | Continuous variable |
| 24015 | Traffic-related air pollution | Sum of road length of major roads within 100m | Continuous variable |
| 24009 | Traffic-related air pollution | Traffic intensity on the nearest road | Continuous variable |
| 24013 | Traffic-related air pollution | Total traffic load on major roads | Continuous variable |

**Table S16.** Characteristics for G×E analysis in UK Biobank

| **Phenotype** | **ICD-10** | **Characteristics** | N**_cases_** | N**_controls_** | *P* |
| --- | --- | --- | --- | --- | --- |
| GORD | K21 |  | 53588 | 341027 |  |
|  |  | Age | 58.76 ± 7.43 | 57.01 ± 8.01 | ＜0.01 |
|  |  | Townsend deprivation index | -1.25 ± 3.07 | -1.52 ± 2.95 | ＜0.01 |
|  |  | Sex |  |  | ＜0.01 |
|  |  | Female | 29884 (55.8%) | 185531 (54.4%) | ＜0.01 |
|  |  | Male | 23704 (44.2%) | 155496 (45.6%) | ＜0.01 |
|  |  | Education |  |  | ＜0.01 |
|  |  | College or University | 40889 (76.3%) | 230957 (67.7%) | ＜0.01 |
|  |  | Others | 11628 (21.7%) | 104654 (30.7%) | ＜0.01 |
|  |  | NA | 1071 (2.0%) | 5416 (1.6%) | ＜0.01 |
| IBD | K50, K51 |  | 7067 | 387548 | ＜0.01 |
|  |  | Age | 57.52 ± 7.93 | 57.24 ± 7.96 | ＜0.01 |
|  |  | Townsend deprivation index | -1.22 ± 3.09 | -1.49 ± 2.97 | ＜0.01 |
|  |  | Sex |  |  | ＜0.01 |
|  |  | Female | 3617 (51.2%) | 211798 (54.7%) | ＜0.01 |
|  |  | Male | 3450 (48.8%) | 175750 (45.3%) | ＜0.01 |
|  |  | Education |  |  | ＜0.01 |
|  |  | College or University | 5222 (73.9%) | 266624 (68.8%) | ＜0.01 |
|  |  | Others | 1725 (24.4%) | 114557 (29.6%) | ＜0.01 |
|  |  | NA | 120 (1.7%) | 6367 (1.6%) | ＜0.01 |
| PUD | K25, K26, K27, K28 |  | 12984 | 381631 | ＜0.01 |
|  |  | Age | 59.79 ± 7.12 | 57.16 ± 7.97 | ＜0.01 |
|  |  | Townsend deprivation index | -0.82 ± 3.27 | -1.51 ± 2.96 | ＜0.01 |
|  |  | Sex |  |  | ＜0.01 |
|  |  | Female | 5840 (45.0%) | 209575 (54.9%) | ＜0.01 |
|  |  | Male | 7144 (55.0%) | 172056 (45.1%) | ＜0.01 |
|  |  | Education |  |  | ＜0.01 |
|  |  | College or University | 10197 (78.5%) | 261649 (68.6%) | ＜0.01 |
|  |  | Others | 2547 (19.6%) | 113735 (29.8%) | ＜0.01 |
|  |  | NA | 240 (1.8%) | 6247 (1.6%) | ＜0.01 |
| IBS | K58 |  | 12796 | 381819 | ＜0.01 |
|  |  | Age | 57.13 ± 7.82 | 57.25 ± 7.96 | ＜0.01 |
|  |  | Townsend deprivation index | -1.17 ± 3.11 | -1.50 ± 2.97 | ＜0.01 |
|  |  | Sex |  |  | ＜0.01 |
|  |  | Female | 9542 (74.6%) | 205873 (53.9%) | ＜0.01 |
|  |  | Male | 3254 (25.4%) | 175946 (46.1%) | ＜0.01 |
|  |  | Education |  |  | ＜0.01 |
|  |  | College or University | 9469 (74.0%) | 262377 (68.7%) | ＜0.01 |
|  |  | Others | 3028 (23.7%) | 113254 (29.7%) | ＜0.01 |
|  |  | NA | 299 (2.3%) | 6188 (1.6%) | ＜0.01 |
| DD | K57 |  | 63475 | 331140 | ＜0.01 |
|  |  | Age | 59.86 ± 6.96 | 56.75 ± 8.04 | ＜0.01 |
|  |  | Townsend deprivation index | -1.40 ± 3.03 | -1.50 ± 2.96 | ＜0.01 |
|  |  | Sex |  |  | ＜0.01 |
|  |  | Female | 33807 (53.3%) | 181608 (54.8%) | ＜0.01 |
|  |  | Male | 29668 (46.7%) | 149532 (45.2%) | ＜0.01 |
|  |  | Education |  |  | ＜0.01 |
|  |  | College or University | 15603 (24.6%) | 100679 (30.4%) | ＜0.01 |
|  |  | Others | 46691 (73.6%) | 225155 (68.0%) | ＜0.01 |
|  |  | NA | 1181 (1.9%) | 5306 (1.6%) | ＜0.01 |
| AMD | H353 |  | 9928 | 384687 | ＜0.01 |
|  |  | Age | 62.96 ± 5.30 | 57.10 ± 7.96 | ＜0.01 |
|  |  | Townsend deprivation index | -1.50 ± 2.96 | -1.49 ± 2.97 | 0.71 |
|  |  | Sex |  |  |  |
|  |  | Female | 6076 (61.2%) | 209339 (54.4%) | ＜0.01 |
|  |  | Male | 3852 (38.8%) | 175348 (45.6%) | ＜0.01 |
|  |  | Education |  |  | ＜0.01 |
|  |  | College or University | 7368 (74.2%) | 264478 (68.8%) | ＜0.01 |
|  |  | Others | 2362 (23.8%) | 113920 (29.6%) | ＜0.01 |
|  |  | NA | 198 (2.0%) | 6289 (1.6%) | ＜0.01 |
| DR | H360 |  | 3723 | 390892 | ＜0.01 |
|  |  | Age | 60.55 ± 6.81 | 57.22 ± 7.96 | ＜0.01 |
|  |  | Townsend deprivation index | -0.60 ± 3.36 | -1.49 ± 2.97 | ＜0.01 |
|  |  | Sex |  |  | ＜0.01 |
|  |  | Female | 1392 (37.4%) | 214023 (54.8%) | ＜0.01 |
|  |  | Male | 2331 (62.6%) | 176869 (45.2%) | ＜0.01 |
|  |  | Education |  |  | ＜0.01 |
|  |  | College or University | 2908 (78.1%) | 268938 (68.8%) | ＜0.01 |
|  |  | Others | 732 (19.7%) | 115550 (29.6%) | ＜0.01 |
|  |  | NA | 83 (2.2%) | 6404 (1.6%) | ＜0.01 |
| DED | M350 |  | 1110 | 393505 | ＜0.01 |
|  |  | Age | 59.35 ± 7.01 | 57.24 ± 7.96 | ＜0.01 |
|  |  | Townsend deprivation index | -1.34 ± 3.01 | -1.49 ± 2.97 | 0.10 |
|  |  | Sex |  |  |  |
|  |  | Female | 995 (89.6%) | 214420 (54.5%) | ＜0.01 |
|  |  | Male | 115 (10.4%) | 179085 (45.5%) | ＜0.01 |
|  |  | Education |  |  |  |
|  |  | College or University | 797 (71.8%) | 271049 (68.9%) | 0.06 |
|  |  | Others | 292 (26.3%) | 115990 (29.5%) | 0.06 |
|  |  | NA | 21 (1.9%) | 6466 (1.6%) | 0.06 |
| Uveitis | H20, H30, H221 |  | 914 | 393701 |  |
|  |  | Age | 58.55 ± 7.53 | 57.24 ± 7.96 | ＜0.01 |
|  |  | Townsend deprivation index | -1.23 ± 3.15 | -1.49 ± 2.97 | 0.01 |
|  |  | Sex |  |  |  |
|  |  | Female | 470 (51.4%) | 214945 (54.6%) | 0.06 |
|  |  | Male | 444 (48.6%) | 178756 (45.4%) | 0.06 |
|  |  | Education |  |  |  |
|  |  | College or University | 656 (71.8%) | 271190 (68.9%) | 0.14 |
|  |  | Others | 242 (26.5%) | 116040 (29.5%) | 0.14 |
|  |  | NA | 16 (1.8%) | 6471 (1.6%) | 0.14 |
| Myopia | H521 |  | 3831 | 390784 |  |
|  |  | Age | 59.95 ± 6.70 | 57.22 ± 7.96 | ＜0.01 |
|  |  | Townsend deprivation index | -1.42 ± 3.01 | -1.49 ± 2.97 | 0.20 |
|  |  | Sex |  |  |  |
|  |  | Female | 2178 (56.9%) | 213237 (54.6%) | ＜0.01 |
|  |  | Male | 1653 (43.1%) | 177547 (45.4%) | ＜0.01 |
|  |  | Education |  |  | ＜0.01 |
|  |  | College or University | 2232 (58.3%) | 269614 (69.0%) | ＜0.01 |
|  |  | Others | 1535 (40.1%) | 114747 (29.4%) | ＜0.01 |
|  |  | NA | 64 (1.7%) | 6423 (1.6%) | ＜0.01 |
| Cataract | H25 |  | 33120 | 361495 | ＜0.01 |
|  |  | Age | 62.62 ± 5.42 | 56.75 ± 7.97 | ＜0.01 |
|  |  | Townsend deprivation index | -1.42 ± 2.98 | -1.49 ± 2.97 | ＜0.01 |
|  |  | Sex |  |  | ＜0.01 |
|  |  | Female | 19643 (59.3%) | 195772 (54.2%) | ＜0.01 |
|  |  | Male | 13477 (40.7%) | 165723 (45.8%) | ＜0.01 |
|  |  | Education |  |  | ＜0.01 |
|  |  | College or University | 24751 (74.7%) | 247095 (68.4%) | ＜0.01 |
|  |  | Others | 7764 (23.4%) | 108518 (30.0%) | ＜0.01 |
|  |  | NA | 605 (1.8%) | 5882 (1.6%) | ＜0.01 |
| PACG | H402 |  | 1778 | 392837 | ＜0.01 |
|  |  | Age | 61.18 ± 6.26 | 57.23 ± 7.96 | ＜0.01 |
|  |  | Townsend deprivation index | -1.32 ± 3.09 | -1.49 ± 2.97 | 0.03 |
|  |  | Sex |  |  |  |
|  |  | Female | 1153 (64.8%) | 214262 (54.5%) | ＜0.01 |
|  |  | Male | 625 (35.2%) | 178575 (45.5%) | ＜0.01 |
|  |  | Education |  |  | ＜0.01 |
|  |  | College or University | 1357 (76.3%) | 270489 (68.9%) | ＜0.01 |
|  |  | Others | 369 (20.8%) | 115913 (29.5%) | ＜0.01 |
|  |  | NA | 52 (2.9%) | 6435 (1.6%) | ＜0.01 |
| Keratitis | H16 |  | 600 | 394015 | ＜0.01 |
|  |  | Age | 58.91 ± 7.51 | 57.24 ± 7.96 | ＜0.01 |
|  |  | Townsend deprivation index | -1.25 ± 3.21 | -1.49 ± 2.97 | 0.07 |
|  |  | Sex |  |  |  |
|  |  | Female | 353 (58.8%) | 215062 (54.6%) | 0.04 |
|  |  | Male | 247 (41.2%) | 178953 (45.4%) | 0.04 |
|  |  | Education |  |  |  |
|  |  | College or University | 436 (72.7%) | 271410 (68.9%) | 0.05 |
|  |  | Others | 151 (25.2%) | 116131 (29.5%) | 0.05 |
|  |  | NA | 13 (2.2%) | 6474 (1.6%) | 0.05 |

**Table S17.** Significant results between 64 modifiable exposures and 40 trait pairs in UK Biobank

| **Trait-pairs** | **Catgory** | **Modifiable exposure** | **OR** | **CI_low** | **CI_up** | ***P*** | ***P_*FDR** |
| --- | --- | --- | --- | --- | --- | --- | --- |
| AMD-GORD | Antibiotic exposure | Long-term/recurrent antibiotics as child or teenager | 1.50 | 1.14 | 1.96 | 3.37E-03 | 6.31E-03 |
| AMD-GORD | Aspirin exposure | Medication for pain relief, constipation, heartburn: Aspirin | 1.41 | 1.26 | 1.58 | 1.91E-09 | 6.10E-09 |
| AMD-GORD | Diet | Cooked vegetable intake | 1.15 | 1.03 | 1.30 | 1.68E-02 | 2.83E-02 |
| AMD-GORD | Diet | Dried fruit intake | 0.96 | 0.93 | 0.99 | 8.32E-03 | 1.44E-02 |
| AMD-GORD | Lifestyles | Alcohol drinker status | 0.87 | 0.79 | 0.96 | 3.55E-03 | 6.31E-03 |
| AMD-GORD | Lifestyles | Smoking status | 1.22 | 1.13 | 1.31 | 6.84E-08 | 1.99E-07 |
| AMD-GORD | Lifestyles | Number of days/week walked 10+ minutes | 0.96 | 0.94 | 0.99 | 2.25E-03 | 4.51E-03 |
| AMD-GORD | Lifestyles | Number of days/week of moderate physical activity 10+ minutes | 0.95 | 0.93 | 0.96 | 9.94E-08 | 2.77E-07 |
| AMD-GORD | Lifestyles | Number of days/week of vigorous physical activity 10+ minutes | 0.94 | 0.91 | 0.96 | 2.70E-06 | 6.64E-06 |
| AMD-GORD | Lifestyles | Body mass index (BMI, Physical measures) | 1.05 | 1.04 | 1.06 | 1.83E-30 | 3.90E-29 |
| AMD-GORD | Lifestyles | Body mass index (BMI, Body composition) | 1.05 | 1.04 | 1.06 | 3.31E-30 | 5.29E-29 |
| AMD-GORD | Mental health | Mood swings | 1.56 | 1.42 | 1.71 | 4.39E-20 | 2.81E-19 |
| AMD-GORD | Mental health | Miserableness | 1.58 | 1.44 | 1.74 | 2.70E-21 | 1.92E-20 |
| AMD-GORD | Mental health | Irritability | 1.49 | 1.34 | 1.66 | 7.05E-14 | 2.90E-13 |
| AMD-GORD | Mental health | Sensitivity / hurt feelings | 1.46 | 1.32 | 1.62 | 7.26E-14 | 2.90E-13 |
| AMD-GORD | Mental health | Fed-up feelings | 1.62 | 1.47 | 1.78 | 2.96E-23 | 3.16E-22 |
| AMD-GORD | Mental health | Nervous feelings | 1.45 | 1.31 | 1.61 | 1.34E-12 | 4.77E-12 |
| AMD-GORD | Mental health | Worrier / anxious feelings | 1.43 | 1.30 | 1.58 | 1.46E-12 | 4.93E-12 |
| AMD-GORD | Mental health | Tense / 'highly strung' | 1.53 | 1.37 | 1.72 | 3.30E-13 | 1.24E-12 |
| AMD-GORD | Mental health | Worry too long after embarrassment | 1.34 | 1.21 | 1.47 | 3.17E-09 | 9.66E-09 |
| AMD-GORD | Mental health | Suffer from 'nerves' | 1.58 | 1.42 | 1.77 | 8.34E-17 | 4.11E-16 |
| AMD-GORD | Mental health | Loneliness, isolation | 1.60 | 1.43 | 1.79 | 1.11E-16 | 5.06E-16 |
| AMD-GORD | Mental health | Guilty feelings | 1.31 | 1.19 | 1.45 | 1.48E-07 | 3.96E-07 |
| AMD-GORD | Mental health | Seen doctor (GP) for nerves, anxiety, tension or depression | 1.82 | 1.65 | 2.00 | 9.72E-35 | 3.11E-33 |
| AMD-GORD | Mental health | Seen a psychiatrist for nerves, anxiety, tension or depression | 1.76 | 1.55 | 2.00 | 2.25E-18 | 1.31E-17 |
| AMD-GORD | Mental health | Ever depressed for a whole week | 1.38 | 1.17 | 1.62 | 1.02E-04 | 2.32E-04 |
| AMD-GORD | Mental health | Ever unenthusiastic/disinterested for a whole week | 1.36 | 1.15 | 1.61 | 2.90E-04 | 6.19E-04 |
| AMD-IBD | Diet | Mineral and other dietary supplements: Calcium | 2.23 | 1.54 | 3.24 | 2.58E-05 | 1.65E-03 |
| AMD-IBD | Lifestyles | Smoking status | 1.43 | 1.17 | 1.74 | 4.35E-04 | 1.39E-02 |
| AMD-PUD | Antibiotic exposure | Long-term/recurrent antibiotics as child or teenager | 2.85 | 1.69 | 4.80 | 8.24E-05 | 3.51E-04 |
| AMD-PUD | Aspirin exposure | Medication for pain relief, constipation, heartburn: Aspirin | 1.88 | 1.53 | 2.31 | 2.88E-09 | 4.61E-08 |
| AMD-PUD | Lifestyles | Smoking status | 1.33 | 1.16 | 1.53 | 6.91E-05 | 3.16E-04 |
| AMD-PUD | Lifestyles | Number of days/week of moderate physical activity 10+ minutes | 0.93 | 0.90 | 0.97 | 1.50E-03 | 5.65E-03 |
| AMD-PUD | Lifestyles | Number of days/week of vigorous physical activity 10+ minutes | 0.89 | 0.84 | 0.94 | 5.61E-05 | 2.76E-04 |
| AMD-PUD | Lifestyles | Body mass index (BMI, Physical measures) | 1.08 | 1.06 | 1.10 | 3.06E-18 | 1.70E-16 |
| AMD-PUD | Lifestyles | Body mass index (BMI, Body composition) | 1.08 | 1.06 | 1.10 | 5.30E-18 | 1.70E-16 |
| AMD-PUD | Mental health | Mood swings | 1.50 | 1.24 | 1.81 | 3.48E-05 | 2.19E-04 |
| AMD-PUD | Mental health | Miserableness | 1.50 | 1.24 | 1.81 | 3.76E-05 | 2.19E-04 |
| AMD-PUD | Mental health | Fed-up feelings | 1.54 | 1.27 | 1.86 | 1.22E-05 | 9.74E-05 |
| AMD-PUD | Mental health | Suffer from 'nerves' | 1.40 | 1.12 | 1.75 | 3.35E-03 | 1.13E-02 |
| AMD-PUD | Mental health | Loneliness, isolation | 1.52 | 1.21 | 1.91 | 3.51E-04 | 1.40E-03 |
| AMD-PUD | Mental health | Seen doctor (GP) for nerves, anxiety, tension or depression | 1.72 | 1.42 | 2.09 | 3.62E-08 | 3.86E-07 |
| AMD-PUD | Mental health | Seen a psychiatrist for nerves, anxiety, tension or depression | 1.70 | 1.31 | 2.19 | 5.34E-05 | 2.76E-04 |
| AMD-PUD | Mental health | Ever depressed for a whole week | 1.68 | 1.17 | 2.42 | 5.26E-03 | 1.60E-02 |
| AMD-PUD | Mental health | Ever unenthusiastic/disinterested for a whole week | 1.71 | 1.18 | 2.46 | 4.25E-03 | 1.36E-02 |
| AMD-IBS | Antibiotic exposure | Long-term/recurrent antibiotics as child or teenager | 2.97 | 1.94 | 4.55 | 5.19E-07 | 2.08E-06 |
| AMD-IBS | Diet | Mineral and other dietary supplements: Calcium | 1.68 | 1.28 | 2.19 | 1.60E-04 | 4.64E-04 |
| AMD-IBS | Lifestyles | Alcohol drinker status | 0.75 | 0.63 | 0.89 | 7.66E-04 | 1.89E-03 |
| AMD-IBS | Lifestyles | Body mass index (BMI, Physical measures) | 1.03 | 1.01 | 1.05 | 1.64E-03 | 3.61E-03 |
| AMD-IBS | Lifestyles | Body mass index (BMI, Body composition) | 1.03 | 1.01 | 1.05 | 6.96E-04 | 1.78E-03 |
| AMD-IBS | Mental health | Mood swings | 2.11 | 1.73 | 2.57 | 1.88E-13 | 1.72E-12 |
| AMD-IBS | Mental health | Miserableness | 1.69 | 1.39 | 2.06 | 1.59E-07 | 7.25E-07 |
| AMD-IBS | Mental health | Irritability | 1.67 | 1.35 | 2.06 | 2.16E-06 | 8.11E-06 |
| AMD-IBS | Mental health | Sensitivity / hurt feelings | 1.79 | 1.44 | 2.23 | 1.30E-07 | 6.39E-07 |
| AMD-IBS | Mental health | Fed-up feelings | 1.67 | 1.37 | 2.03 | 3.38E-07 | 1.44E-06 |
| AMD-IBS | Mental health | Nervous feelings | 1.97 | 1.61 | 2.41 | 4.43E-11 | 2.84E-10 |
| AMD-IBS | Mental health | Worrier / anxious feelings | 2.23 | 1.77 | 2.80 | 6.70E-12 | 5.36E-11 |
| AMD-IBS | Mental health | Tense / 'highly strung' | 2.06 | 1.66 | 2.56 | 7.58E-11 | 4.41E-10 |
| AMD-IBS | Mental health | Worry too long after embarrassment | 1.62 | 1.32 | 1.98 | 2.75E-06 | 9.79E-06 |
| AMD-IBS | Mental health | Suffer from 'nerves' | 2.22 | 1.80 | 2.74 | 1.30E-13 | 1.39E-12 |
| AMD-IBS | Mental health | Loneliness, isolation | 1.66 | 1.33 | 2.07 | 7.66E-06 | 2.45E-05 |
| AMD-IBS | Mental health | Guilty feelings | 1.53 | 1.25 | 1.87 | 3.47E-05 | 1.06E-04 |
| AMD-IBS | Mental health | Seen doctor (GP) for nerves, anxiety, tension or depression | 2.27 | 1.86 | 2.76 | 2.74E-16 | 5.84E-15 |
| AMD-IBS | Mental health | Seen a psychiatrist for nerves, anxiety, tension or depression | 2.26 | 1.78 | 2.87 | 1.71E-11 | 1.22E-10 |
| AMD-IBS | Mental health | Ever depressed for a whole week | 1.54 | 1.07 | 2.22 | 2.09E-02 | 4.06E-02 |
| AMD-IBS | Mental health | Ever unenthusiastic/disinterested for a whole week | 1.96 | 1.38 | 2.80 | 2.01E-04 | 5.37E-04 |
| AMD-IBS | Mental health | Severity of manic/irritable episodes | 4.06 | 1.71 | 9.66 | 1.51E-03 | 3.45E-03 |
| AMD-IBS | Mental health | Number of unenthusiastic/disinterested episodes | 1.00 | 1.00 | 1.01 | 8.87E-04 | 2.10E-03 |
| AMD-DD | Air Pollutants | Nitrogen dioxide air pollution | 1.01 | 1.00 | 1.01 | 3.20E-02 | 4.19E-02 |
| AMD-DD | Air Pollutants | Nitrogen oxides air pollution | 1.00 | 1.00 | 1.01 | 1.72E-02 | 2.34E-02 |
| AMD-DD | Air Pollutants | Particulate matter air pollution (pm10) | 1.03 | 1.01 | 1.06 | 6.89E-03 | 1.13E-02 |
| AMD-DD | Air Pollutants | Particulate matter air pollution (pm2.5) | 1.09 | 1.04 | 1.14 | 1.10E-04 | 2.82E-04 |
| AMD-DD | Antibiotic exposure | Long-term/recurrent antibiotics as child or teenager | 1.84 | 1.46 | 2.31 | 1.88E-07 | 6.67E-07 |
| AMD-DD | Aspirin exposure | Medication for pain relief, constipation, heartburn: Aspirin | 1.47 | 1.33 | 1.62 | 1.82E-14 | 1.29E-13 |
| AMD-DD | Lifestyles | Exposure to tobacco smoke outside home | 1.16 | 1.04 | 1.28 | 6.74E-03 | 1.13E-02 |
| AMD-DD | Diet | Cooked vegetable intake | 1.19 | 1.06 | 1.32 | 1.90E-03 | 3.80E-03 |
| AMD-DD | Diet | Dried fruit intake | 0.97 | 0.94 | 0.99 | 1.71E-02 | 2.34E-02 |
| AMD-DD | Lifestyles | Alcohol drinker status | 0.89 | 0.82 | 0.97 | 8.91E-03 | 1.43E-02 |
| AMD-DD | Lifestyles | Smoking status | 1.29 | 1.21 | 1.38 | 4.47E-15 | 3.58E-14 |
| AMD-DD | Lifestyles | Number of days/week walked 10+ minutes | 0.95 | 0.93 | 0.97 | 2.69E-06 | 7.82E-06 |
| AMD-DD | Lifestyles | Number of days/week of moderate physical activity 10+ minutes | 0.96 | 0.94 | 0.98 | 1.90E-05 | 5.29E-05 |
| AMD-DD | Lifestyles | Number of days/week of vigorous physical activity 10+ minutes | 0.95 | 0.93 | 0.98 | 4.62E-05 | 1.23E-04 |
| AMD-DD | Lifestyles | Body mass index (BMI, Physical measures) | 1.05 | 1.04 | 1.06 | 2.15E-35 | 4.59E-34 |
| AMD-DD | Lifestyles | Body mass index (BMI, Body composition) | 1.05 | 1.05 | 1.06 | 1.18E-35 | 3.77E-34 |
| AMD-DD | Mental health | Mood swings | 1.38 | 1.27 | 1.50 | 1.43E-13 | 9.13E-13 |
| AMD-DD | Mental health | Miserableness | 1.33 | 1.22 | 1.45 | 1.06E-10 | 4.82E-10 |
| AMD-DD | Mental health | Irritability | 1.35 | 1.22 | 1.48 | 1.19E-09 | 5.07E-09 |
| AMD-DD | Mental health | Sensitivity / hurt feelings | 1.29 | 1.18 | 1.41 | 1.29E-08 | 5.15E-08 |
| AMD-DD | Mental health | Fed-up feelings | 1.42 | 1.31 | 1.55 | 1.35E-15 | 1.44E-14 |
| AMD-DD | Mental health | Nervous feelings | 1.19 | 1.08 | 1.31 | 6.29E-04 | 1.34E-03 |
| AMD-DD | Mental health | Worrier / anxious feelings | 1.25 | 1.14 | 1.36 | 9.29E-07 | 2.83E-06 |
| AMD-DD | Mental health | Tense / 'highly strung' | 1.32 | 1.18 | 1.47 | 6.16E-07 | 2.08E-06 |
| AMD-DD | Mental health | Worry too long after embarrassment | 1.14 | 1.04 | 1.24 | 3.58E-03 | 6.19E-03 |
| AMD-DD | Mental health | Suffer from 'nerves' | 1.30 | 1.17 | 1.44 | 8.11E-07 | 2.60E-06 |
| AMD-DD | Mental health | Loneliness, isolation | 1.36 | 1.22 | 1.51 | 2.12E-08 | 7.98E-08 |
| AMD-DD | Mental health | Guilty feelings | 1.16 | 1.05 | 1.27 | 2.52E-03 | 4.74E-03 |
| AMD-DD | Mental health | Risk taking | 1.20 | 1.08 | 1.33 | 4.17E-04 | 9.20E-04 |
| AMD-DD | Mental health | Seen doctor (GP) for nerves, anxiety, tension or depression | 1.62 | 1.48 | 1.76 | 2.36E-27 | 3.77E-26 |
| AMD-DD | Mental health | Seen a psychiatrist for nerves, anxiety, tension or depression | 1.61 | 1.43 | 1.82 | 2.22E-15 | 2.03E-14 |
| AMD-DD | Mental health | Ever depressed for a whole week | 1.34 | 1.15 | 1.56 | 1.39E-04 | 3.35E-04 |
| AMD-DD | Mental health | Ever unenthusiastic/disinterested for a whole week | 1.35 | 1.16 | 1.58 | 1.72E-04 | 3.93E-04 |
| AMD-DD | Mental health | Ever manic/hyper for 2 days | 1.44 | 1.04 | 2.01 | 2.96E-02 | 3.95E-02 |
| AMD-DD | Mental health | Length of longest manic/irritable episode | 1.96 | 1.26 | 3.04 | 2.61E-03 | 4.77E-03 |
| AMD-DD | Mental health | Severity of manic/irritable episodes | 1.69 | 1.10 | 2.58 | 1.63E-02 | 2.32E-02 |
| DR-GORD | Antibiotic exposure | Long-term/recurrent antibiotics as child or teenager | 1.94 | 1.24 | 3.04 | 3.68E-03 | 6.74E-03 |
| DR-GORD | Aspirin exposure | Medication for pain relief, constipation, heartburn: Aspirin | 5.12 | 4.40 | 5.96 | 2.20E-98 | 4.69E-97 |
| DR-GORD | Diet | Cooked vegetable intake | 1.36 | 1.14 | 1.63 | 7.86E-04 | 1.68E-03 |
| DR-GORD | Diet | Fresh fruit intake | 1.07 | 1.03 | 1.10 | 2.27E-04 | 5.00E-04 |
| DR-GORD | Diet | Dried fruit intake | 0.89 | 0.83 | 0.94 | 1.12E-04 | 2.56E-04 |
| DR-GORD | Lifestyles | Alcohol drinker status | 0.65 | 0.58 | 0.74 | 6.71E-11 | 2.39E-10 |
| DR-GORD | Lifestyles | Smoking status | 1.23 | 1.11 | 1.36 | 9.98E-05 | 2.37E-04 |
| DR-GORD | Lifestyles | Number of days/week walked 10+ minutes | 0.87 | 0.84 | 0.90 | 1.14E-14 | 6.07E-14 |
| DR-GORD | Lifestyles | Number of days/week of moderate physical activity 10+ minutes | 0.89 | 0.86 | 0.92 | 7.51E-13 | 3.43E-12 |
| DR-GORD | Lifestyles | Number of days/week of vigorous physical activity 10+ minutes | 0.87 | 0.83 | 0.91 | 1.81E-10 | 6.10E-10 |
| DR-GORD | Lifestyles | Body mass index (BMI, Physical measures) | 1.14 | 1.13 | 1.15 | 9.59E-140 | 6.14E-138 |
| DR-GORD | Lifestyles | Body mass index (BMI, Body composition) | 1.14 | 1.13 | 1.16 | 1.30E-137 | 4.16E-136 |
| DR-GORD | Mental health | Mood swings | 1.79 | 1.55 | 2.08 | 6.46E-15 | 3.76E-14 |
| DR-GORD | Mental health | Miserableness | 1.54 | 1.33 | 1.78 | 7.07E-09 | 2.16E-08 |
| DR-GORD | Mental health | Irritability | 1.61 | 1.38 | 1.88 | 1.40E-09 | 4.49E-09 |
| DR-GORD | Mental health | Sensitivity / hurt feelings | 1.40 | 1.21 | 1.63 | 8.53E-06 | 2.37E-05 |
| DR-GORD | Mental health | Fed-up feelings | 1.90 | 1.64 | 2.20 | 1.26E-17 | 8.97E-17 |
| DR-GORD | Mental health | Tense / 'highly strung' | 1.34 | 1.11 | 1.60 | 1.79E-03 | 3.37E-03 |
| DR-GORD | Mental health | Loneliness, isolation | 1.74 | 1.48 | 2.05 | 4.62E-11 | 1.85E-10 |
| DR-GORD | Mental health | Seen doctor (GP) for nerves, anxiety, tension or depression | 1.85 | 1.60 | 2.14 | 1.61E-16 | 1.03E-15 |
| DR-GORD | Mental health | Seen a psychiatrist for nerves, anxiety, tension or depression | 1.74 | 1.44 | 2.10 | 8.37E-09 | 2.44E-08 |
| DR-GORD | Mental health | Ever depressed for a whole week | 1.45 | 1.12 | 1.86 | 4.28E-03 | 7.60E-03 |
| DR-GORD | Mental health | Ever unenthusiastic/disinterested for a whole week | 1.54 | 1.19 | 1.98 | 9.25E-04 | 1.85E-03 |
| DR-GORD | Mental health | Ever manic/hyper for 2 days | 1.73 | 1.12 | 2.68 | 1.43E-02 | 2.41E-02 |
| DR-GORD | Mental health | Ever highly irritable/argumentative for 2 days | 1.81 | 1.36 | 2.41 | 5.20E-05 | 1.28E-04 |
| DR-GORD | Mental health | Number of depression episodes | 1.00 | 1.00 | 1.01 | 9.86E-03 | 1.71E-02 |
| DR-IBD | Aspirin exposure | Medication for pain relief, constipation, heartburn: Aspirin | 6.19 | 4.05 | 9.48 | 4.48E-17 | 2.87E-15 |
| DR-IBD | Diet | Mineral and other dietary supplements: Calcium | 2.88 | 1.60 | 5.20 | 4.35E-04 | 3.09E-03 |
| DR-IBD | Lifestyles | Alcohol drinker status | 0.58 | 0.42 | 0.82 | 1.68E-03 | 8.97E-03 |
| DR-IBD | Lifestyles | Number of days/week of vigorous physical activity 10+ minutes | 0.86 | 0.76 | 0.97 | 1.19E-02 | 4.47E-02 |
| DR-IBD | Lifestyles | Body mass index (BMI, Physical measures) | 1.11 | 1.08 | 1.15 | 3.70E-11 | 1.19E-09 |
| DR-IBD | Lifestyles | Body mass index (BMI, Body composition) | 1.11 | 1.08 | 1.15 | 1.65E-10 | 3.51E-09 |
| DR-IBD | Mental health | Miserableness | 1.70 | 1.13 | 2.57 | 1.18E-02 | 4.47E-02 |
| DR-IBD | Mental health | Fed-up feelings | 1.79 | 1.18 | 2.71 | 5.89E-03 | 2.51E-02 |
| DR-IBD | Mental health | Seen a psychiatrist for nerves, anxiety, tension or depression | 2.13 | 1.29 | 3.51 | 3.01E-03 | 1.48E-02 |
| DR-PUD | Aspirin exposure | Medication for pain relief, constipation, heartburn: Aspirin | 5.21 | 4.06 | 6.68 | 1.94E-38 | 4.15E-37 |
| DR-PUD | Diet | Fresh fruit intake | 1.09 | 1.04 | 1.14 | 1.35E-04 | 4.78E-04 |
| DR-PUD | Diet | Dried fruit intake | 0.86 | 0.78 | 0.96 | 6.71E-03 | 1.65E-02 |
| DR-PUD | Diet | Salad / raw vegetable intake | 0.91 | 0.84 | 0.98 | 9.73E-03 | 2.22E-02 |
| DR-PUD | Lifestyles | Alcohol drinker status | 0.50 | 0.42 | 0.60 | 4.57E-14 | 4.18E-13 |
| DR-PUD | Lifestyles | Number of days/week walked 10+ minutes | 0.89 | 0.84 | 0.95 | 1.10E-04 | 4.13E-04 |
| DR-PUD | Lifestyles | Number of days/week of moderate physical activity 10+ minutes | 0.90 | 0.86 | 0.95 | 1.68E-04 | 5.65E-04 |
| DR-PUD | Lifestyles | Number of days/week of vigorous physical activity 10+ minutes | 0.84 | 0.78 | 0.90 | 2.52E-06 | 1.24E-05 |
| DR-PUD | Lifestyles | Body mass index (BMI, Physical measures) | 1.14 | 1.12 | 1.16 | 2.80E-54 | 1.79E-52 |
| DR-PUD | Lifestyles | Body mass index (BMI, Body composition) | 1.15 | 1.13 | 1.17 | 1.09E-53 | 3.48E-52 |
| DR-PUD | Mental health | Mood swings | 2.04 | 1.59 | 2.60 | 1.36E-08 | 1.09E-07 |
| DR-PUD | Mental health | Miserableness | 1.51 | 1.18 | 1.92 | 8.83E-04 | 2.57E-03 |
| DR-PUD | Mental health | Irritability | 1.71 | 1.34 | 2.20 | 2.29E-05 | 1.05E-04 |
| DR-PUD | Mental health | Sensitivity / hurt feelings | 1.36 | 1.07 | 1.74 | 1.32E-02 | 2.91E-02 |
| DR-PUD | Mental health | Fed-up feelings | 1.97 | 1.55 | 2.51 | 4.08E-08 | 2.61E-07 |
| DR-PUD | Mental health | Tense / 'highly strung' | 1.49 | 1.11 | 2.00 | 8.52E-03 | 2.02E-02 |
| DR-PUD | Mental health | Loneliness, isolation | 2.07 | 1.59 | 2.69 | 6.38E-08 | 3.71E-07 |
| DR-PUD | Mental health | Seen doctor (GP) for nerves, anxiety, tension or depression | 1.55 | 1.21 | 1.98 | 4.73E-04 | 1.51E-03 |
| DR-PUD | Mental health | Seen a psychiatrist for nerves, anxiety, tension or depression | 1.64 | 1.20 | 2.26 | 2.12E-03 | 5.91E-03 |
| DR-PUD | Mental health | Ever highly irritable/argumentative for 2 days | 2.11 | 1.25 | 3.56 | 5.47E-03 | 1.40E-02 |
| DR-IBS | Aspirin exposure | Medication for pain relief, constipation, heartburn: Aspirin | 6.37 | 4.62 | 8.77 | 1.02E-29 | 2.18E-28 |
| DR-IBS | Diet | Dried fruit intake | 0.85 | 0.73 | 0.97 | 1.94E-02 | 3.88E-02 |
| DR-IBS | Lifestyles | Alcohol drinker status | 0.62 | 0.48 | 0.79 | 1.39E-04 | 4.06E-04 |
| DR-IBS | Lifestyles | Number of days/week walked 10+ minutes | 0.82 | 0.76 | 0.88 | 1.62E-08 | 9.44E-08 |
| DR-IBS | Lifestyles | Number of days/week of moderate physical activity 10+ minutes | 0.87 | 0.81 | 0.93 | 7.26E-05 | 2.21E-04 |
[truncated: 55,413 more chars]
